# Circuits with broken fibration symmetries perform core logic computations in biological networks

**DOI:** 10.1371/journal.pcbi.1007776

**Published:** 2020-06-17

**Authors:** Ian Leifer, Flaviano Morone, Saulo D. S. Reis, José S. Andrade, Mariano Sigman, Hernán A. Makse

**Affiliations:** 1 Levich Institute and Physics Department, City College of New York, New York, New York, United States of America; 2 Departamento de Física, Universidade Federal do Ceará, Fortaleza, Ceará, Brazil; 3 Laboratorio de Neurociencia, Universidad Torcuato Di Tella, Buenos Aires, Argentina; 4 CONICET (Consejo Nacional de Investigaciones Científicas y Tecnicas), Argentina; 5 Facultad de Lenguas y Educacion, Universidad Nebrija, Madrid, Spain; University of Chicago, UNITED STATES

## Abstract

We show that logic computational circuits in gene regulatory networks arise from a fibration symmetry breaking in the network structure. From this idea we implement a constructive procedure that reveals a hierarchy of genetic circuits, ubiquitous across species, that are surprising analogues to the emblematic circuits of solid-state electronics: starting from the transistor and progressing to ring oscillators, current-mirror circuits to toggle switches and flip-flops. These canonical variants serve fundamental operations of synchronization and clocks (in their symmetric states) and memory storage (in their broken symmetry states). These conclusions introduce a theoretically principled strategy to search for computational building blocks in biological networks, and present a systematic route to design synthetic biological circuits.

## Author summary

We show that the core functional logic of genetic circuits arises from a fundamental symmetry breaking of the interactions of the biological network. The idea can be put into a hierarchy of symmetric genetic circuits that reveals their logical functions. We do so through a constructive procedure that naturally reveals a series of building blocks, widely present across species. This hierarchy maps to a progression of fundamental units of electronics, starting with the transistor, progressing to ring oscillators and current-mirror circuits and then to synchronized clocks, switches and finally to memory devices such as latches and flip-flops.

## Introduction

In all biological networks [1] some simple ‘network motifs’ appear more often than they would by pure chance [2–4]. This regularity has been interpreted as evidence that these motifs are basic building blocks of biological machineries, a proposal that has had a major impact on systems biology [4, 5]. However, mere statistical abundance by itself does not imply that these circuits are core bricks of biological systems. In fact, whether these network motifs may have a functional role in biological computation remains controversial [6–9].

Functional building blocks should offer computational repertoires drawing parallels between biological networks and electronic circuits [10]. Indeed, the idea of using electronic circuitry and devices to mimic aspects of gene regulatory networks has been in circulation almost since the inception of regulatory genetics itself [11]. This idea has been a driving force in synthetic biology [12]; with several demonstrations showing that engineered biological circuits can perform computations [13], such as toggle switches [14–17], logic [18], memory storage [15, 19, 20], pulse generators and oscillators [14, 21–23].

In a previous work [24], we have shown that the building blocks of gene regulatory networks can be identified by the fibration symmetries of these networks. In the present paper, we first demonstrate the functionality of these symmetric building blocks in terms of synchronized biological clocks. We then show that the breaking of the fibration symmetries of the network identifies additional building blocks with core logic computational functions. We do so through a constructive procedure based on symmetry breaking that naturally reveals a hierarchy of genetic building blocks widely present across biological networks and species. This hierarchy maps to a progression of fundamental units of electronics, starting with the transistor, and progressing to ring oscillators and current-mirror circuits and then to memory devices such as toggle switches and flip-flops. We show that, while symmetric circuits work as synchronized oscillators, the breaking of these symmetries plays a fundamental role by switching the functionality of circuits from synchronized clocks to memory units.

Thus, our constructive theoretical framework identifies 1) building blocks of genetic networks in a unified way from fundamental symmetry and broken symmetry principles, which 2) assure that they perform core logic computations, 3) suggests a natural mapping onto the foundational circuits of solid state electronics [12, 13], and 4) endows the mathematical notion of symmetry fibration [24] with biological significance.

## Results

### Feed-forward loops do not synchronize

**Fig 1:**
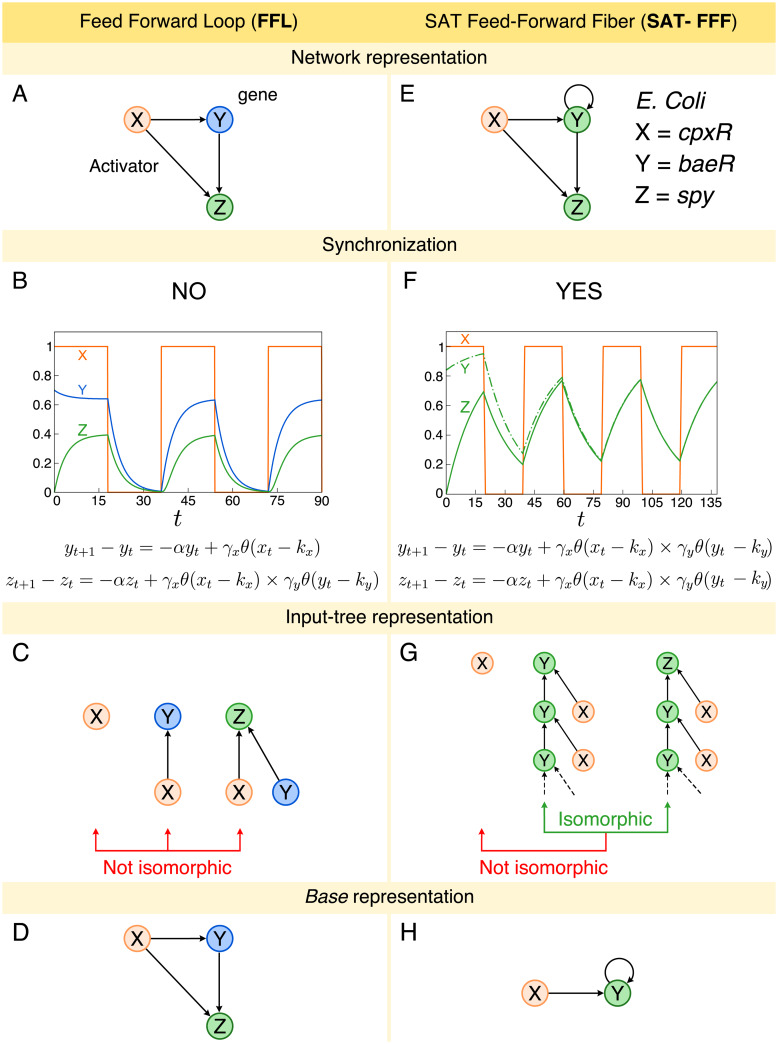
Feed-Forward Loop (FFL) and Feed-Forward Fiber (FFF). A: FFL network representation. B: Numerical solution of FFL dynamics. The expression levels of genes Y and Z do not synchronize. The oscillation pattern presented is due to the square-wave behavior of gene X expression levels. We use *α* = 2.0, *γ*_*x*_ = 0.12, *γ*_*y*_ = 0.7, *k*_*x*_ = 0.5, *k*_*y*_ = 0.1, *y*_0_ = 0.7 and *z*_0_ = 0.0. C: Input tree representation of FFL. The input trees of genes X, Y, and Z are not isomorphic, as a consequence, their expression levels do not synchronize. D: Base representation of FFL. The *base* is the same as the original circuits since there are no symmetries. E: SAT-FFF network representation. As an example, we consider genes *cpxR* = X, *baeR* = Y, and *spy* = Z from the gene regulatory network of *E. coli*. The addition of the autoregulation leads to a symmetry between the expression levels of genes Y and Z. F: The numerical solution of the SAT-FFF dynamics shows the synchronization of the expression levels of genes Y and Z. We use *α* = 0.06, *γ*_*x*_ = 0.775, *γ*_*y*_ = 0.775, *k*_*x*_ = 0.5, *k*_*y*_ = 0.1, *y*_0_ = 0.85 and *z*_0_ = 0.0. Again, the oscillation is due to the wave-like pattern of X. G: As a result, genes Y and Z have isomorphic input trees. However, the input tree of the external regulator *cpxR* is not isomorphic, despite the fact that it directly regulates the *fiber*. H: Since Y and Z synchronize, gene Z can be collapsed into Y, resulting in a simpler *base* representation.

We start our analysis by considering the dynamics of the most abundant network motif in transcriptional regulatory networks, the so-called feed-forward loop (FFL) introduced by Alon and coworkers [3, 4, 25]. A cFFL motif consists of three genes (X, Y and Z; c refers to coherent where all regulators are activators) where the transcription factor expressed by gene X positively regulates the transcription of Y and Z, and, in turn Y regulates Z (Fig 1A). Fig 1A shows an example of cFFL motif in *E. coli* with X = *cpxR*, Y = *baeR*, and Z = *spy*. Numerical and analytic solutions for the expression levels of the genes in the cFFL (Fig 1B) demonstrate that the FFL does not reach synchronization (unless for very specific setting of parameters) nor oscillations in expression levels. This is consistent with previous research which has interpreted the functionality of the FFL as signal delay [3, 25, 26].

We illustrate this result by presenting an analytical solution of the FFL [2–4]. We use a discrete time, continuous state variable model with a logic Boolean interaction function in the spirit of the Glass and Kauffman model of biochemical networks [27]. The dynamics of the expression levels *y*_*t*_ and *z*_*t*_ of genes Y and Z, respectively, as a function of time *t* in the cFFL is given by the following difference equations [4]:

$$\begin{array}{c} \begin{array}{cc} y_{t+1} & =\left(1-\alpha \right)y_{t}+\gamma_{x}\theta \left(x_{t}-k_{x}\right),\\ z_{t+1} & =\left(1-\alpha \right)z_{t}+\gamma_{x}\theta \left(x_{t}-k_{x}\right)\times \gamma_{y}\theta \left(y_{t}-k_{y}\right), \end{array} \end{array}$$ (1)

where *x*_*t*_ is the expression level of gene X, *α* is the degradation rate of the gene, *γ*_*x*_ and *γ*_*y*_ are the strength of the interaction representing the maximum expression rate of genes X and Y, respectively, and the thresholds *k*_*x*_ and *k*_*y*_ are the dissociation constant between the transcription factor and biding site. The expression level is measured in terms of abundance of gene product, e.g., mRNA concentration. The Heaviside step functions *θ*(*x*_*t*_ − *k*_*x*_) and *θ*(*y*_*t*_ − *k*_*y*_) represent the activator regulation from gene X and Y, respectively. They represent the Boolean logic approximation of Hill input functions in the limit of strong cooperativity [4, 27]. We consider an AND gate for the combined interaction of transcription factors of genes X and Y onto the binding sites of gene Z [4]. Analogous results shown in S1 File Section I can be obtained with an OR gate and with ODE continuum models.

In S1 File Sec. I A, we show that the expression levels of the genes Y and Z do not synchronize, in other words, *lim*_*t* → ∞_(*y*_*t*_ − *z*_*t*_) ≠ 0. For example, Fig 1B shows a particular set of parameters which results in a non-synchronized state. Such state is obtained under initial condition *y*_0_ > *k*_*y*_ and *α* < *γ*_*x*_. Specifically, we use the parameters: *α* = 0.2, *γ*_*x*_ = 0.12, *γ*_*y*_ = 0.7, *k*_*x*_ = 0.5 and *k*_*y*_ = 0.1. For this combination, *y*_*t*_ and *z*_*t*_ do not synchronize since *y*_*t*_ saturates at *y*_*t*_ → *γ*_*x*_/*α* = 0.6 when *t* → ∞, and *z*_*t*_ saturates at *z*_*t*_ → *γ*_*x*_*γ*_*y*_/*α* = 0.42, for *t* → ∞. In this figure, we set *x*_*t*_ equal to a square wave and then monitor the expression levels of *y*_*t*_ and *x*_*t*_. When *x* < *k*_*x*_, both *y*_*t*_ and *z*_*t*_ decay exponentially to zero. On the other hand, when *x* > *k*_*x*_, both variables evolve to saturate again at *y*_*t*_ = *γ*_*x*_/*α* and *z*_*t*_ = *γ*_*x*_*γ*_*y*_/*α*, in agreement with the analytical solution.

### Feed-forward fibers synchronize via a symmetry fibration

In the FFL, gene Z receives input from X and Y, while gene Y, instead, only from X, and therefore the inputs are not symmetric (Fig 1C). But as it turns out, a search of motifs in biological networks [24] shows that the FFL circuit in Fig 1A regularly appears in conjunction with an autoregulation (AR) loop [28] at Y = *baeR* (Fig 1E). This minimal inclusion in the FFL symmetrizes the circuit and we show, next, that this symmetry results in a circuit with a first and minimal form of function: synchronization in gene expression. This can be formalized by analyzing invariances in the input tree [24], which represents all the paths that converge to a given gene (Fig 1C and 1G). The mathematical principle of symmetry fibration in dynamical systems introduced in [24, 29, 30] predicts this, since symmetries Y ↔ Z that leave invariant the tree of inputs (but not necessarily the outputs as imposed by automorphisms) are necessary and sufficient to achieve synchronization. When two genes have isomorphic input trees, their expression levels are synchronized, see [24] for details.

For instance, by means of its autoregulation, *baeR* forms a symmetry fibration with gene *spy*, that can be formalized and characterized by an isomorphism between the input trees of these genes (Fig 1G). When two input trees, like those of *baeR* and *spy*, are isomorphic, the expression levels of these two genes are synchronized. These genes are said to belong to the same *‘fiber’* [24, 30]. Genes in the same *fiber* are redundant, and can be collapsed into the ‘*base*’ (see Fig 1H) by a symmetry fibration [24] (the FFL, instead, cannot be reduced since it has no symmetry, see Fig 1D). Numerical simulations and analytical solutions (Fig 1F, S1 File Section II A and Section II B) confirm that the addition of the AR loop to the FFL—leading to a circuit that we call the Feed-Forward Fiber (FFF)—changes its functionality qualitatively, leading to synchronization of genes Y and Z into coherent co-expression. This prediction is confirmed with experimental co-expression profiles in Ref. [24].

The FFF with activator regulations in Fig 1E has a simple dynamics converging to a synchronized fixed point: all interactions are satisfied meaning that the Heaviside step functions evaluate to 1 and we call this circuit SAT-FFF. Instead, when the autoregulation is a repressor, the loop behaves as a logical NOT gate. When expression is high it inhibits itself shifting to low state. Instead, if it is low it promotes itself to shift to a high state. Hence, the activity of this gene oscillates indefinitely [14, 27]. This is the simplest expression of *frustration* [27, 31], a core concept in physics which refers to a system which is always in tension and thus never reaches a stable fixed configuration.

### A biological transistor as a core building block

**Fig 2:**
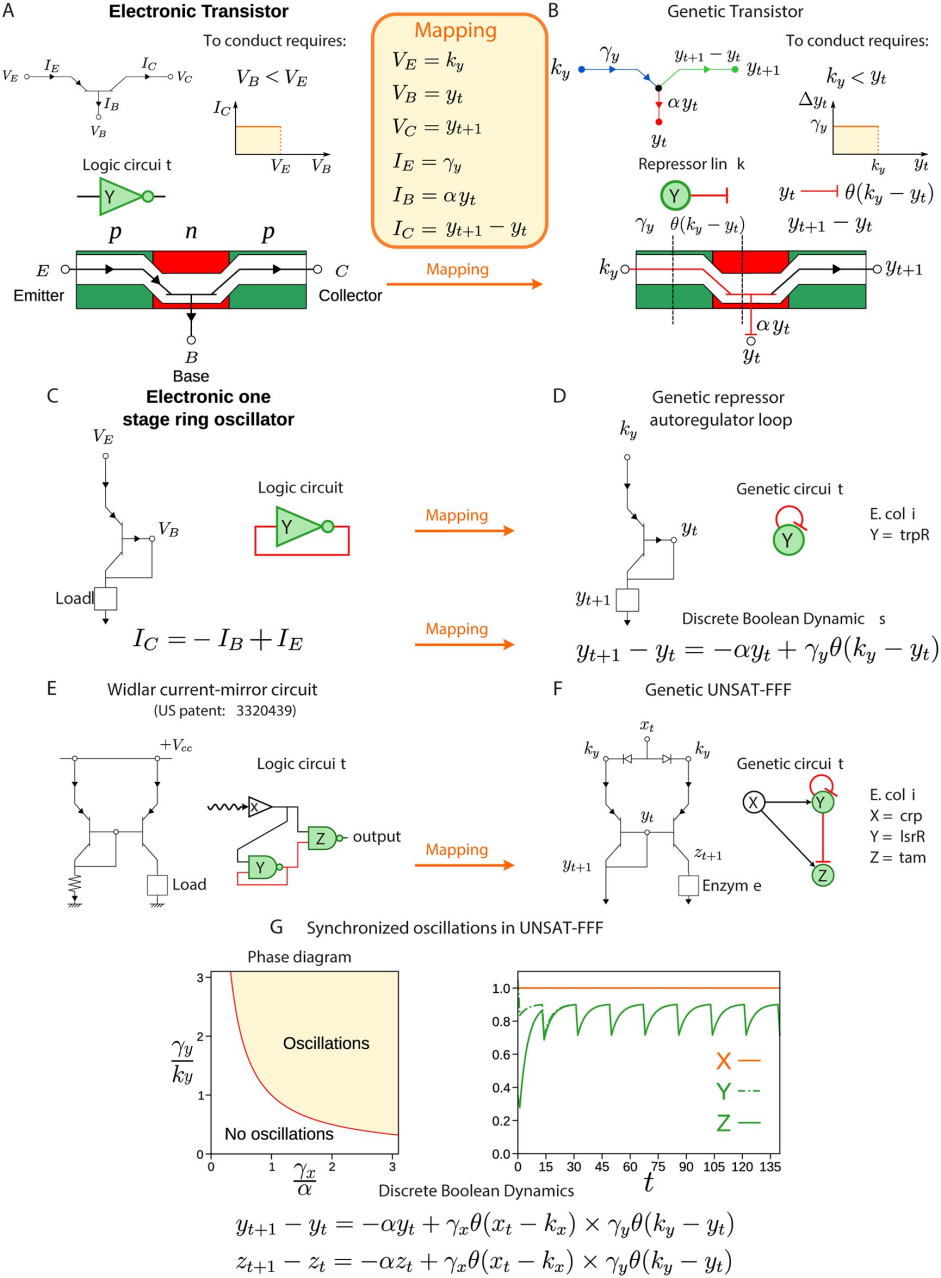
Mapping between electronic and biological transistor. A: A pnp transistor allows current flow if the voltage applied to its base is lower than the voltage at its emitter (*V*_*B*_ < *V*_*E*_). Since it has a high (low) output for a lower (high) input, it is logically represented by a NOT gate. The yellow box shows the mapping between the pnp transistor and the biological repressor. B: A repressor regulation link plays the role of the pnp transistor since the rate of expression of a gene is repressed by gene Y if *k*_*y*_ < *y*_*t*_. C: By connecting the base of the transistor to its collector, one forms a one stage ring oscillator. D: This connection is translated to the biological analogue as a repressor autoregulation at gene Y. In this way, the rate expression of gene Y is able to oscillate, depending on the adjustment of parameters *α*, *k*_*y*_ and *γ*_*y*_ (see S1 File Section III). E: Widlar current-mirror circuit and F: its biological analogue (UNSAT-FFF). By mirroring the ring oscillator, the Widlar mirror circuit allows synchronization and oscillations (see S1 File Section III). G: Phase diagram of oscillations of the UNSAT-FFF. An oscillatory phase is defined by the condition *γ*_*y*_/*k*_*y*_ > (*γ*_*x*_/*α*)^−1^ (see S1 File Section III). For instance, on the right side, we plot the solution of the discrete dynamics for a set of parameters satisfying such condition. Specifically, *α* = 0.205, *γ*_*x*_ = 0.454, *γ*_*y*_ = 0.454, *k*_*x*_ = 0.5, and *k*_*y*_ = 1.0.

To understand the computation rationale of symmetric and frustrated circuits made of repressors, we map them to electronic analogues. We begin the analogy with the simplest circuit of a single gene with a feedback loop with repression (AR loop, Fig 2D). The dynamics for the expression level *y*_*t*_ is described by the discrete time model with Boolean interaction [27]:

$$\begin{array}{c} \Delta y_{t}=y_{t+1}-y_{t}=-\alpha y_{t}+\gamma_{y}\theta \left(k_{y}-y_{t}\right). \end{array}$$ (2)

Here, *α* is the degradation rate of the Y gene product, *γ*_*y*_ is the maximum expression rate of gene Y, and *k*_*y*_ is the dissociation constant.

The Heaviside step function *θ*(*k*_*y*_ − *y*_*t*_) reflects the repressor autoregulation in the Boolean logic approximation. We will show that this genetic repressor interaction, shown as the stub in Fig 2B, is the genetic analogue of a solid-state transistor shown in Fig 2A.

A transistor is typically made up of three semiconductors, a base sandwiched between an emitter and a collector (Fig 2A). The current flows between the emitter and collector only if voltage applied to the base is lower than at the emitter (*V*_*B*_ < *V*_*E*_) and thus the transistor acts as a switch and inverter. In the genetic circuit, the expression *y*_*t*_ drives the rate of expression of gene Y, like the voltage drives current around an electric circuit. Simply comparing Eq (2) to the pnp transistor in Fig 2A leads to the analogy in which the expression *y*_*t*_ is an analogue for the base potential *V*_*B*_ of a transistor, *k*_*y*_ an analogue for *V*_*E*_, *γ*_*y*_ an analogue for the emitter current *I*_*E*_, *αy*_*t*_ an analogue for *I*_*B*_, and Δ*y*_*t*_ an analogue for *I*_*C*_. Then, Eq (2) provides the genetic equivalent of the equation for a transistor’s collector current *I*_*C*_ = *I*_*E*_ − *I*_*B*_ (Fig 2C and 2D).

The repressor AR genetic circuit of Fig 2D becomes a one-stage ring oscillator (Fig 2C) where the collector of the transistor connects to the base forming the minimal signal feedback loop. As shown in Fig 2D, an example of the AR is the gene Y = *trpR* from the *E. coli* transcriptional network. The repressor AR loop can be extended to the FFF by symmetrizing it, adding gene Z, such that it synchronizes with Y to express an enzyme that catalyzes a biochemical reaction (Fig 2F). The circuit is completed with the external regulator X which keeps the symmetry between Y and Z. The resulting circuit is called UNSAT-FFF (since it is frustrated).

The UNSAT-FFF maps to the so-called Widlar current-mirror electronic circuit shown in Fig 2E, a popular building block of integrated circuits used since the foundations of the semiconductor industry (1967 US patent [32, 33]). It serves two key functions as we show below: mirror synchronization of *y*_*t*_ = *z*_*t*_ and oscillatory activity (Fig 2G and S1 File Section III).

### UNSAT-FFF solution oscillates and synchronize

Next, we show analytically and numerically that the UNSAT-FFF circuit has an oscillatory solution plus synchronization of genes Y and Z. S1 File Section III A shows that the prediction of oscillatory behavior can be obtained also from a model of gene expression in the continuum time-domain using a first-order ODE model with time delay due to the process of transcription and translation. Below, we focus on the discrete dynamics.

The UNSAT-FFF circuit is obtained by the addition of a repressor AR loop to the FFL: in Fig 2F, gene Y acts as a repressor regulator on the gene Z and on itself. There are many variants of this circuit depending on the combinations of activator and repressor regulators. Here, we use X gene as an activator and Y gene as a repressor. Different variants yields analogous results and will be discussed elsewhere. The important ingredient is the existence of frustration in the interactions. For instance if gene X is high, then it will make genes Y and Z high too. However, this configuration does not satisfy the repressor autoregulation bond neither the repression from Y → Z. Thus, two bonds are unsatisfied and this circuit unsatisfied: UNSAT-FFF. The discrete-time dynamics of the expression levels of genes *y*_*t*_ and *z*_*t*_ are given by:

$$\begin{array}{c} \begin{array}{cc} y_{t+1} & =\left(1-\alpha \right)y_{t}+\gamma_{x}\theta \left(x_{t}-k_{x}\right)\times \gamma_{y}\theta \left(k_{y}-y_{t}\right)\;,\\ z_{t+1} & =\left(1-\alpha \right)z_{t}+\gamma_{x}\theta \left(x_{t}-k_{x}\right)\times \gamma_{y}\theta \left(k_{y}-y_{t}\right)\;, \end{array} \end{array}$$ (3)

where *γ*_*x*_ and *γ*_*y*_ are the strength of the interaction (maximum expression rate) of genes *X* and *Y*, respectively, and *k*_*x*_ and *k*_*y*_ are the dissociation constant, respectively. Similarly to the SAT-FFF case, synchronization between *y* and *z* occurs for the UNSAT-FFF (see S1 File Section II). However, the impact of the repressor feedback loop on the dynamical behavior of this circuit is more profound, since it leads to oscillations. Thus, while both, SAT-FFF and UNSAT-FFF, lead to synchronization of Y and Z, the former synchronizes into a fixed point and the later into an oscillatory limit cycle.

We set λ = *γ*_*x*_*γ*_*y*_/*k*_*y*_ *α*, and *β* = 1 − *α*, so that we rewrite Eq (3) for the rescaled variables *ψ*_*t*_ = *y*_*t*_/*k*_*y*_ and *ζ*_*t*_ = *z*_*t*_/*k*_*y*_ as

$$\begin{array}{c} \begin{array}{cc} \psi_{t+1} & =\beta \psi_{t}+\alpha \lambda \theta \left(x_{t}-k_{x}\right)\theta \left(k_{y}-\psi_{t}\right),\\ \zeta_{t+1} & =\beta \zeta_{t}+\alpha \lambda \theta \left(x_{t}-k_{x}\right)\theta \left(k_{y}-\psi_{t}\right). \end{array} \end{array}$$ (4)

Now, we set *x*_*t*_ = *x* constant in time for simplicity. For *x* < *k*_*x*_, the solutions exponentially decay as *ψ*_*t*_ = *ψ*_0_*e*^−*t*/*τ*^ and *ζ*_*t*_ = *ζ*_0_*e*^−*t*/*τ*^, where *ψ*_0_ is the initial condition. For *x* > *k*_*x*_, Eq (4) defines an iterative map which satisfies the following recursive equation:

$$\begin{array}{c} f^{t}\left(\psi \right)=f^{t-1}\left(\beta \psi \right)\theta \left(\psi -1\right)+f^{t-1}\left(\beta \psi +\alpha \lambda \right)\theta \left(1-\psi \right). \end{array}$$ (5)

This iterative map results in different solutions depending on the value of λ.

We consider first the case where the initial condition is *ψ*_0_ > 1. Thus, the solution of Eq (4) is *ψ*_*t*_ = *ψ*_0_*e*^−*t*/*τ*^, where *τ*^−1^ = −log(1 − *α*). This solution is correct as long as *ψ*_*t*_ > 1, but ceases to be valid at a certain time t* such that *ψ*_*t*_ < 1, which is given by t* = ⌈*τ* log *ψ*_0_⌉.

Next, we consider the case *ψ*_0_ < 1. In this case the solution is given by *ψ*_*t*_ = *ψ*_0_*e*^−*t*/*τ*^ + λ(1 − *e*^−*t*/*τ*^), which is always valid for λ < 1. Thus, when λ < 1 the system does not oscillate but it converges monotonically to a fixed point *ψ*_∞_ = λ. However when λ > 1, this solution ceases to be valid at the time $t^{*}=\left\lceil \tau \;\text{log}\frac{\lambda -\psi_{0}}{\lambda -1}\right\rceil$ such that *ψ*_*t*_ > 1. Therefore, the solution *ψ*_*t*_ oscillates in time for λ > 1. For the case of *ψ*_0_ > 1, the explicit solution is given by the general analytical expression which is plotted in Fig 2G, right:

$$\begin{array}{c} \begin{array}{ccc} \psi_{t} & =\psi_{0}e^{-t/\tau }\;\;\; & \text{for}\;t\in \{0,1,\ldots ,t_{1}=\lceil \tau \;\text{log}\;\psi_{0}\rceil \},\\ \psi_{t} & =\psi_{1}e^{-(t-t_{1})/\tau }+\lambda (1-e^{-(t-t_{1})/\tau })\;\;\; & \text{for}\;t\in \{t_{1},\ldots ,t_{2}=t_{1}+\lceil \tau \;\text{log}\frac{\lambda -\psi_{1}}{\lambda -1}\rceil \},\\ \psi_{t} & =\psi_{2}e^{-(t-t_{2})/\tau }\;\;\; & \text{for}\;t\in \{t_{2},\ldots ,t_{3}=t_{2}+\left\lceil \tau \;\text{log}\;\psi_{2}\right\rceil \}. \end{array} \end{array}$$ (6)

The general solution with initial condition *ψ*(*t*_0_) < 1 can be written in a similar way.

Thus, the main condition for oscillations in the circuits is λ > 1, and if λ < 1, there is no oscillatory behavior, and the solution *ψ*_*t*_ converges monotonically to λ. Therefore, the oscillatory phase is separated from the non-oscillatory phase by the condition:

$$\begin{array}{c} \frac{\gamma_{y}}{k_{y}}={\left(\frac{\gamma_{x}}{\alpha }\right)}^{-1}, \end{array}$$ (7)

which is depicted in the phase diagram in Fig 2G, left.

Thus, the repressor autoregulation at gene Y converts the circuit into a synchronized clock, a primary building block in any logic computational device. S1 File Section V and S2 File show all FFFs found across gene regulatory networks of the studied species spanning *A. thaliana*, *M. tuberculosis*, *B. subtilis*, *E. coli*, *salmonella*, *yeast*, mouse and humans. We also show in Table 1 the count of circuits across species and their associated Z-scores showing that these circuits are statistically significant. The algorithm to find these *fibers* in biological networks is explained in [24] and S1 File Section IV and it is available at https://github.com/makselab/FiberCodes.

**Table 1:** Symmetric circuits (*fibers*) count [24].

Species	Database	Nodes	Edges	AR Fiber			FFF			Fibonacci Fiber			n = 2 Fiber		
				*N*_real_	*N*_rand_ ± *SD*	Z-score	*N*_real_	*N*_rand_ ± *SD*	Z-score	*N*_real_	*N*_rand_ ± *SD*	Z-score	*N*_real_	*N*_rand_ ± *SD*	Z-score
Arabidopsis Thaliana	ATRM	790	1431	2	0.2 ± 0.5	4	2	0 ± 0	Inf	5	0.3 ± 0.6	8.1	0	N/A	N/A
Micobacterium tuberculosis	Research article	1624	3212	11	0.7 ± 0.8	13.2	6	0.2 ± 0.4	14.6	4	1.7 ± 1.4	1.7	0	N/A	N/A
Bacillus subtilis	SubtiWiki	1717	2609	35	0.3 ± 0.5	64.6	13	0.3 ± 0.5	23.4	1	1.3 ± 1.2	-0.2	2	0 ± 0	63.2
Escherichia coli	RegulonDB	879	1835	14	0.2 ± 0.5	29.1	12	0.1 ± 0.2	49.4	2	0.5 ± 0.8	1.9	1	0 ± 0	> 3
Salmonella SL1344	SalmoNet	1622	2852	21	0.7 ± 0.8	25	14	0.2 ± 0.4	32	2	1.4 ± 1.3	0.5	3	0 ± 0	> 3
Yeast				10			5			3			0		
	YTRP_regulatory	3192	10947	10	0.3 ± 0.6	17.3	4	0.2 ± 0.4	8.5	2	1.8 ± 1.3	0.2	0	N/A	N/A
	YTRP_binding	5123	38085	2	0.1 ± 0.3	6.3	0	N/A	N/A	0	N/A	N/A	0	N/A	N/A
Mouse	TRRUST	2456	7057	1	0.1 ± 0.4	2.3	0	N/A	N/A	6	0.3 ± 0.6	9.3	0	N/A	N/A
Human				1			1			100			1		
	TRRUST	2718	8215	0	N/A	N/A	0	N/A	N/A	10	0.4 ± 0.6	16.3	0	N/A	N/A
	TRRUST_2	2862	9396	0	N/A	N/A	0	N/A	N/A	11	0.4 ± 0.7	16	0	N/A	N/A
	KEGG	5164	59680	1	0.06 ± 0.25	3.76	1	0 ± 0	> 3	79	0.6 ± 0.7	112	1	0 ± 0	> 3

We report the Z-scores showing that all found fibers are statistically significant. We use a random null model with the same degree sequence (and sign of interaction) as the original network to calculate the random count *N*_rand_ and compare with the real circuit count *N*_real_ to get the Z-score.

### A broad class of logic and dynamic repertoires in the class of fibers

**Fig 3:**
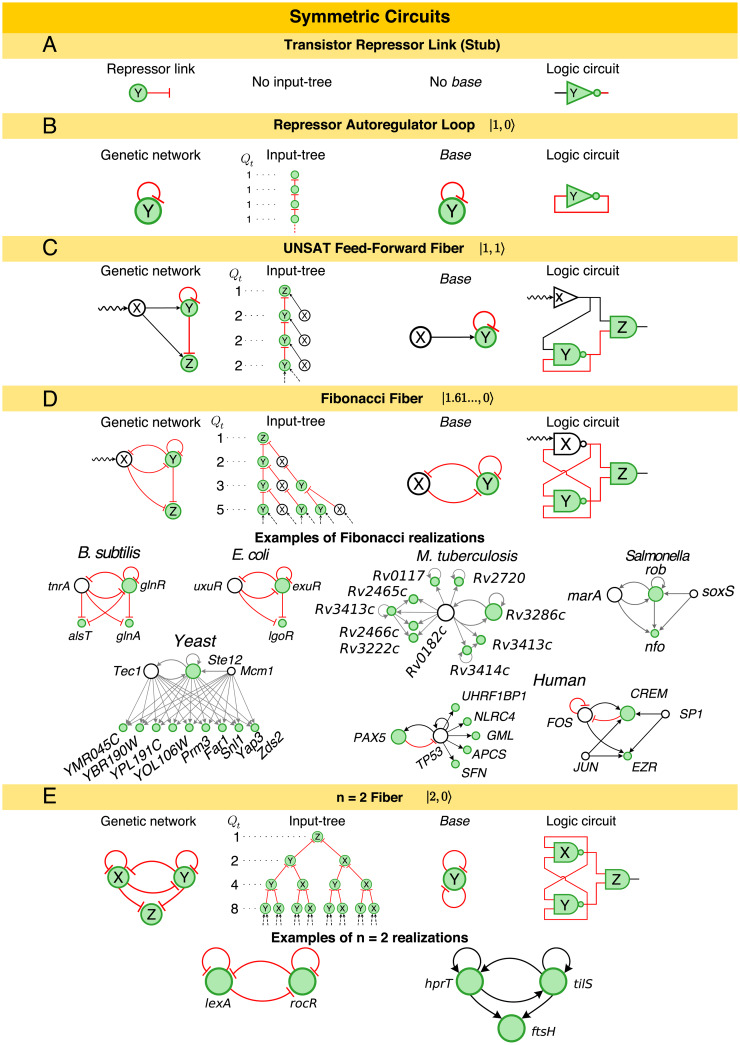
Symmetric circuits [24] function as clocks. The addition of autoregulation loops and feedback loops results on a hierarchy of circuits of increasing complexity. For example, turning the A: repressor link into a B: repressor autoregulation results on an input tree that feeds its own expression levels with *Q*_*t*_ = 1 and branching ratio *n* = 1 and it is equivalent to its own *base*. C: The addition of an external regulator (*ℓ* = 1) creates the UNSAT-FFF where genes Y and Z synchronize and oscillate. This can be verified in its corresponding input tree and logic circuit. D: The addition of a second feedback loop results in an input tree that follows the Fibonacci sequence *Q*_*t*_ = 1, 2, 3, 5, 8, …. Here, gene X is not part of the *base*. The branching structure of the input tree implies that the Fibonacci Fiber can oscillate and synchronize, but is unable to store static information. Examples are shown from all studied species. E: The second autoregulation at X results in a symmetric input tree with *Q*_*t*_ = 2*Q*_*t*−1_ and branching ratio *n* = 2. This *fiber* collapses into a *base* with two autoregulators. Examples of *n* = 2 Fiber are two *fibers* from the regulatory networks of *B. subtilis*. In this figure, we present activator links (black), repressor links (red), and interactions with unknown functionality (grey).

The procedure to build more complex fibers can be systematically extended through an algebra of circuits that adds external regulators and loops to grow the *base* of symmetric circuits (Fig 3 and S1 File Section IV A). In this space, the AR is the core loop unit referred to as |*n* = 1, *ℓ* = 0〉 in the nomenclature of [24] since it has *n* = 1 loop and *ℓ* = 0 external regulator (Fig 3B), and the FFF is |*n* = 1, *ℓ* = 1〉 (Fig 3C).

From this starting point, one can grow the number of regulator genes, |1, *ℓ*〉 with *ℓ* > 1. This does not affect the complexity because all the relevant dynamics remain constrained to the sole loop in the FFF circuit. Likewise, there are a number of circuits that can correspond to |1, 1〉. However, only certain modifications conserve the topological class identified by |1, 1〉. For instance, changing the sign of the edges is allowed as long as the edges of each gene are the same, but removing the edge X→Z will break the symmetry of the fiber, so it is not allowed. Adding a second node downstream of Y will conserve the topological class but only if it interacts with X. This situation changes as soon as the *fiber* feeds information back to the external world. This is the case of the circuits in Fig 3D, where the gene Y now regulates its own regulator gene X. The inclusion of this second feedback loop results in the coexistence of two time-scales in the network. This, in turn, increases the diversity of trajectories and delays in the network; a dynamic complexity that is measured by the divergence of the input tree, which is captured by the sequence *Q*_*t*_ representing the number of source genes with paths of length *t* − 1 to the target gene, see Fig 3 and [24]. Simply put, the input tree is a rooted tree with a gene at the root (*Q*_1_ = 1), and *Q*_*t*_ represents the number of genes in the *t*−th layer of the input tree. Then, the divergence of the input tree is captured by its branching ratio measured by $n={\left(\frac{Q_{t+1}}{Q_{t}}\right)}_{t\to \infty }$, see [24] for details.

A quantitative analysis of this measure yields exactly the golden ratio ${\left(\frac{Q_{t+1}}{Q_{t}}\right)}_{t\to \infty }=\varphi =(1+\sqrt{5})/2=1.6180\ldots$ for the circuits in Fig 3D, revealing that the input tree is a Fibonacci sequence *Q*_*t*_ = *Q*_*t*−1_ + *Q*_*t*−2_ updating the current state two steps backwards, see [24]. We have called this class of circuits, Fibonacci Fibers, in [24]. For example, the repressor interactions between genes X = *uxuR*, Y = *exuR*, and Z = *lgoR* from the *E. coli* network function exactly as a Fibonacci Fiber (Fig 3D). Other typical examples of Fibonacci Fibers in the transcriptional networks across species are also shown in Fig 3D. S1 File Section V and S2 File show all found *fibers*, and Table 1 the counts for all *fibers* and their associated Z-scores showing that these circuits are statistically significant. The important component of these circuits is the delay in the feedback loop through the regulator from Y → X and back to Y captured by the *Q*_*t*−2_ term in the Fibonacci sequence. This circuit has been synthetically implemented by Stricker *et al*. [22] using a hybrid promoter that drives the transcription of genes *araC* and *lacI* forming a dual-feedback circuit. The functionality of this circuit has been demonstrated to be robust oscillations due to the negative feedback loop [22]. We will show next that a symmetry breaking in this Fibonacci circuit forms the *base* of the JK flip-flop, which is the universal storage device of computer memories [33].

The complexity of the Fibonacci Fiber with feedback to the regulator is 1.6180…, which is lower than the number of loops in the circuit (two). The intuition of what this reveals is that, in this circuit the regulator X is still not part of the *base* (see Fig 3D), since it does not receive input from itself and hence it is not within the symmetry of the *fiber*. This, in turn, indicates naturally that the next element in the hierarchy of *fibers* results from the inclusion of an AR loop in X. This creates a fully symmetric circuit (Fig 3E) with a core |2, *ℓ* = 0〉 that feeds the reporter/enzyme Z. In this case, the complexity of the circuit is exactly two (the number of autoregulators within the *fiber*). Examples are shown in Fig 3E and Supplementary Materials.

### Broken symmetry circuits as memory storage devices

All symmetric circuits shown in Fig 3 [24] work as clocks with varying levels of sophistication and robustness given by their time-scales or loops (see S1 File Sections III C and IV A). Additionally, the more complex Fibonacci Fibers store memory dynamically by integrating sequences of its two immediate past states, according to the input tree, which computes temporal convolutions. However, this is only a short-term memory, stored dynamically and continually erased. This raises the necessity of understanding how these canonical biological circuits can perform controlled memory set and reset, a fundamental component of all computing devices [33].

We show next that static memory storage requires breaking the fibration symmetry of each circuit creating structures analogous to ‘flip-flops’ [33] in electronics that use a bistable toggle switch [14, 15] to store a bit of binary information into computer memory. As we show below, in genetic networks, symmetry breaking endows the circuits with the ability to remember.

**Fig 4:**
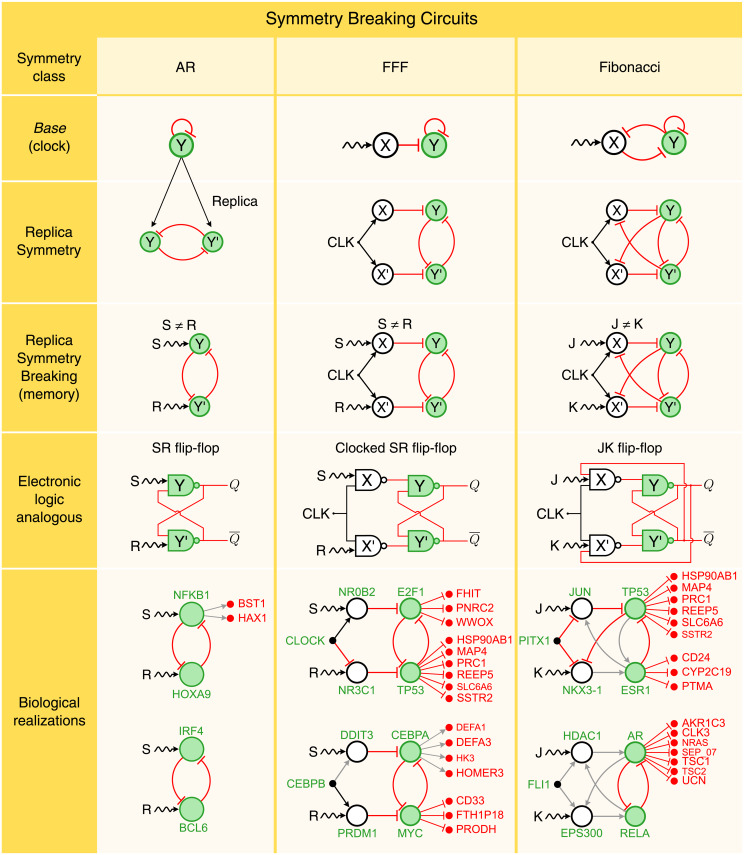
Broken symmetry circuits function as memory. **AR (first column)**: The replica symmetry duplication of the AR symmetric circuit results in a network that is analogous to the SR flip-flop circuit. The symmetry between Y and Y’ is broken by the inputs S and R, such that S ≠ R, resulting into a pair logical outputs *Q* and $\bar{Q}=\text{not}(Q)$. **FFF (second column)**: Following the same strategy, we replicate the FFF through a replica symmetry duplication. Note that this operation adds a second level of logic gates to the SR flip-flop. In order to have consistent logic operations, we add an input clock gene CLK in addition to S and R. The resulting circuit is analogous to the Clocked SR flip-flop logic circuit. **Fibonacci (third column**: The replica symmetry duplication of the Fibonacci Fiber results in a logic circuit which is analogous to the JK flip-flop. For each symmetry breaking class, we show two examples of circuits from the human regulatory network. The external regulator genes, S and R (J and K), provide inputs which are logically processed by the circuit, accordingly to the type of interaction links between the genes, activators (black arrows) or repressors (red flat links). The outputs of the circuits (green genes) regulate the expression levels of other genes (in red) without affecting the circuit functionality. Here, grey arrows correspond to interactions with unknown functionality.

Next, we extend the constructive process described above to include symmetry breaking. We do so by mimicking an evolutionary process where circuits ‘grow’ by a ‘duplication’ event (analogous to gene duplication in evolution) that conserves the *base* of the original circuit (Fig 4, first and second row). Then, breaking the symmetry creates a new functionality. We begin this with the simplest case of AR gene Y: |*n* = 1, *ℓ* = 0〉. This gene is duplicated by ‘opening up’ the AR loop into two mutually repressed replica genes, Y and Y’ (Fig 4, second row). This creates a bistable toggle switch as studied in synthetic genetic circuits [14, 15] that is topologically equivalent to the core AR loop |1, 0〉 since both have the same input tree and *base* (Fig 4, first row).

The symmetry of the replica circuit is then explicitly broken by including different inputs, S and R, to regulate genes Y and Y’, respectively (Fig 4, third row). S and R represent either genes or effectors that break the symmetry of the circuit. Symmetry is now broken, and the genetic circuit becomes analogous to a Set-Reset (SR) flip-flop [33], the simplest canonical circuit to store a bit of binary information in electronic computer memory (see Fig 4, fourth row).

The symmetry is explicitly broken by applying set-reset inputs S ≠ R. Specifically, when S = 1 and R = 0, the circuit stores a bit of information. But here is the interesting fact: this state is kept in memory even when S = R = 1. That is, the circuit remains in the broken symmetry state even if the inputs are now equal and symmetric. In other words, the symmetry is now ‘spontaneously’ broken [34], since a specific state is selected even without external bias, thus allowing the circuit to remember its state.

Thus, this genetic network is a toggle switch as studied in synthetic genetic circuits [14, 15] analogous to the SR flip-flop logic circuit shown in Fig 4, fourth row. When the controlling inputs are *S* = 0 and *R* = 1, the SR flip-flop stores one bit of information resulting in *Q* = 1 and $\bar{Q}=0$ ($\bar{Q}=\text{not}\;Q$, and the output *Q* is the logic conversion of **not** *Y*). The SR flip-flop retains this logical state even when the controlling inputs change. In other words, when *S* = 1 and *R* = 1, the feedback (the repressor interactions between Y and Y’) maintains the outputs *Q* and $\bar{Q}$ to its previous state. This state changes only when we reset the circuits with *S* = 1 and *R* = 0. In this last case, the SR flip-flop stores *Q* = 0 and $\bar{Q}=1$, which is also remembered when both inputs are high (*S* = 1 and *R* = 1, S1 File Section IV B).

This spontaneous symmetry breaking [34] has analogy with a ferromagnetic material. When the temperature is high enough, the ferromagnet does not show any magnetic property. Moreover, even lowering the temperature, in absence of any polarizing field, the material does not magnetize. On the contrary, if an external magnetic field is applied to the ferromagnet, like a magnet put in contact to a needle for enough time, and then removed, then the needle becomes magnetic itself, in that it remembers the direction of the previously applied magnetic field, thus breaking, spontaneously, the rotational symmetry. Biological realizations of this replica symmetry breaking process are shown in Fig 4, last row. Full list of symmetry broken circuits in gene regulatory networks across species appears in S1 File Section V and S2 File. Table 2 shows the Z-scores of these circuits indicating their significance. The algorithm to identify these broken symmetry flip-flops in biological networks is developed in S1 File Section VII and is available at https://github.com/makselab/CircuitFinder.

**Table 2:** Broken symmetry circuits count.

Species	Database	Nodes	Edges	SR flip-flop			Clocked SR flip-flop			JK flip-flop		
				*N*_real_	*N*_rand_ ± *SD*	Z-score	*N*_real_	*N*_rand_ ± *SD*	Z-score	*N*_real_	*N*_rand_ ± *SD*	Z-score
Arabidopsis Thaliana	ATRM	790	1431	47	1.6 ± 1.2	36.40	3	0.2 ± 0.5	5.80	2	0 ± 0	> 3
Micobacterium tuberculosis	Research article	1624	3212	6	1.7 ± 1.4	3.20	0	N/A	N/A	0	N/A	N/A
Bacillus subtilis	SubtiWiki	1717	2609	3	2.1 ± 1.4	0.6	0	N/A	N/A	0	N/A	N/A
Escherichia coli	RegulonDB	879	1835	14	2.1 ± 1.4	8.40	3	0.3 ± 0.8	3.30	0	N/A	N/A
Salmonella SL1344	SalmoNet	1622	2852	6	1.4 ± 1.2	3.80	0	N/A	N/A	0	N/A	N/A
Yeast				27			58			1		
	YTRP_regulatory	3192	10947	9	5 ± 2.5	1.60	3	3 ± 3.6	0	0	N/A	N/A
	YTRP_binding	5123	38085	31	21.6 ± 5.8	1.60	192	103.3 ± 45.6	1.90	2	6.8 ± 6.1	-0.8
Mouse	TRRUST	2456	7057	82	4 ± 2.1	37.70	216	1.9 ± 2.7	79.50	25	0.004 ± 0.06	417
Human				192			566			90		
	TRRUST	2718	8215	89	4.3 ± 2.1	40.50	247	3.5 ± 4.8	50.60	45	0.02 ± 0.2	225
	TRRUST_2	2862	9396	103	5 ± 2.3	43	319	5.9 ± 7.2	43.20	45	0.02 ± 0.3	150

We report the corresponding Z-score statistics as computed in Table 1.

Extending this duplication and symmetry breaking process to the FFF (Fig 4, second column), one can replicate the X and Y genes from the FFF *base* to create a circuit isomorphic to the Clocked SR flip-flop, another computer memory building block [33] (see S1 File Section IV B). The symmetry is broken by regulators or inducers of genes X and X’ acting as S (set) and R (reset) of memory, and it is restored when S = R, leaving the system ‘magnetized’. The hierarchy continues by replicating the Fibonacci Fiber and consequent breaking of symmetry when the inputs J and K are different (Fig 4 third column, see S1 File Section IV B). This structure is isomorphic (i.e., has the same *base*) to the JK flip-flop in electronics, which is the most widely used of all flip-flop designs [33]. In its symmetric state, the JK flip-flop is isomorphic to the symmetric Fibonacci *base*. In the symmetry broken phase, it acts as a memory device which presents two possibilities. A ‘chiral’ symmetry (where Y feeds X and Y’ feeds X’) and a ‘parity’ symmetry (left-right reflection, where Y feeds X’ and Y’ feeds X). This last one is the one realized in biological circuits, see Fig 4, third column. Examples of JK flip-flops are abundant in gene regulatory networks in human and mouse. They are shown in Fig 4, last row and full list in S1 File Section V, S2 File, and Z-scores in Table 2.

## Discussion

In summary, fibration symmetries and broken symmetries in gene regulatory networks reveal the functions of synchronization, clocks and memory through electronic analogues of transistors, ring oscillators, current-mirror circuits, and flip-flops. They result in a hierarchy of building blocks with progressively more complex dynamics obtained by iterating a procedure of replication and symmetry breaking. Beyond the circuits discussed here, the biological hierarchy can be extended to any number *m* of loops of length *d* and autoregulators in the *fiber* *n*, to form ever more sophisticated circuits whose complexity is expressed in generalized Fibonacci sequences *Q*_*t*_ = *nQ*_*t*−1_ + *mQ*_*t*−*d*_.

Gene regulatory structures are a mixture of combinational logic circuits, like FFF, and sequential logic circuits, like FF. They provide the network with a structure analogous to a programmable logic device or chip where the ‘register’ is a set of flip-flop circuits tied up together acting as the memory clock of the genetic network that feeds the combinatorial logic circuits made of simpler feed-forward circuit of low symmetry. Complex biological circuitry can then be seen as an emergent process guided by the laws of symmetry that determine biological functions analogous to electronic components. The discovery of these building blocks and building rules of logic computation will allow to: (1) systematically design synthetic genetic circuits following biological symmetry, and (2) systematically map the structure and function of all biological networks, from the symmetries of the connectome [35] to genetic [24], protein and metabolic networks, following a first principle theoretical approach.

## Supporting information

S1 File Supplementary materials. Detailed description of all analytical solutions mentioned in the main text, of the data acquisition and treatment, and detailed description of the proposed algorithm to find fibers.
(PDF)

S2 File List of symmetry and broken symmetry circuits. In S2 File we present a list of circuits found in different species.
(PDF)
