# Supplementary material for: Circuits with broken fibration symmetries perform core logic computations in biological networks
Source: PLoS Comput Biol. 2020 Jun 17;16(6):e1007776. doi: 10.1371/journal.pcbi.1007776 (PMC7299331; doi:10.1371/journal.pcbi.1007776)
Supplement: S1 File — Detailed description of all analytical solutions mentioned in the main text, of the data acquisition and treatment, and detailed description of the proposed algorithm to find fibers. (PDF) [file pcbi.1007776.s001.pdf]

Supplementary Materials:

Circuits with broken fibration symmetries perform core logic computations in biological networks

Ian Leifer, Flaviano Morone, Saulo D. S. Reis, José S. Andrade Jr., Mariano Sigman, and Hernán A. Makse

CONTENTS

I. Feed-forward loop: FFL 3

A. FFL discrete time model 3

B. FFL ODE 4

C. FFL with OR gate 6

II. Satisfied Feed-Forward Fiber: SAT-FFF 7

A. SAT-FFF discrete time model 7

B. SAT-FFF ODE 9

III. Unsatisfied Feed-Forward Fiber: UNSAT-FFF 11

A. UNSAT-FFF ODE 11

B. Period-amplitude relationship 15

C. UNSAT-FFF clock functionality 16

IV. Examples of symmetry and broken symmetry circuits 17

A. Symmetry circuits (*fibers*) from [24] 17

1. Repressor regulator link (stub) 17

2. Repressor AR loop: $|1, 0\rangle$ 17

3. UNSAT-FFF: $|1, 1\rangle$ 18

4. Fibonacci Fiber: $|1.6180..., 0\rangle$ 18

5. $n = 2$ Fiber: $|2, 0\rangle$ 18

B. Symmetry breaking circuits 19

1. AR symmetry breaking circuit: SR flip-flop 19

2. FFF symmetry breaking circuit: Clocked SR flip-flop 20

3. Fibonacci symmetry breaking circuit: JK flip-flop 20

V. Description of datasets 21

VI. Algorithm to find fibers 21

VII. Algorithm to find broken symmetry circuits 24

References 27

I. FEED-FORWARD LOOP: FFL

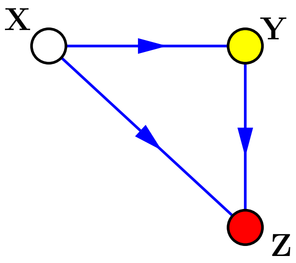

In what follows, we analyze in detail the FFL. First, we present the analytical solution for the discrete time model of the FFL, where we show that the FFL does not synchronize nor oscillates. After that, we reach the same conclusion by using a continuous variable approach. Finally, we show the discrete time solution with the OR gate. Solutions for the FFL have been considered in the literature. Here we adapt those results to the particular models used in our studies to perform consistent comparisons with the solutions of the fiber dynamics obtained through the paper.

A. FFL discrete time model

Starting from Eq. (1) in the main text, we define rescaled variables $\psi_t = y_t/k_y$ and $\zeta_t = z_t/k_y$, we rewrite Eq. (1) as

$$\begin{aligned} \psi_{t+1} &= \beta\psi_t + \alpha\eta\theta(x_t - k_x), \\ \zeta_{t+1} &= \beta\zeta_t + \alpha\lambda\theta(x_t - k_x)\theta(\psi_t - 1), \end{aligned} \tag{1}$$

where we set $\beta = (1-\alpha)$, $\eta = \gamma_x/\alpha k_y$, and $\lambda = \gamma_x\gamma_y/\alpha k_y$. Equation (1) defines an iterative map $\psi_{t+1} = f(\psi_t)$ which provides a solution

$$\psi_t = f(\psi_{t-1}) = f^2(\psi_{t-2}) = \ldots = f^t(\psi_0). \tag{2}$$

A closed form for ψ_t depends on the value of x. For the sake of simplicity, consider $x_t = x$ constant in time. If $x < k_x$, the solution is simple, and always decays as $\psi_t = \psi_0 e^{-t/\tau}$, where we choose to write $\beta^t = e^{-t/\tau}$ such as $\tau^{-1} = -\log(1-\alpha)$. On the other hand, if $x > k_x$, the iterative map is $f(\psi_t) = \beta\psi_t + \eta$, leading to a solution that converges to η as $\psi_t = \psi_0 e^{-t/\tau} + \eta\left(1 - e^{-t/\tau}\right)$. Therefore, the solution to ψ_t is given by:

$$\psi_t = \psi_0 e^{-t/\tau} + \eta(1 - e^{-t/\tau})\theta(x_t - k_x). \tag{3}$$

Similarly, the solution for ζ_t depends on x, but it also depends on ψ_0 and η. For $x < k_x$, it always decays to zero as $\zeta_t = \zeta_0 e^{-t/\tau}$. When $x > k_x$, the variable ζ_t follows different behaviors.

For $\psi_0 < 1$, we also find a solution that decays as $\zeta_t = \zeta_0 e^{-t/\tau}$. However, if $\eta > 1$, this solution ceases to be valid at a given time t^* such that $\psi_{t^*} > 1$, which is given by $t^* = \lceil \tau \log((\eta - \psi_0)/(\eta - 1)) \rceil$. Here, $\lceil x \rceil$ denotes the smallest integer larger than x, e.g., $\lceil 1.5 \rceil = 2$. For $t > t^*$, as ψ_t saturates to η, the rescaled variable ζ_t converges to λ as $\zeta_t = \zeta_0 e^{-(t-t^*)/\tau} + \lambda\left(1 - e^{-(t-t^*)/\tau}\right)$.

Next, we consider the case $\psi_0 > 1$. In this case, the solution for ζ_t is given by $\zeta_t = \zeta_0 e^{-t/\tau} + \lambda(1 - e^{-t/\tau})$, for $\eta > 1$. In contrast, when $\eta < 1$, this solution is valid only up to $t^* = \lceil \tau \log((\psi_0 - \eta)/(1 - \eta)) \rceil$, such that $\psi_t < 1$. As ψ_t saturates to η, the rescaled variable ζ_t exponentially decays as $\zeta_t = \zeta_0 e^{-(t-t^*)/\tau}$.

To summarize, the possible solutions for ζ_t depending on ψ_0 and η are:

$$
\begin{aligned}
\psi_0 > 1, \eta > 1 \quad &\rightarrow \quad \zeta_t = \zeta_0 e^{-t/\tau} + \lambda(1 - e^{-t/\tau}), \\
\psi_0 > 1, \eta < 1 \quad &\rightarrow \quad \zeta_t = \zeta_0 e^{-t/\tau} + \lambda(1 - e^{-t/\tau}) \quad \text{for } t \in \left\{0, 1, \ldots, t_1 = \left\lceil \tau \log \frac{\psi_0 - \eta}{1 - \eta} \right\rceil \right\}, \\
&\qquad\; \zeta_t = \zeta_1 e^{-(t-t_1)/\tau} \quad \text{for } t > t_1, \\
\psi_0 < 1, \eta > 1 \quad &\rightarrow \quad \zeta_t = \zeta_0 e^{-t/\tau} \quad \text{for } t \in \left\{0, 1, \ldots, t_1 = \left\lceil \tau \log \frac{\eta - \psi_0}{\eta - 1} \right\rceil \right\}, \\
&\qquad\; \zeta_t = \zeta_1 e^{-(t-t_1)/\tau} + \lambda\left(1 - e^{-(t-t_1)/\tau}\right) \quad \text{for } t > t_1, \\
\psi_0 < 1, \eta < 1 \quad &\rightarrow \quad \zeta_t = \zeta_0 e^{-t/\tau}.
\end{aligned}
\tag{4}
$$

Here, $\zeta_1 = \zeta_{t=t_1}$.

From this discussion, we find that the rescaled variables ψ_t and ζ_t do not synchronize. That is, they do not reach the same value at their fixed points: $\psi_t \neq \zeta_t$ when $t \to \infty$, unless we use a specific set of parameters. Moreover, ψ_t and ζ_t also do not reach oscillatory states. The same conclusion is extended to the original variables y_t and z_t.

B. FFL ODE

In order to show that our results presented in the main text are consistent with a continuous variable approach, now we focus our attention on the modelling of the FFL [4, 20, 21] by using ordinary differential equations (ODE). First, we write the ODE governing the

dynamics of expression levels $y(t)$ and $z(t)$:

$$\begin{aligned} \dot{y}(t) &= -\alpha y(t) + \gamma_x \theta(x(t) - k_x), \\ \dot{z}(t) &= -\alpha z(t) + \gamma_x \theta(x(t) - k_x) \times \gamma_y \theta(y(t) - k_y). \end{aligned} \tag{5}$$

For the sake of simplicity, we consider $x(t) = x$ constant in time.

By using the rescaled functions $\psi(t) = y(t)/k_y$ and $\zeta(t) = k_y$, we rewrite Eq. (5) as the following set of ODEs:

$$\begin{aligned} \dot{\psi}(t) + \alpha\psi(t) &= \alpha\eta\theta(x_t - k_x), \\ \dot{\zeta}(t) + \alpha\zeta(t) &= \alpha\lambda\theta(x_t - k_x)\theta(\psi_t - 1), \end{aligned} \tag{6}$$

where $\eta = \gamma_x/\alpha k_y$ and $\lambda = \gamma_x\gamma_y/\alpha k_y$.

For the case of $x < k_x$, Eqs. (6) become a set of homogeneous ODEs with solutions that decay exponentially:

$$\begin{aligned} \psi(t)_{x<k_x} &= \psi_0 e^{-\alpha t}, \\ \zeta(t)_{x<k_x} &= \zeta_0 e^{-\alpha t}. \end{aligned} \tag{7}$$

Where, ψ_0 and ζ_0 are the initial conditions for the rescaled functions.

When $x > k_x$, we find

$$\psi(t)_{x>k_x} = \psi_0 e^{-\alpha t} + \eta\left(1 - e^{-\alpha t}\right). \tag{8}$$

Therefore, the solution for $\psi(t)$ converges to η as $t \to \infty$, similar to the solution of the discrete time equation presented in the main text.

On the other hand, the solution for $\zeta(t)$ also depends the values of ψ_0 and η. By carrying on a calculation similar to the one presented on the main text for the discrete time case, one finds:

$$\begin{aligned}
\psi_0 > 1, \eta > 1 \quad &\rightarrow \quad \zeta(t)_{x>k_x} = \zeta_0 e^{-\alpha t} + \lambda(1 - e^{-\alpha t}), \\
\psi_0 > 1, \eta < 1 \quad &\rightarrow \quad \zeta(t)_{x>k_x} = \zeta_0 e^{-\alpha t} + \lambda(1 - e^{-\alpha t}) \quad \text{for } t \in \left\{0, 1, \ldots, t_1 = \frac{1}{\alpha}\log\left(\frac{\psi_0 - \eta}{1 - \eta}\right)\right\}, \\
&\qquad\quad \zeta(t)_{x>k_x} = \zeta_1 e^{-\alpha(t - t_1)} \quad \text{for } t > t_1, \\
\psi_0 < 1, \eta > 1 \quad &\rightarrow \quad \zeta(t)_{x>k_x} = \zeta_0 e^{-\alpha t} \quad \text{for } t \in \left\{0, 1, \ldots, t_1 = \frac{1}{\alpha}\log\left(\frac{\eta - \psi_0}{\eta - 1}\right)\right\}, \\
&\qquad\quad \zeta(t)_{x>k_x} = \zeta_1 e^{-\alpha(t - t_1)} + \lambda\left(1 - e^{-\alpha(t - t_1)}\right) \quad \text{for } t > t_1, \\
\psi_0 < 1, \eta < 1 \quad &\rightarrow \quad \zeta(t)_{x>k_x} = \zeta_0 e^{-\alpha t}.
\end{aligned} \tag{9}$$

Here, $\zeta_1 = \zeta(t)|_{t=t_1}$.

Clearly, from the above solutions, the expression levels from genes Y and X do not synchronize, since $y(t) \neq z(t)$ as $t \to \infty$. In addition, $y(t)$ and $z(t)$ also do not reach oscillatory states, in accordance with the results of the discrete time model.

C. FFL with OR gate

We present the solution for the FFL [4, 20, 21] with an OR gate. We consider the coherent version cFFL where all regulations are activators. The discrete-time dynamics of expression levels y_t and z_t of genes Y and Z in the cFFL with a Boolean OR gate are given by the following pair of difference equations:

$$\begin{aligned} y_{t+1} &= (1-\alpha)y_t + \gamma_x \theta(x_t - k_x), \\ z_{t+1} &= (1-\alpha)z_t + \gamma_x \theta(x_t - k_x) + \gamma_y \theta(y_t - k_y), \end{aligned} \tag{10}$$

where α, γ_y, and γ_z have the same definition as in the main text. By adopting the same rescaled variables, $\psi_t = y_t/k_y$ and $\zeta_t = z_t/k_y$, we rewrite Eq. (10) as:

$$\begin{aligned} \psi_{t+1} &= \beta\psi_t + \alpha\eta\theta(x_t - k_x), \\ \zeta_{t+1} &= \beta\zeta_t + \alpha\lambda_x\theta(x_t - k_x) + \alpha\lambda_x\theta(\psi_t - 1), \end{aligned} \tag{11}$$

where we have $\beta = (1-\alpha)$, $\eta = \gamma_x/\alpha k_y$, $\lambda_x = \gamma_x/\alpha k_y$, and $\lambda_y = \gamma_y/\alpha k_y$. Again, without lack of generality, we consider $x_t = x$ constant in time. Clearly, the solution for ψ_t is not affected by the OR gate, therefore, it depends only on the value of x and is given by:

$$\psi_t = \psi_0 e^{-t/\tau} + \eta(1 - e^{-t/\tau})\theta(x_t - k_x). \tag{12}$$

Again, $\tau = -\log(1-\alpha)$. Thus, if $x < k_x$, ψ_t exponentially decays to zero. On the contrary, if $x > k_x$, ψ_t converges to η. The solution for ζ_t displays different solutions, depending on the combination of x and the initial value ψ_0.

First, consider the case of $x < k_x$. For $\psi_0 < 1$, we find a solution that always decays as $\zeta_t = \zeta_0 e^{-t/\tau}$. When $\psi_0 > 1$, the solution for ζ_t is given by $\zeta_t = \zeta_0 e^{-t/\tau} + \lambda_y \left(1 - e^{-t/\tau}\right)$. However, since $x < k_x$ and ψ_t exponentially decays, this solution is valid only until $t^* = \lceil \tau \log \psi_0 \rceil$, the instant that $\psi_{t^*} < 1$. As ψ_t decays, ζ_t also decays as $\zeta_t = \zeta_{t^*} e^{-(t-t^*)/\tau}$.

We consider next the case of $x > k_x$. In this case, ψ_t always converges to η. Therefore, the solution for ζ_t depends not only on ψ_0, but also on η. For $\psi_0 < 1$ and $\eta < 1$, we find

that ζ_t converges to λ_x as $\zeta_t = \zeta_0 e^{-t/\tau} + \lambda_x \left(1 - e^{-t/\tau}\right)$. However, if $\eta > 1$, this solution is not valid after $t^* = \lceil \tau \log\left(\psi_0 - \eta\right) / \left(1 - \eta\right) \rceil$, when $\psi_t > 1$. In this case, as ψ_t saturates to η, ζ_t converges to $\lambda_x + \lambda_y$ as $\zeta_t = \zeta_{t^*} e^{-(t-t^*)/\tau} + (\lambda_x + \lambda_y)\left(1 - e^{-(t-t^*)/\tau}\right)$.

For the case of $\psi_0 > 1$ and $\eta > 1$, the rescaled variable ζ_t always converges to $\lambda_x + \lambda_y$ as $\zeta_t = \zeta_0 e^{-t/\tau} + (\lambda_x + \lambda_y)\left(1 - e^{-t/\tau}\right)$. However, if $\eta < 1$, this solution ceases to be valid at $t^* = \lceil \tau \log\left(\left(\eta - \psi_0\right) / \left(\eta - 1\right)\right) \rceil$. Therefore, for this case ζ_t converges to λ_x as $\zeta_t = \zeta_{t^*} e^{-(t-t^*)/\tau} + \lambda_x \left(1 - e^{-(t-t^*)/\tau}\right)$.

From the discussion above, it is clear that the rescaled variables ψ_t and ζ_t neither synchronize nor oscillate. Extending this results to the original variables y_t and z_t, we conclude that the expression levels of the genes Y and Z also do not synchronize: $y_t \neq z_t$ when $t \to \infty$.

II. SATISFIED FEED-FORWARD FIBER: SAT-FFF

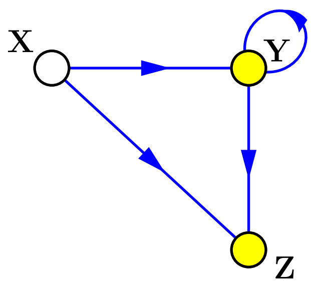

Below, we describe the solutions of the SAT FFF circuit in the discrete time continuous variable model and in the ODE model.

A. SAT-FFF discrete time model

The SAT-FFF is a Feed-Forward Fiber with activator autoregulation where all interactions are satisfied. That is, it does not present the phenomenon of frustration and the dynamics converges to a fixed point. This can be simply seen by considering gene X high, which makes also gene Y high and Z high too. Finally, the configuration satisfies the AR loop, so all bonds are satisfied. The SAT-FFF is constructed on top of the cFFL by the addition of an autoregulator loop on the Y gene, as depicted in Fig. 1E (main text) and S1 Fig. 1A. The discrete time dynamics of the SAT-FFF with a logic interaction term is given

by:

$$y_{t+1} = (1-\alpha)y_t + \gamma_x\theta(x_t - k_x) \times \gamma_y\theta(y_t - k_y),$$
$$z_{t+1} = (1-\alpha)z_t + \gamma_x\theta(x_t - k_x) \times \gamma_y\theta(y_t - k_y). \tag{13}$$

Note that the Heaviside function $\theta(y_t - k_y)$ represents the activator feedback on the autoregulation of the Y gene. We consider an AND gate for the interactions [4]. Analogous results can be obtained for OR gates or with an first-order ODE model. Writing down the set of equations for the rescaled variables $\psi_t = y_t/k_y$ and $\zeta_t = z_t/k_y$, we get:

$$\psi_{t+1} = \beta\psi_t + \alpha\lambda\theta(x_t - k_x)\theta(\psi_t - 1),$$
$$\zeta_{t+1} = \beta\zeta_t + \alpha\lambda\theta(x_t - k_x)\theta(\psi_t - 1). \tag{14}$$

Here, we made use of $\lambda = \gamma_x\gamma_y/\alpha k_y$ and $\beta = (1-\alpha)$. We note that, since the second term on the right-hand side of both equations are equal, the dynamical variables ψ_t and ζ_t must synchronize, as well as y_t and z_t. Again, considering $x_t = x$ constant, for $x < k_x$, the solutions for ψ_t and ζ_t are trivial: both variables decay exponentially as $\psi_t = \psi_0 e^{-t/\tau}$ and $\zeta_t = \zeta_0 e^{-t/\tau}$, where $\tau^{-1} = -\log(1-\alpha)$. This behavior is shown by the red solid line in S1 Fig. S1B with $\psi_0 = 0.9$.

In terms of the iterative map, the dynamics of the SAT-FFF for the rescaled variable ψ_t with $x > k_x$ is:

$$\psi_{t+1} = \beta\psi_t + \alpha\lambda\theta(\psi_t - 1) \equiv f(\psi_t), \tag{15}$$

so we find

$$f^t(\psi) = f^{t-1}(\beta\psi)\theta(1-\psi) + f^{t-1}(\beta\psi + \lambda)\theta(\psi - 1). \tag{16}$$

This iterative map $\psi_t = f(\psi_t)$ provides different solutions depending on ψ_0. Similar to the case of $x < k_x$, if $\psi_0 < 1$, the solution decays to zero as $\psi_t = \psi_0 e^{-t/\tau}$. However, if $\psi_0 > 1$, there are two possibilities, depending on the values of $\lambda = \gamma_x\gamma_y/\alpha k_y$.

First, if $\lambda > 1$, the solution for both rescaled variables converges to λ as $\psi_t = \psi_0 e^{-t/\tau} + \lambda(1-e^{-t/\tau})$ and $\zeta_t = \zeta_0 e^{-t/\tau} + \lambda(1-e^{-t/\tau})$, such that $\psi_{t\to\infty} \to \lambda$ and $\zeta_{t\to\infty} \to \lambda$, as presented by the blue dash-dotted line in S1 Fig. S1B. For this case, we use $\psi_0 = 1.1$ and $\lambda = 2$.

For $\lambda < 1$, ψ_t approaches 1 at a time t^* given by

$$t^* = \left\lceil \frac{1}{\log(1-\alpha)} \log\left(\frac{1-\lambda}{\psi_0 - \lambda}\right) \right\rceil. \tag{17}$$

Then, for $t > t^*$, the solutions decay to zero as $\psi_t = e^{-(t-t^*)/\tau}$ and $\zeta_t = \zeta_{t^*} e^{1(t-t^*)/\tau}$. This behavior is presented on S1 Fig. S1B by the dashed green line, where we use $\psi_0 = 2$ and

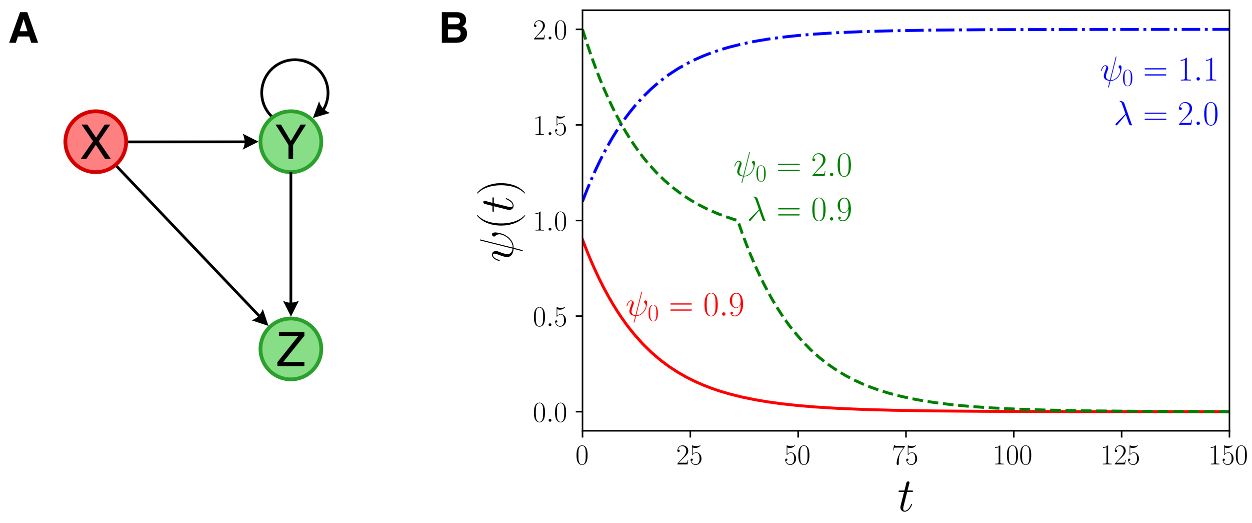

FIG. 1. **SAT-FFF. a,** Network representation of the SAT-FFF. All regulations are activators. **b,** Different behaviors for the analytical solutions of ψ_t depending on ψ_0 and λ, as discussed in the text.

$\lambda = 0.9$. The rescaled variables ψ_t and ζ_t always synchronize, so do y_t and z_t. This can be proved by finding the difference $\epsilon_t = \psi_t - \zeta_t$. For all the cases discussed above, ϵ_t decays exponentially fast as $\epsilon_t = (\psi_0 - \zeta_0)\, e^{-t/\tau}$.

Now, we can use the solution with x_t constant to qualitatively understand the SAT-FFF in general. An example of the SAT-FFF with non-constant x_t is depicted on Fig. 1F in the main text. As shown, variable y_t and z_t do synchronize but with no internal oscillations. We feed an external oscillatory pattern of x_t as a square wave. For $x_t < k_x$, both y_t an z_t decay exponentially. When $x_t > k_x$, they tend to saturate at $\gamma_x\gamma_y/\alpha$. The SAT-FFF synchronizes at a fixed point.

B. SAT-FFF ODE

Here, we consider the ODE model of SAT-FFF to confirm results presented in Section II A. The dynamics of gene X is driven by outside sources, so we only consider the dynamics of genes Y and Z which are described by equations:

$$\begin{cases} \dot{y} = -\alpha y(t) + \gamma_x \theta(x(t) - k_x) \times \gamma_y \theta(y(t) - k_y), \\ \dot{z} = -\alpha z(t) + \gamma_x \theta(x(t) - k_x) \times \gamma_y \theta(y(t) - k_y). \end{cases} \tag{18}$$

Taking $\psi(t) = y(t)/k_y$, $\zeta(t) = z(t)/k_y$ and $\delta = \gamma_x\gamma_y/k_y$ we transform Eq. (18) to:

$$\begin{cases} \dot{\psi} = -\alpha\psi(t) + \delta\ \theta(x(t) - k_x) \times \theta(\psi(t) - 1), \\ \dot{\zeta} = -\alpha\zeta(t) + \delta\ \theta(x(t) - k_x) \times \theta(\psi(t) - 1). \end{cases} \tag{19}$$

Without loss of generality, we consider the case of $x(t) = x$ constant in time. If $x < k_x$, then the solution of Eq. (19) is given by:

$$\begin{cases} \psi(t)_{x<k_x} = \psi_0 e^{-\alpha t}, \\ \zeta(t)_{x<k_x} = \zeta_0 e^{-\alpha t}, \end{cases} \tag{20}$$

where ψ_0 and ζ_0 are the initial conditions. Now, let's consider the case $x > k_x$. Equation (19) then transforms into:

$$\begin{cases} \dot{\psi} = -\alpha\psi(t) + \delta\ \theta(\psi(t) - 1) \\ \dot{\zeta} = -\alpha\zeta(t) + \delta\ \theta(\psi(t) - 1) \end{cases} \tag{21}$$

Due to the existence of isomorphic input trees between both genes, $\psi(t)$ and $\zeta(t)$ synchronize and therefore $y(t)$ and $z(t)$ synchronize also, so we only consider dynamics of the first equation:

$$\dot{\psi} = -\alpha\psi(t) + \delta\ \theta(\psi(t) - 1). \tag{22}$$

It's easy to see that for $\psi_0 < 1$, Eq. (20) will be the solution of Eq. (22). When $\psi_0 > 1$ and $\delta/\alpha > 1$, the solution is given by:

$$\psi(t) = \delta/\alpha + (\psi_0 - \delta/\alpha)e^{-\alpha t}. \tag{23}$$

In the case $\psi_0 > 1$ and $\delta/\alpha < 1$, the dynamics of ψ is split into two parts. One part before ψ decays to 1 and the other one after ψ crossed 1. The time when $\psi(t)$ crosses 1 is equal to:

$$t_c = \frac{1}{\alpha} ln(\frac{\psi_0 - \delta/\alpha}{1 - \delta/\alpha}), \tag{24}$$

and the dynamics can be written as:

$$
\begin{cases} t \in [0, t_c] & \psi(t) = \delta/\alpha + (\psi_0 - \delta/\alpha)e^{-\alpha t}, \\ t \in [t_c, \infty] & \psi(t) = \frac{\psi_0 - \delta/\alpha}{1-\delta/\alpha} e^{-\alpha t}. \end{cases} \tag{25}
$$

To summarize, the solution is:

$$
\begin{cases} \psi_0 < 1 & \psi(t) = \psi_0 e^{-\alpha t} \\ \psi_0 > 1, \frac{\delta}{\alpha} > 1 & \psi(t) = \delta/\alpha + (\psi_0 - \delta/\alpha)e^{-\alpha t} \\ \psi_0 > 1, \frac{\delta}{\alpha} < 1 & \begin{cases} t \in [0, t_c] & \psi(t) = \delta/\alpha + (\psi_0 - \delta/\alpha)e^{-\alpha t} \\ t \in [t_c, \infty] & \psi(t) = \frac{\psi_0 - \delta/\alpha}{1-\delta/\alpha} e^{-\alpha t}. \end{cases} \end{cases} \tag{26}
$$

This solution is analogous to the one obtained for the discrete time model in Section II A.

III. UNSATISFIED FEED-FORWARD FIBER: UNSAT-FFF

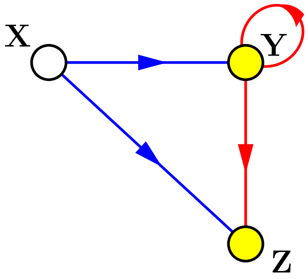

Now, we turn our attention to the UNSAT-FFF. The solution of this circuit is developed in the main text using a discrete-time difference equation with a logistic interaction. Below we elaborate on the solution of the ODE continuum model and on the conditions on the period-amplitude relation and the clock functionality.

A. UNSAT-FFF ODE

The UNSAT-FFF circuit can be reduced to study the base of the circuit since both genes Y and Z synchronize their behaviour as shown in the main text. The base of this circuit is a negative autorregulation loop (Fig. 3) plus an external regulator given by X. This circuit has been synthetically implemented by Stricker *et al.* in Ref. [18] using a promoter that drives the expression in the absence of LacI (and acts as a negative feedback loop) or in the

presence of IPTG, which acts as an activator. It was shown experimentally that this circuit leads to oscillatory behaviour in the expression profiles. This result was corroborated with a dynamical ODE model in [18] which here we adapt to study the case of the UNSAT-FFF with ODE. See also the review paper [53] for further reading.

Following the same approach as above, we consider gene $x(t) = x$ constant in time and larger than $x > k_x$, and rescale the expression of genes $y(t)$ and $z(t)$ as $\psi(t) = y(t)/k_y$ and $\zeta(t) = z(t)/k_z$. Since genes $y(t)$ and $z(t)$ synchronize their activities, then only one equation needs to be considered, $\psi(t)$.

The key to observe oscillations in a first-order ODE is to consider the delay in the signal propagation in the circuit. Without delay the dynamics converge to a fixed point; no oscillatory solution exist in a first-order ODE continuum-time model. The situation is different in the discrete-time model considered in the text. In this case, a discrete time plus a logic approximation lead to oscillations.

Negative feedback loop circuits with delays have been widely investigated in the dynamical systems literature. Here, we adapt the negative feedback loop model with delay of Stricker *et al.* (see Eq. (6) in Supplementary Information in Ref. [18]). We consider delays in the negative feedback loop which is the key feature to explain the experimentally observed robust oscillation in this circuit [18].

Delays in a biological circuit arise from the combined processes of intermediate steps like transcription, translation, folding, multimerization and binding to DNA. This series of biological processes are lumped into a single arrow between two genes in the network representation of the circuit. In reality this arrow represents processes that should be modeled in detail. These biological processes can be approximately taken into account by a delay in the interaction term in the dynamical equations. The interaction term can be written as $\delta\ \theta(1 - \psi(t - \tau))$, where τ represents the delay caused by the fact that the process of self-repression is not instant. Therefore, the dynamics of $\psi(t)$ are described by a first-order delay-differential equation (DDE) [18] of the form:

$$\dot{\psi} = -\alpha\psi(t) + \delta\ \theta(1 - \psi(t - \tau)), \tag{27}$$

where τ represents the delay caused by expression process.

We derive analytical solutions to this equation following a procedure outlined in [54] (Chapter V). We start by noting that initial conditions used to solve a DDE are not given

by the value of the function at one point, but rather by a set of values of the function on an interval of length τ. The solution of a DDE can't be thought of as a sequence of values of $\psi(t)$ as in an ODE, but rather as a set of functions $\{f_0(t), f_1(t), f_2(t), \ldots,\}$, defined over a set of contiguous time intervals $\{[-\tau, 0], [0, \tau], [\tau, 2\tau], \ldots,\}$.

Let's consider Eq. (27) with initial function $f_0(t)$ for $t \in [-\tau, 0]$. Then for $t \in [0, \tau]$ Eq. (27) looks like:

$$\dot{\psi} = -\alpha\psi(t) + \delta\ \theta(1 - f_0(t - \tau)). \tag{28}$$

Moving the degradation term to the left and multiplying by $e^{\alpha t}$ we get:

$$\dot{\psi}e^{\alpha t} + \alpha\psi(t)e^{\alpha t} = \delta\ e^{\alpha t}\theta(1 - f_0(t - \tau)). \tag{29}$$

Re-writing the left part, we obtain:

$$\frac{d(\psi e^{\alpha t})}{dt} = \delta\ e^{\alpha t}\theta(1 - f_0(t - \tau)), \tag{30}$$

and integrating on the interval $\int_0^t$, we get:

$$\psi e^{\alpha t} - \psi(0) = \delta \int_0^t e^{\alpha t'}\theta(1 - f_0(t' - \tau))dt\ . \tag{31}$$

Considering that ψ is continuous at 0 ($\psi(0) = f_0(0)$) and $\psi(t)$ for $t \in [0, \tau]$ is given by $f_1(t)$:

$$f_1(t) = f_0(0)e^{-\alpha t} + \delta \int_0^t e^{\alpha(t'-t)}\theta(1 - f_0(t' - \tau))dt, \tag{32}$$

then, following the same procedure, we can derive the general formula for finding the solution $\psi(t)$ on the interval $[k\tau, (k+1)\tau]$, assuming that the solution on the previous interval $[(k-1)\tau, k\tau]$ is given by $f_{k-1}(t)$. We then need to solve the following iterative equation:

$$\dot{\psi} = -\alpha\psi(t) + \delta\ \theta(1 - f_{k-1}(t - \tau)). \tag{33}$$

The solution of this equation can be found by applying the integrating factor method integrating on $\int_{k\tau}^t$. We obtain:

$$\psi(t) = \psi(k\tau) * e^{\alpha(k\tau - t)} + \delta \int_{k\tau}^t e^{\alpha(t'-t)}\theta(1 - f_{k-1}(t' - \tau))dt'\ . \tag{34}$$

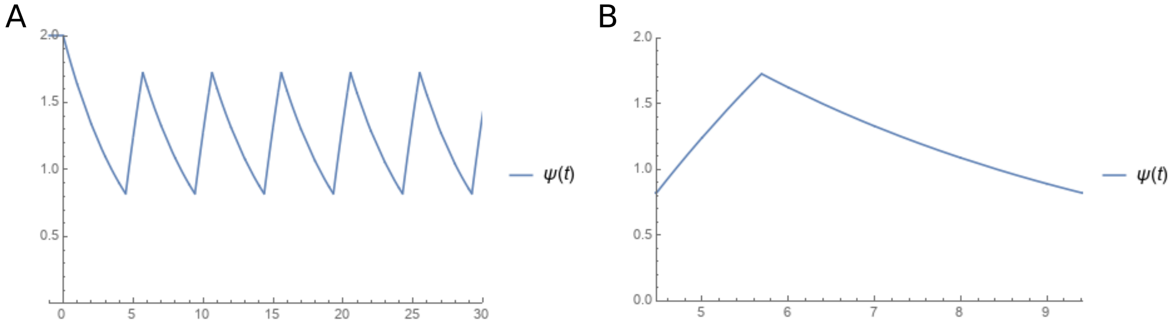

FIG. 2. **UNSAT-FFF delay ODE model. A,** Solution of Eq. (27) using recursive Eq. (34) on $t \in [-\tau, 30\tau]$ for $f_0 = 2$, $\alpha = 0.2$, $\delta = 1$ and $\tau = 1$. **B,** One period of the oscillation of solution in **(A)** consisting of two exponential pieces.

Using Eq. (34) we can recursively find functions $\{f_0(t), f_1(t), f_2(t), \ldots, \}$ on the interval of interest, which provide the solution to Eq. (27). Using Wolfram Mathematica we find functions on the interval $t \in [-\tau, 30\tau]$ for $f_0 = 2$, $\alpha = 0.2$, $\delta = 1$ and $\tau = 1$ and put them together to find the solution plotted in S1 Fig. S2A.

We note from Eq. (34) that all functions f_k are written as the sum of an exponential function and a constant. By looking at Eq. (27), we see that when the Heaviside function is equal to zero, we get a solution that decays exponentially to zero. Likewise, when the Heaviside function is equal to 1, we get a solution that exponentially grows to $\frac{\delta}{\alpha}$. In other words, the solution will grow until $\theta(1 - \psi(t - \tau))$ changes to zero (i.e., when $\psi(t - \tau) > 1$) and will decay until $\theta(1 - \psi(t - \tau))$ changes to one (i.e., when $\psi(t - \tau)$ will cross 1 again, but from different side). Therefore, we get oscillations consisting of two exponential pieces. One period of the oscillation is shown in S1 Fig. S2B. The solution on this interval is given by:

$$\begin{cases} t \in [4.47, 5.69] & \psi(t) = 5 - 10.2 * e^{-0.2t} \\ t \in [5.69, 9.42] & \psi(t) = 5.4 * e^{-0.2t}, \end{cases} \tag{35}$$

which is the predicted behavior. Additionally, we note that this circuit functions as a capacitor charging and discharging in an RC circuit.

B. Period-amplitude relationship

As shown in the main text, the solution of the discrete-time Boolean interaction model for $\lambda > 1$ oscillates in time, as well as the DDE considered in the previous section. Next, we show that this oscillation has a characteristic amplitude and period. First, to compute the amplitude of oscillations A_ψ for the rescaled variable ψ_t, we recall that the iterative map $\psi = f(\psi)$ satisfies the recursive equation:

$$f^t(\psi) = f^{t-1}(\beta\psi)\theta(\psi - 1) + f^{t-1}(\beta\psi + \alpha\lambda)\theta(1 - \psi). \tag{36}$$

Thus, the amplitude of oscillations A_ψ is given by

$$A_\psi = \lim_{\psi\to 1^-} f(\psi) - \lim_{\psi\to 1^+} f(\psi) = \alpha\lambda, \tag{37}$$

which implies that

$$A_\psi = \frac{\gamma_x\gamma_y}{k_y}. \tag{38}$$

To find the period T of the oscillations, we recall from Eq. (6) in the main text that the solution for the minimum value of ψ, $\psi_{\min} < 1$, evolves to its maximum value $\psi_{\max}$ in $T - 1$ iterations as $\psi_{\max} = e^{-(T-1)/\tau}\psi_{\min} + \lambda\left(1 - e^{-(T-1)/\tau}\right)$. Since $\psi_{\min} = (1-\alpha)\psi_{\max}$, due to the fact that $\psi_{\max} > 1$, we find

$$T = \left\lceil 1 + \tau \log\left(1 + \frac{\alpha}{\lambda - 1}\right)\right\rceil, \tag{39}$$

where we used $\psi_{max} = 1$. For example, using $\alpha = 0.2$ and $\lambda = 1.01$, we find $A_\psi = 2.02$ and $T = 15$, which agrees with the numerical simulation presented in S1 Fig. S3A.

Equation (39) allows to define a rescaled amplitude $\mathcal{A} = (\lambda - 1)/\alpha$, and a reduced period $\mathcal{T} = (T-1)/\tau$ such that

$$\mathcal{T} = \log\left(1 + \frac{1}{\mathcal{A}}\right), \tag{40}$$

which corresponds to the *period-amplitude relationship* of the UNSAT-FFF. A plot of this relationship is shown in S1 Fig. S3B, where we plot $(T-1)/\tau$ as a function of $\left[(\gamma_x\gamma_y/\alpha k_y) - 1\right]/\alpha$.

Coming back to the original variable $y_t = k_y\psi_t$, we have that the amplitude of oscillations of y_t, $A = k_y A_\psi$, is given by:

$$A = \gamma_x\gamma_y, \tag{41}$$

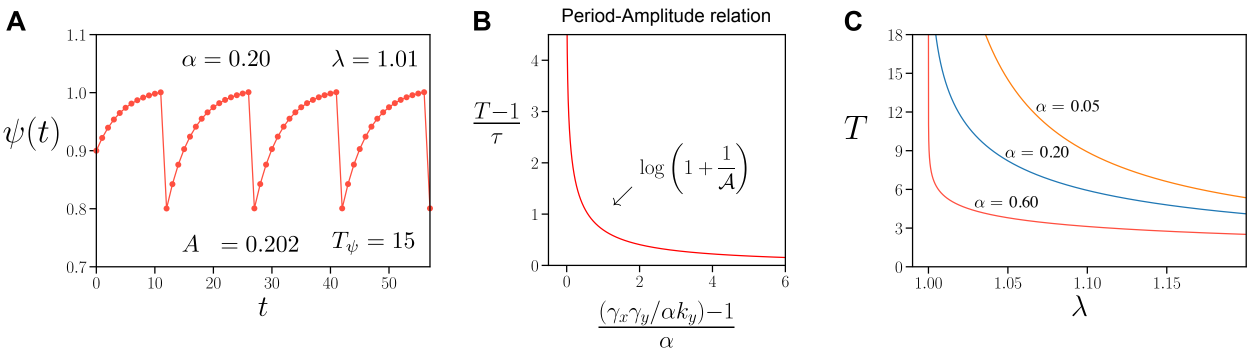

FIG. 3. **Period-amplitude relationship.** **a,** Solutions for $\alpha = 0.2$ and $\lambda = 1.01$. The values for $A_\psi = 0.202$ and $T = 15$ obtained with the use of Eq. (37) and Eq. (39) perfectly agree with the ones found by numerical simulations. **b,** Period-amplitude relationship in terms of the original set of parameters α, k_y, γ_x, and γ_y. **c,** Period of oscillations as a function of λ for different values of α.

and from Eq. (39), we can write the period of oscillations as a function of the original set of parameters as

$$T = 1 - \frac{1}{\log(1-\alpha)} \log\left(1 + \frac{\alpha}{(\gamma_x\gamma_y/\alpha k_y) - 1}\right). \tag{42}$$

S1 Figure S3C shows the period of oscillations T as a function λ for $\alpha = 0.60$, $\alpha = 0.20$, and $\alpha = 0.05$.

C. UNSAT-FFF clock functionality

The clock functionality of the UNSAT-FFF can be understood by analyzing its response function, i.e. the relation between oscillations at the input and at the output of the circuit. The amplitude A_y, and period T of the oscillations are not independent like in the harmonic oscillator, but are related through a 'period-amplitude' relation expressed by Eq. (40) and S1 Fig. S3B. From Eq. (42), for α sufficiently small,

$$T - 1 \sim \frac{k_y}{\gamma_x\gamma_y} = \frac{1}{A} \,, \tag{43}$$

which constrains the 'clock' (T) of the circuit to the power (A_y). As a consequence, A_y and T cannot be controlled arbitrarily, and this (A-T) constraint helps to stabilize the UNSAT-FFF response against disturbance in the input X. For example, for a given available power supply, the system is constrained to dissipate this power, and when the UNSAT-FFF

oscillates, it is automatically set to operate on an extended time window (T large) at low amplitude A when a small expression level is required (A small) and vice-versa. Results for the clock functionality of the Fibonacci Fiber and $n = 2$ Fiber can be carried out in a similar manner. The idea is that Fibonacci fibers with longer and longer loops can carry more robust oscillatory patterns than simple autoregulation negative feedback loops.

IV. EXAMPLES OF SYMMETRY AND BROKEN SYMMETRY CIRCUITS

A. Symmetry circuits (*fibers*) from [24]

Our findings show that simple sub-graphs ubiquitous on gene regulatory networks are analogous to symmetric electronic circuits which can work as clocks, revealing a hierarchy of symmetry circuits. Here, we describe these symmetric circuits in more detail following [24]. Supplementary File 1 presents the full list of circuits found across the regulatory networks of *A. thaliana*, *M. tuberculosis*, *B. subtilis*, *E. coli*, *salmonella*, *yeast*, mouse and humans. Below we enumerate the set of symmetric *fibers* found in Ref. [24] in the genetic networks of these species.

1. Repressor regulator link (stub)

We start by an isolate repressor link. As depicted in Fig. 3A, this repressor regulator alone does not form an input tree, neither it forms a *base*. Since it works as a transistor, its logic representation is a NOT gate.

2. Repressor AR loop: $|1, 0\rangle$

In Fig. 3B, we show the repressor autoregulation loop. When a repressor link is found as an AR loop, we have a genetic network with one single loop ($n = 1$) and no external regulator ($\ell = 0$), which we denote symbolically by $|1, 0\rangle$. Such simple network has an input tree that feeds its own expression levels. Also, it is equivalent to its own *base*. By the analysis of the corresponding logical circuit, one can observe that it naturally oscillates, since it is a NOT gate that feeds itself.

3. UNSAT-FFF: $|1,1\rangle$

The repressor autoregulation loop introduces a symmetry between Y and Z that allows the expression levels y_t and z_t to synchronize and oscillate. The increase of external regulators does not affect the complexity of UNSAT-FFF, since its dynamics remains restricted to the sole loop on the network. This can be verified in the corresponding input tree and in its *base*. The collapse of Z into Y forms a *base* with $n = 1$ autoregulator and $\ell = 1$ external regulator. Because of the repressor feedback, the oscillation and synchronization of Y and Z are again evident in its logic circuit. In Fig. 3C, we use the NAND gate for representing gene Y and an AND gate for gene Z, but other gates (such as an OR logic gate) would result in similar conclusions.

4. Fibonacci Fiber: $|1.6180..., 0\rangle$

The Fibonacci Fiber shown in Fig. 3D is characterized by the addition of a second feedback loop. This regulatory network have a Fibonacci sequence $Q_t = Q_{t-1} + Q_{t-2}$ as an input tree. The branching structure of the input tree implies that the Fibonacci Fiber can store memory dynamically by the interaction with its past states, although it is continually erased in time. As shown in the logic circuit of Fibonacci Fiber, its structure can oscillate and synchronize, but is unable to store static information. This situation changes as soon as we allow symmetries to break, which leads to a number of genetic circuits, as those depicted in Fig. 4. In Fig. 3D, we show examples of the Fibonacci Fiber on regulatory networks of various species. Note the presence of its *base* in the network representation for *B. subtilis* (genes *tnrA* and *glnR*), *E. coli* (genes *uxuR* and *exuR*), *M. tuberculosis* (genes Rv0182c and Rv3286c), *Salmonella* (genes *marA* and *soxS*), *Yeast* (genes Tec1 and Ste12), and in two networks from human genetic network (the pair genes *PAX5* and *TP53*, for the first example, and gene *FOS* and *CREM*, for the second).

5. $n = 2$ Fiber: $|2, 0\rangle$

Starting from a Fibonacci Fiber, the addition of a second autoregulation on gene X results in a symmetric input tree. Besides, the genetic network of the $n = 2$ Fiber collapses into a *base* with a single gene with two autoregulations (Fig. 3E). From the corresponding logic

circuit, one can conclude that it is possible to achieve synchronization between genes X, Y and Z. In Fig. 3E we show examples of the $n = 2$ Fiber from the regulatory networks of *B. subtilis* (the first with the pair of genes *lexA* and *rocR*, and the second with genes *hprT*, *tilS* and *ftsH*).

B. Symmetry breaking circuits

Figure 4 shows the procedure to generate broken symmetry circuits. As we discuss in the main text, this process starts by a replica symmetry operation where the *base* from a given symmetry circuit is duplicated. The symmetry is broken by the addition of two input genes as regulators of the circuit. The resulting broken symmetry circuits are analogous to flip-flops circuits from digital electronics. Such circuits are able to store memory statically, playing a central role in the design of microprocessors. In what follows, we describe the AR, FFF, and Fibonacci broken symmetry circuits and their analogous electronic circuits in detail. Supplementary File 1 presents the full list of circuits found across the regulatory networks of *A. thaliana*, *M. tuberculosis*, *B. subtilis*, *E. coli*, *salmonella*, *yeast*, mouse and humans.

1. *AR symmetry breaking circuit: SR flip-flop*

Through a replica symmetry duplication, gene Y 'opens-up' its AR loop into two mutually regulated genes Y and Y'. We break the symmetry relation between Y and Y' by the inclusion of different inputs S and R as depicted in Fig. 4 (replica symmetry breaking), such that S $\neq$ R.

The SR flip-flop does not provide synchronized outputs. After the input signals arrive at the logic gates, each gate provides its output without waiting for the output of the other. This results in fast oscillations which, in the particular application to integrated circuits in digital electronics, are undesired. Then, in digital electronics, the input $S = 0$ and $R = 0$ is said to be forbidden, since the NAND gates set both $Q = 1$ and $\overline{Q} = 1$, which violates the logical state $\overline{Q} = \textbf{not } Q$. In Fig. 4, we use the NAND gates, but the use of a NOR gates leads to similar conclusions.

Two biological realizations of the AR symmetry breaking class are shown in Fig. 4,

both from human regulatory networks with genes *NFKB1* and *HOXA9* (upper), and the regulatory network of genes *IRF4* and *BCL6* (bottom). Gene *NFKB1* further regulates two genes, *BST1* and *HAX1* as its outputs, but this regulation does not affect the functionality of the flip-flop.

2. *FFF symmetry breaking circuit: Clocked SR flip-flop*

Following the same strategy, we start with the *base* of the UNSAT-FFF and symmetrize it through a replica symmetry duplication. Note that the replica symmetry of FFF adds a second level of NAND logic gates to the SR flip-flop via gene X. In order to have consistent logic operations, we add an input clock gene CLK which symbolizes the activation of gene X, since gene X needs to receive input for its activation. The resulting circuit is analogous to the Clocked SR flip-flop after the addition of the input genes S and R. The second level flip-flop inverts the outputs of the previous SR flip-flop logic circuit, meaning that when $S = 1$ and $R = 0$ ($S = 0$ and $R = 1$), the circuit outputs $Q = 1$ and $\overline{Q} = 0$ ($Q = 0$ and $\overline{Q} = 1$). The input $S = 0$ and $R = 0$ results in an unchanged state. When the input of gene CLK is low, $CLK = 0$, the output of the second level of both NAND gates outputs high signals, independently of the values of S and R, assuring that the outputs Q and $\overline{Q}$ remain unchanged. However, when the clock input is $CLK = 1$, it allows the first level of NAND gates to change the outputs for $S \neq R$. The clocked SR flip-flop also has a forbidden state when $S = 1$ and $R = 1$ in digital electronics. In Fig. 4, we show two biological realizations of the FFF broken symmetry breaking circuit, the set of genes {*CLOCK, NR0B2, NR3C1, E2F1* and *TP53*} and the set {*CEBPB, DDIT3, PRDM1, CEBPA* and *MYC*}, both examples are from human regulatory genetic networks. The outputs of the flip-flops, like *E2F1* and *TP53*, further regulate a set of genes each as indicated by the red genes in the figure. These regulatory interactions do not affect the functionality of the flip-flops since they are outgoing links.

3. *Fibonacci symmetry breaking circuit: JK flip-flop*

The replica symmetry duplication of the Fibonacci Fiber results in a logic circuit similar to the FFF broken symmetry breaking circuit. However, the regulation links $Y \to X'$ and

$Y' \to X$ yields a different logic circuit, which is analogous to the Clocked JK flip-flop, where the input genes are now J and K. The additional links solve the unpredictable output for the $J = 1$ and $K = 1$ case by commuting the values stored in Q and $\overline{Q}$. Two examples of the Fibonacci broken symmetry circuits are shown in Fig. 4 for the sets of genes {*PITX1, JUN, NKX3-1, TP53* and *ESR1*} and {*FLI1, HDAC1, EPS300, AR* and *RELA*}, both from human genetic networks. We also show the set of genes regulated by these flip-flops.

V. DESCRIPTION OF DATASETS

Datasets are described in S1 Table 1. We use a set of datasets of transcriptional regulatory networks found in the literature. All datasets are freely available from online sources. In Supplementary File 1 we present a plot of all found circuits. In the case of symmetric circuits, same colored nodes indicate the genes in the *fiber*. The external regulators are colored with different colors. We also present all the symmetry broken circuits across all species as indicated in the file. The statistics, count and Z-scores of the circuits are presented in Table 1 and 2 in the main text. The file with all circuits can be found at `https://bit.ly/2YM5x3H`.

VI. ALGORITHM TO FIND FIBERS

To obtain the set of nodes in the graph that belong to a *fiber*, we use the algorithm described in detail by Kamei and Cock [41] and developed in Ref. [24] to obtain the '*minimal balanced coloring*' of the graph (referred as balanced coloring for simplicity), where we color the network by assigning a different color to each *fiber*.

To understand what balanced coloring means in the context of graph theory, we need to define the concept of input set (which is a part of the input tree). In a directed graph, the input set of a given node is the set of nodes with edges pointing into that node. Thus, the input set is the first layer of the input tree. Next, we define the Input Set Color Vector (ISCV) of a node as a vector of length equal to the number of colors in the graph, that is the number of *fibers*. Each entry of the ISCV of a given node counts how many nodes of each color are in the input set of this node. The balanced coloring is achieved by iteration by increasing the number of colors (length of ISCV) until all nodes of the same color have the same ISCVs. At this point, each color identifies each *fiber* in the graph.

Species	Database	Additional information
Arabidopsis Thaliana	ATRM [32]	We use high-confidence functionally confirmed transcriptional regulatory interactions from the ATRM database of the broadly used model plant Arabidopsis. `http://atrm.cbi.pku.edu.cn/`
Micobacterium Tuberculosis	Research article [33]	Supplementary Information of Ref. [33] `https://www.ncbi.nlm.nih.gov/pmc/articles/PMC2600667/bin/msb200863-s2.xls`
Bacillus subtilis	SubtiWiki [34]	We download the database from SubtiWiki website and consider all repressor and activation links as "Repression" and "Activation". This database is considered the primary source of information for Bacillus. `http://subtiwiki.uni-goettingen.de/`
Escherichia coli	RegulonDB [35]	We use the TF - operon interaction network from [35]. RegulonDB combines transcriptional regulator interactions obtained by curating literature and using NLP high-quality data and partially confirmed experimentally and computationally predicted data. `http://regulondb.ccg.unam.mx/`
Salmonella SL1344	SalmoNet [36]	We use the regulatory layer of the strain Salmonella Typhimurium SL1344. SalmoNet consists of manually curated low-throughput and high-throughput experiments and predictions based on experimentally verified binding sites and TF-gene binding site data from RegulonDB. `http://salmonet.org/`
Yeast	YTRP [38]	We use the TF-gene regulatory and TF-gene binding networks. Results of the TFPEs (Transcription Factor Perturbation Experiments) identify the regulatory targets of TFs. This is further refined by using literature-curated data. `http://cosbi3.ee.ncku.edu.tw/YTRP/Home`
Mouse	TRRUST [39]	Downloaded from TRRUST website. TRRUST is constructed using sentence-based text mining of more than 20 million abstracts from research articles, refined by manual curation. `https://www.grnpedia.org/trrust/`
Human	TRRUST [39]<br>TRRUST_2 [39]<br>KEGG [40]	Downloaded from TRRUST website.<br>Downloaded from TRRUST website and curated.<br>We use KEGG API to download all pathways of Human gene regulatory network. After that, all networks are put together and duplicates are removed. `https://www.genome.jp/kegg/pathway.html`

TABLE 1. **Description of dataset acquisition.** All data are gathered from publicly available sources [32–40].

Finding graph balanced coloring is equivalent to finding node sets with isomorphic input trees. A brief explanation of that is the following. If two nodes have the same ISCVs,

nodes of their input sets have same colors. Thus these nodes are said to belong to the same equivalence class [41]. Inductively, the same can be said of the input set of the nodes in the input set. This recurrent relation implies that two nodes that have the same input sets will have isomorphic input trees and will belong to the same *fiber* (for rigorous proof see chapter 4 in [42]).

The algorithm to find balanced coloring was described in detail by Kamei and Cock [41]. In their algorithm, all nodes of the graph have the same initial color and, through a series of operations, they are recolored until balanced coloring is reached. The detailed description of the algorithm is as follows:

1. Initially, all nodes are assigned the same color.
2. Each node is assigned with the N-dimensional vector (ISCV), where N is current number of colors at the current iteration of the algorithm. Each entry of this vector is the number of nodes of certain color with ingoing link to the respective node. In the first iteration, N = 1 and each entry is the in-degree of the node.
3. If vectors for all nodes of the same color are equal, the balanced coloring is achieved and the algorithm stops.
4. Otherwise, if coloring is unbalanced, each unique vector is assigned a new color and the graph is recolored accordingly.
5. Steps 2-4 are repeated until condition in Step 3 is satisfied.

For example consider the FFF graph in S1 Fig. S4A. Initially, we assign the same color, white, to all nodes. Then, we assign a 1-dimensional vector to each node which counts the in-degree of each node (S1 Fig. S4B). Since ISCVs of X and Y (which have the same color) are different, ISCV(X) = 0 and ISCV(Y) = 2, where the entry refers to the number of inputs of white color, then the condition in Step 3 is not satisfied. There are two unique ISCVs, thus only two new colors are necessary. Thus, a 2-dimensional vector is assigned to each node: ISCV(X) $= (0, 0)$, ISCV(Y) $= (1, 1)$, and ISCV(Z) $= (1, 1)$. Here, the first entry refers to the number of inputs of green color and the second entry of red color. Thus, each entry of this vector is related to a new color, for example, red and green (see S1 Fig. S4D). Then, the network is recolored accordingly, as depicted in S1 Fig. S4C. At this step, ISCV(Y)

and ISCV(Z) are the same, and different from the ISCV(X), and both have the same color, also different from the color of X, therefore balanced coloring is reached and the algorithm stops. We provide an implementation of this algorithm at `https://github.com/makselab/fiberCodes`.

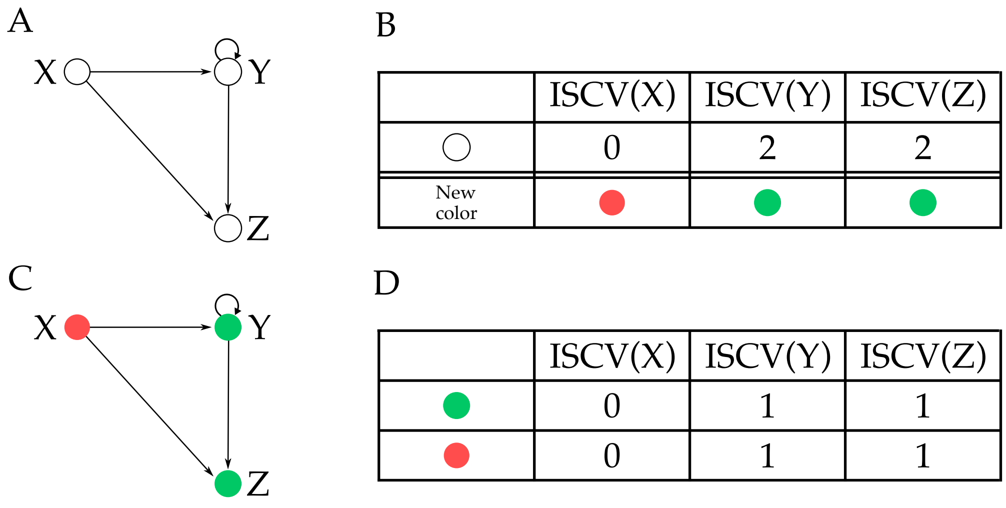

FIG. 4. Illustration of the balanced coloring algorithm to find *fibers*.

VII. ALGORITHM TO FIND BROKEN SYMMETRY CIRCUITS

To count the number of occurrences of broken symmetry circuits in a given graph we count the number of appearances of induced subgraphs as defined in Refs. [43–45]. Subgraphs and induced subgraphs are graph-theoretical concepts introduced in the social and computer sciences as applications to graph matching and pattern recognition [44]. We allow symmetry breaking to come from any gene in the circuit. Let's consider a graph $G = \{V, E\}$, where V is the set of nodes and E is the set of links. For instance in S1 Fig. S5A $V = \{A, B, C, D, Y, Y'\}$ and $E = \{A \to Y, Y \to B, Y \to Y', Y' \to Y, Y' \to C, D \to Y'\}$. A *subgraph* $G' = \{V', E'\}$ of a graph $G = \{V, E\}$ is a graph such that $V' \subset V$ and $E' \subset E$ [43]. For example S1 Fig. S5B is a subgraph of the graph in S1 Fig. S5A with $V' = \{Y, Y'\} \subset V$ and $E' = \{Y' \to Y\} \subset E$. An *induced subgraph* $G' = \{V', E'\}$ of a graph $G = \{V, E\}$ is a subgraph with a set of nodes $V' \subset V$ and all links $E' \subset E$ such that their heads and tails are in V'. For example the subgraph of S1 Fig. S5B is not induced graph of G since it is missing the link $\{Y \to Y'\}$. That is, S1 Fig. S5B is not an induced subgraph (but it is just a subgraph of G), because one

of the links $\{Y \to Y'\}$ with endpoints in V' is missing. However the graph of S1 Fig. S5C is an induced subgraph of G since $V' = \{Y, Y'\}$ and $E' = \{Y \to Y', Y' \to Y\}$ are included, but links $\{A \to Y, Y \to B, Y' \to C, D \to Y'\}$ don't belong to G'. The problem of finding broken symmetry circuits consists on three steps: (1) identify a *base* and create a replica symmetry circuit, (2) find subgraphs isomorphic to the replica symmetry circuit, and (3) remove subgraphs that are not induced.

The first step is to find subgraphs isomorphic to the replica symmetry circuit. Let's consider an example. The matrix in S1 Fig. S5D represents the adjacency matrix A of the symmetric part of the SR flip-flop (i.e., a toggle switch). That is, A is the replica symmetry part (Fig. 4, third row) of the broken symmetry circuit. Similarly, S1 Fig. S5G,H show the adjacency matrices of clocked SR flip-flop and JK flip-flop circuits. The general idea is to choose a subgraph and check if it is isomorphic to the circuit and continue doing this for all possible subgraphs in the entire network. However, this task is computationally expensive. Even for circuits with 5 nodes, the computational time is N^5, where N is the total number of nodes in G, which means that for big enough graphs the algorithm can take very long computational time. Different approaches to this problem have been widely studied and are nicely reviewed in Ref. [46]. Time costs can be cut if unprofitable paths are identified and skipped in the search space. One of the recent works in the field is the VF2 algorithm developed by Cordella *et al.* [47]. It is designed to deal with large graphs and uses state of the art techniques in order to reduce computational time. We use the algorithm implemented in a popular R package igraph [48] as a function *subgraph_isomorphisms*(...). We provide the analysis and plotting scripts allowing to reproduce our results at `https://github.com/makselab/CircuitFinder`.

The second step is to remove all the subgraphs that are not induced or, simply speaking, have extra links between the genes in the broken symmetry circuit. We follow this procedure: take a node set identified above, find the induced subgraph of the complete graph with this node set and compare the adjacency matrix of the induced subgraph with the adjacency matrix of the circuit. If the matrices are different, then the circuit is removed. All remaining circuits are the broken symmetry flip-flops that we are looking for. Multi-links and self-loops are removed from the network prior to consideration.

By applying the steps described above we get the full list of induced subgraphs that are isomorphic to the given circuit.

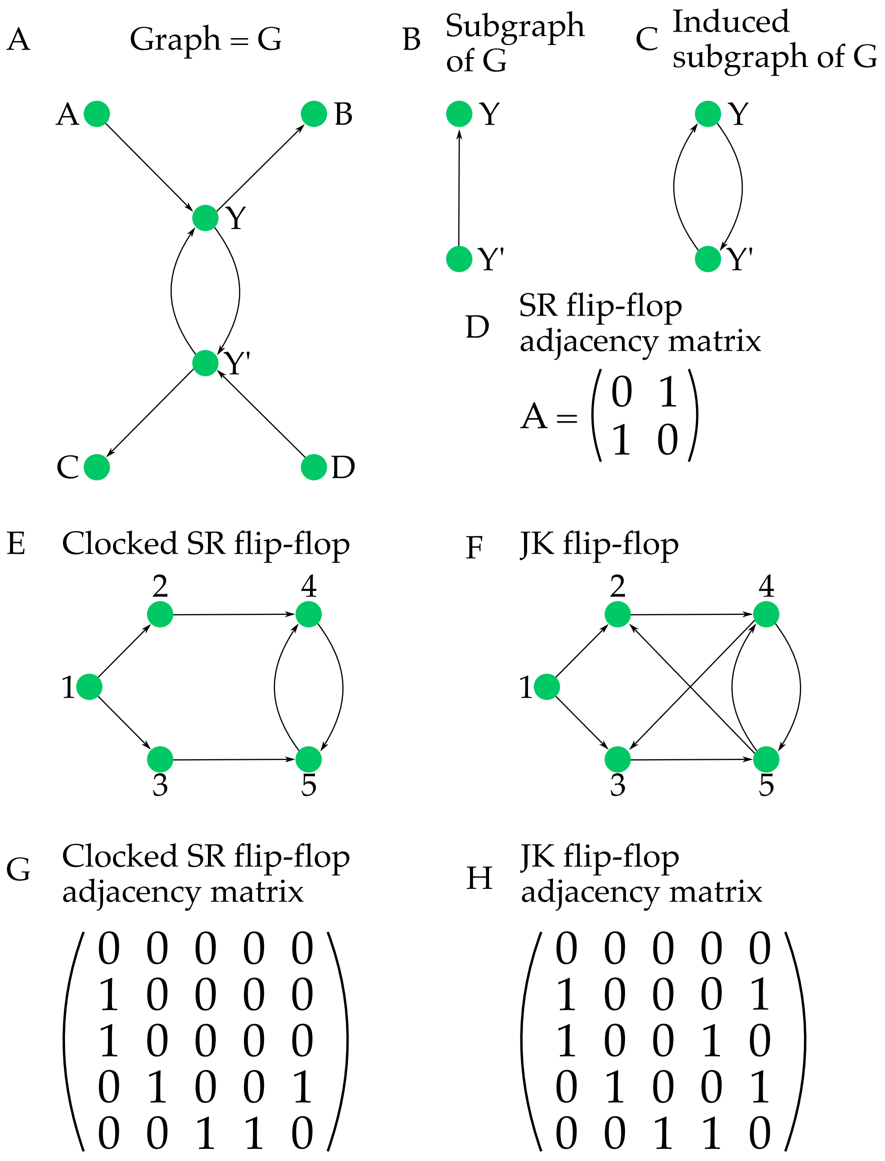

FIG. 5. Definition of the induced subgraph used to obtain the broken symmetry circuits. **A,** Graph G. **B,** Subgraph of G. **C,** Induced Subgraph of G. **D,** Adjacency matrix of SR flip-flop. **E,** Clocked SR flip-flop. **F,** JK flip-flop. **G,** Adjacency matrix of Clocked SR flip-flop. **H,** Adjacency matrix of JK flip-flop.

[1] Hartwell LH, Hopfield JJ, Leibler S, Murray AW. From molecular to modular cell biology. Nature. 1999;402: C47-C52.

[2] Milo R, Shen-Orr SS, Itzkovitz S, Kashtan N, Chklovskii D, Alon U. Network motifs: simple building blocks of complex networks. Science. 2002;298: 824-827.

[3] Shen-Orr SS, Milo R, Mangan S, Alon U. Network motifs in the transcriptional regulation network of *Escherichia coli*. Nature Genet. 2002;31: 64-68.

[4] Alon U. An Introduction to Systems Biology: Design Principles of Biological Circuits. Boca Raton: CRC Press; 2006.

[5] Klipp E, Liebermeister W, Wierling C, Kowald A, Herwig R. Systems Biology: a textbook. Weinheim: Wiley-VCH; 2016.

[6] Tyson JJ, Chen KC, Novak B. Sniffers, buzzers, toggles and blinkers: dynamics of regulatory and signaling pathways in the cell. Curr Opin Cell Biol. 2003;15(2): 221-31.

[7] Monod J, Jacob F. General conclusions: teleonomic mechanisms in cellular metabolism, growth and differentiation. Cold Spring Harb Symp Quant Biol. 1961;26: 389-401.

[8] Teo JY, Woo SS, Sarpeshkar R. Synthetic Biology: A Unifying View and Review Using Analog Circuits. IEEE Trans. on Biomed. Circuits and Syst. 2015;9: 453-474.

[9] Dalchau N, Szé G, Hernansaiz-Ballesteros R, Barnes CP, Cardelli L, Phillips A, et al. Computing with biological switches and clocks. Natural Computing. 2018;17: 761-779.

[10] Atkinson MR, Savageau MA, Myers JT, Ninfa AJ. Development of genetic circuitry exhibiting toggle switch or oscillatory behavior in *Escherichia coli*. Cell. 2003;113: 597-607.

[11] Gardner TS, Cantor CR, Collins JJ. Construction of a genetic toggle switch in *Escherichia coli*. Nature. 2000;403: 339-342.

[12] Kramer BP, Fussenegger M. Hysteresis in a synthetic mammalian gene network. Proc Natl Acad Sci USA. 2005;102: 9517-9522.

[13] Kramer BP, Viretta AU, Baba MD-E, Aube D, Weber W, Fussenegger M. An engineered epigenetic transgene switch in mammalian cells. Nature Biotech. 2004;22: 867-870.

[14] Guet CC, Elowitz MB, Hsing W, Leibler S. Combinatorial synthesis of genetic networks. Science. 2002;296: 1466-1470.

[15] Ajo-Franklin CM, Drubin DA, Eskin JA, Gee EP, Landgraf D, Phillips I, et al. Rational design

of memory in eukaryotic cells. Genes Dev. 2007;21: 2271-2276.

[16] Ham TS, Lee SK, Keasling JD, Arkin AP. Design and construction of a double inversion recombination switch for heritable sequential genetic memory. PLoS One. 2008;3: e2815.

[17] Elowitz MB, Leibler S. A synthetic oscillatory network of transcriptional regulators. Nature. 2000;403: 335-338.

[18] Stricker J, Cookson S, Bennett MR, Mather WH, Tsimring LS, Hasty J. A fast, robust and tunable synthetic gene oscillator. Nature. 2008;456: 516-519.

[19] Tigges M, Marquez-Lago TT, Stelling J, Fussenegger M. A tunable synthetic mammalian oscillator. Nature. 2009;457: 309-312.

[20] Mangan S, Alon U. Structure and function of the feed-forward loop network motif. Proc Natl Acad Sci USA. 2003;100: 11980-11985.

[21] Mangan S, Zaslaver A, Alon U. The coherent feedforward loop serves as a sign-sensitive delay element in transcription networks. J Mol Biol. 2003;334: 197-204.

[22] Glass L, Kauffman SA. The logical analysis of continuous, non-linear biochemical control networks. J Theor Biol. 1973;38: 103-129.

[23] Mangan S, Zaslaver A, Alon U. Negative autoregulation increases the input dynamic-range of the arabinose system of *Escherichia coli*. BMC Sys Biol. 2011;5: 111.

[24] Morone F, Leifer I, Makse HA. Fibration symmetries uncover the building blocks of biological networks. Preprint at `https://bit.ly/2Z94B6o` (2019).

[25] Golubitsky M, Stewart I. Nonlinear dynamics of networks: the groupoid formalism. Bull Am Math Soc. 2006;43: 305-364.

[26] Boldi P, Vigna S. Fibrations of graphs. Discrete Mathematics. 2001;243: 21-66.

[27] Anderson PW. The concept of frustration in spin glasses. J of the Less-Common Metals. 1978;62: 291-294.

[28] Widlar RJ, Some circuit design techniques for linear integrated circuits. IEEE Trans Circuit Theory. 1965;4: 586-590. See also Widlar RJ: US Patent Number 3,320,439; Filed May 26, 1965; Granted May 16, 1967: Low-value current source for integrated circuits and Widlar RJ. Design techniques for monolithic operational amplifiers. IEEE Solid-State Circuits. 1969;4: 184-191.

[29] Horowitz P, Hill W. The Arts of Electronics. 3rd ed. New York:Cambridge University Press; 2015.

[30] Weinberg S. The Quantum Theory of Fields. Cambridge: Cambridge University Press; 2005.

[31] Morone F, Makse HA. Symmetry group factorization reveals the structure-function relation in the *Caenorhabditis elegans* connectome. Nat Commun. 2019;10: 4961.

[32] Jin J, He K, Tang X, Zhe L, Lv L, Zhao Y, et al. An Arabidopsis transcriptional regulatory map reveals distinct functional and evolutionary features of novel transcription factors. Mol Biol Evol. 2015;32: 1767-1773.

[33] Balázsi G, Heath AP, Shi L, Gennaro ML. The temporal response of the Mycobacterium tuberculosis gene regulatory network during growth arrest. Mol Syst Biol. 2008;4: 225.

[34] Zhu, B. & Stülke J. SubtiWiki in 2018: From genes and proteins to functional network annotation of the model organism Bacillus subtilis. Nucleic Acids Res. 2017;46: D743-D748.

[35] Gama-Castro S, Salgado H, Santos A, Ledezma-Tejeida D, Muñiz-Rascado L, Santiago G-S, et al. RegulonDB version 9.0: High-level integration of gene regulation, coexpression, motif clustering and beyond. Nucleic Acids Res. 2015;44: D133-D143.

[36] Métris A, Sudhakar P, Fazekas D, Demeter A, Ari E, Olbei M, et al. SalmoNet, an integrated network of ten Salmonella enterica strains reveals common and distinct pathways to host adaptation. NPJ Systems Biology and Applications. 2017;3: 31.

[37] Cherry JM, Hong EL, Amundsen C, Balakrishnan R, Binkley G, Chan E, et al. Saccharomyces Genome Database: The genomics resource of budding yeast. Nucleic Acids Res. 2011;40: D700-D705.

[38] Yang T-H, Wang C-C, Wang Y-C, Wu W-S. YTRP: a repository for yeast transcriptional regulatory pathways. Database 2014;2014: bau014.

[39] Han H, Cho J-W, Lee S, Yun A, Kim H, Bae D, et al. TRRUST v2: An expanded reference database of human and mouse transcriptional regulatory interactions. Nucleic Acids Res. 2017;46: D380-D386.

[40] Kanehisa M, Sato Y, Kawashima M, Furumichi M, Tanabe M. KEGG as a reference resource for gene and protein annotation. Nucleic Acids Res. 2015;44: D457-D462.

[41] Kamei H, Cock PJ. A. Computation of balanced equivalence relations and their lattice for a coupled cell network. SIAM J Appl Dyn Syst. 2013;12: 352-382.

[42] Aldis JW. A polynomial time algorithm to determine maximal balanced equivalence relations. Int J Bifurc Chaos Appl Sci Eng. 2008;18: 407-427.

[43] Weisstein EW, "Subgraph". From MathWorld–A Wolfram Web Resource. `http://`

mathworld.wolfram.com/Subgraph.html

[44] Harary F. Graph Theory. Reading: Addison-Wesley; 1994.

[45] https://en.wikipedia.org/wiki/Induced_subgraph

[46] Conte D, Foggia P, Sansone C, Vento M. Thirty years of graph matching in pattern recognition. Int J Pattern Recognit Artif Intell. 2004;18: 265-298.

[47] Cordella LP, Foggia P, Sansone C, Vento M. A (sub)graph isomorphism algorithm for matching large graphs. IEEE Trans Pattern Anal Mach Intell. 2004;26: 1367-1372.

[48] Csardi G, Nepusz T. The Igraph software package for complex network research. Inter Journal, Complex Systems. 2006;1695: 1-9.

[49] Ingram PJ, Stumpf MP, Stark J. Network motifs: structure does not determine function. BMC Genomic. 2006;7: 108.

[50] Payne JL, Wagner A. Function does not follow form in gene regulatory circuits. Sci Rep. 2015;5: 13015.

[51] Macía J, Widder S, Solé R. Specialized or flexible feed-forward loop motifs: a question of topology. BMC Syst Biol. 2009;3: 84.

[52] Ahnert SE, Fink TMA. Form and function in gene regulatory networks: the structure of network motifs determines fundamental properties of their dynamical state space. J Royal Soc Interface. 2016;13: 20160179.

[53] Purcell O, Savery N, Grierson C, Di Bernardo M. A comparative analysis of synthetic genetic oscillators. J Royal Soc Interface. 2010;7: 1503-24.

[54] Driver RD. Ordinary and delay differential equations. New York: Springer Verlag; 1977.
