# Supplementary material for: Circuits with broken fibration symmetries perform core logic computations in biological networks
Source: PLoS Comput Biol. 2020 Jun 17;16(6):e1007776. doi: 10.1371/journal.pcbi.1007776 (PMC7299331; doi:10.1371/journal.pcbi.1007776)

Ecoli

SR flip-flop

CSPA; HNS

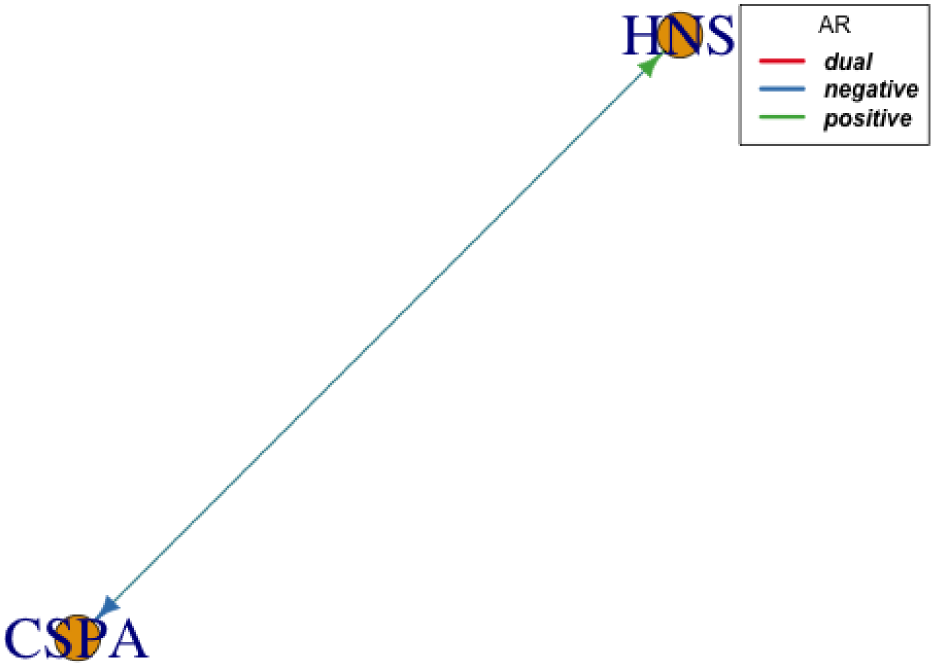

Human TRRUST

Clocked SR flip-flop

KAT2B; SMAD4; E2F1; JUN; AR

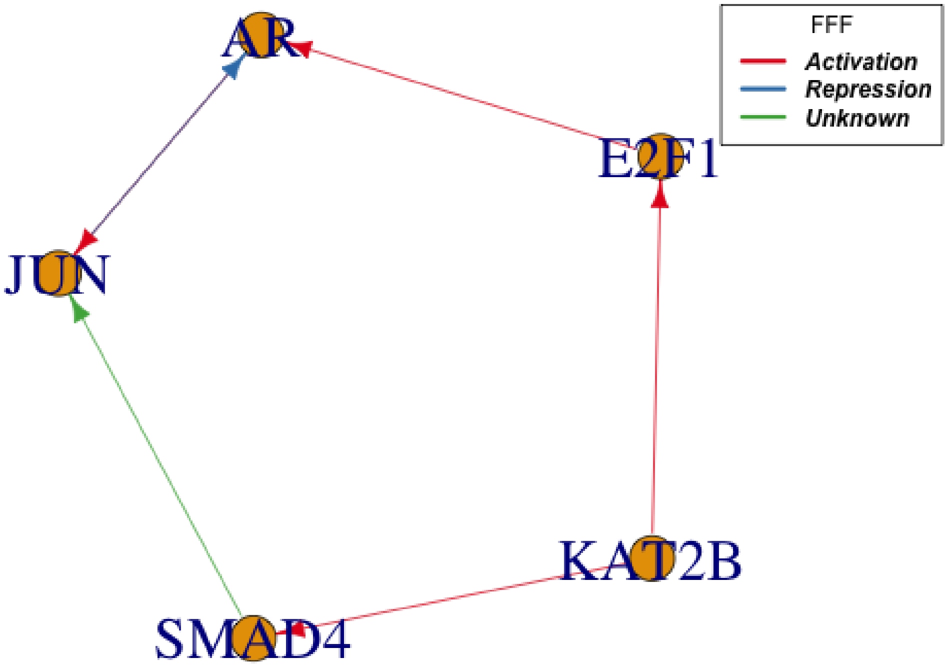

Human TRRUST

Clocked SR flip-flop

CEBPB; SMAD3; DDIT3; MYC; CEBPA

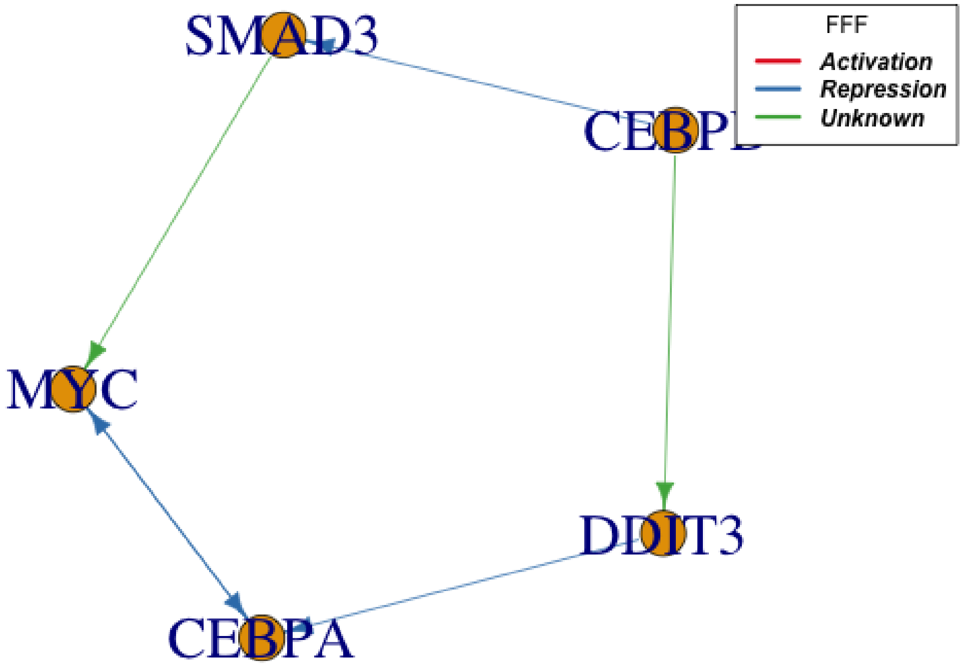

Human TRRUST

Clocked SR flip-flop

CREB1; JUN; ETV3; TP53; RELA

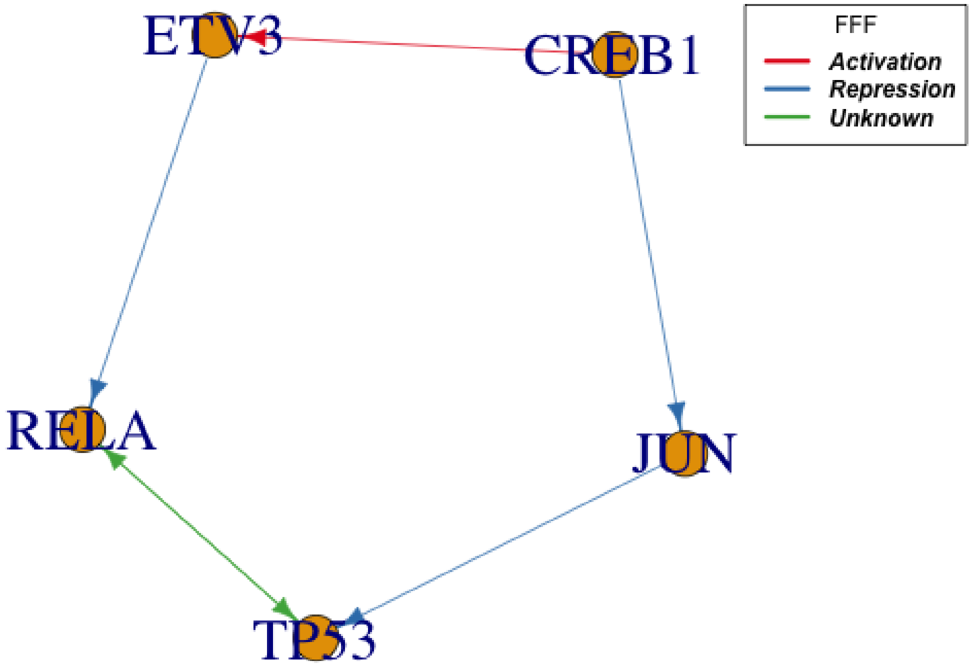

Human TRRUST

Clocked SR flip-flop

ARNT; HIF1A; JUN; TWIST1; RUNX2

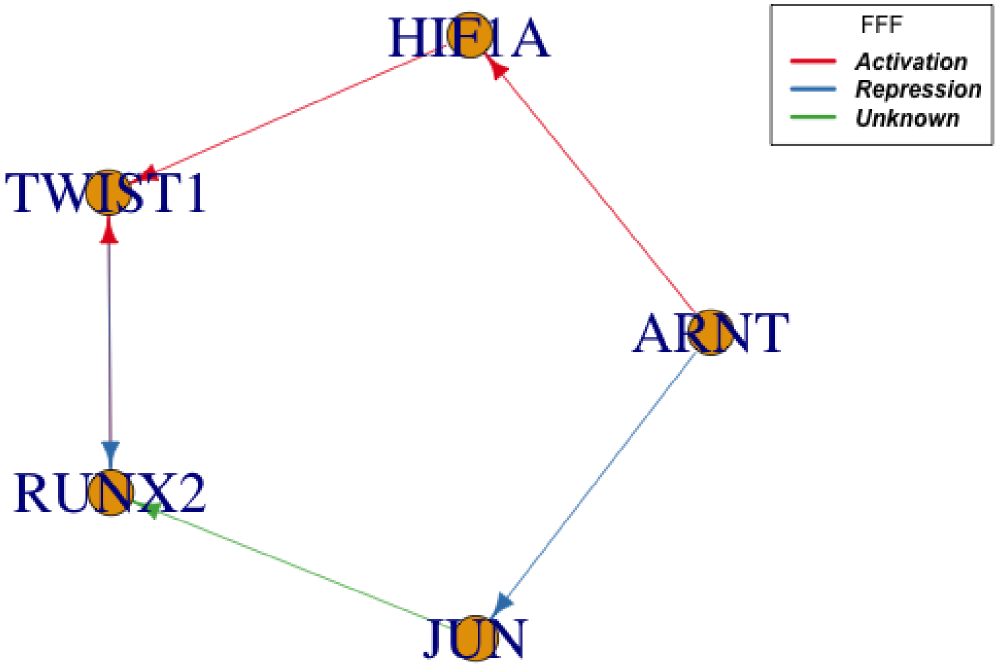

Human TRRUST

SR flip-flop

ATM; SP1

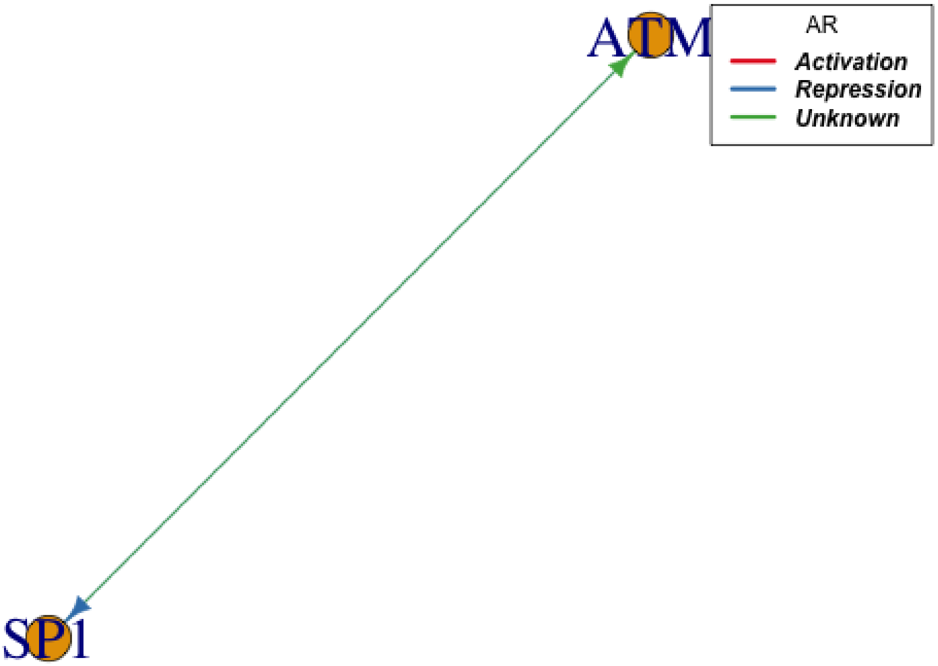

Human TRRUST

Clocked SR flip-flop

HDAC1; EGR1; ESR1; TP73; E2F1

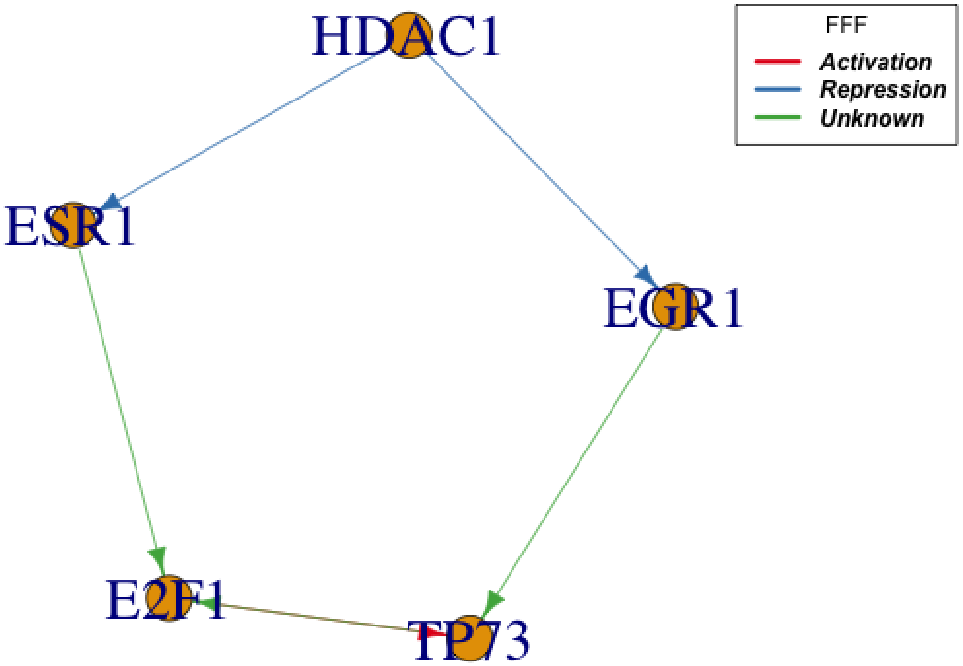

Human TRRUST

SR flip-flop

CIITA; IRF1

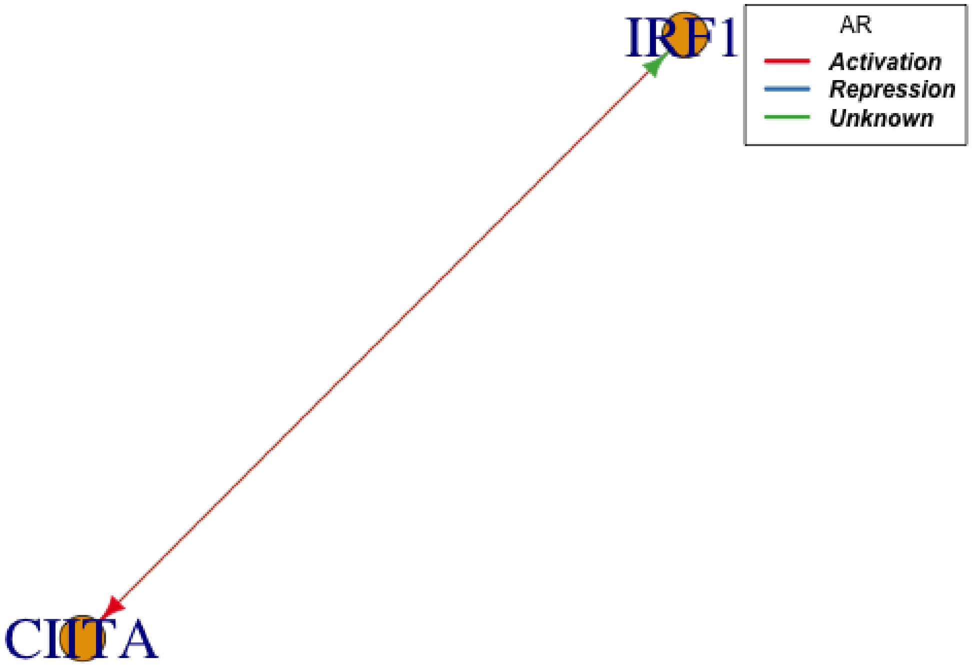

Human TRRUST

SR flip-flop

SOX3; TGIF1

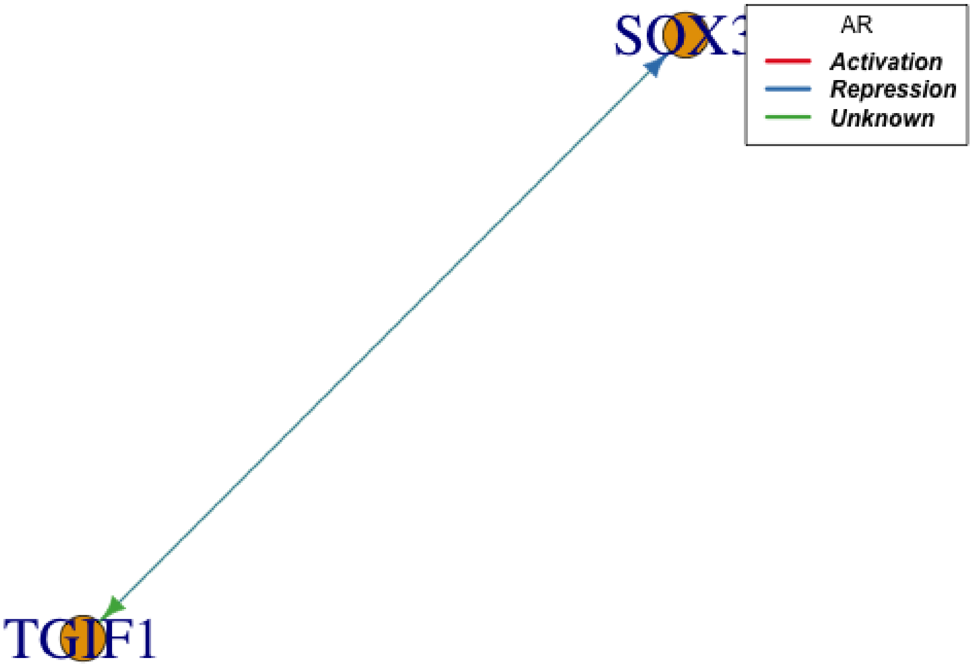

Human TRRUST

Clocked SR flip-flop

RELA; MYC; NCOR2; ID2; MYCN

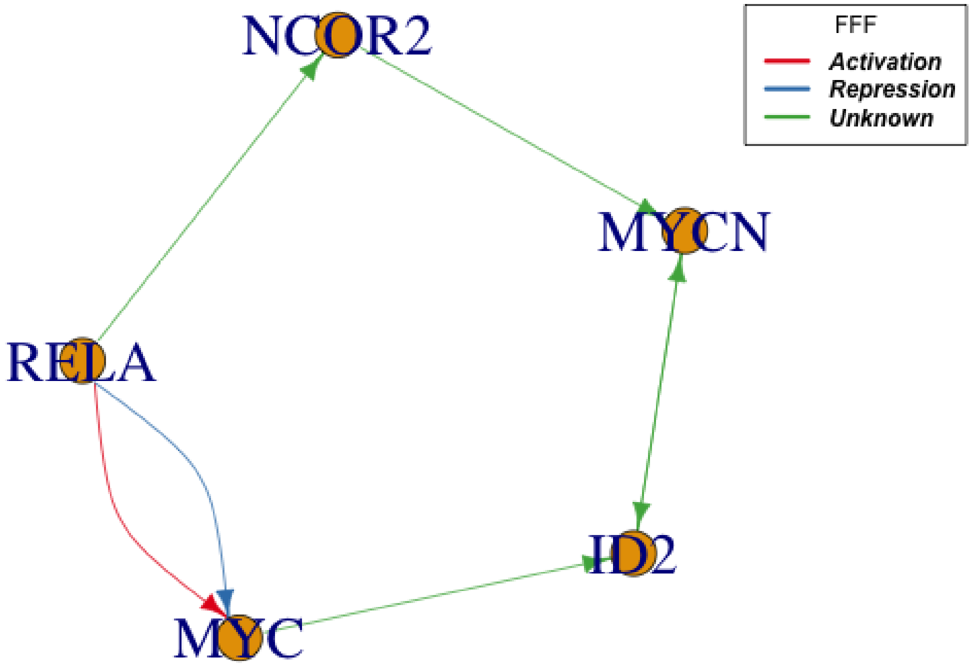

Human TRRUST

Clocked SR flip-flop

CREB1; NF1; LYL1; TP53; NFKB1

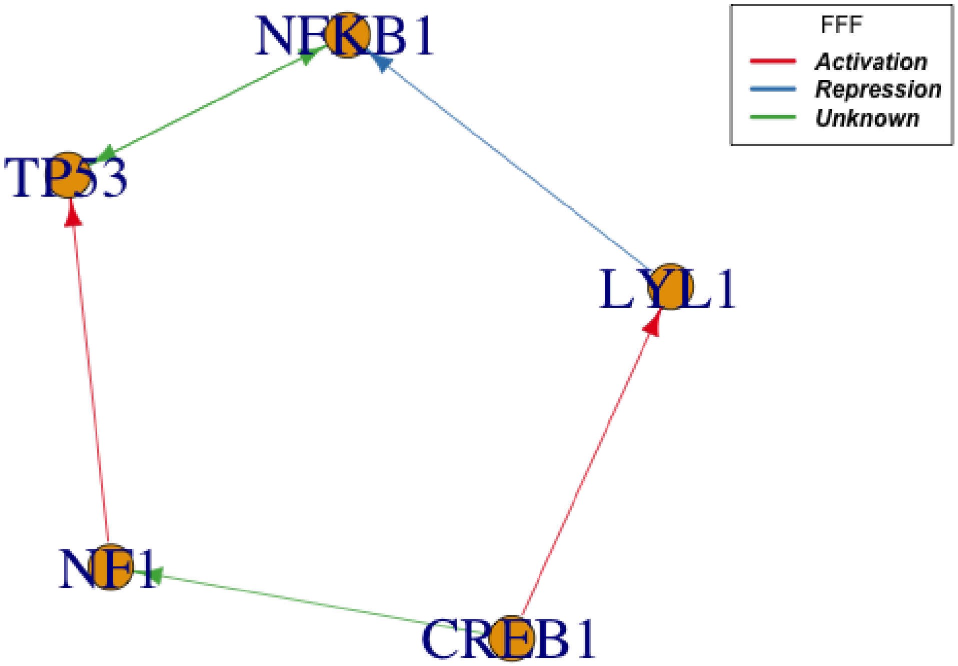

Human TRRUST

SR flip-flop

FLI1; GATA1

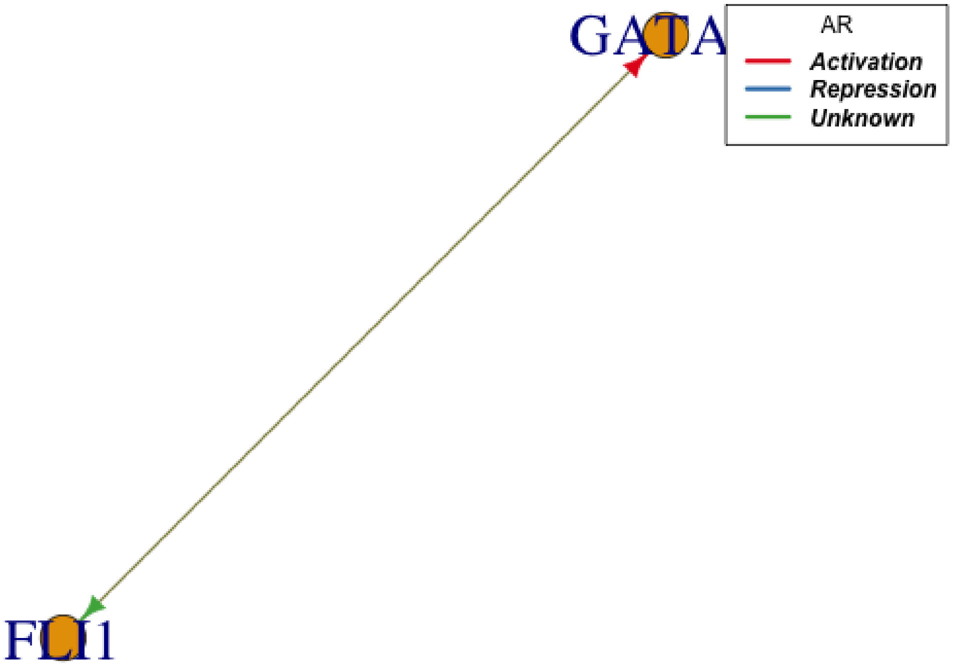

Human TRRUST

Clocked SR flip-flop

RUNX1; FHL2; NF1; NFKB1; TP53

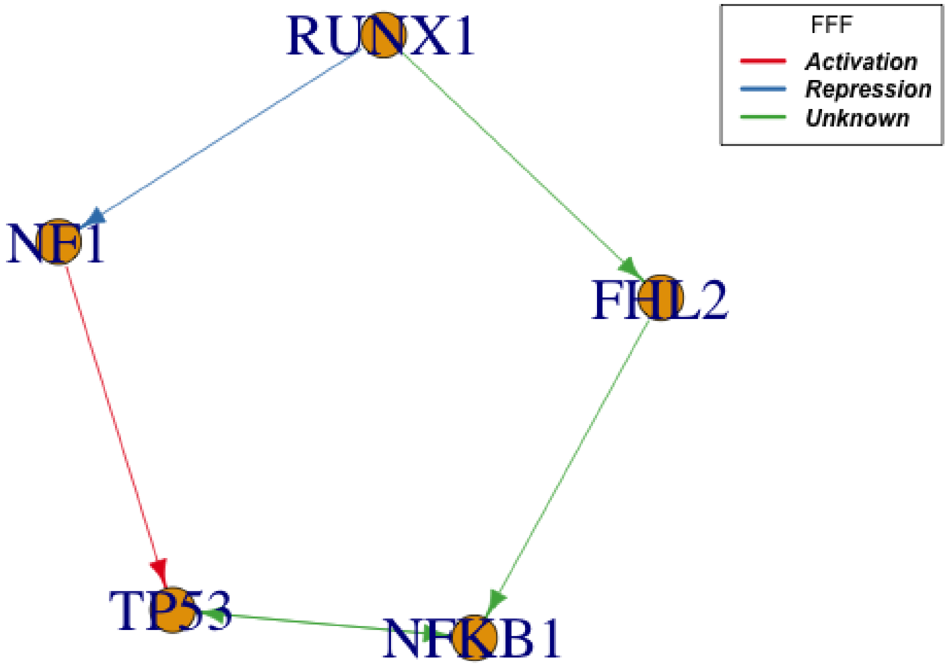

Human TRRUST

Clocked SR flip-flop

NCOA6; FOS; HSF1; PGR; RELA

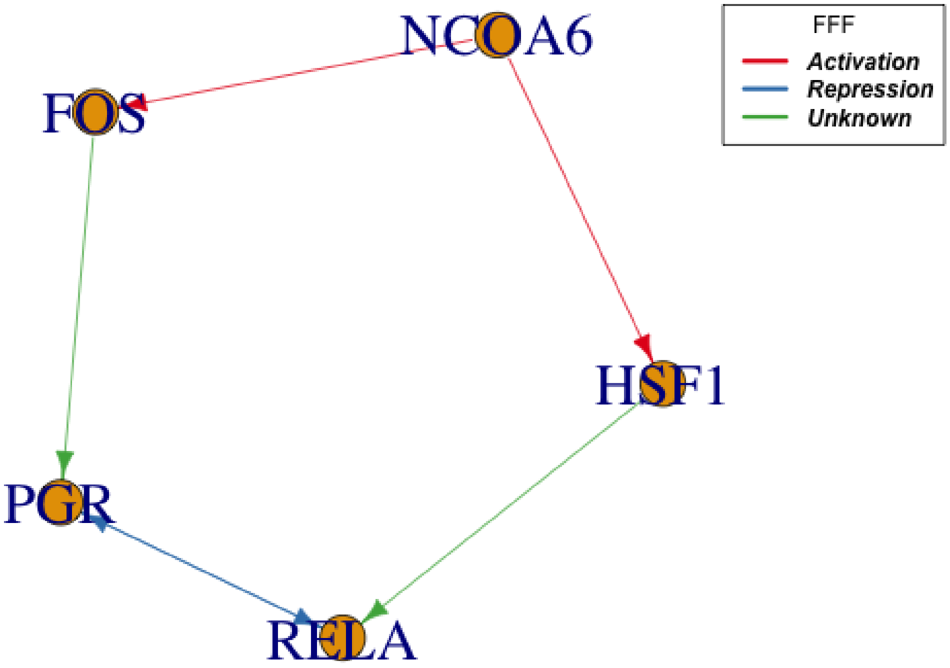

Human TRRUST

SR flip-flop

AR; NKX3-1

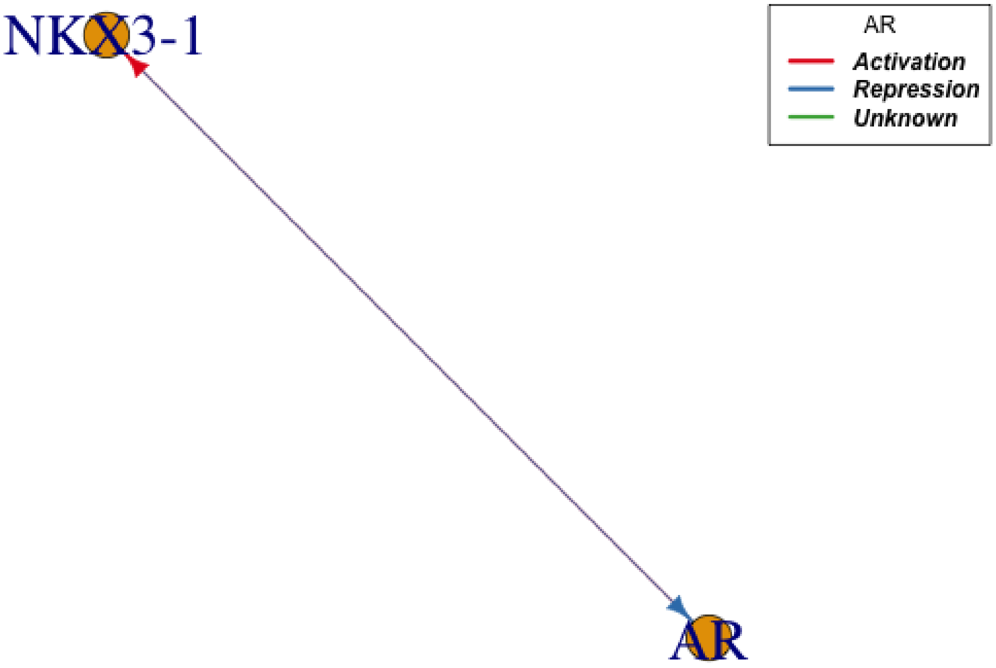

Human TRRUST

SR flip-flop

DBP; HLF

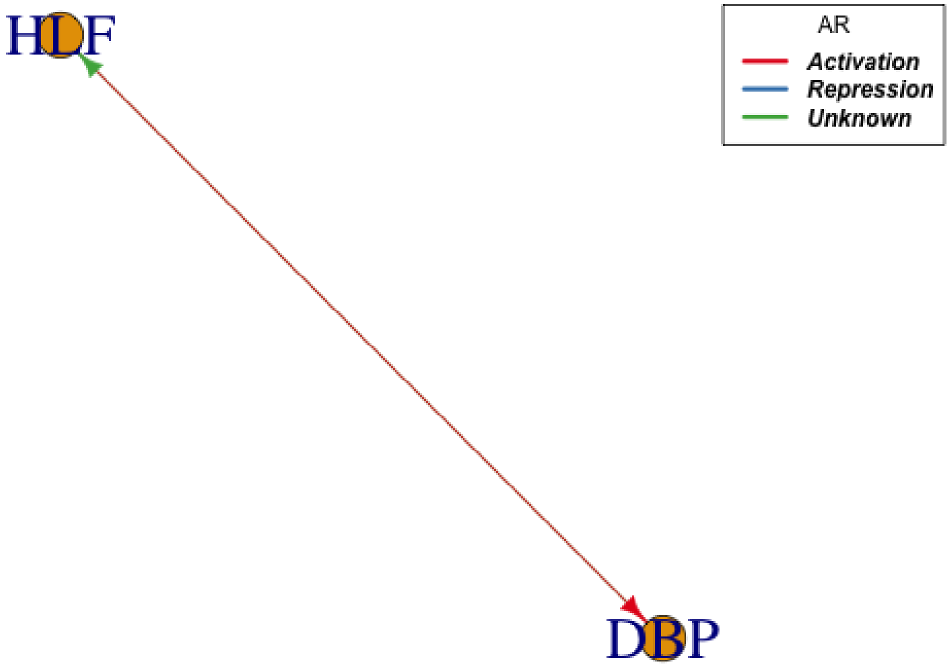

Human TRRUST

SR flip-flop

HDAC2; MYC

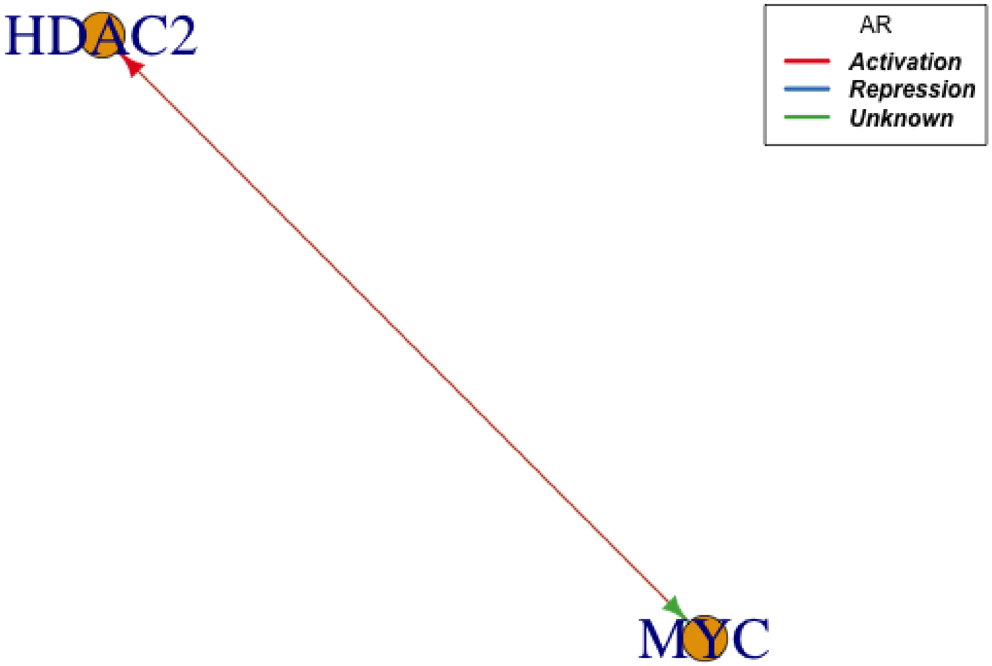

Human TRRUST

Clocked SR flip-flop

CREB1; AR; NF1; EP300; MYC

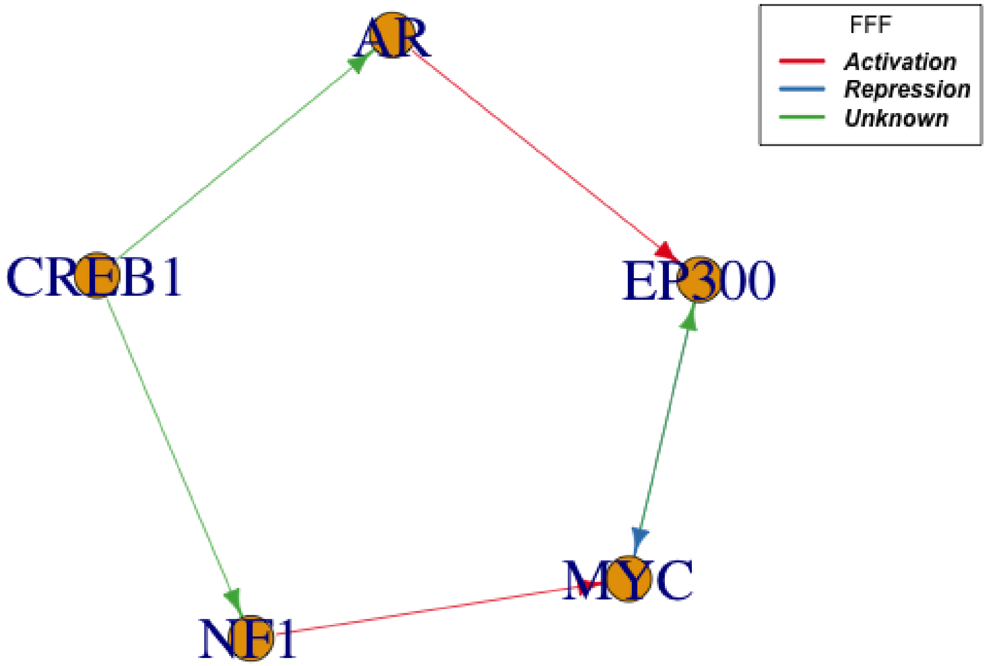

Human TRRUST

SR flip-flop

RELA; TP53

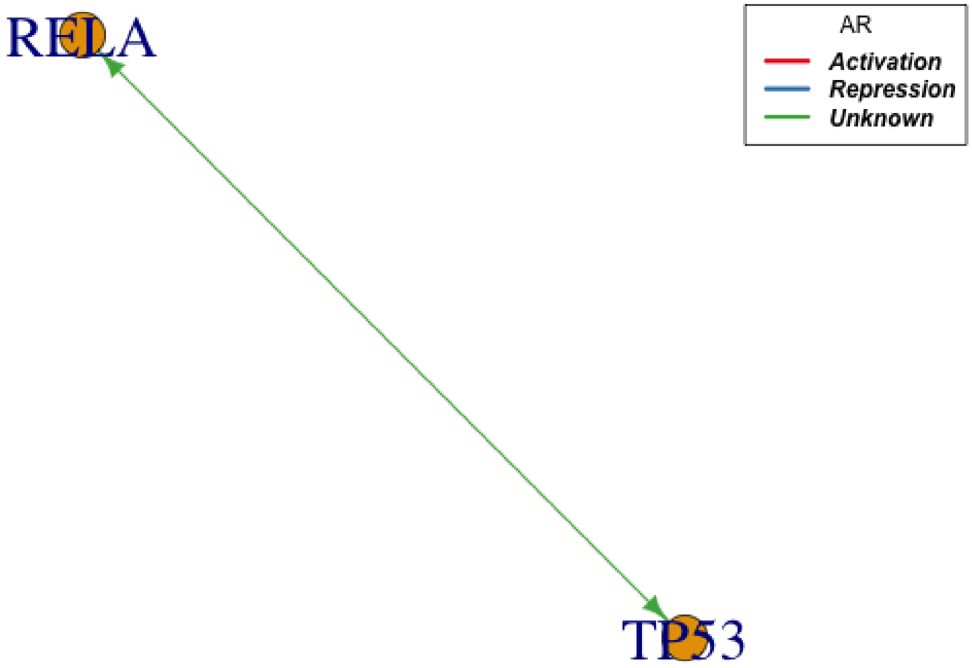

Human TRRUST

Clocked SR flip-flop

SIRT1; PARP1; FOXO3; JUN; AR

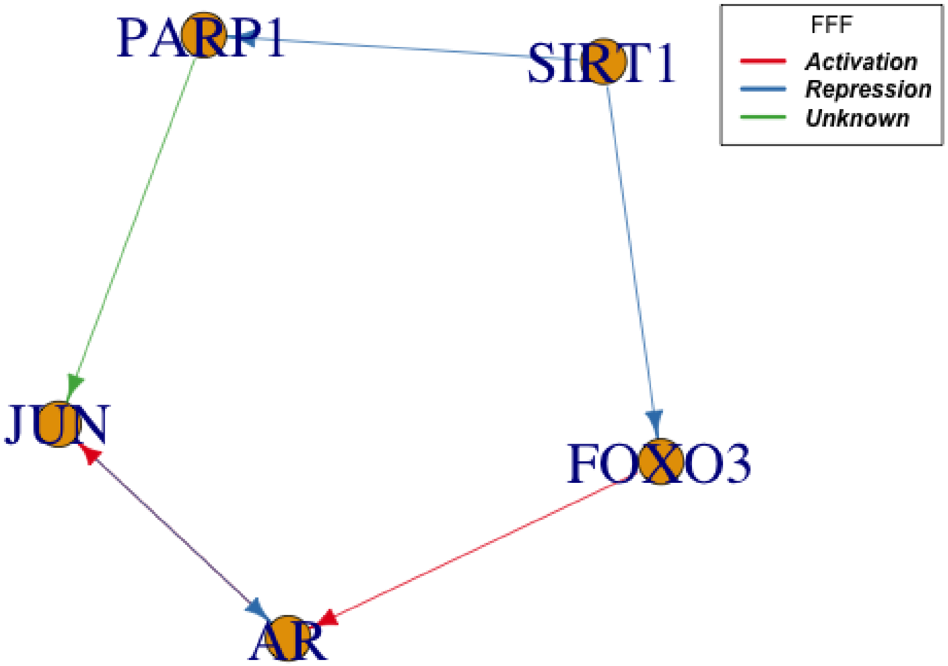

Human TRRUST

SR flip-flop

ESR1; TP53

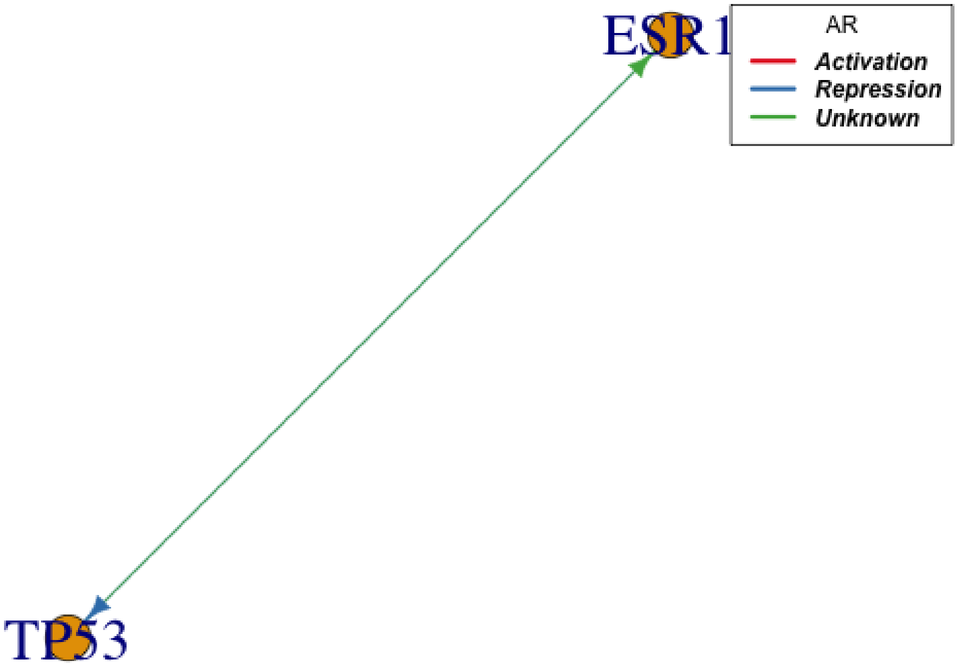

Human TRRUST

Clocked SR flip-flop

SP1; MYCN; SOX3; TP53; STAT3

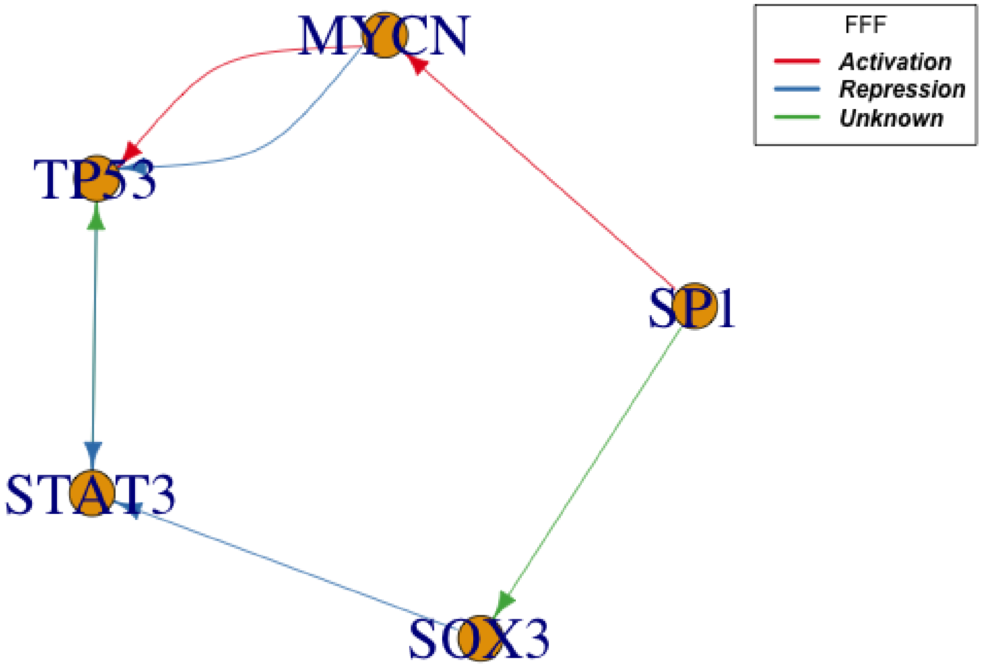

Human TRRUST

Clocked SR flip-flop

STAT1; EGR1; STAT3; EP300; MYC

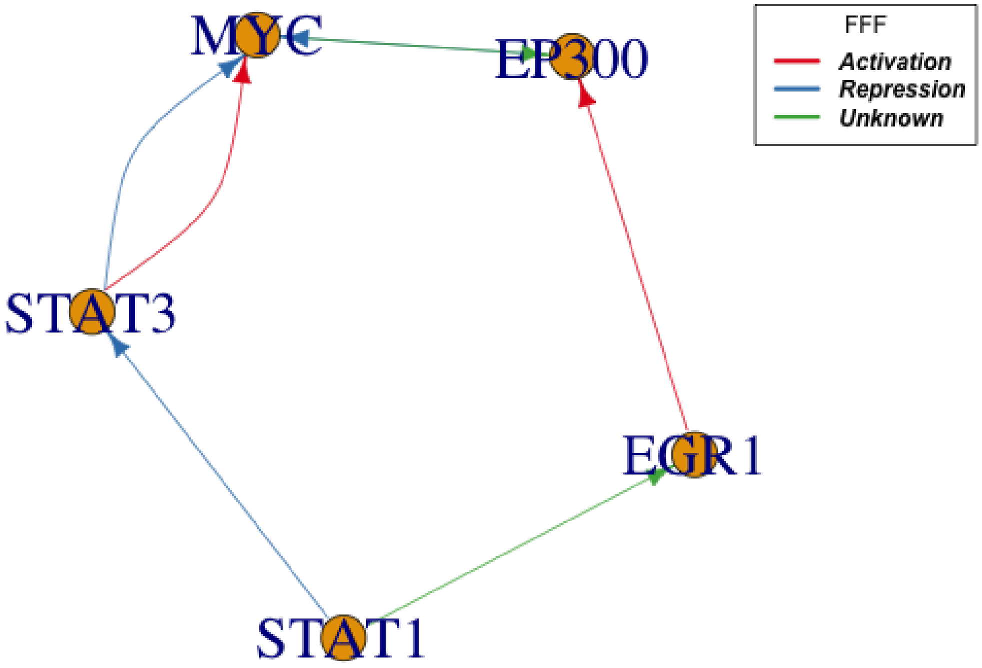

Human TRRUST

SR flip-flop

HDAC2; MYCN

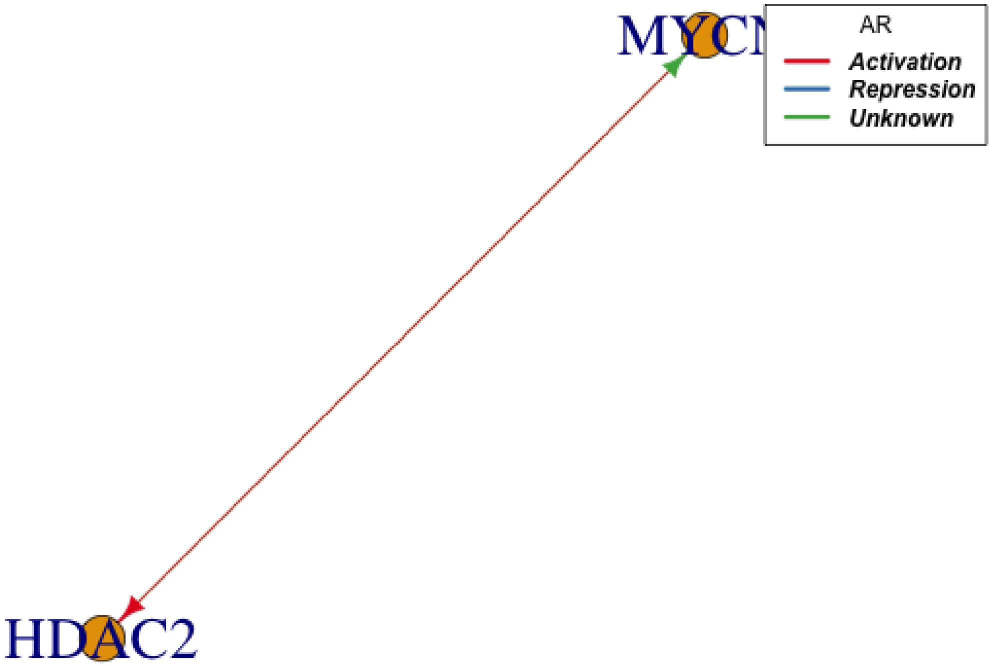

Human TRRUST

SR flip-flop

ETV2; SOX9

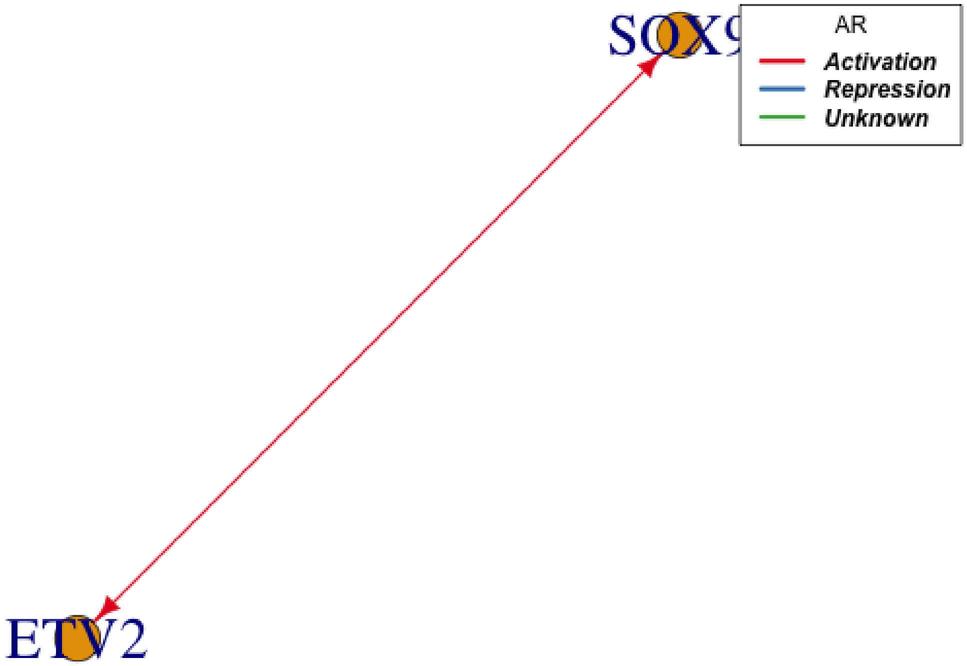

Human TRRUST

SR flip-flop

ESRRG; NR0B1

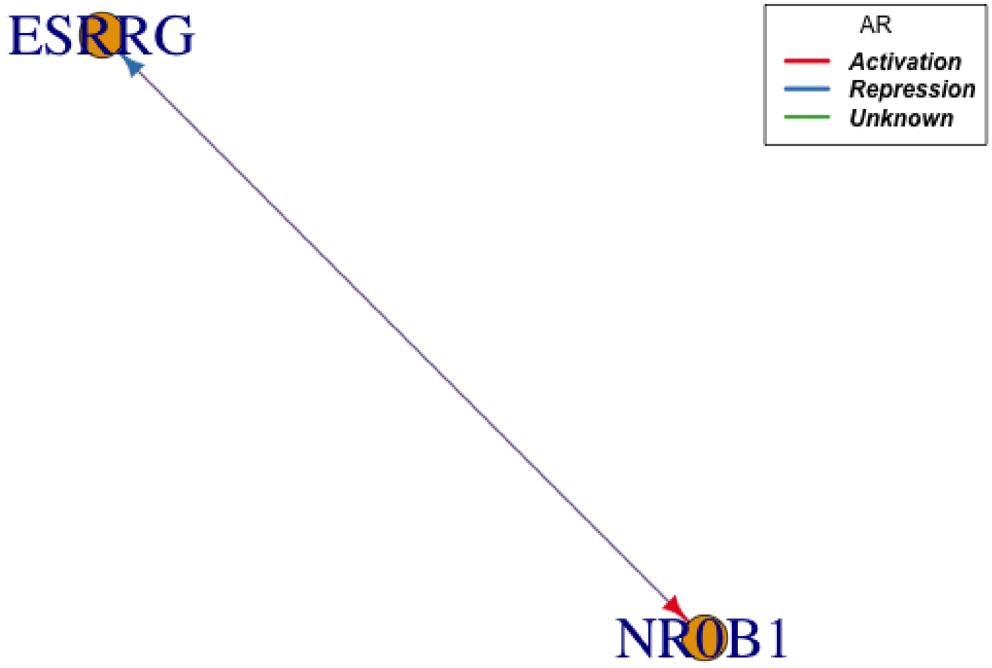

Human TRRUST

Clocked SR flip-flop

ABL1; JUN; FOXO3; MYC; FOXM1

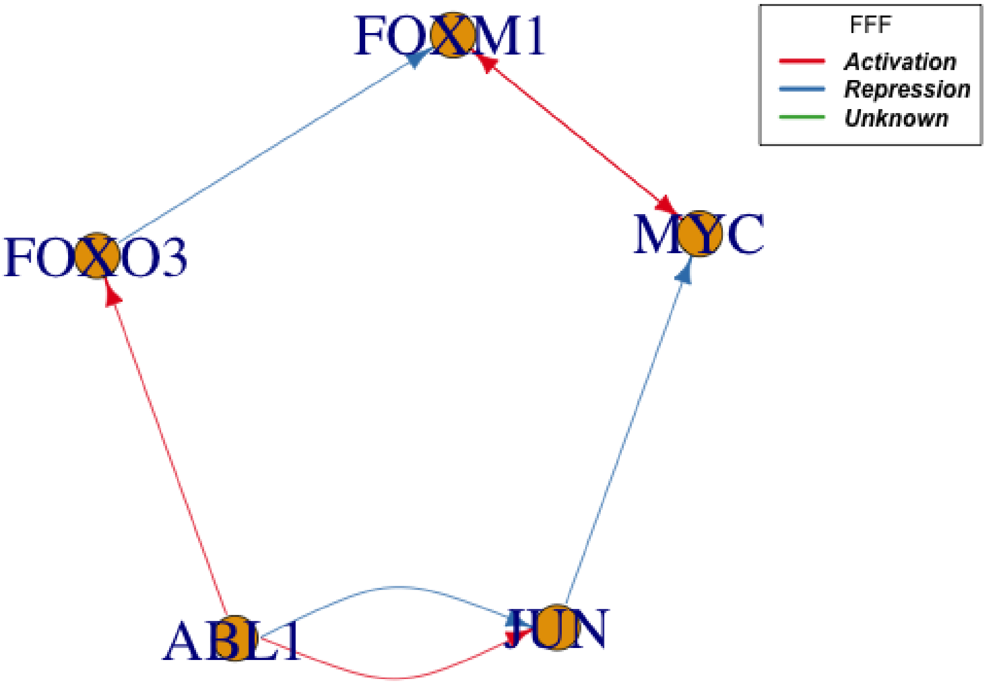

Human TRRUST

Clocked SR flip-flop

EZH2; APC; TWIST1; MYC; MYB

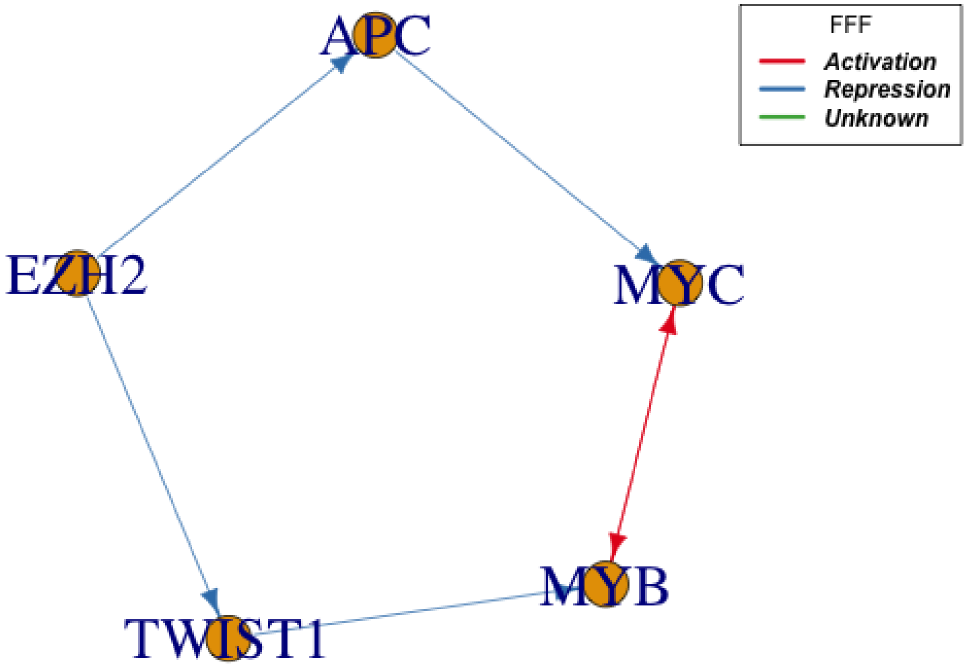

Human TRRUST

Clocked SR flip-flop

EP300; DNMT1; PARP1; RB1; E2F1

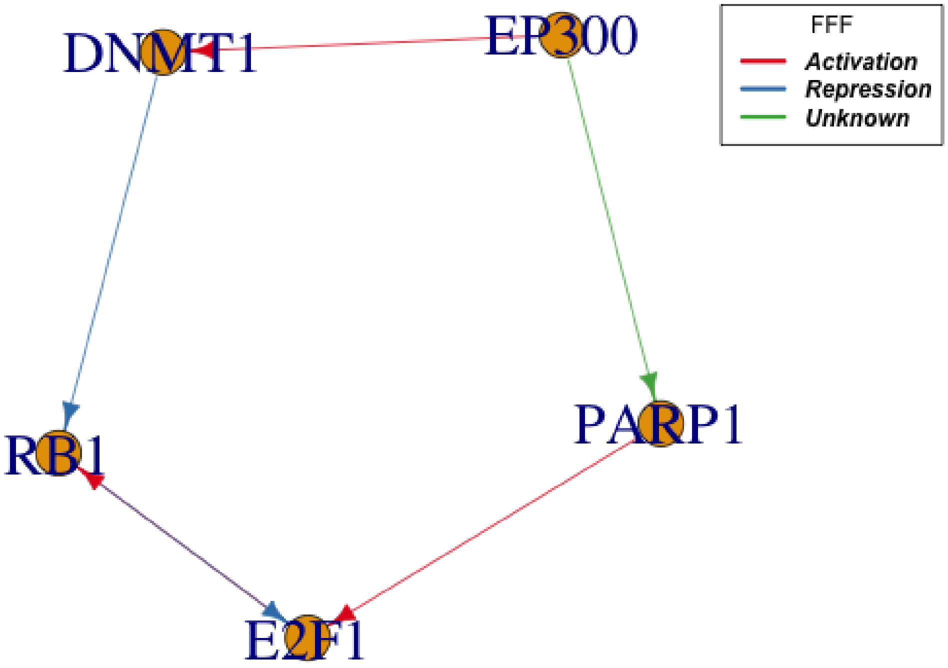

Human TRRUST

Clocked SR flip-flop

ZNF382; JUN; STAT3; RUNX2; TWIST1

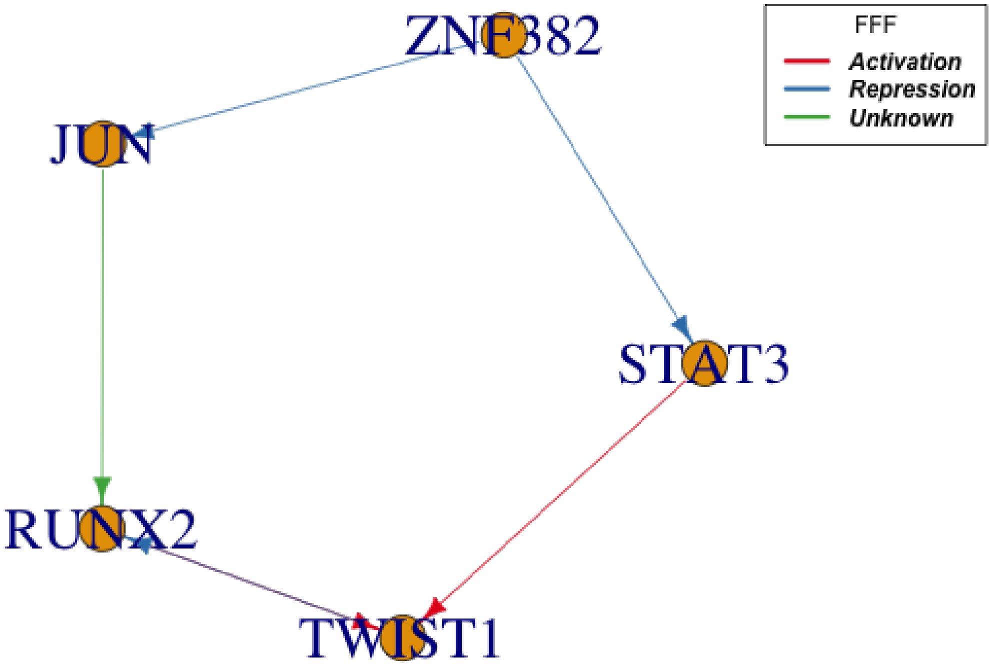

Human TRRUST

Clocked SR flip-flop

RELA; TWIST1; PPARA; ESR1; STAT5A

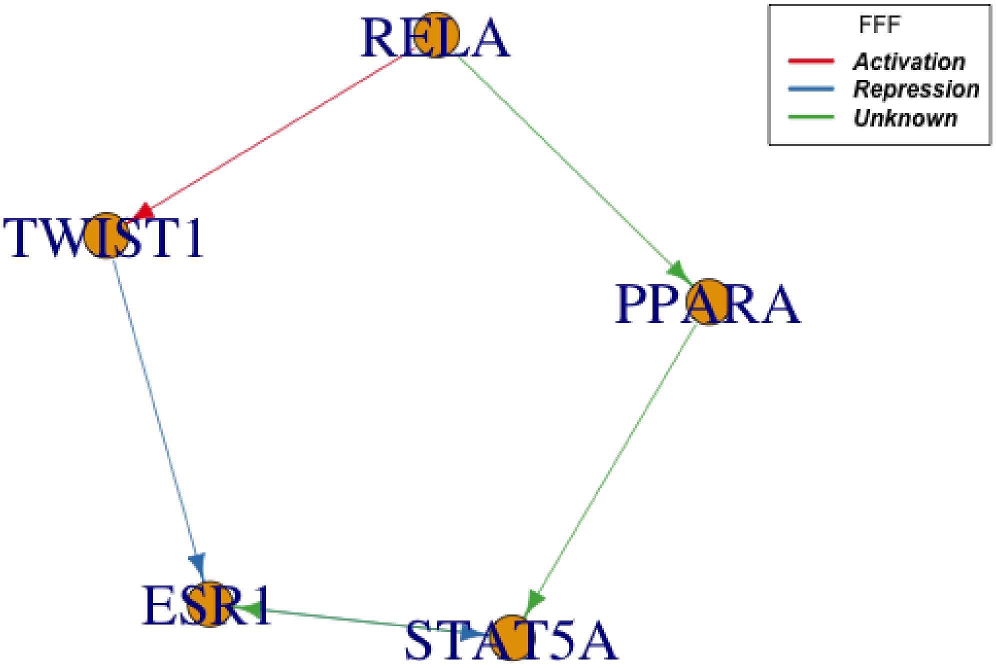

Human TRRUST

SR flip-flop

ELF4; TP53

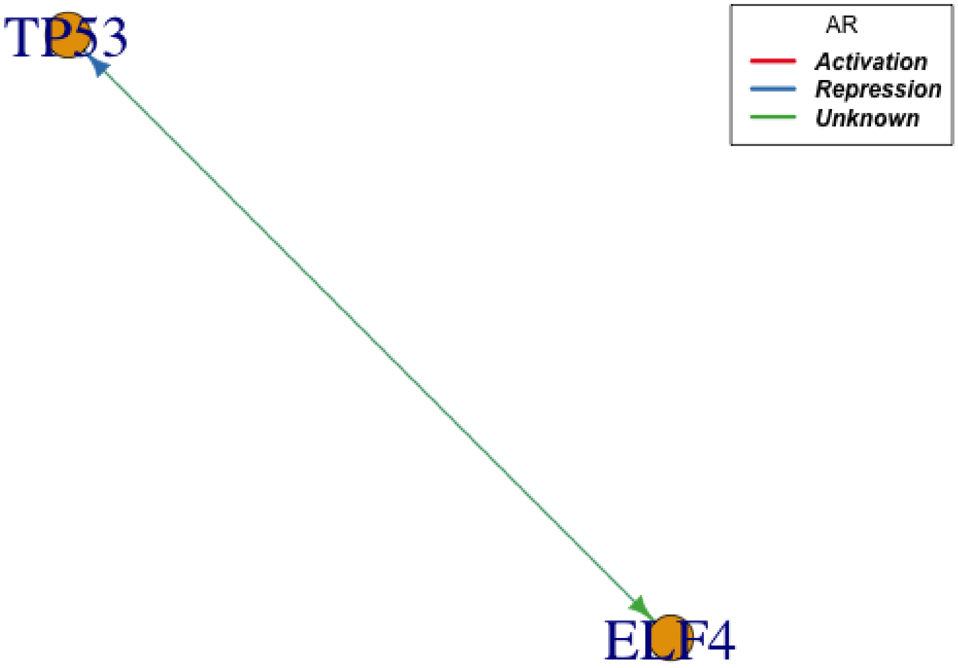

Human TRRUST

Clocked SR flip-flop

RUNX1; KLF4; FHL2; TP53; RELA

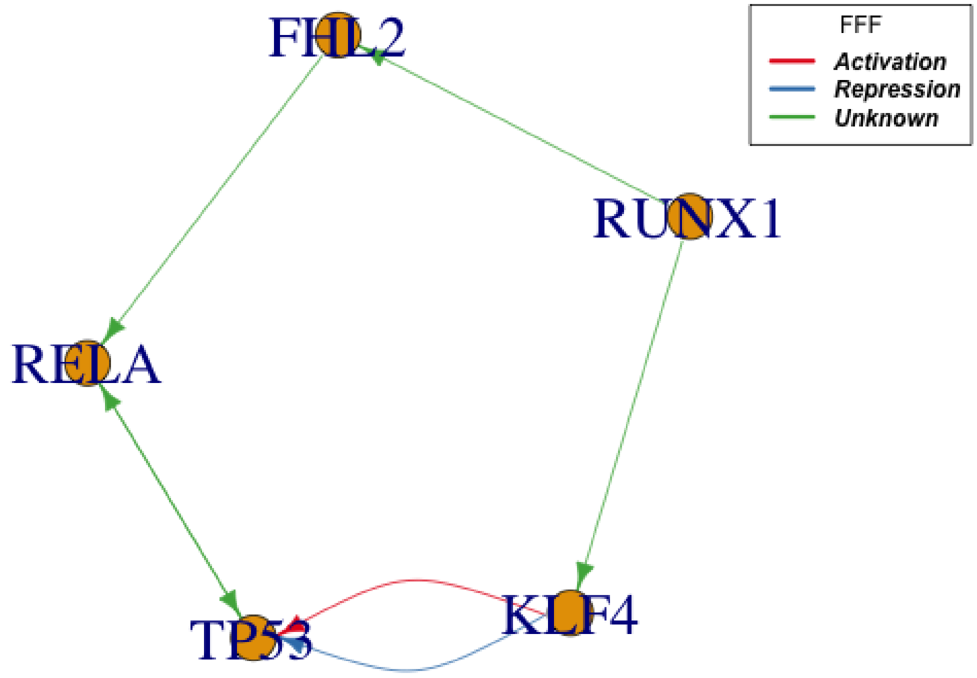

Human TRRUST

SR flip-flop

BRCA1; E2F6

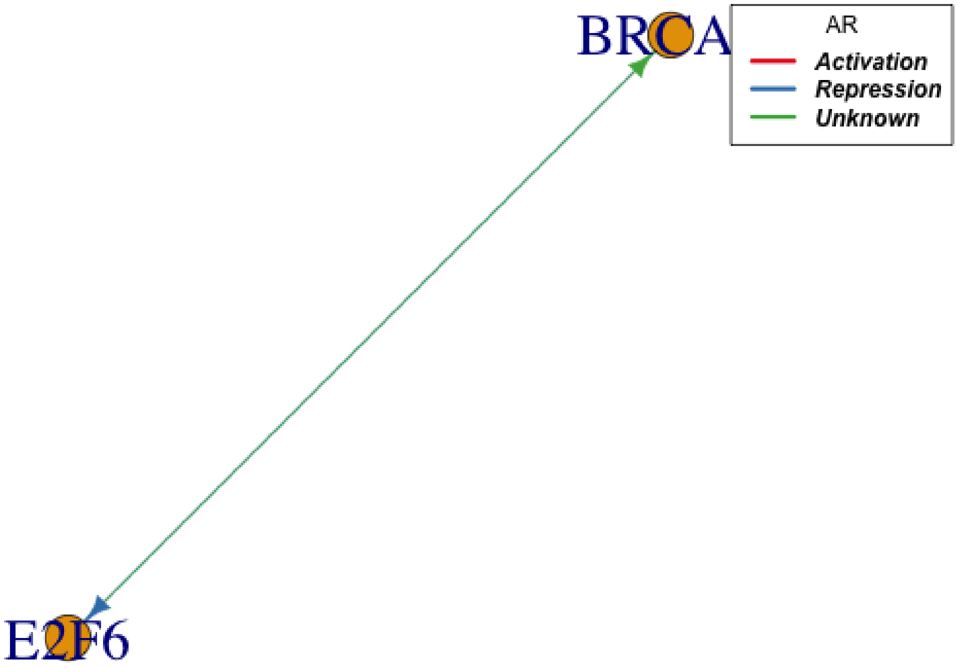

Human TRRUST

SR flip-flop

ESR1; PGR

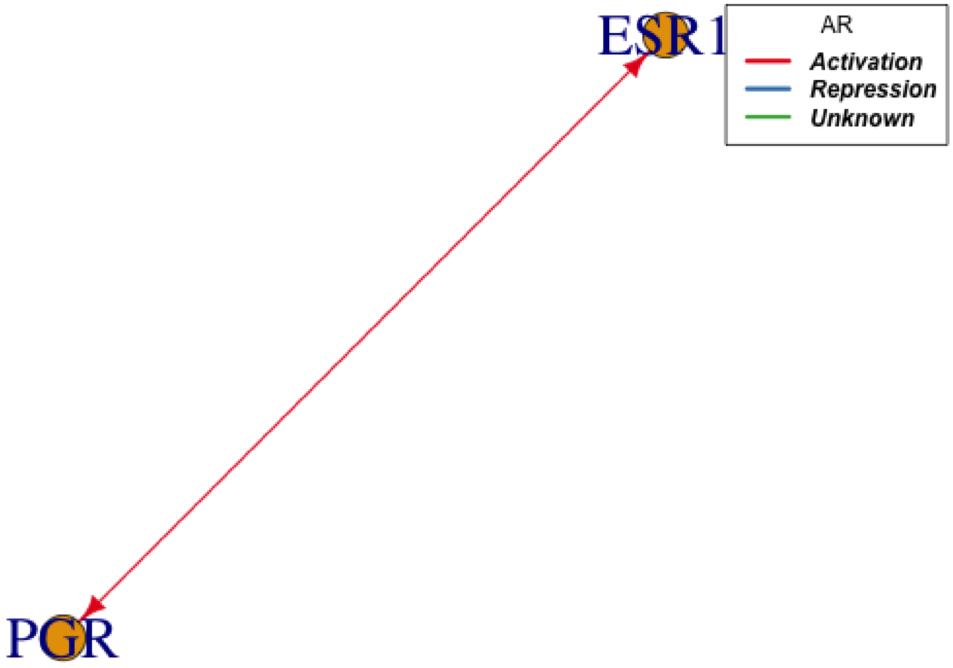

Human TRRUST

SR flip-flop

NFKB1; TP53

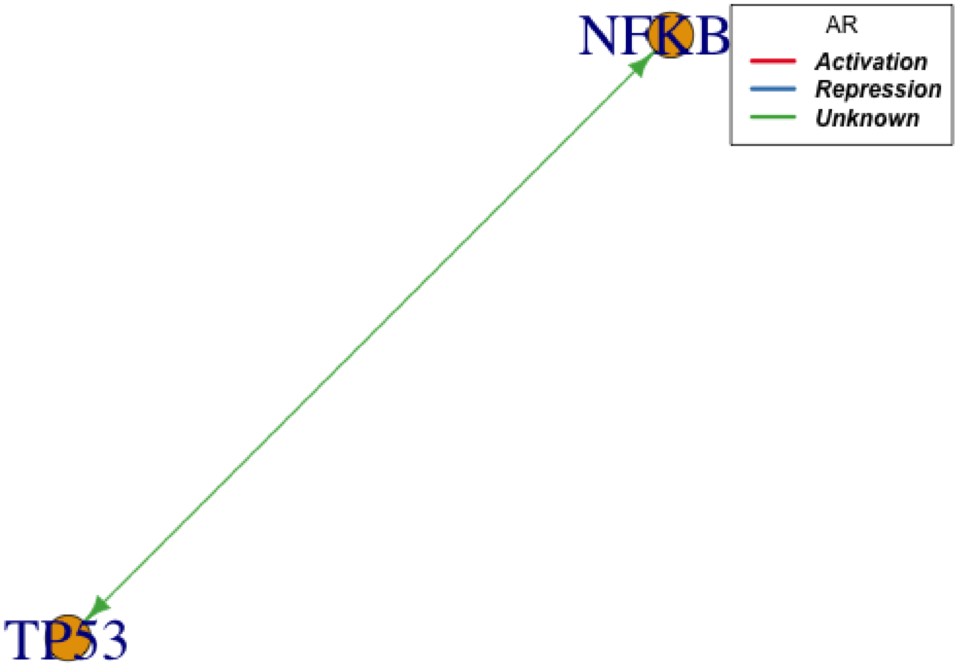

Human TRRUST

Clocked SR flip-flop

NFKB1; MYC; NCOR2; ID2; MYCN

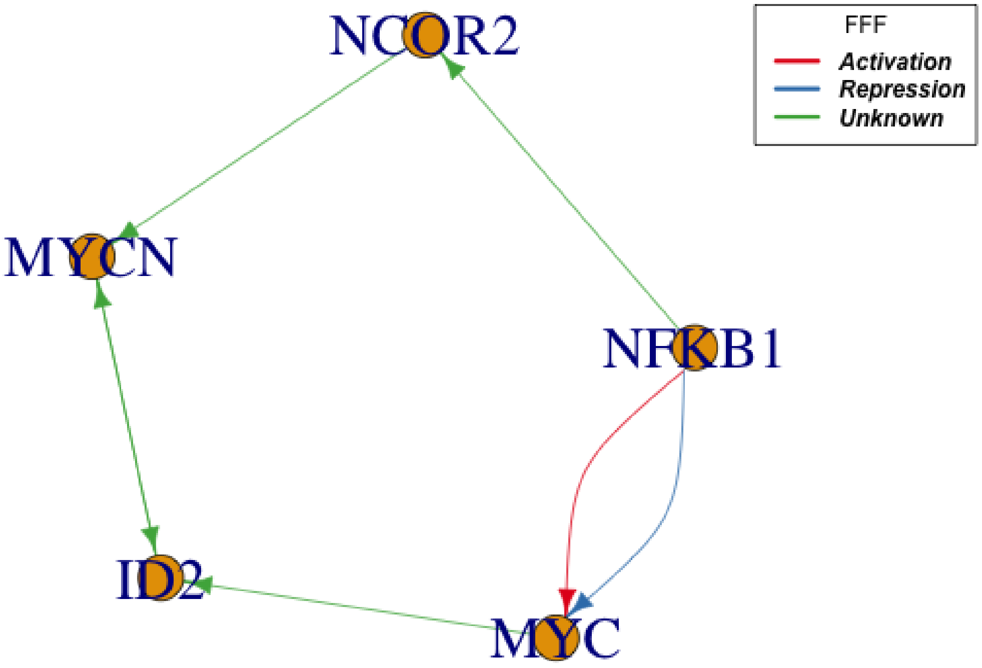

Human TRRUST

Clocked SR flip-flop

HIF1A; TWIST1; PPARA; ESR1; STAT5A

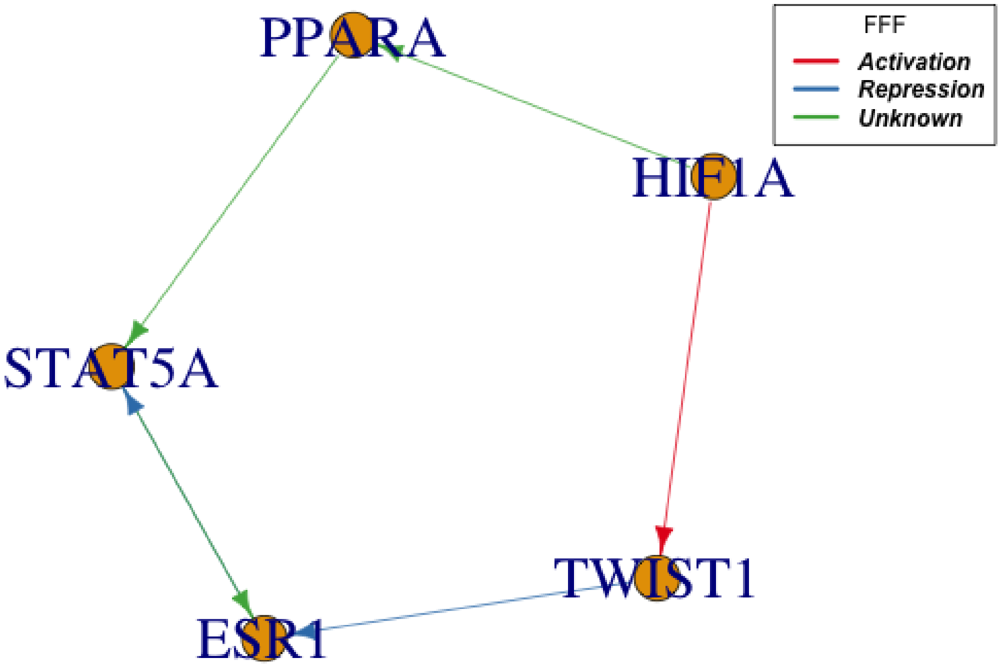

Arabidopsis Thaliana

SR flip-flop

AP1; AG

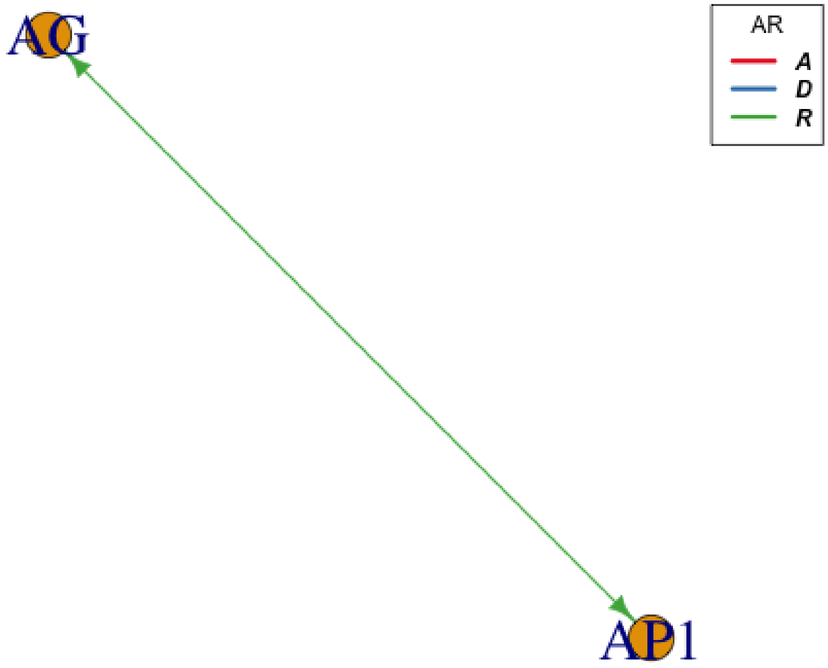

Arabidopsis Thaliana

SR flip-flop

AS2; PHB

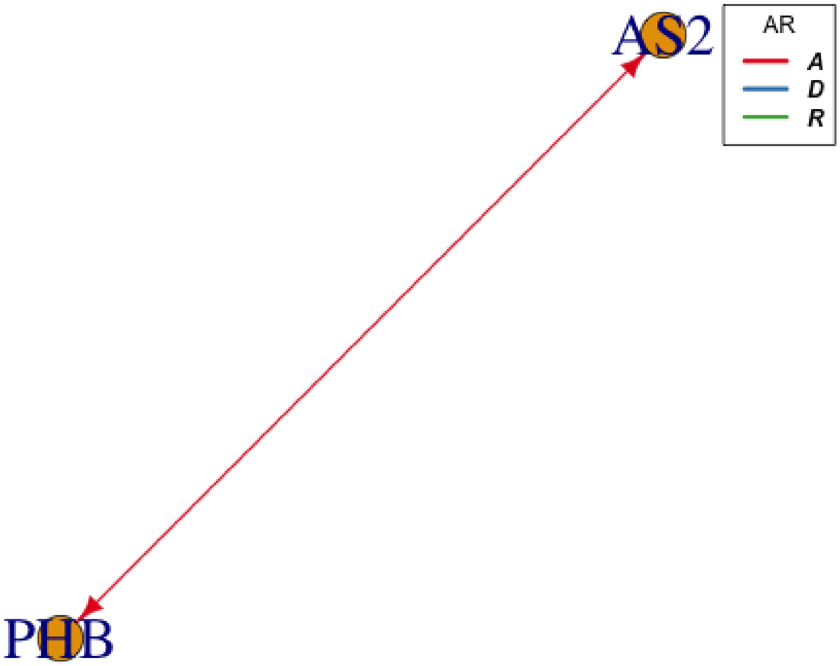

Arabidopsis Thaliana

SR flip-flop

ABI3; FUS3

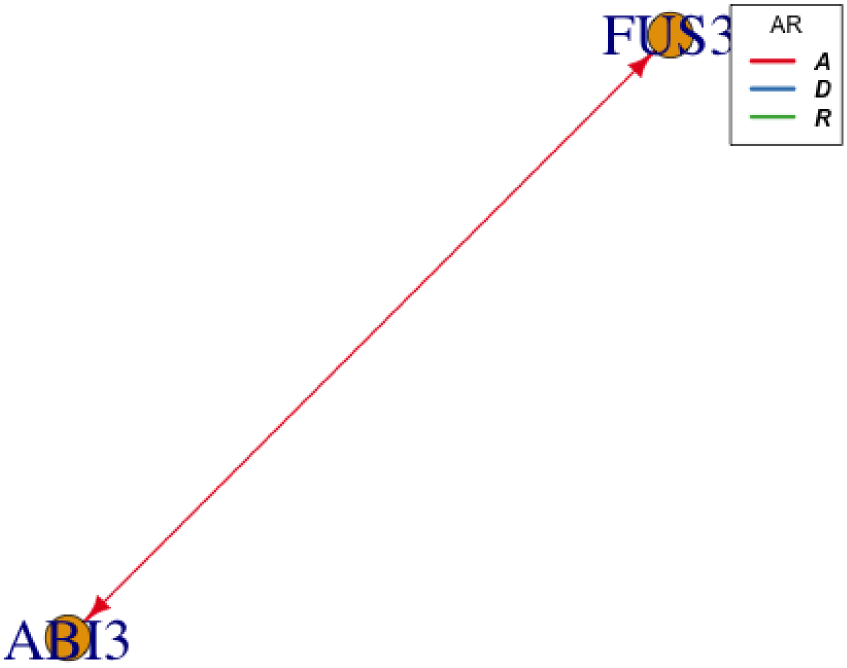

Arabidopsis Thaliana

SR flip-flop

LHY; PCL1

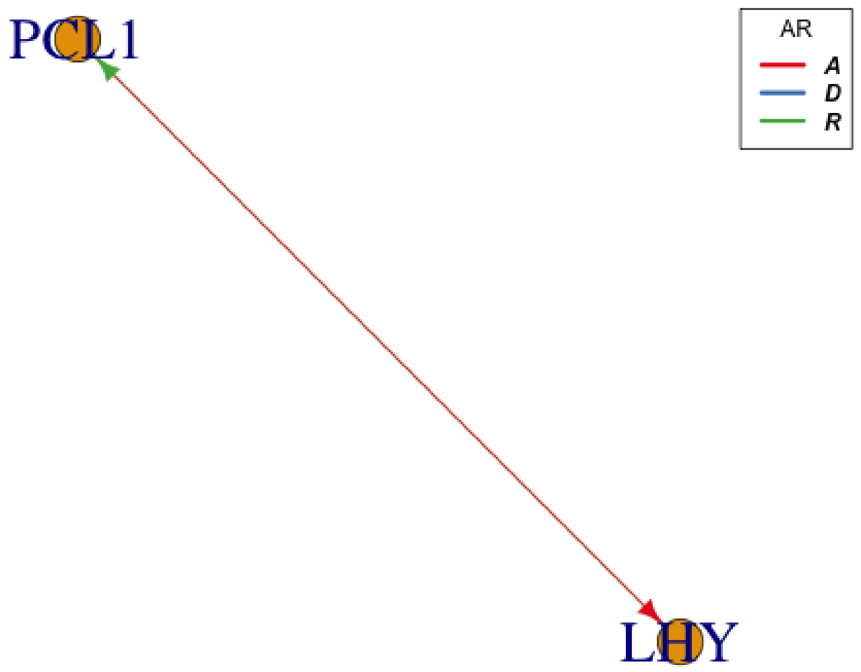

Arabidopsis Thaliana

SR flip-flop

WUS; AG

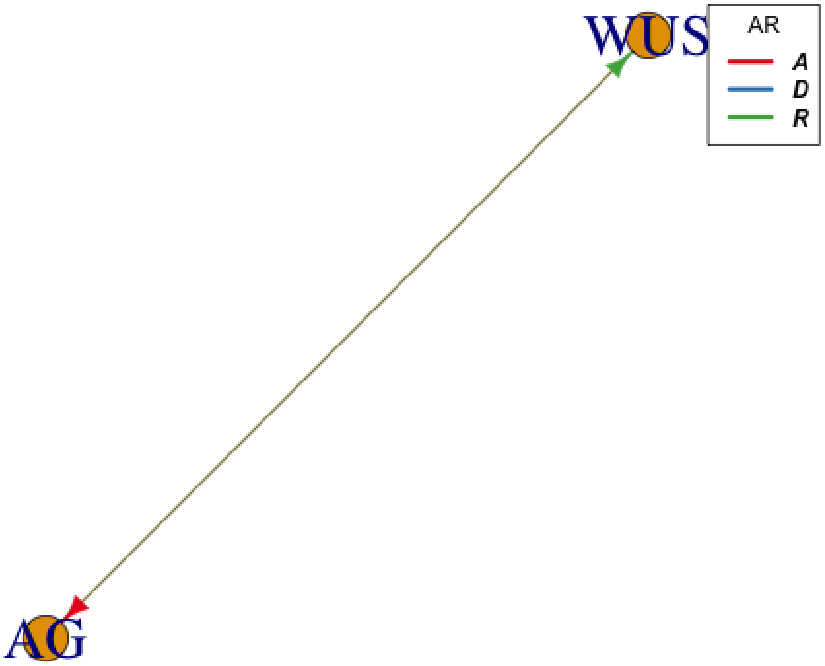

Human TRRUST2

Clocked SR flip-flop

MEN1; BRCA1; JUND; STAT3; BCL6

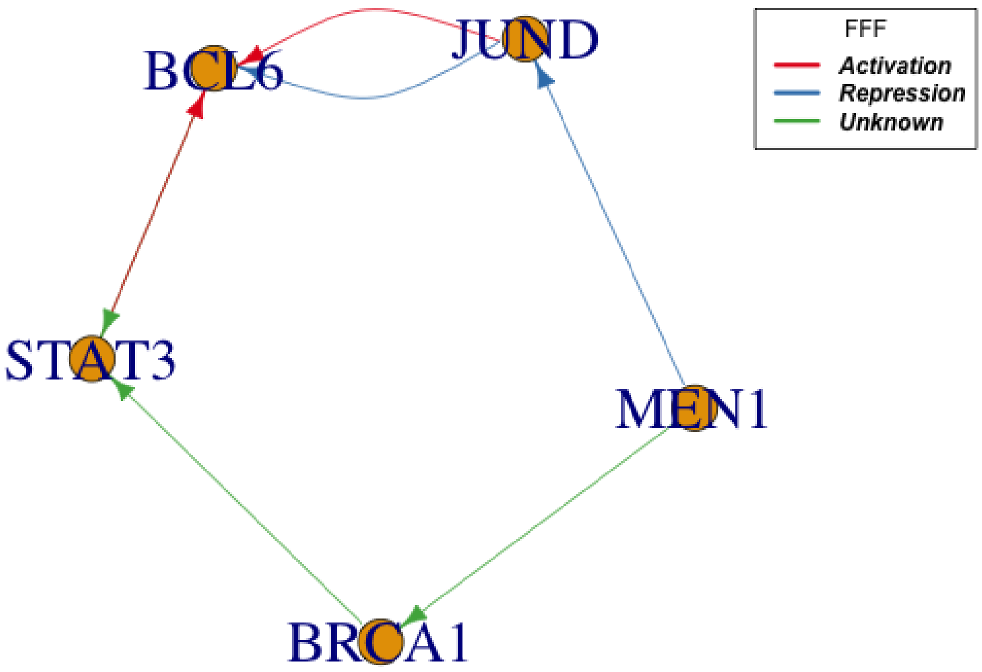

Human TRRUST2

Clocked SR flip-flop

E2F1; BRCA1; PPARG; STAT1; CIITA

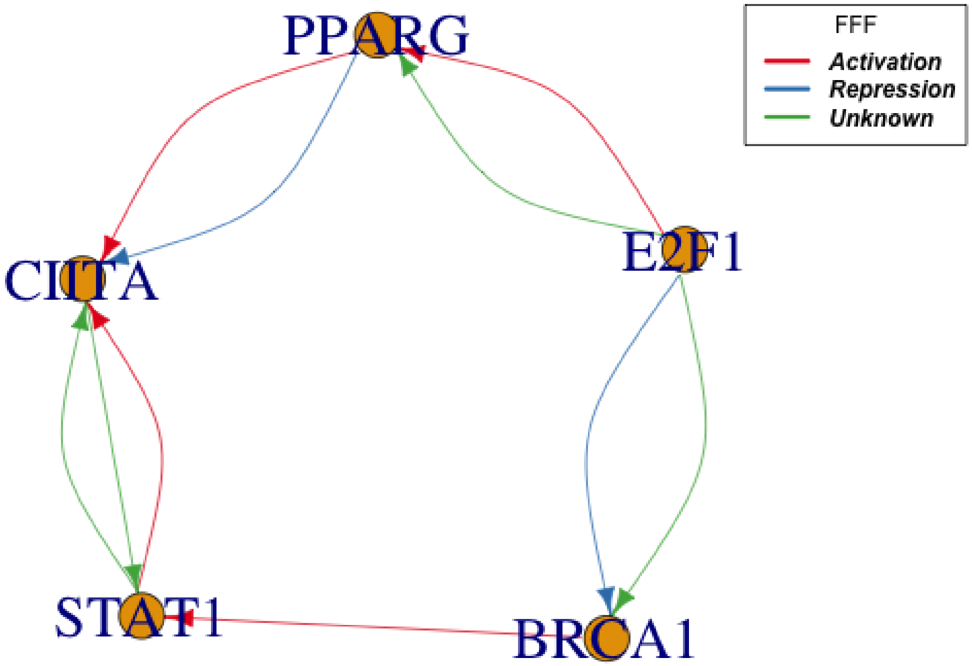

Human TRRUST2

Clocked SR flip-flop

SIRT1; HSF1; HIF1A; JUN; AR

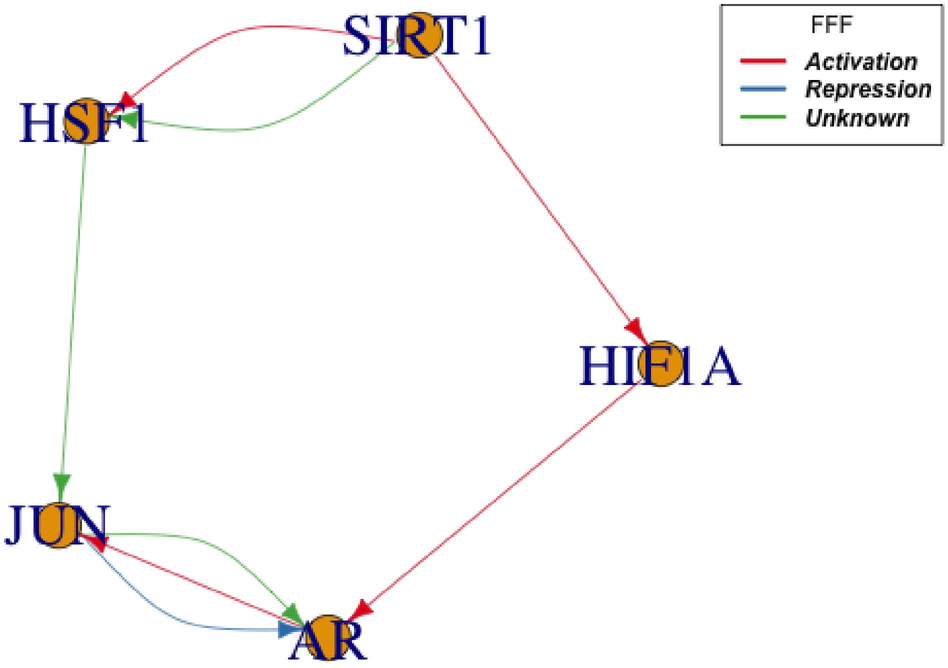

Human TRRUST2

SR flip-flop

WT1; ETS1

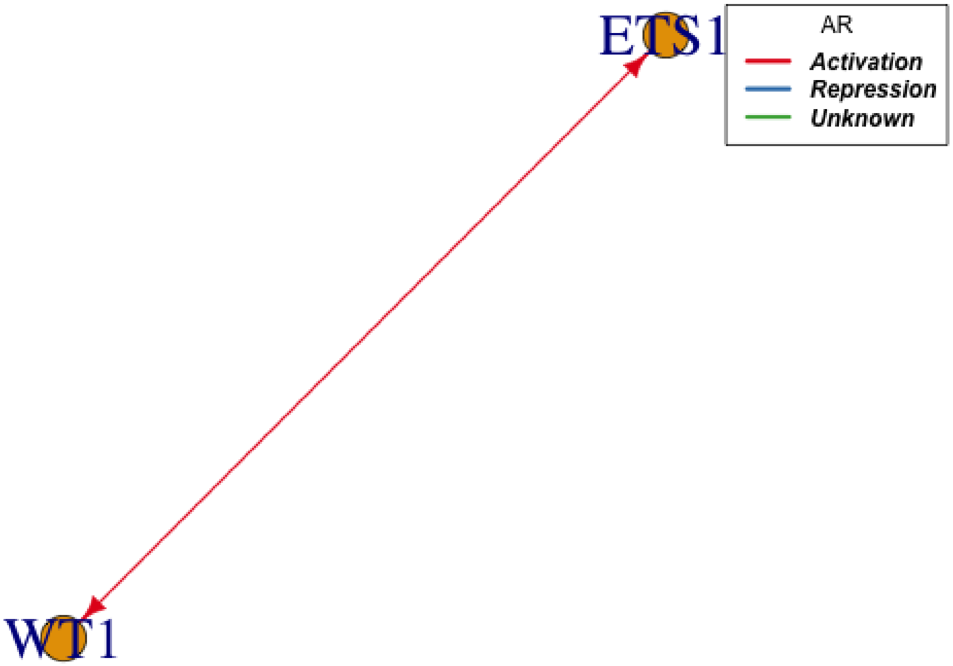

Human TRRUST2

SR flip-flop

NR0B1; NR5A1

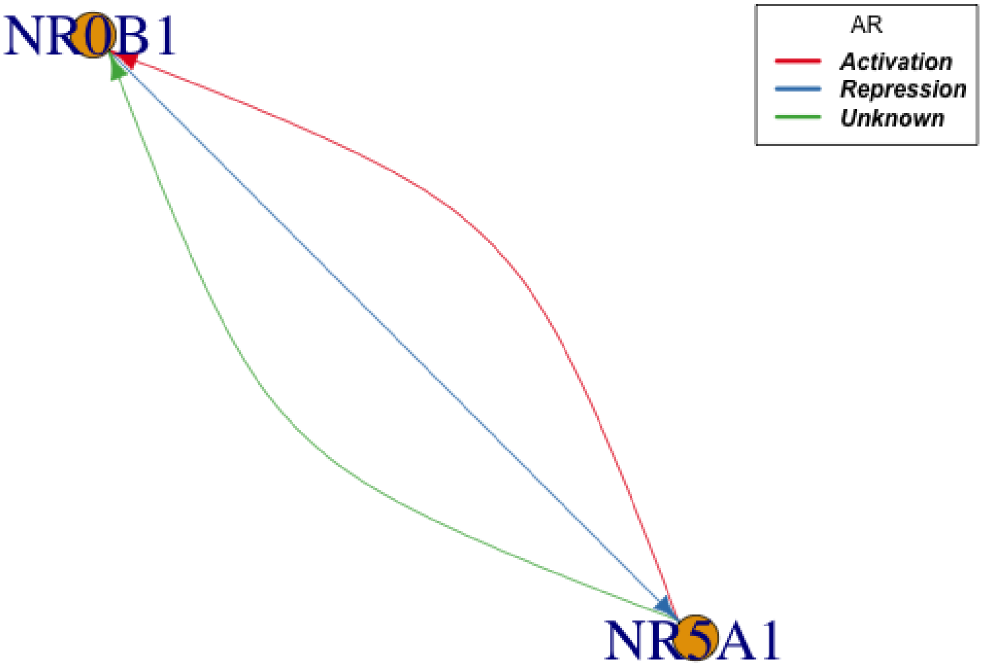

Human TRRUST2

Clocked SR flip-flop

PITX1; JUN; HES1; TP53; E2F1

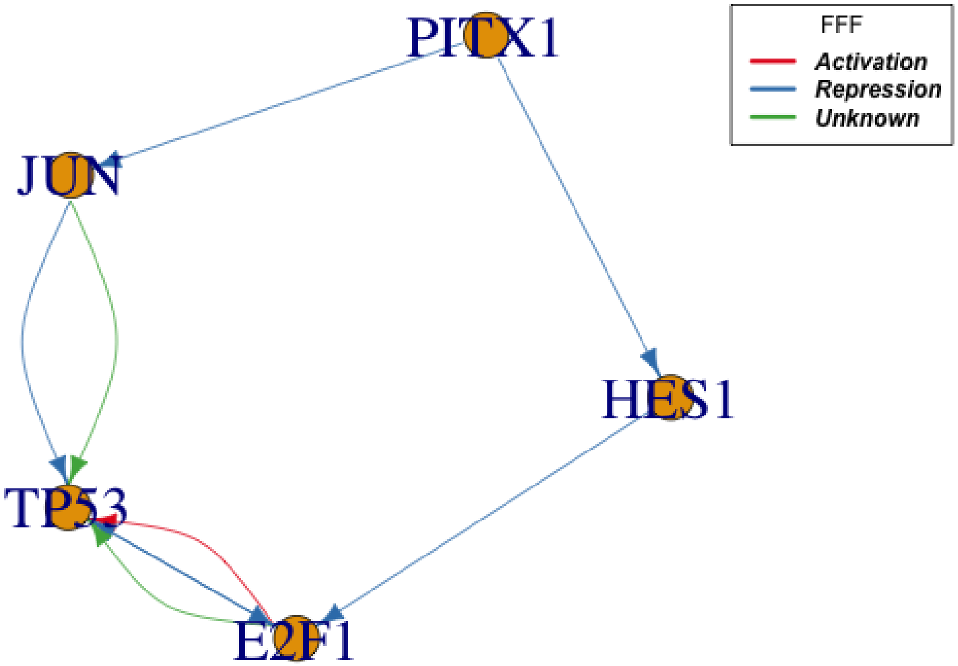

Human TRRUST2

Clocked SR flip-flop

SP1; BRCA1; HDAC4; IRF7; ATF4

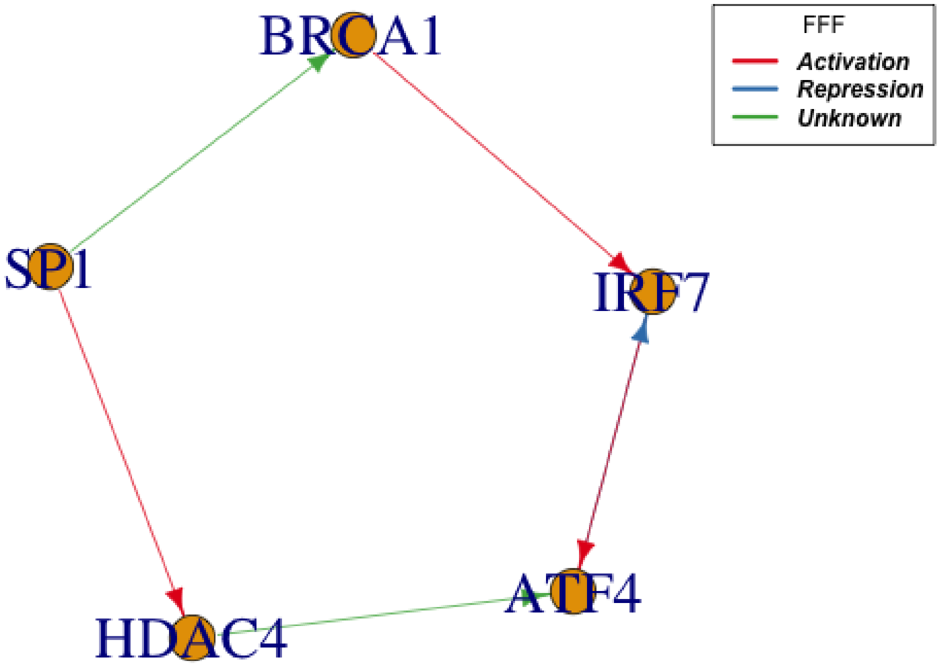

Human TRRUST2

Clocked SR flip-flop

EZH2; TWIST1; PAX8; MYB; WT1

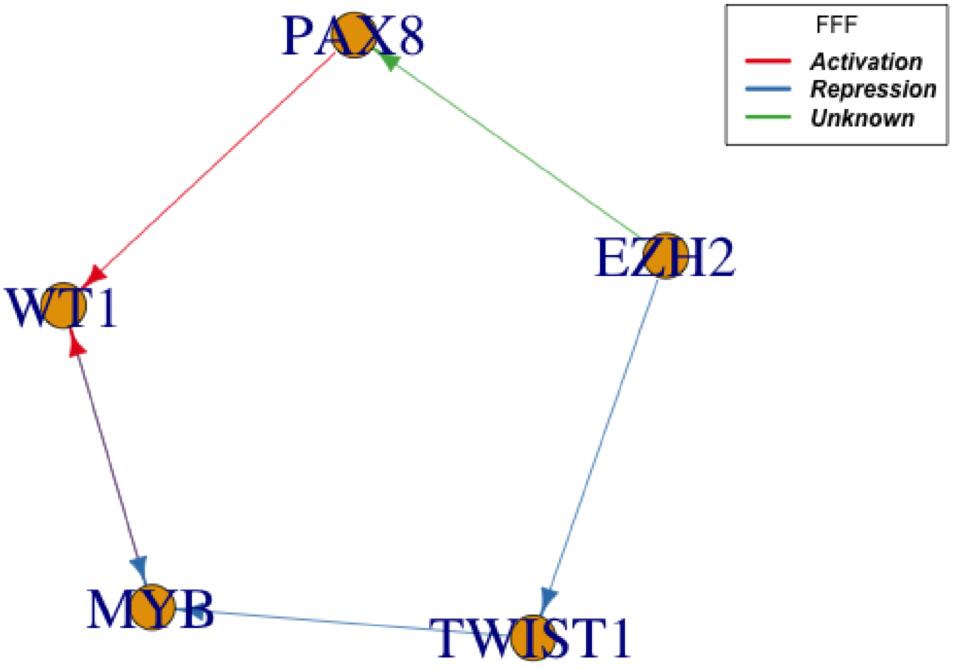

Human TRRUST2

Clocked SR flip-flop

HDAC1; TP53; SP1; TCF7L2; GATA3

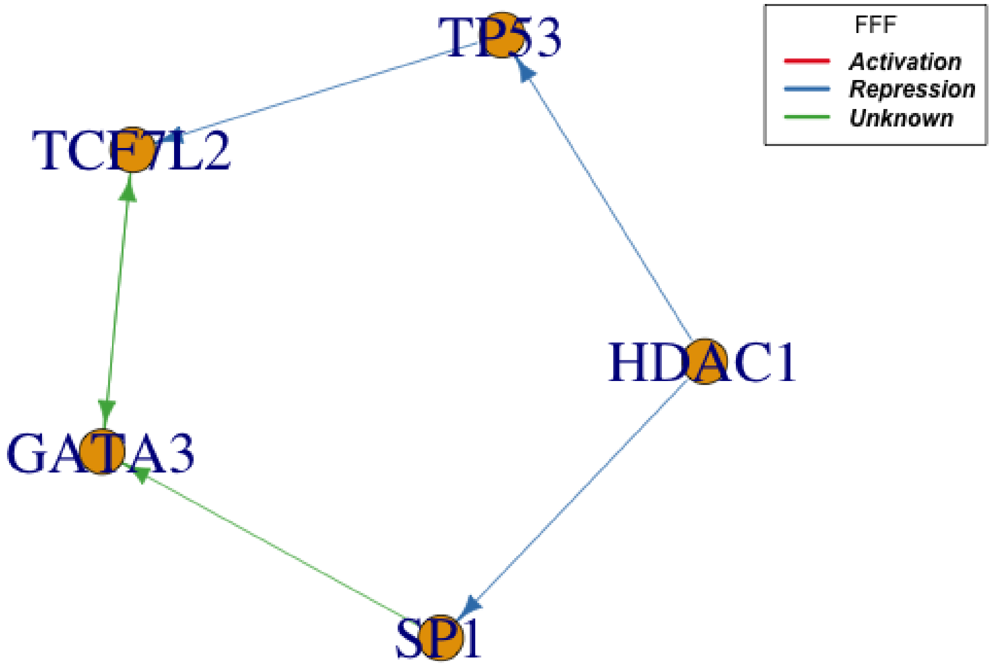

Human TRRUST2

Clocked SR flip-flop

SP3; MYCN; RELA; CIITA; STAT1

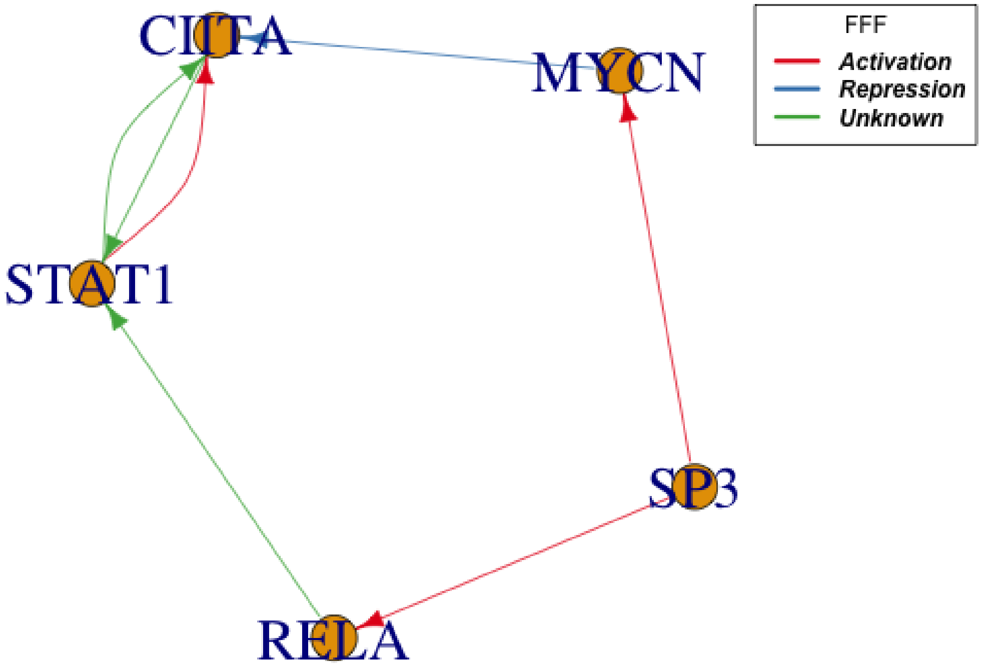

Human TRRUST2

Clocked SR flip-flop

DDIT3; SIRT1; ATF5; TP53; EGR1

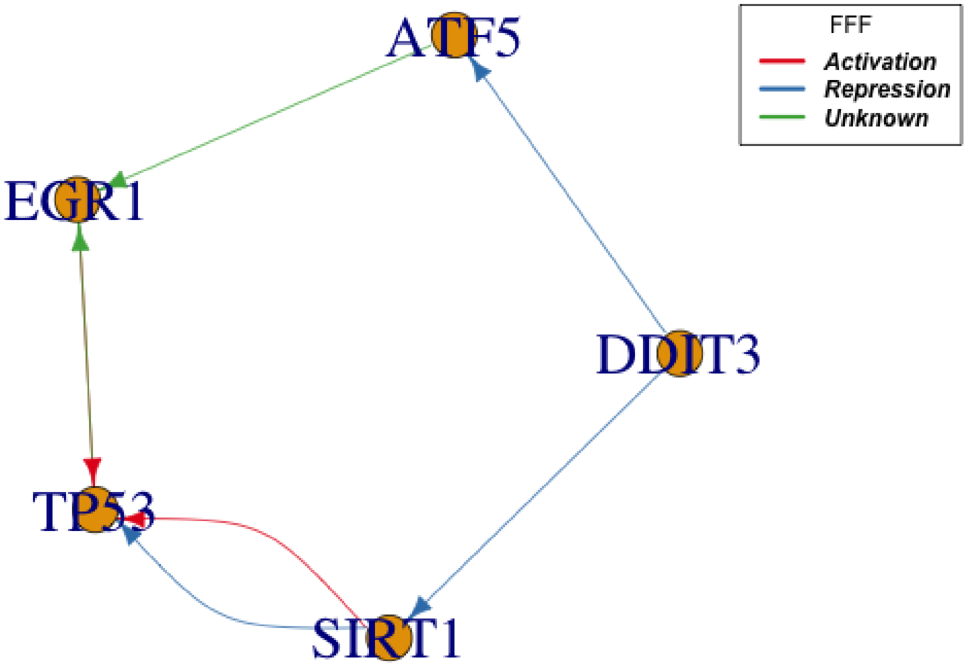

Human TRRUST2

SR flip-flop

STAT1; STAT3

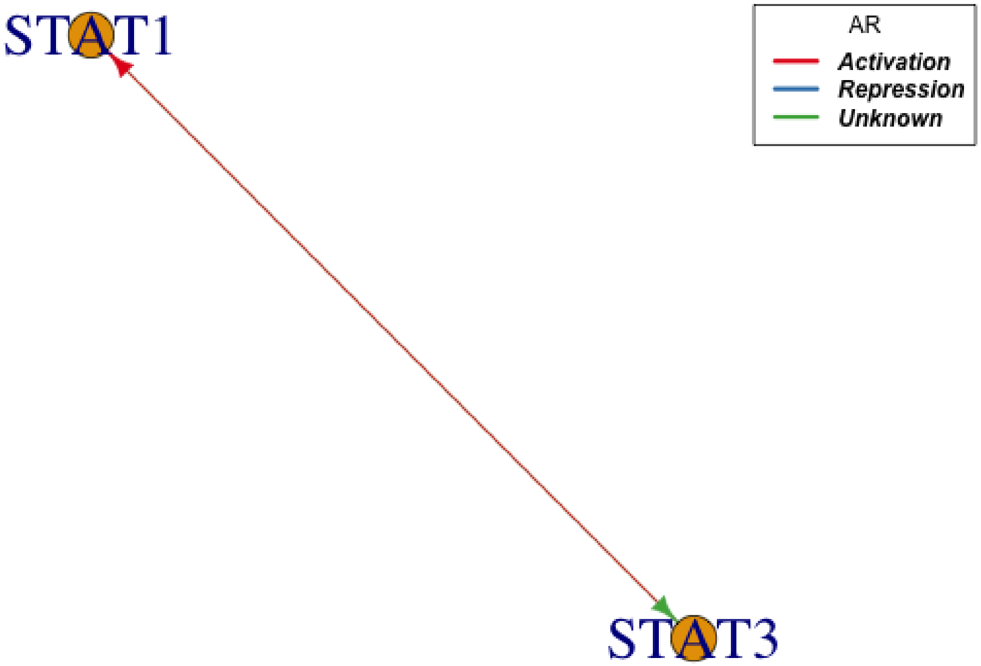

Human TRRUST2

Clocked SR flip-flop

TWIST2; FOS; MYB; NFKB1; GATA3

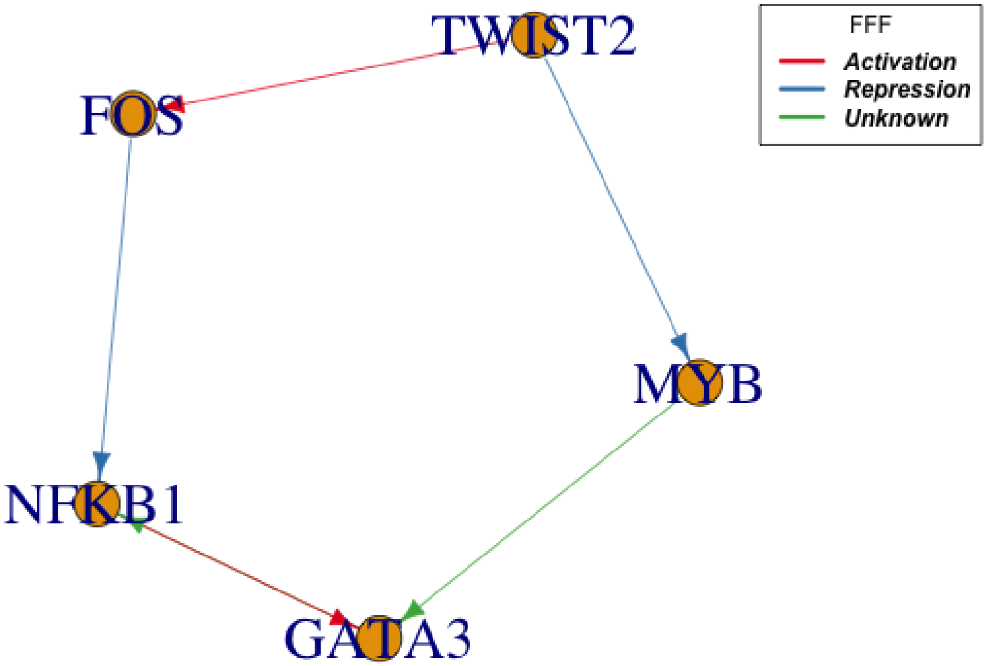

Human TRRUST2

Clocked SR flip-flop

HDAC2; SP1; KLF4; EGR1; TP53

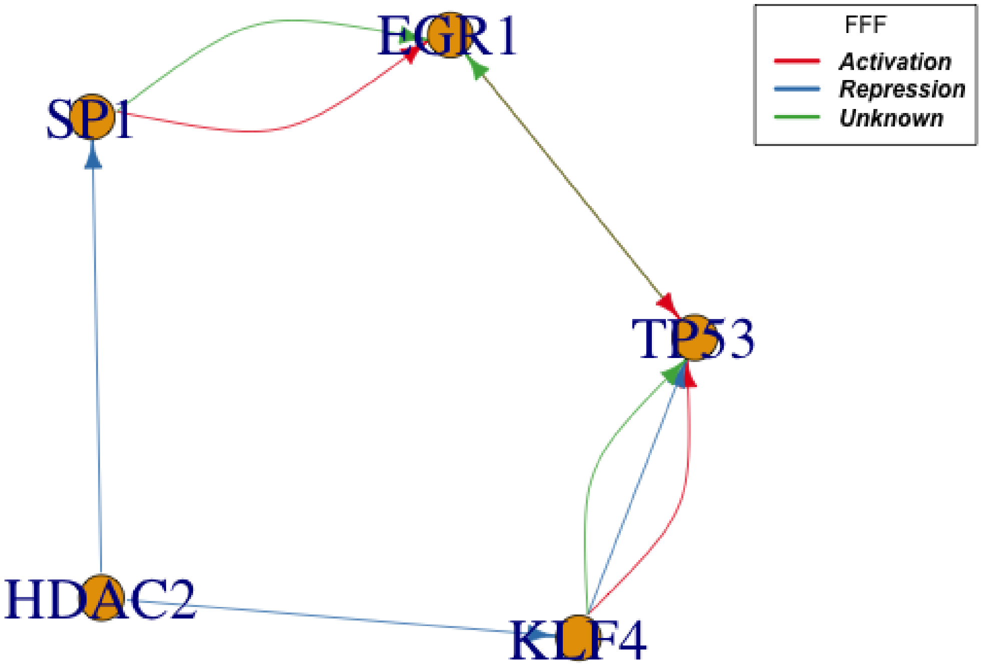

Human TRRUST2

SR flip-flop

SF1; SOX9

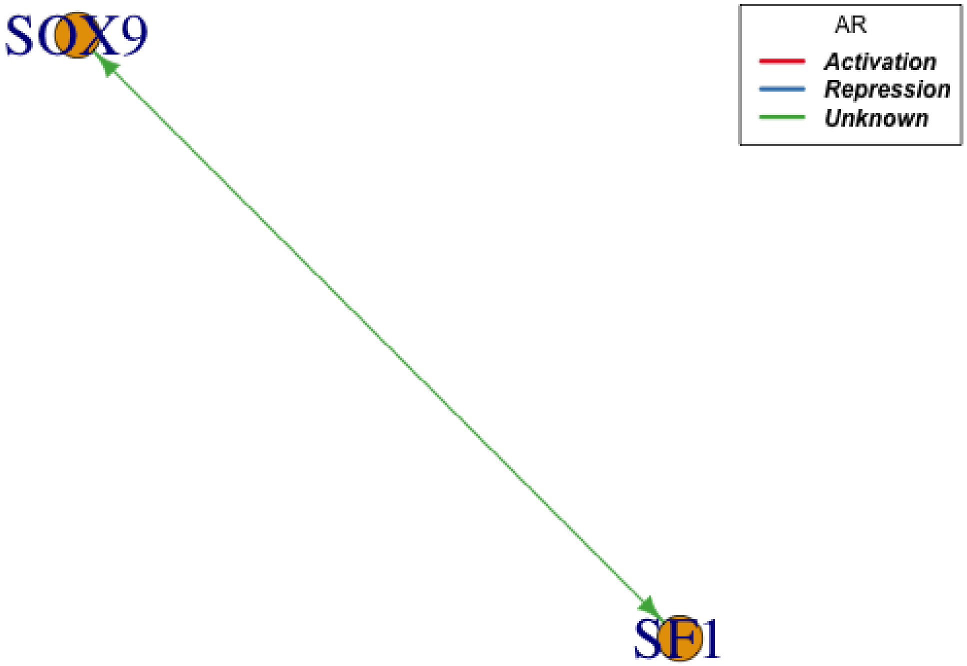

Human TRRUST2

Clocked SR flip-flop

SP1; AR; FOS; IFI16; TP53

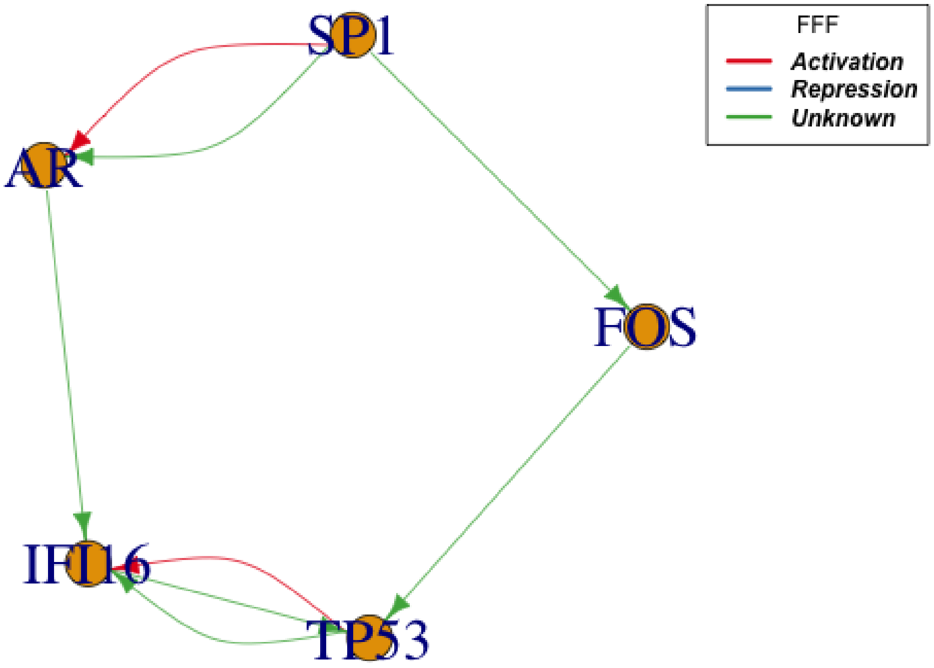

Human TRRUST2

Clocked SR flip-flop

CLOCK; NR3C1; NR0B2; TP53; E2F1

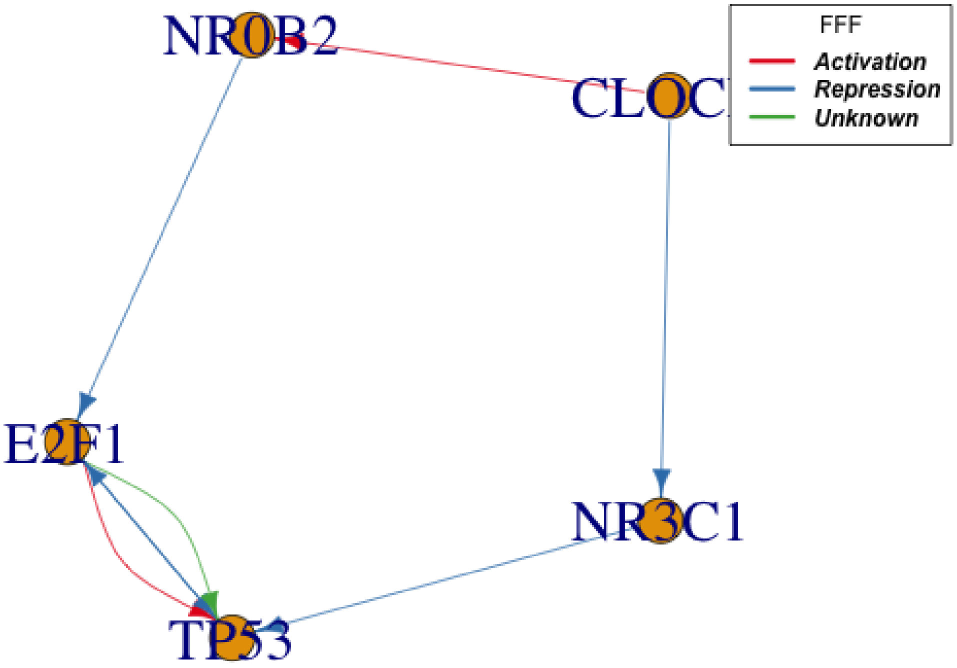

Human TRRUST2

SR flip-flop

AR; RELA

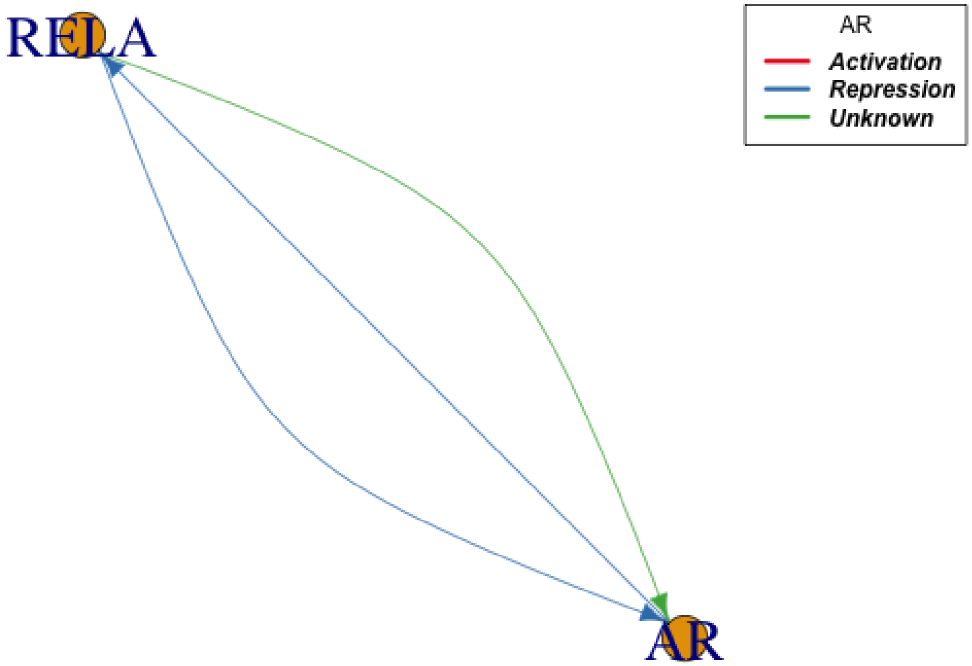

Human TRRUST2

Clocked SR flip-flop

FOXO4; BCL6; CREB1; NFKB1; AR

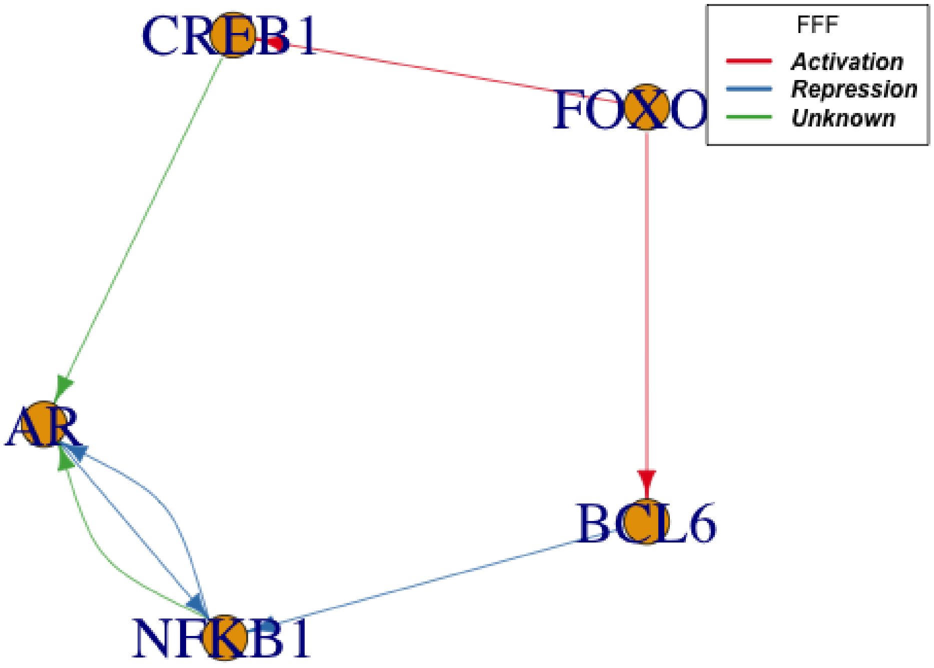

Human TRRUST2

Clocked SR flip-flop

USF1; NFKB1; CIITA; FOXO3; SIRT1

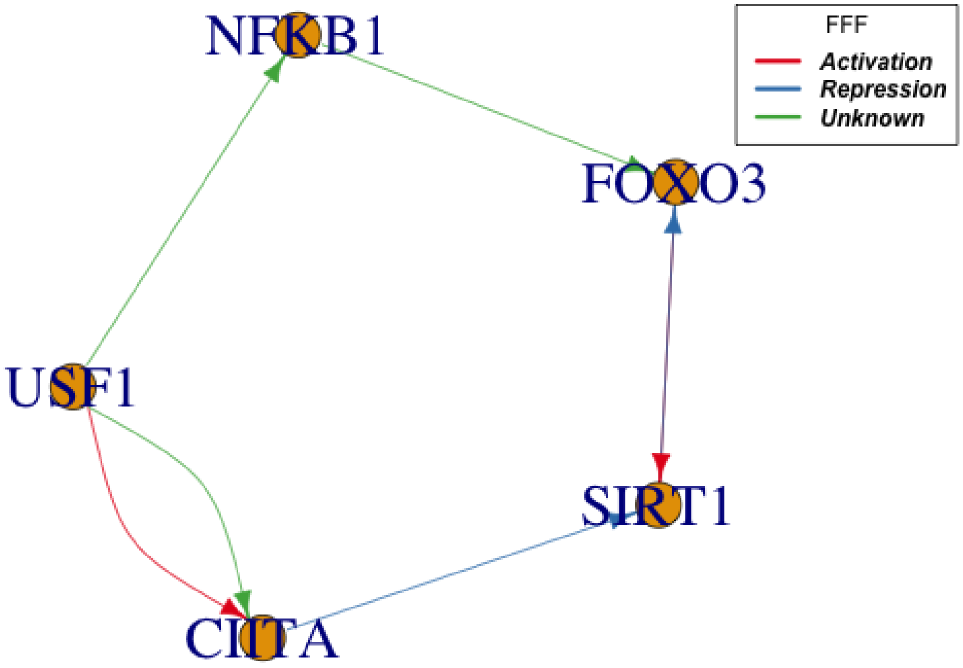

Human TRRUST2

Clocked SR flip-flop

ATF2; JUN; RELA; TP73; E2F1

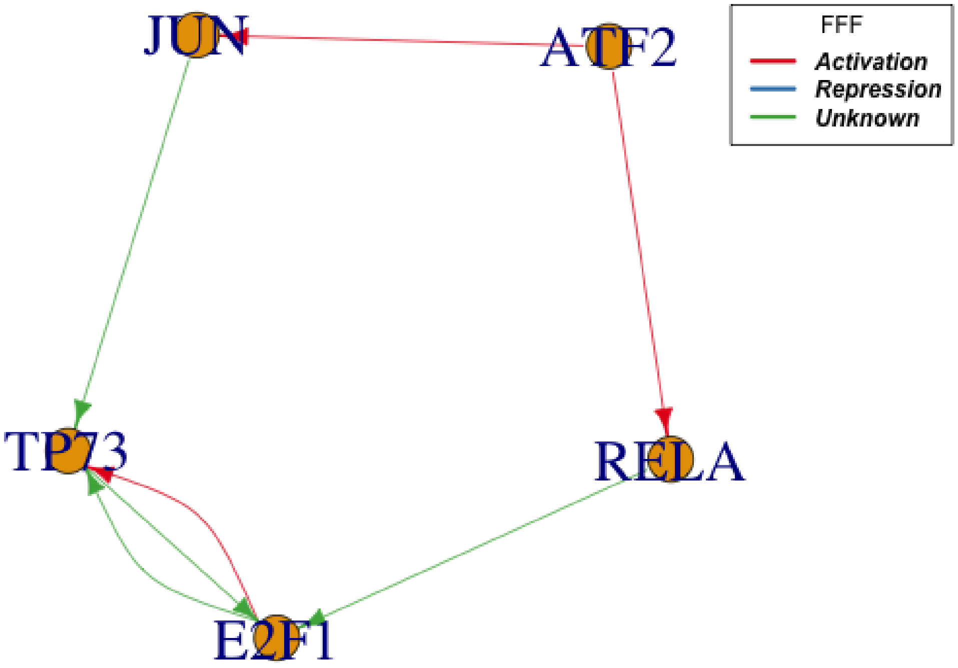

Human TRRUST2

Clocked SR flip-flop

SP1; EGF; SIRT1; STAT1; TP53

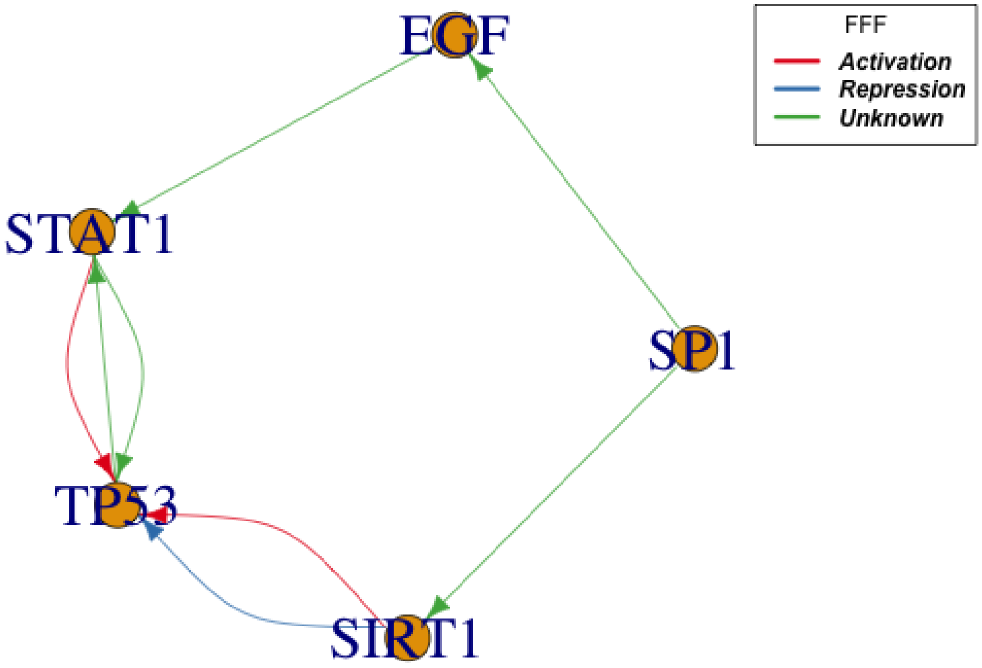

Human TRRUST2

SR flip-flop

CIITA; IRF1

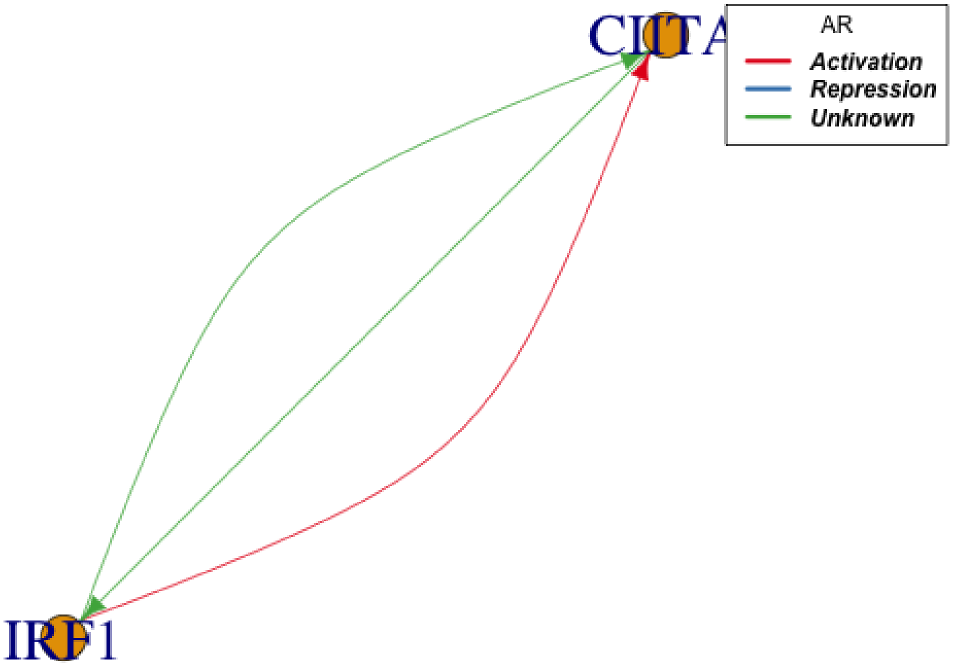

Human TRRUST2

Clocked SR flip-flop

HIPK2; MEF2C; HIF1A; JUN; AR

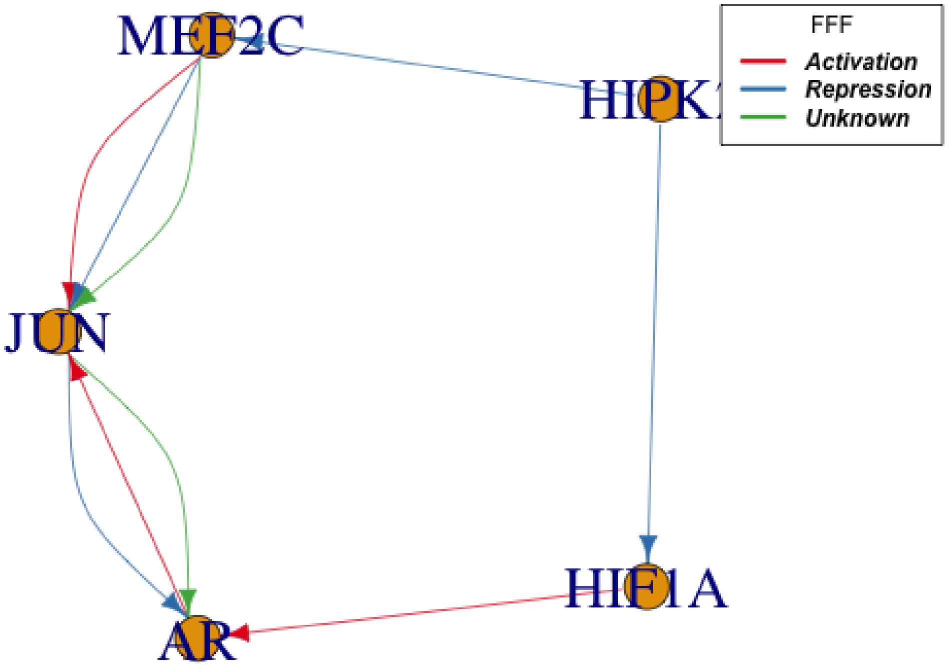

Human TRRUST2

SR flip-flop

PML; TP53

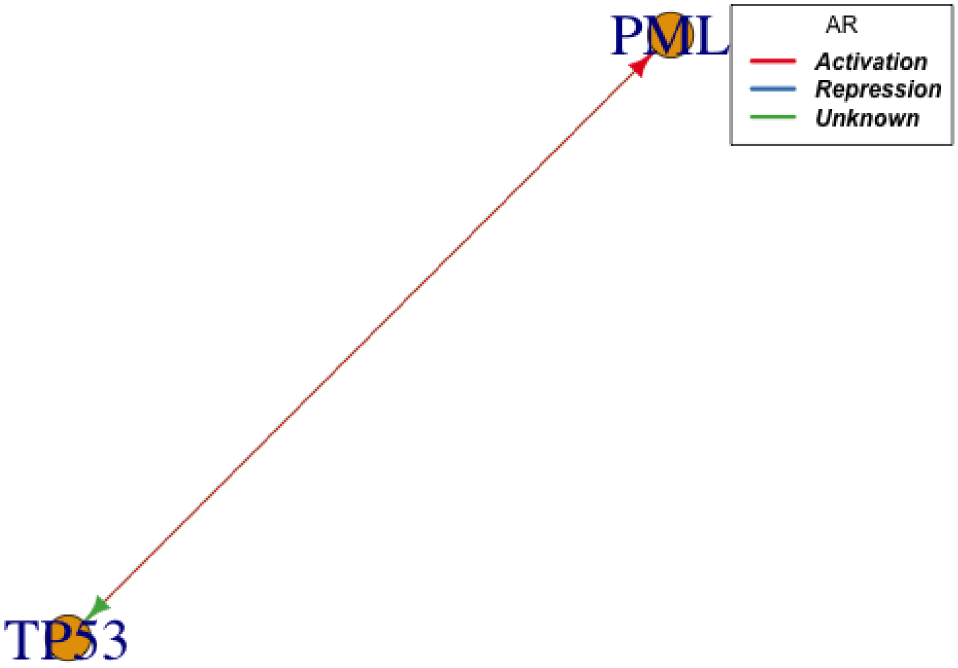

Human TRRUST2

Clocked SR flip-flop

TWIST2; FOS; RUNX2; NFKB1; AR

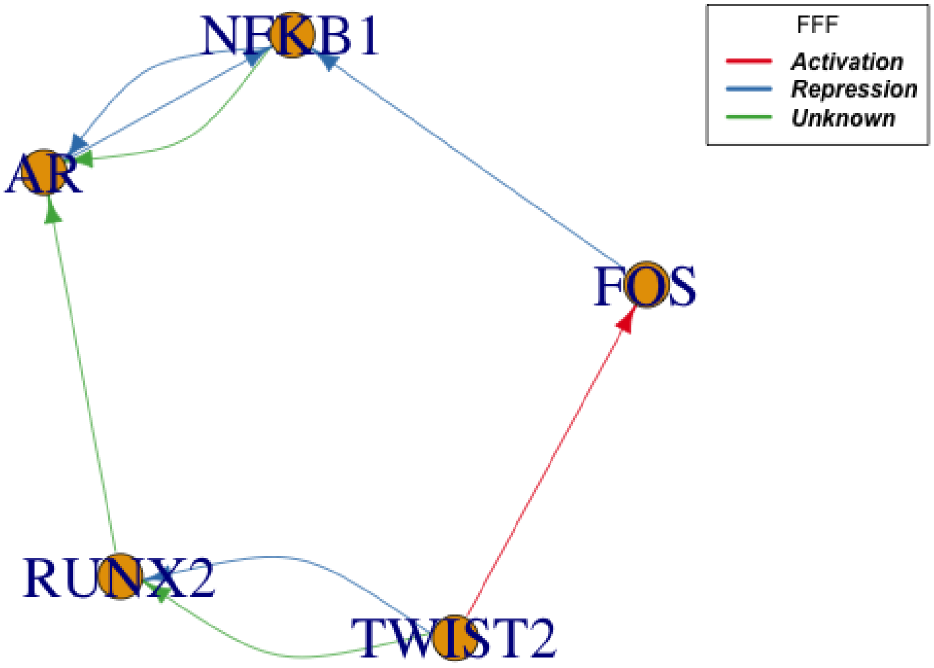

Human TRRUST2

Clocked SR flip-flop

TWIST1; YBX1; NF1; E2F1; TP53

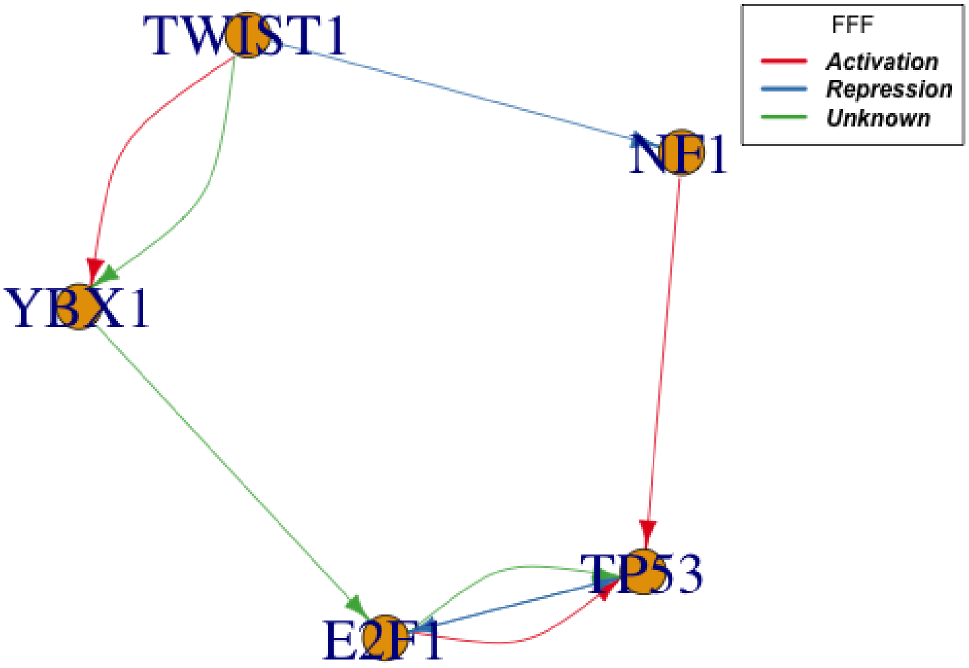

Human TRRUST2

Clocked SR flip-flop

USF1; RELA; APC; HDAC1; DNMT1

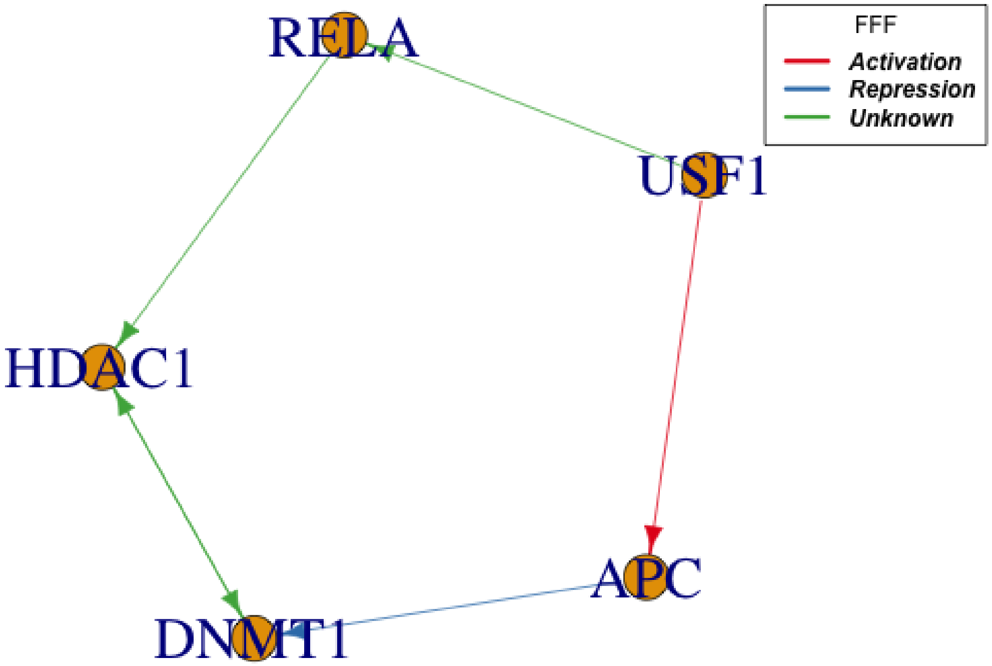

Human TRRUST2

Clocked SR flip-flop

ARID3A; RBL1; PML; MYC; EP300

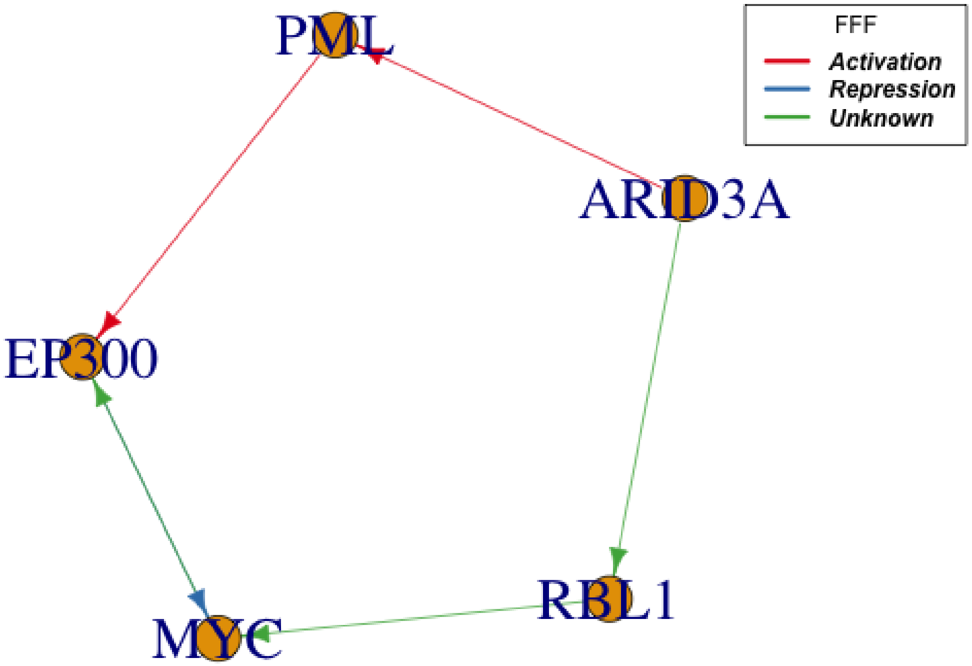

Human TRRUST2

Clocked SR flip-flop

POU2F1; TWIST1; GATA1; MYB; WT1

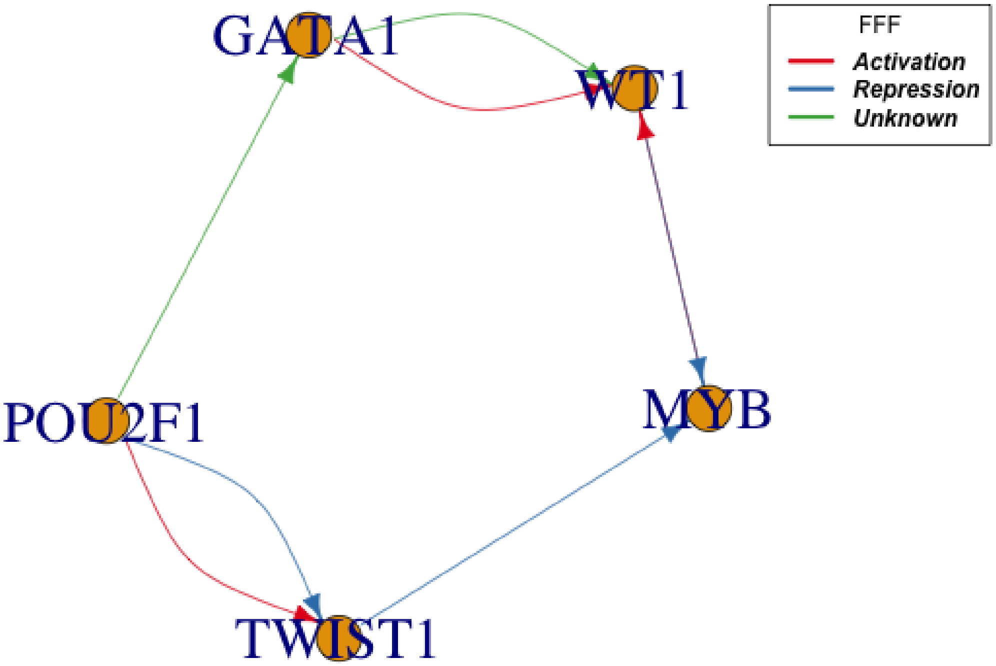

Human TRRUST2

Clocked SR flip-flop

NFKB1; HDAC7; YY1; ESR1; BRCA1

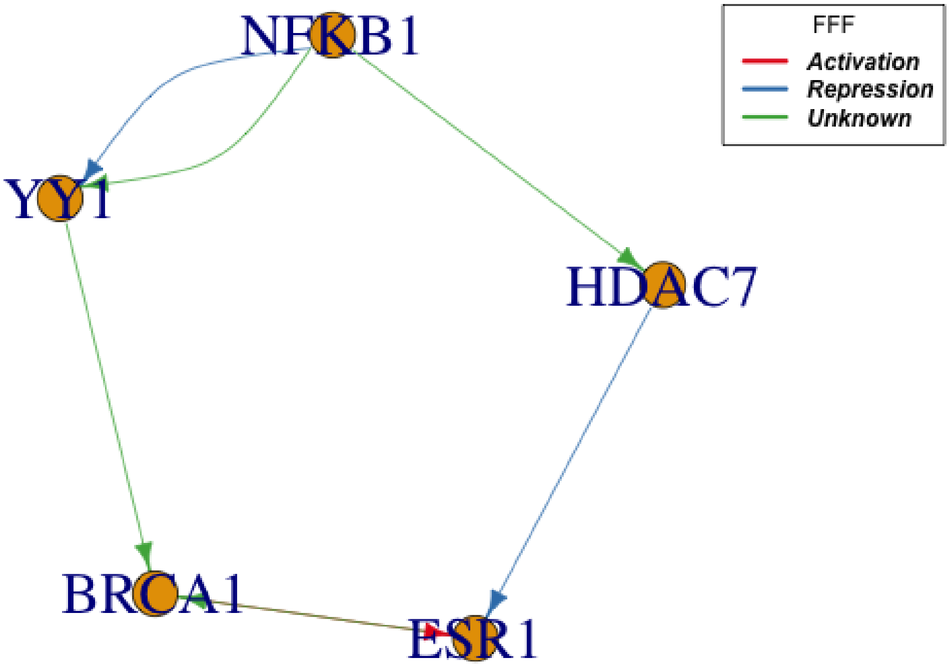

Human TRRUST2

SR flip-flop

HMGA1; SP1

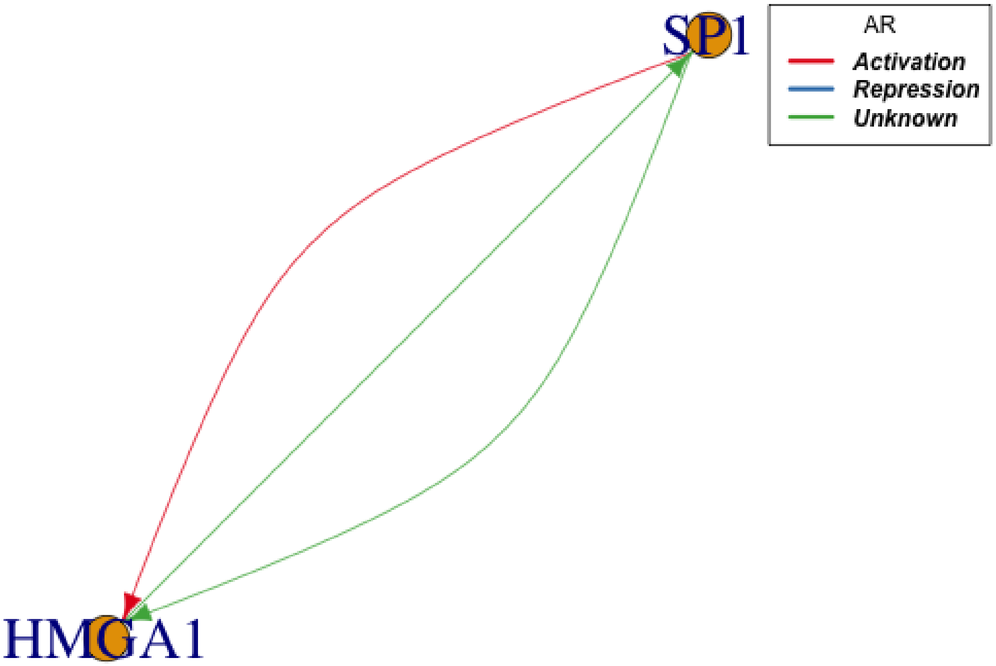

Human TRRUST2

SR flip-flop

ATF4; TRIB3

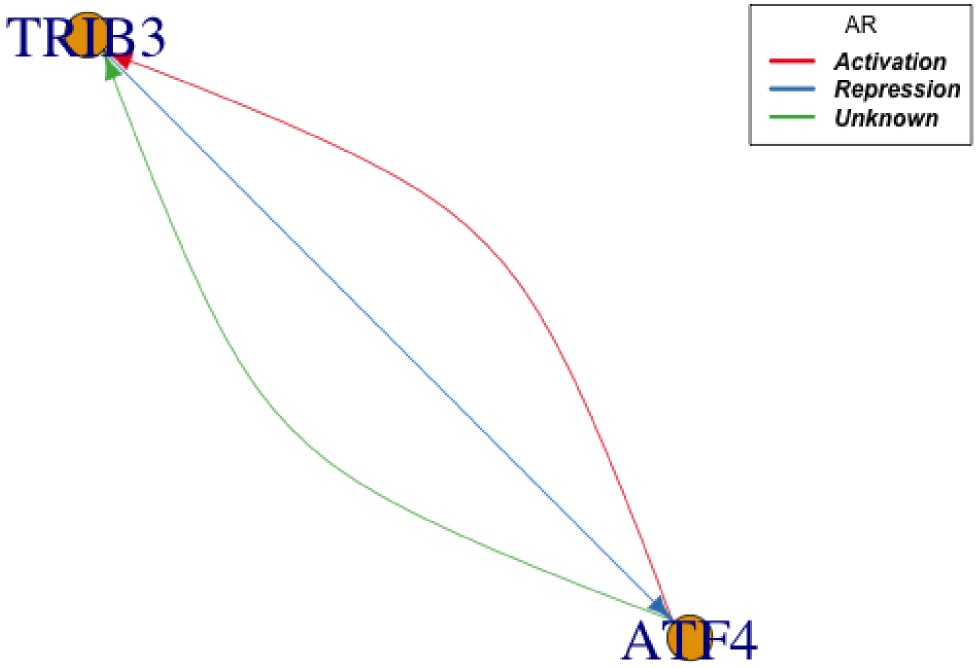

Human TRRUST2

SR flip-flop

FOXO3; SIRT1

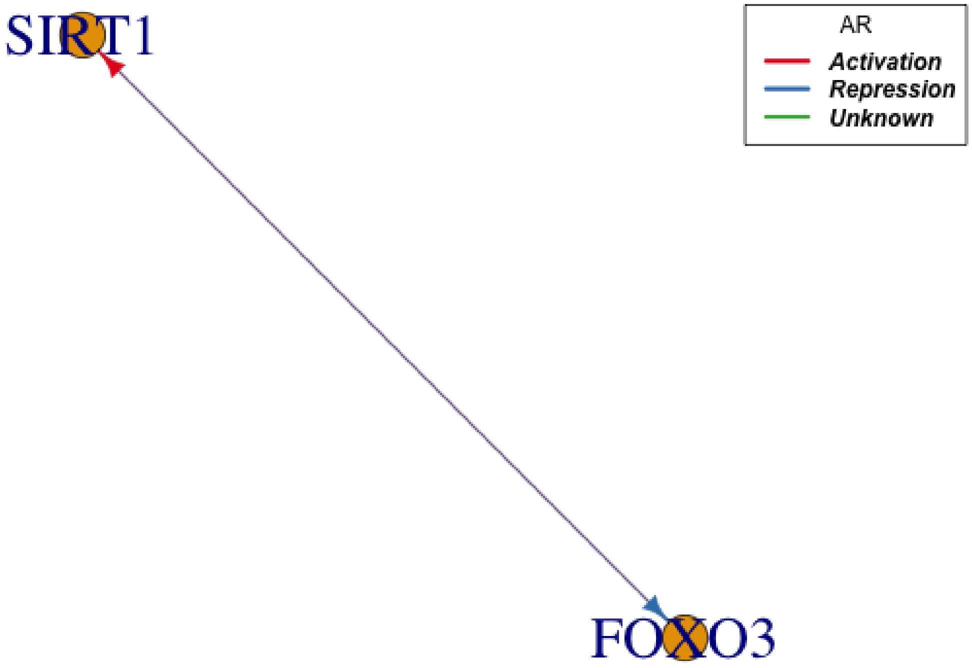

Human TRRUST2

Clocked SR flip-flop

HDAC7; HIF1A; MEF2A; AR; JUN

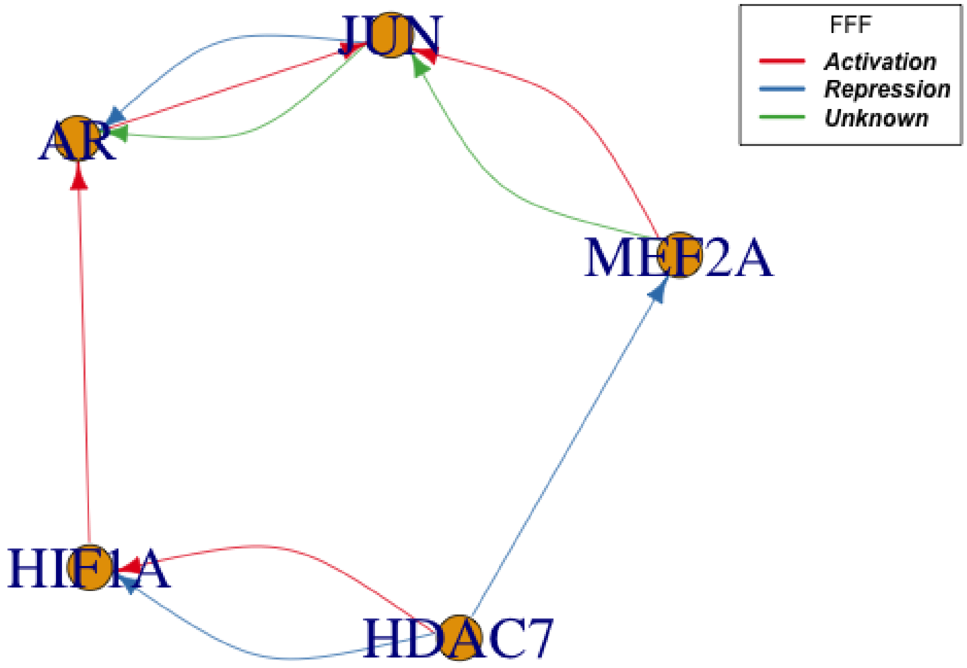

Human TRRUST2

Clocked SR flip-flop

SRF; FOSL1; FOS; EZH2; TP53

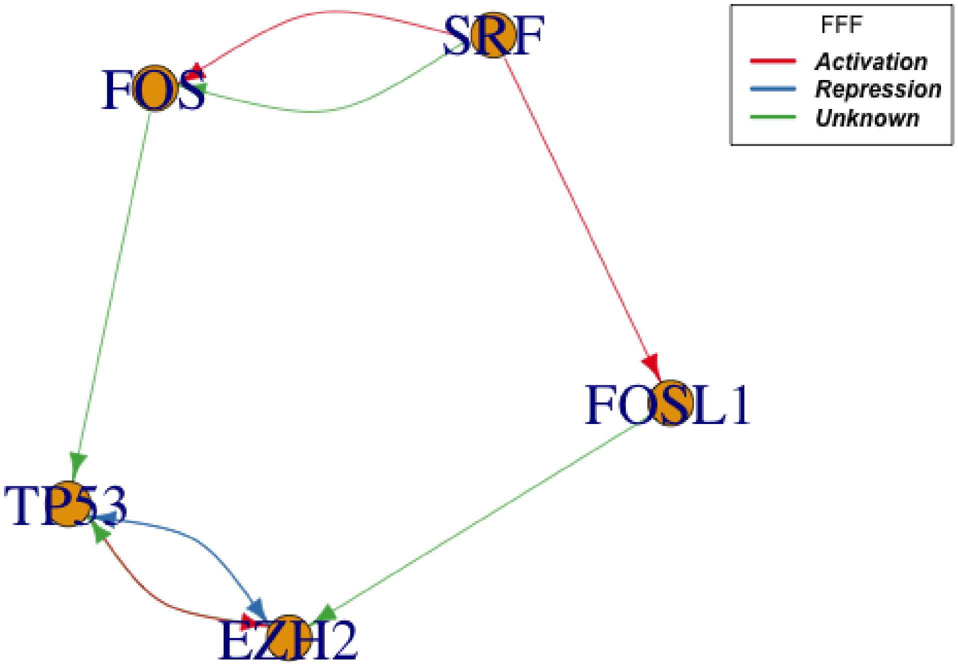

Human TRRUST2

Clocked SR flip-flop

ETS1; MYB; FLI1; MYC; EP300

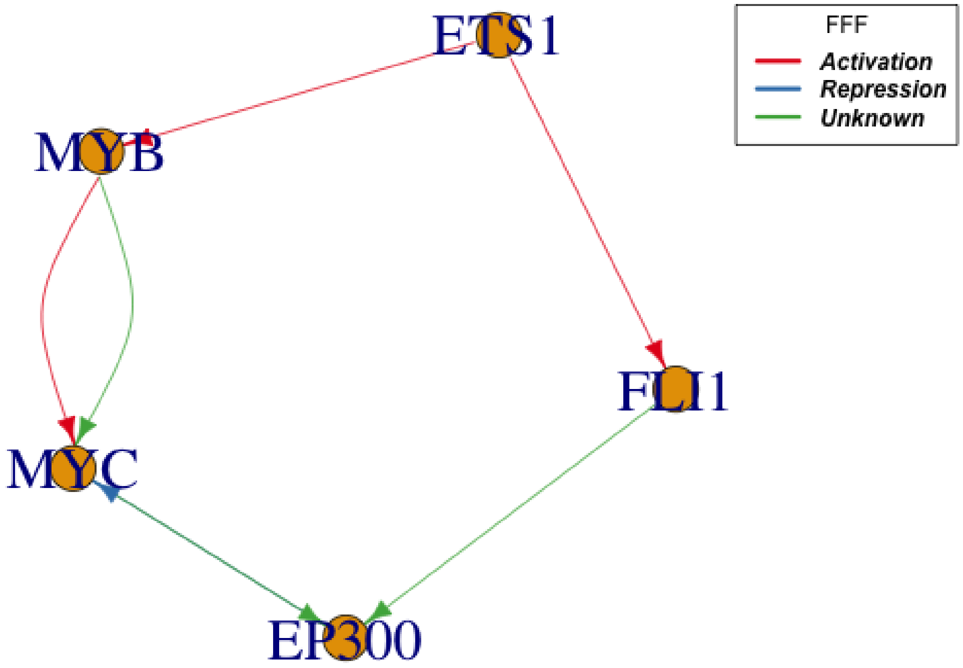

Human TRRUST2

SR flip-flop

PIAS3; STAT3

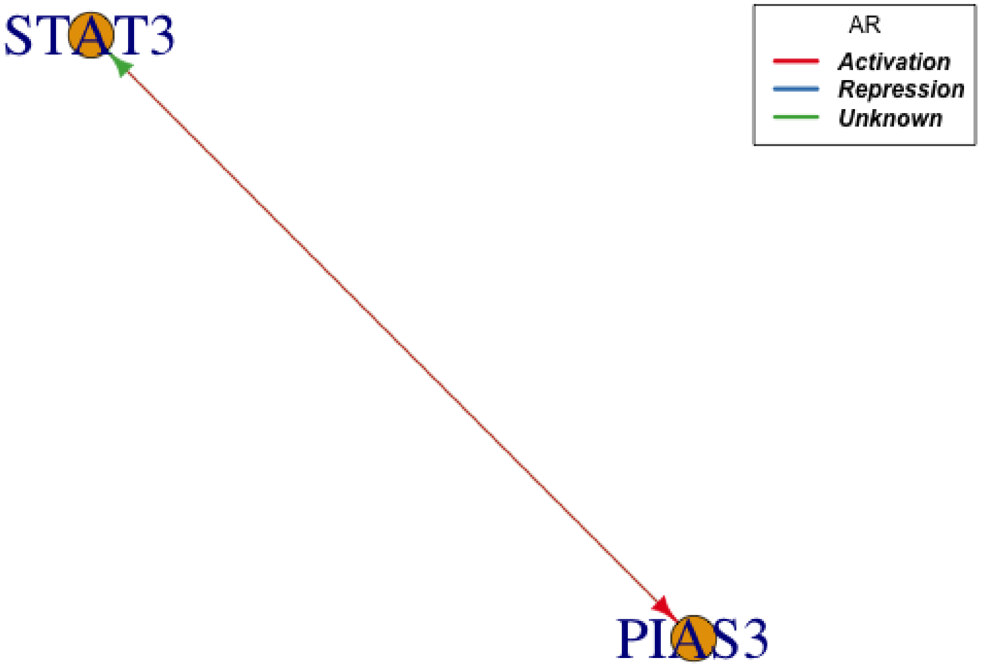

Human TRRUST2

Clocked SR flip-flop

SP1; EGF; FOS; STAT1; TP53

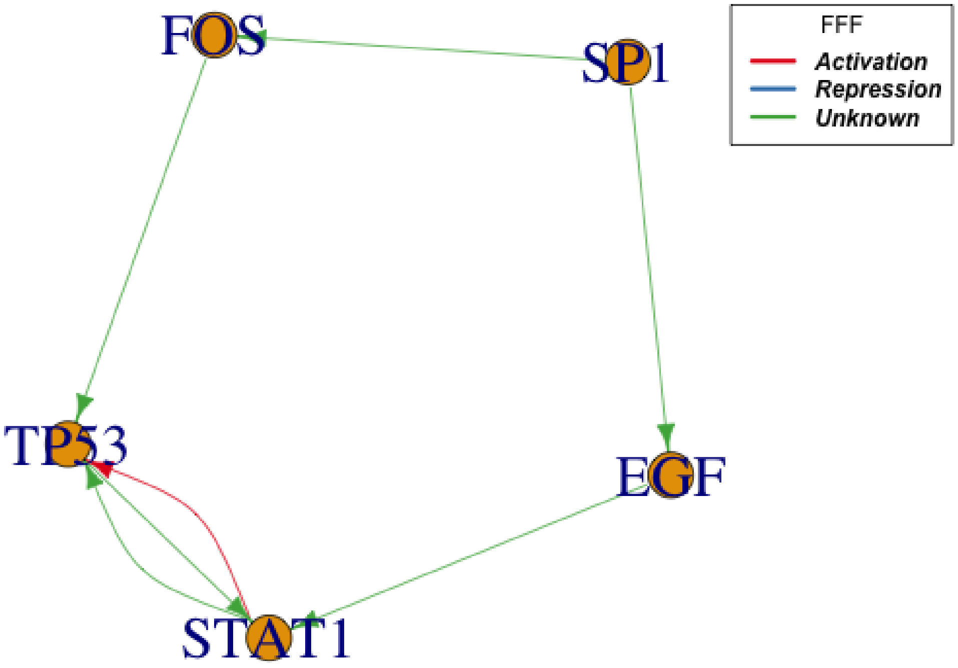

Human TRRUST2

Clocked SR flip-flop

RELA; YY1; NCOR2; HDAC2; MYCN

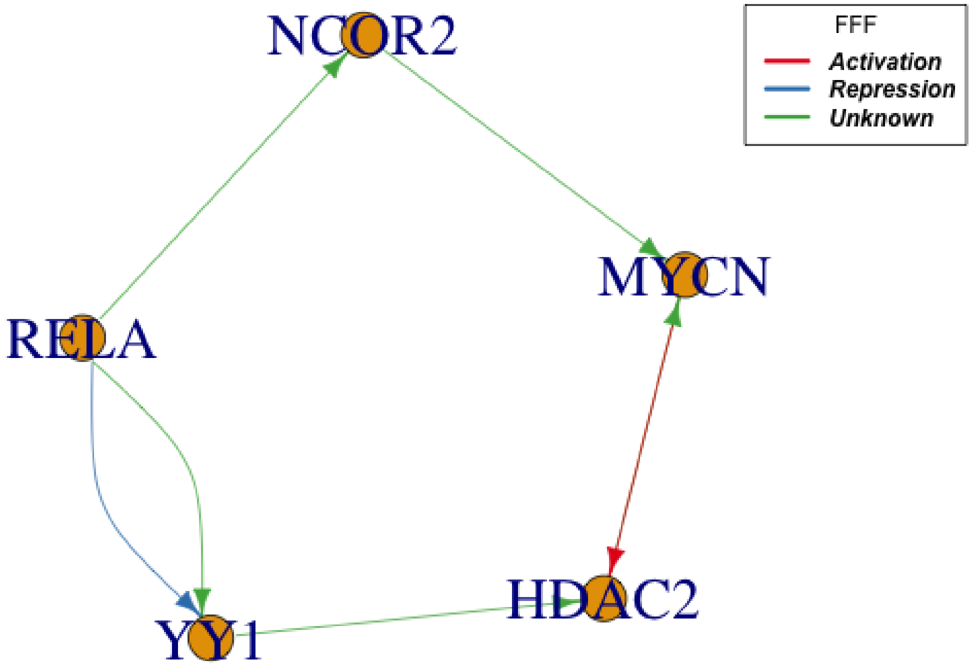

Human TRRUST2

Clocked SR flip-flop

MYB; CEBPA; NR3C1; STAT3; STAT1

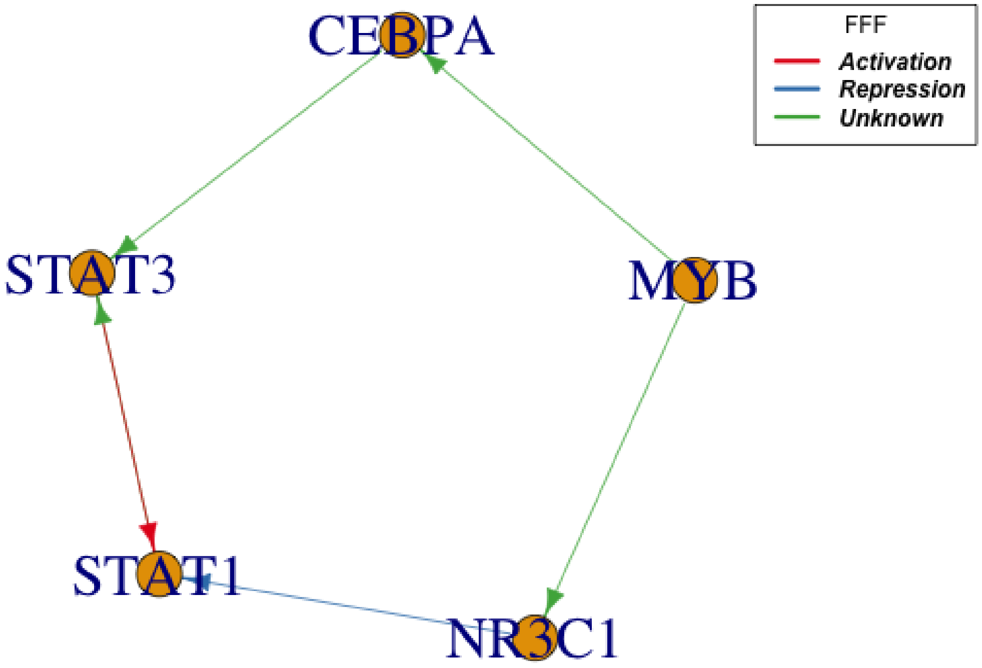

Human TRRUST2

Clocked SR flip-flop

CLOCK; KAT5; NR3C1; STAT3; TP53

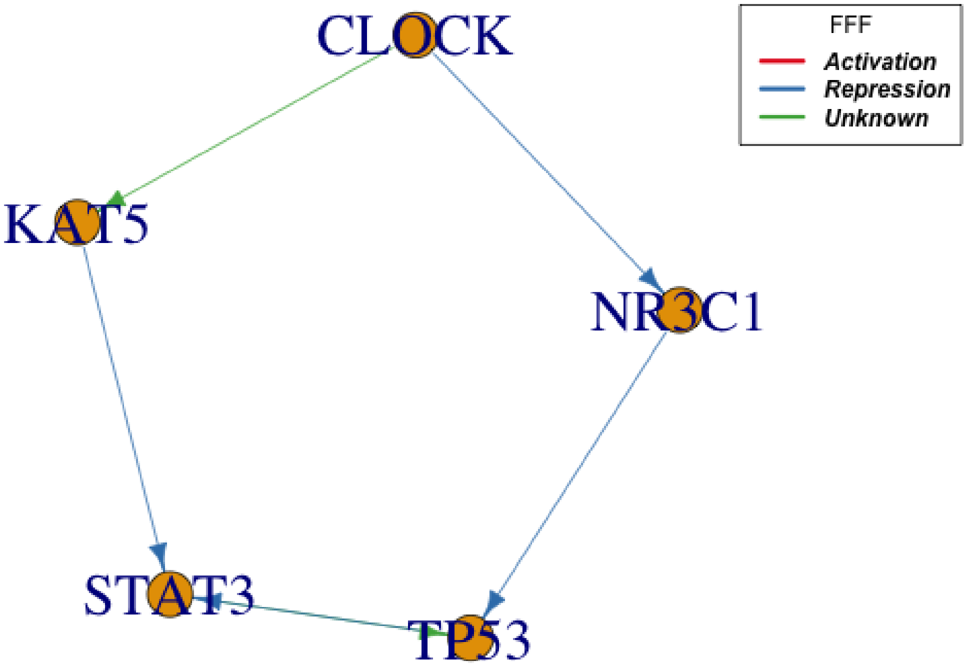

Human TRRUST2

Clocked SR flip-flop

RUNX1; SPI1; KLF4; STAT3; TP53

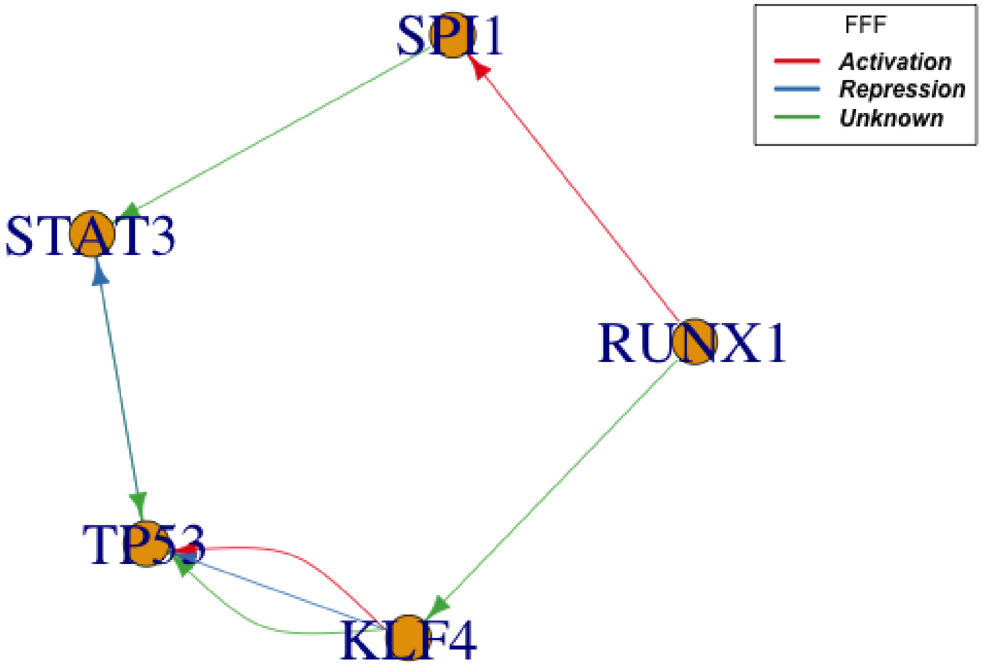

Human TRRUST2

Clocked SR flip-flop

E2F4; PPARG; AR; TP53; IFI16

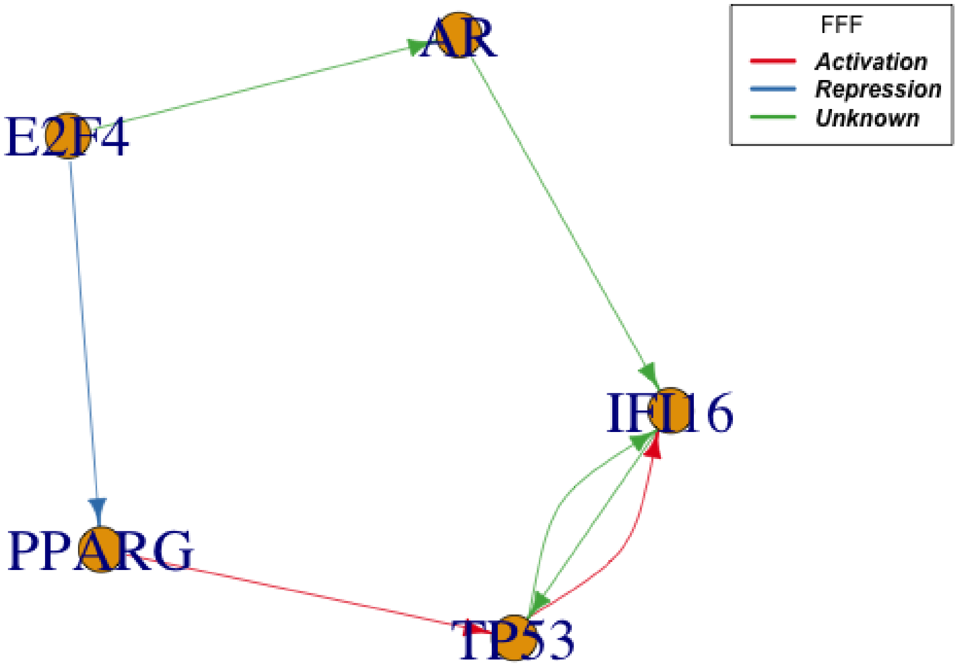

Human TRRUST2

Clocked SR flip-flop

LMO2; LYL1; LEF1; NFKB1; AR

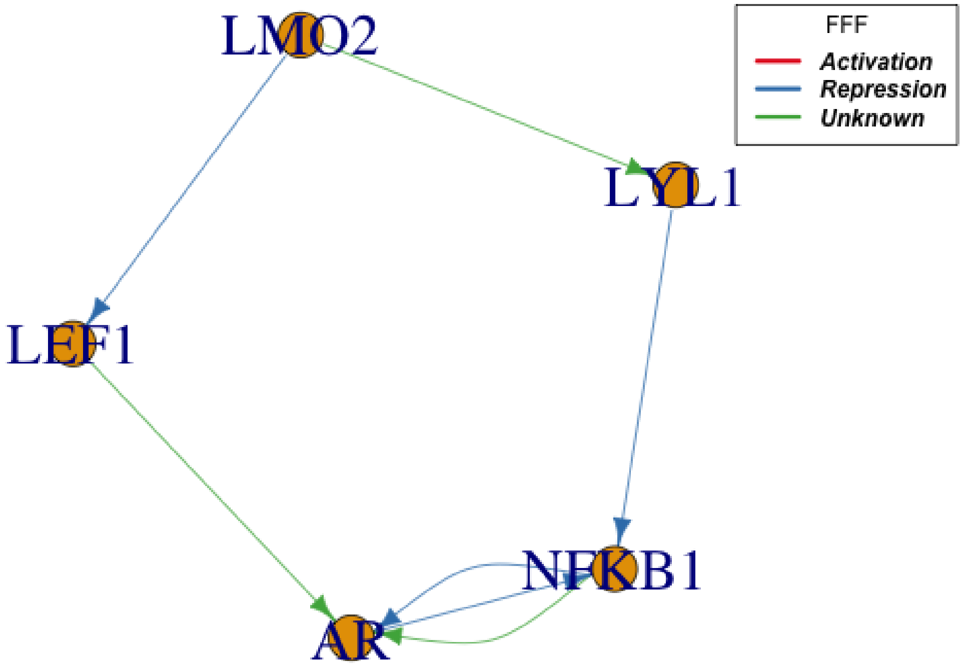

Human TRRUST2

SR flip-flop

HIC1; SIRT1

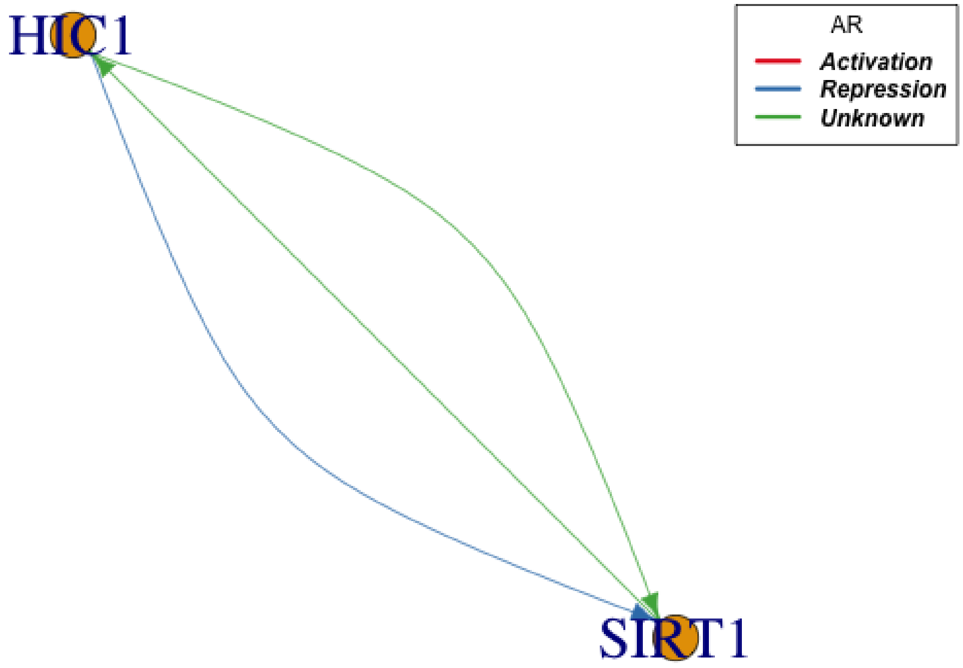

Mouse TRRUST

Clocked SR flip-flop

SPI1; TAL1; NFKB1; TCF3; LEF1

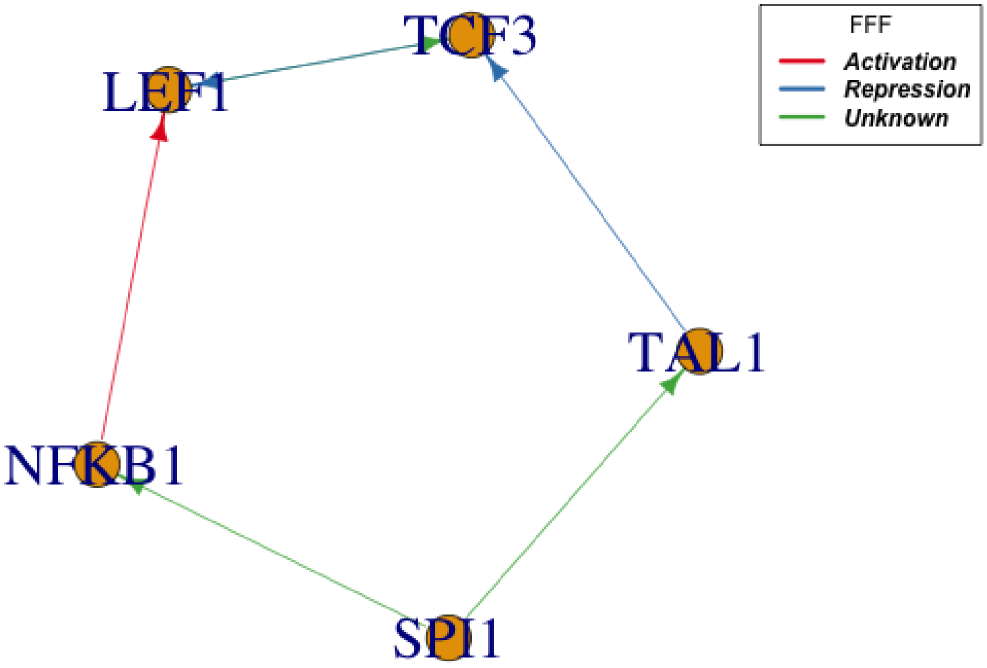

Mouse TRRUST

Clocked SR flip-flop

NR5A1; POU5F1; PBX1; PAX2; PAX6

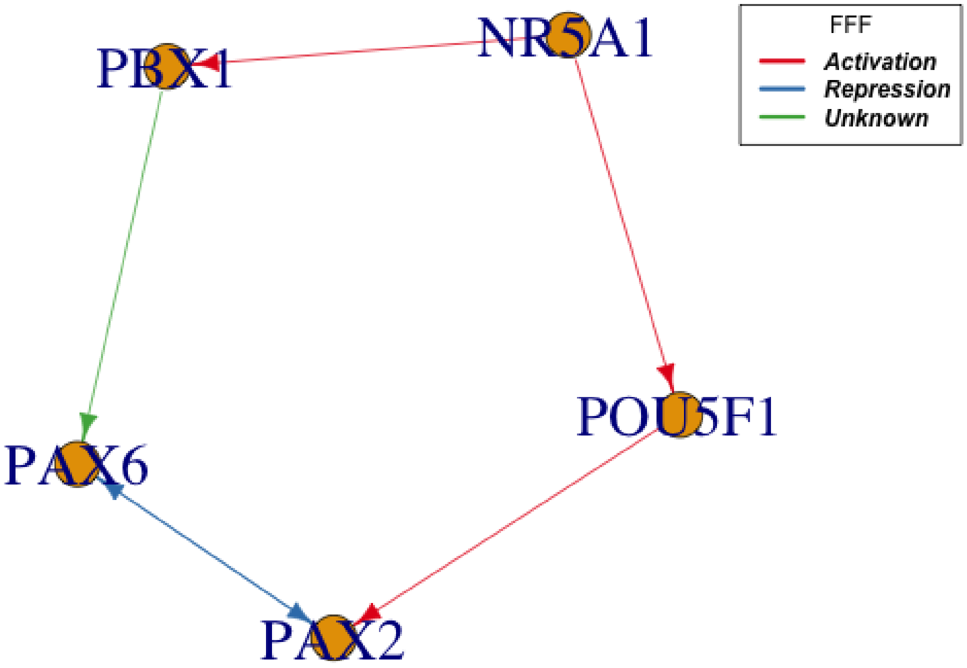

Mouse TRRUST

Clocked SR flip-flop

NFKB1; KLF5; CTNNB1; POU5F1; CDX2

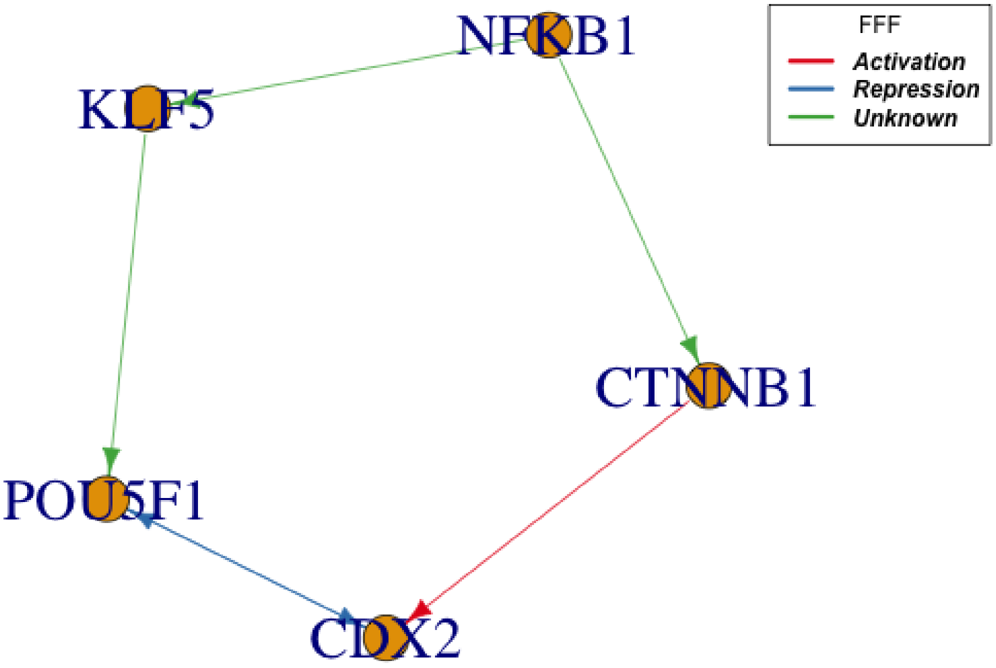

Mouse TRRUST

Clocked SR flip-flop

TFAP2C; DMRT1; DNMT3B; SOX2; POU5F1

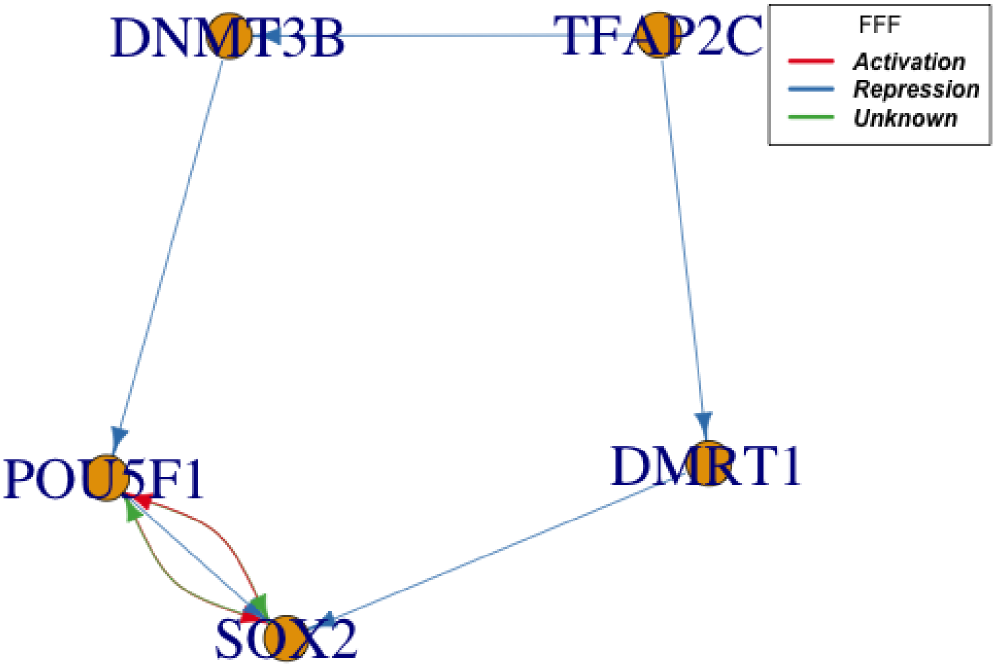

Mouse TRRUST

SR flip-flop

RELA; TRP53

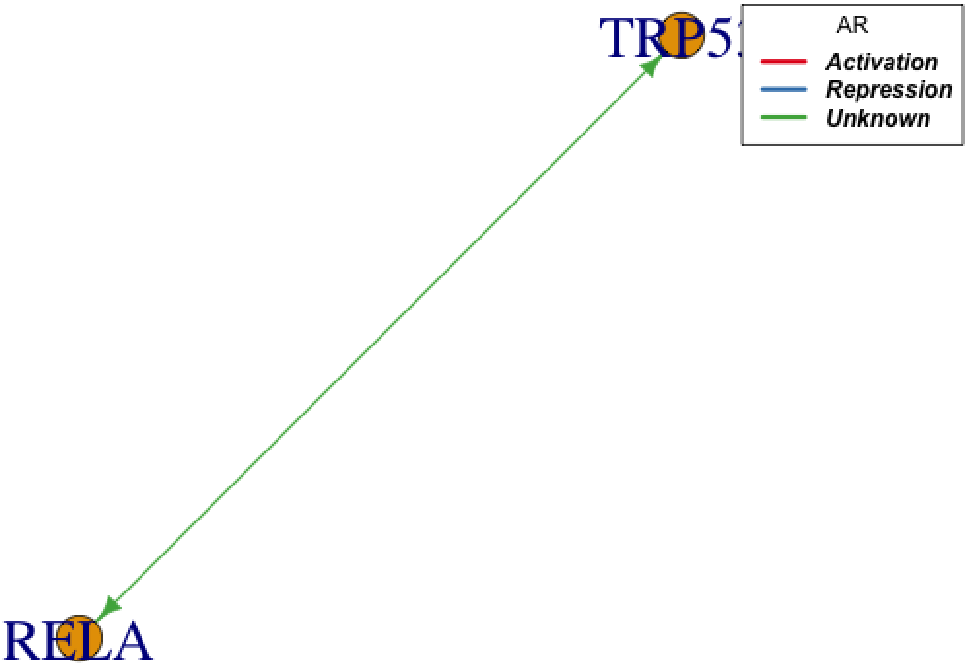

Mouse TRRUST

Clocked SR flip-flop

BRCA1; HMGA2; SIRT1; NFKB1; TRP53

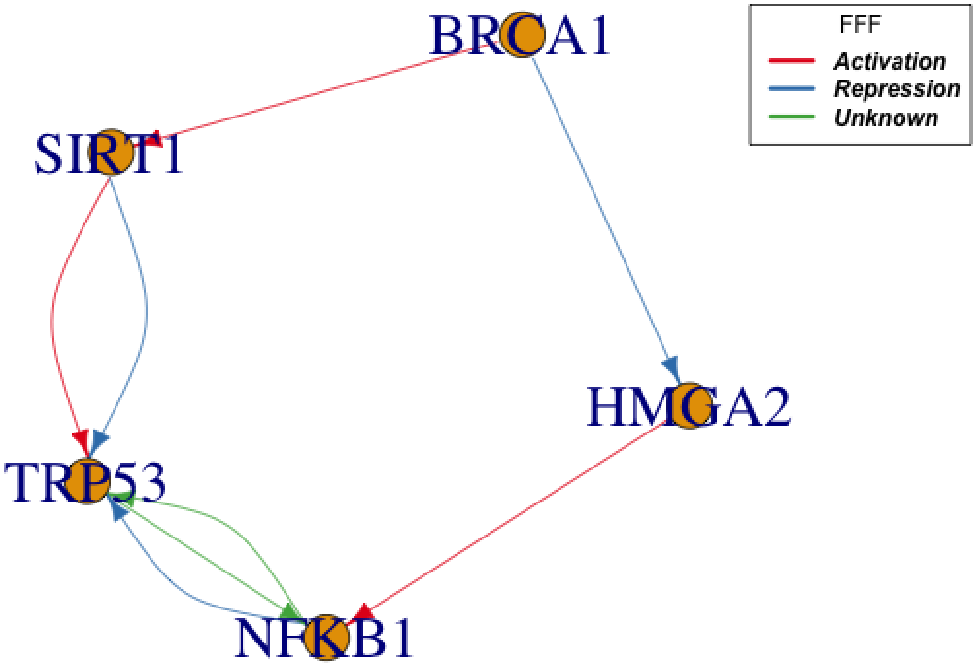

Mouse TRRUST

Clocked SR flip-flop

ID2; SPI1; TCF3; NFKB1; FOXP3

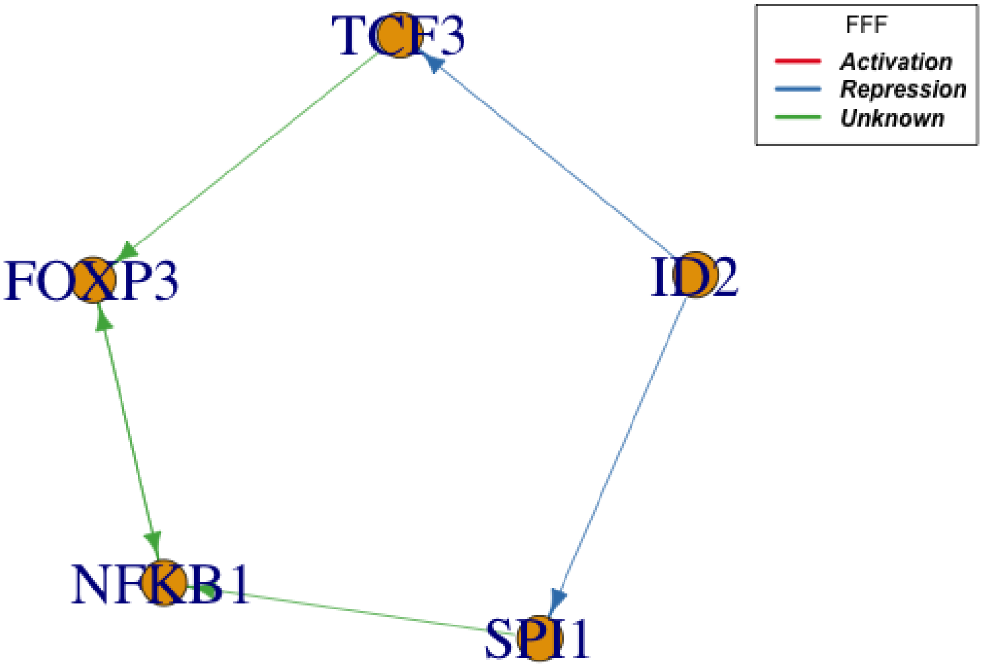

Mouse TRRUST

SR flip-flop

ARNTL; PER1

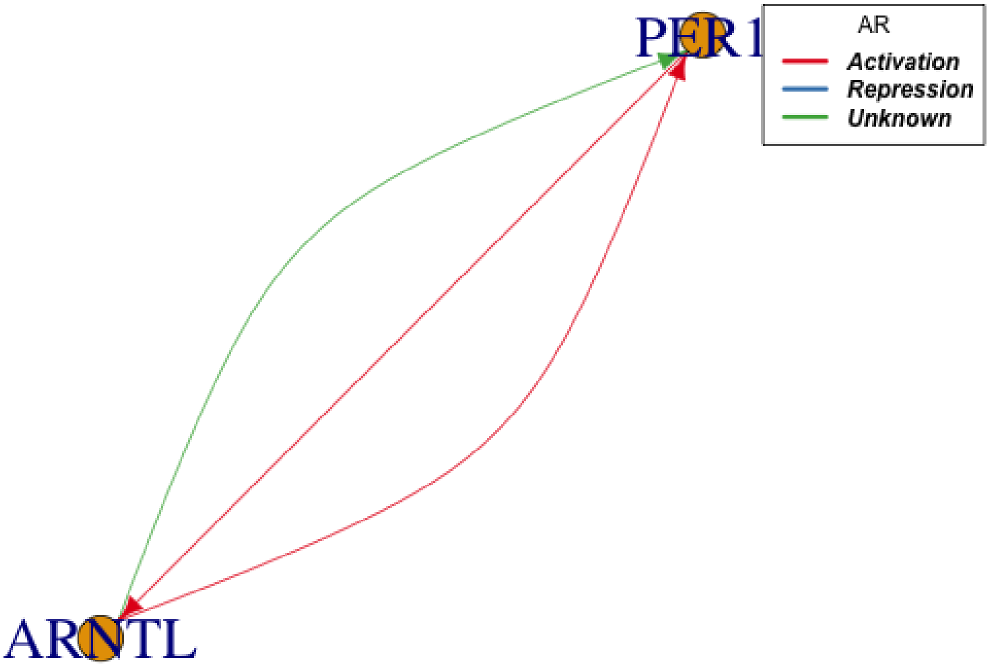

Mouse TRRUST

Clocked SR flip-flop

TCF21; AR; NR5A1; SOX2; POU5F1

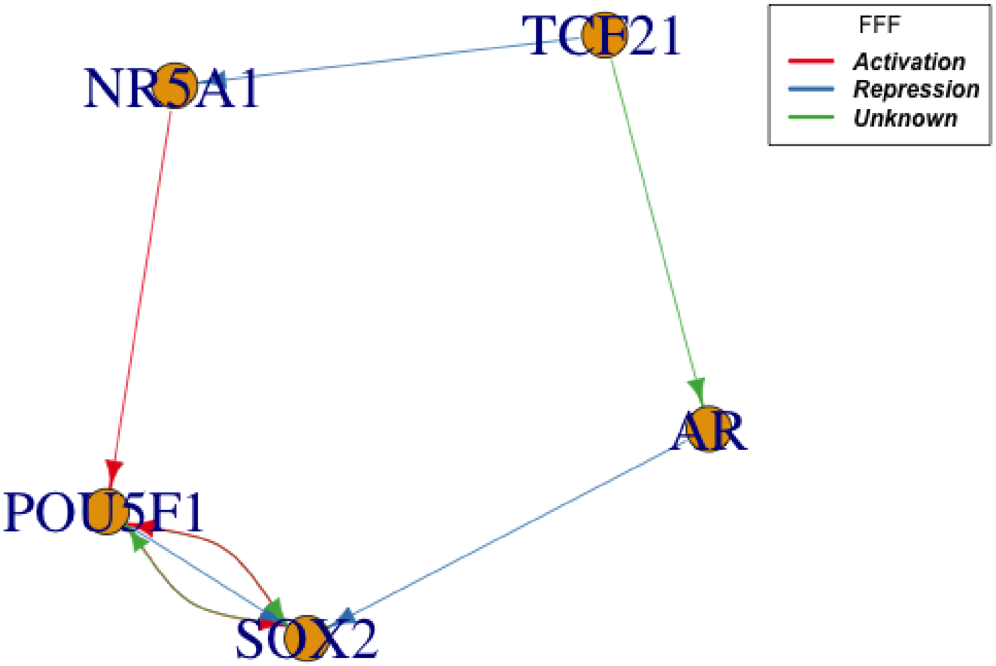

Mouse TRRUST

Clocked SR flip-flop

EP300; POU5F1; NEUROD1; PAX2; PAX6

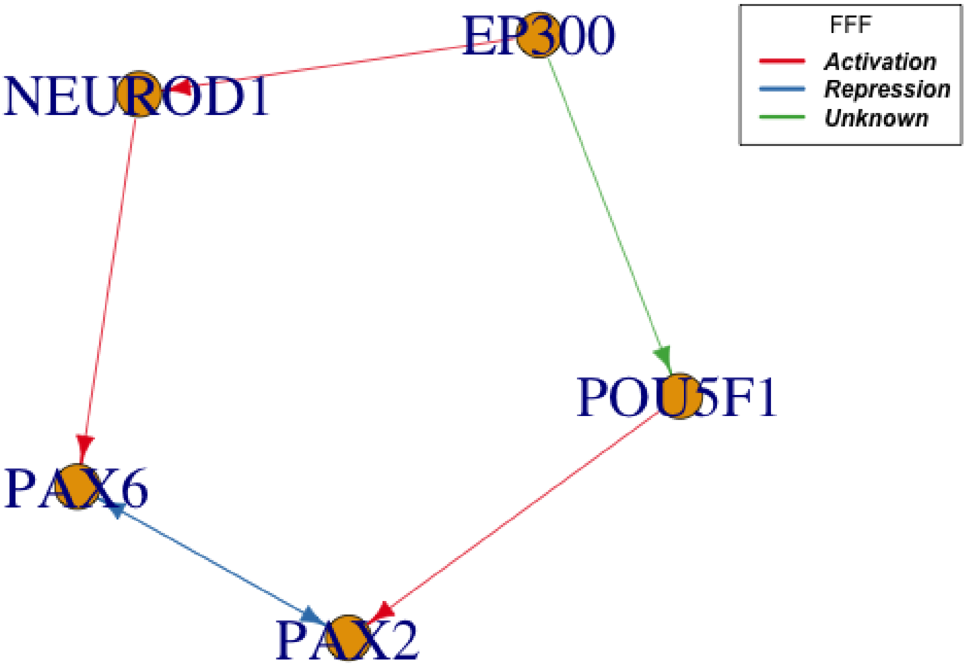

Mouse TRRUST

SR flip-flop

AR; NKX3_1

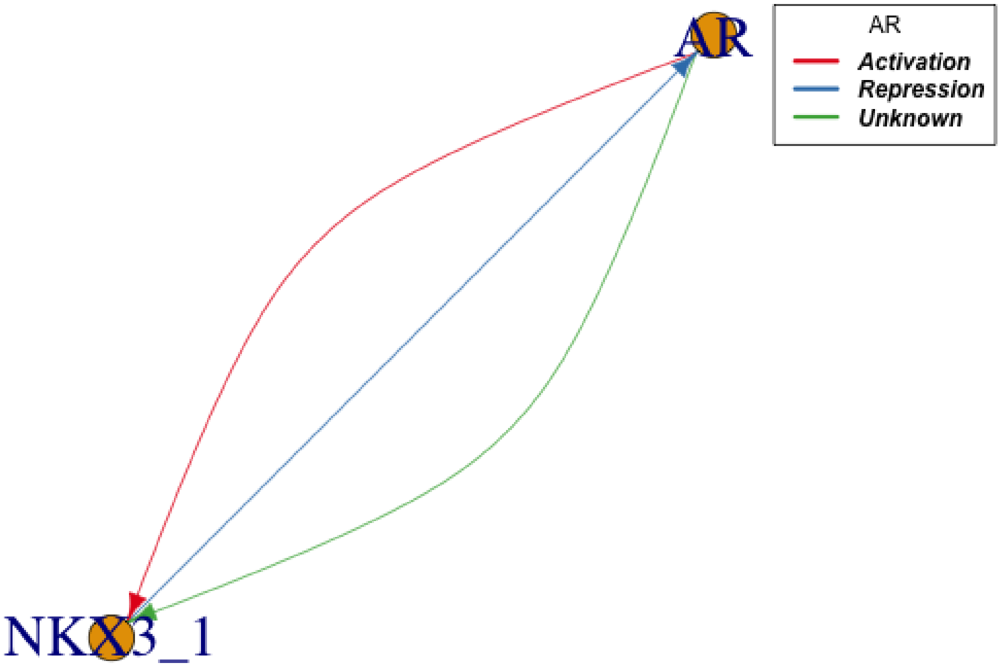

Mouse TRRUST

SR flip-flop

PITX2; TBX1

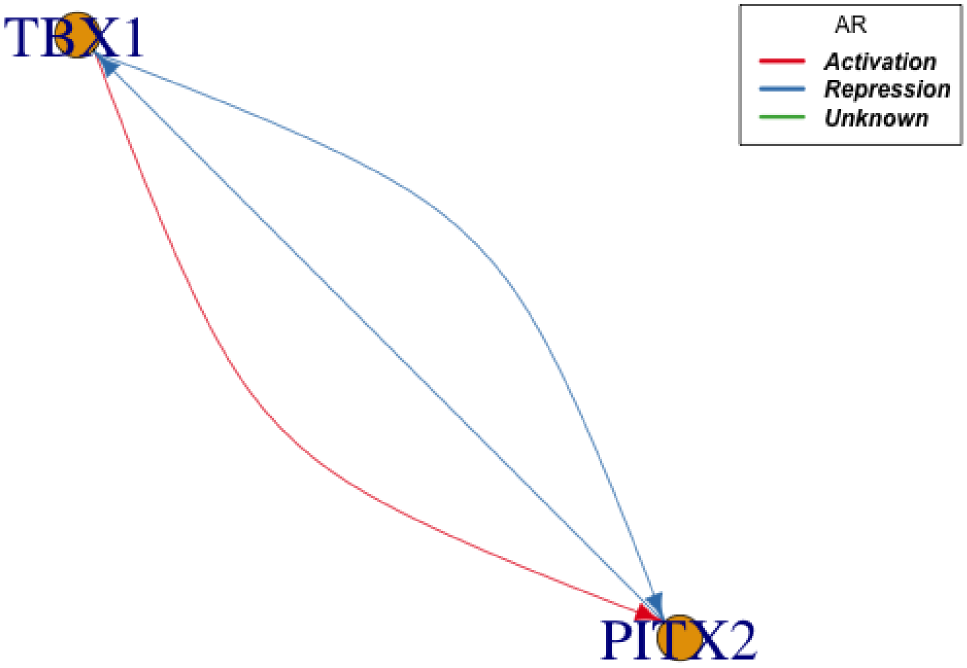

Mouse TRRUST

Clocked SR flip-flop

TCF4; ID3; ID2; NOTCH1; TCF3

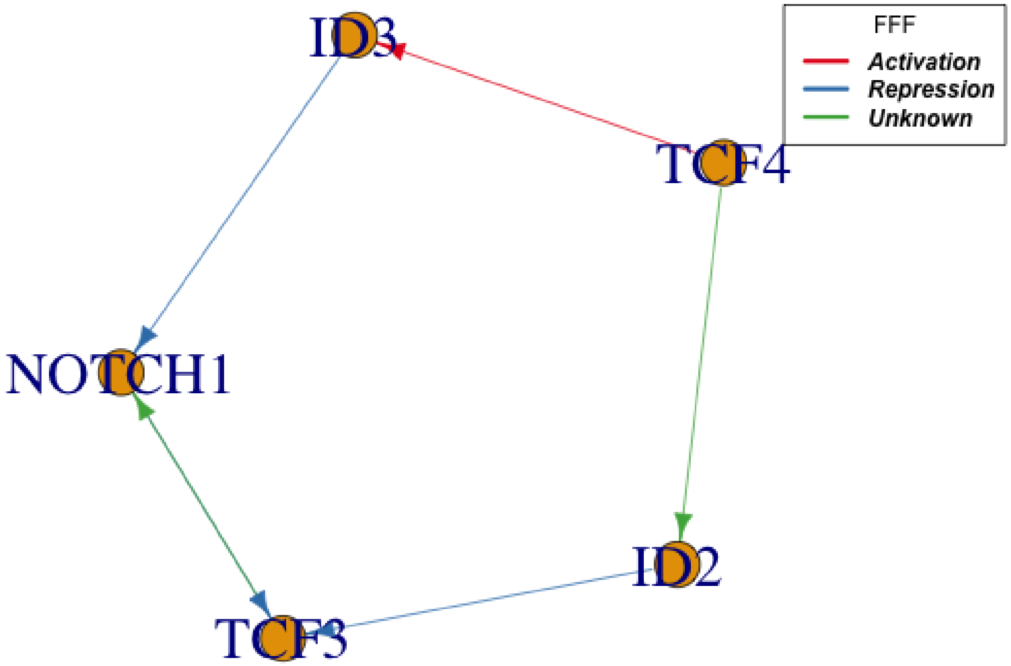

Mouse TRRUST

Clocked SR flip-flop

SIRT1; NR1I2; RELA; E2F1; TRP73

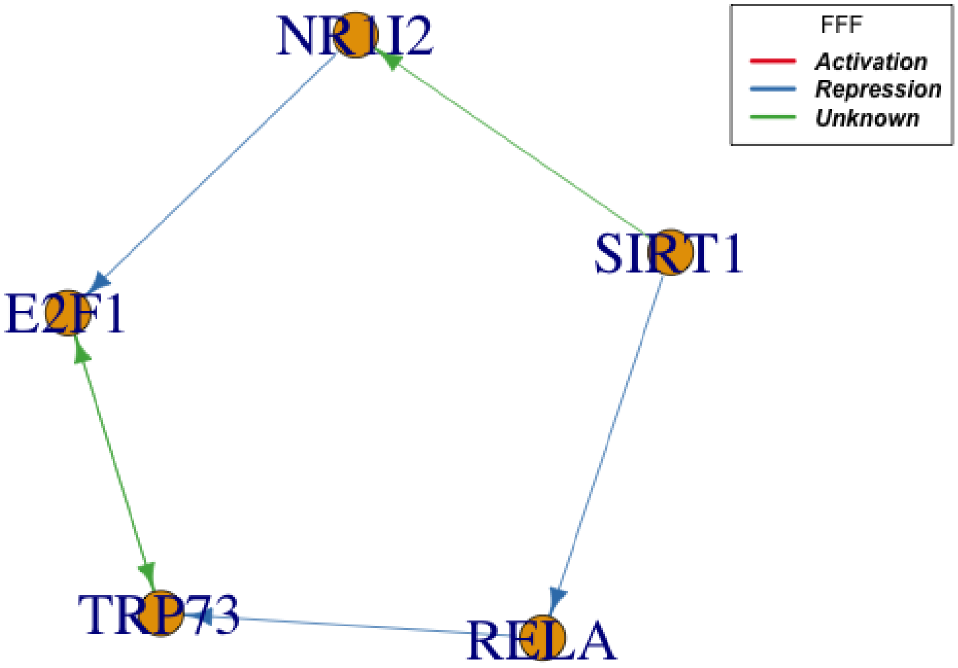

Mouse TRRUST

SR flip-flop

MXI1; MYC

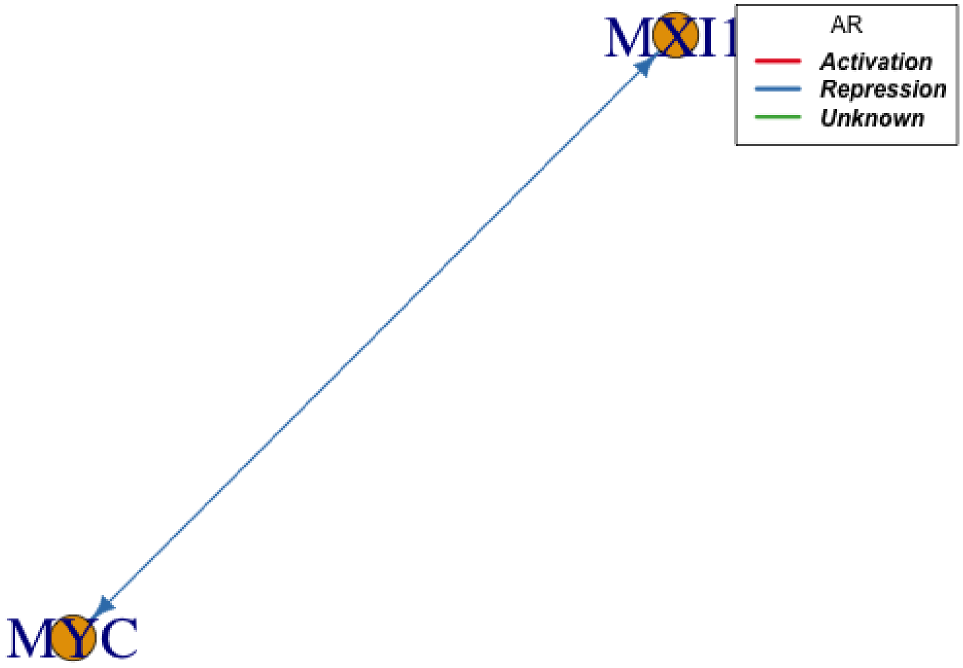

Mouse TRRUST

Clocked SR flip-flop

SP1; HMGA2; ETS1; NFKB1; FOXP3

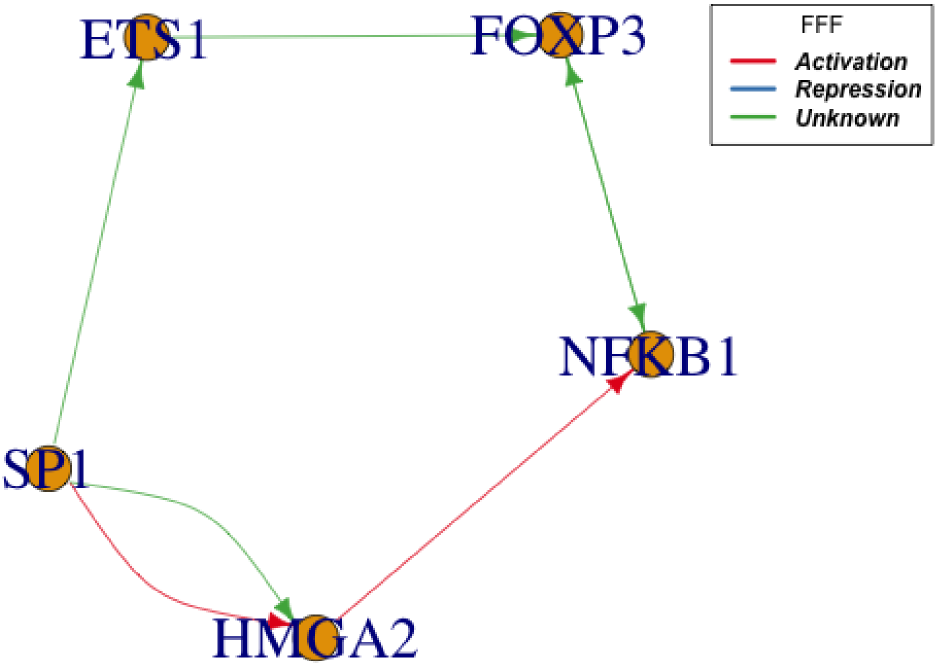

Mouse TRRUST

SR flip-flop

E2F1; PURA

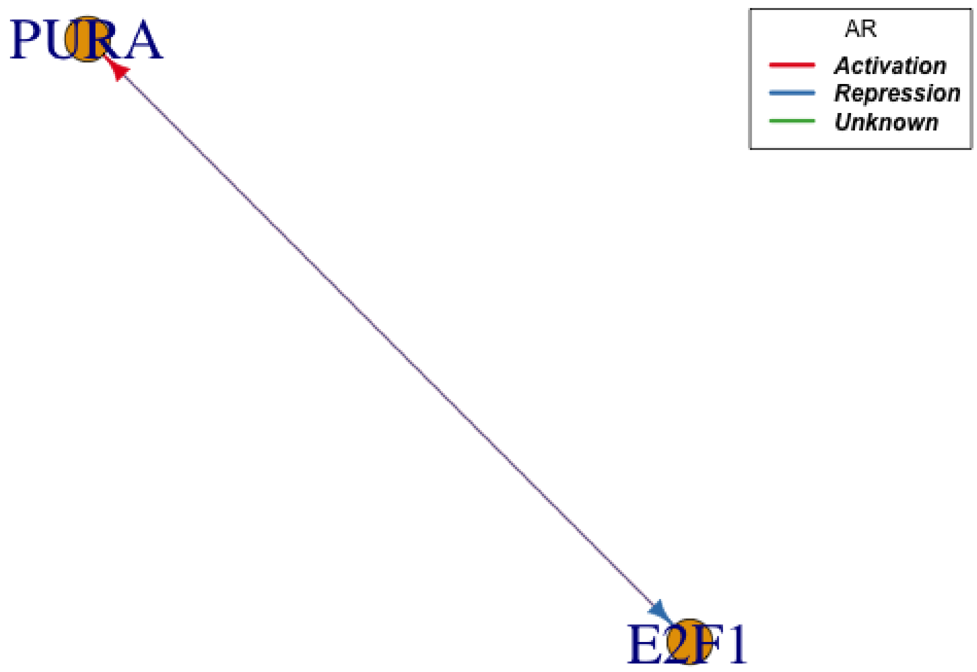

Mouse TRRUST

Clocked SR flip-flop

KAT5; SOX9; TRP53; BMI1; NANOG

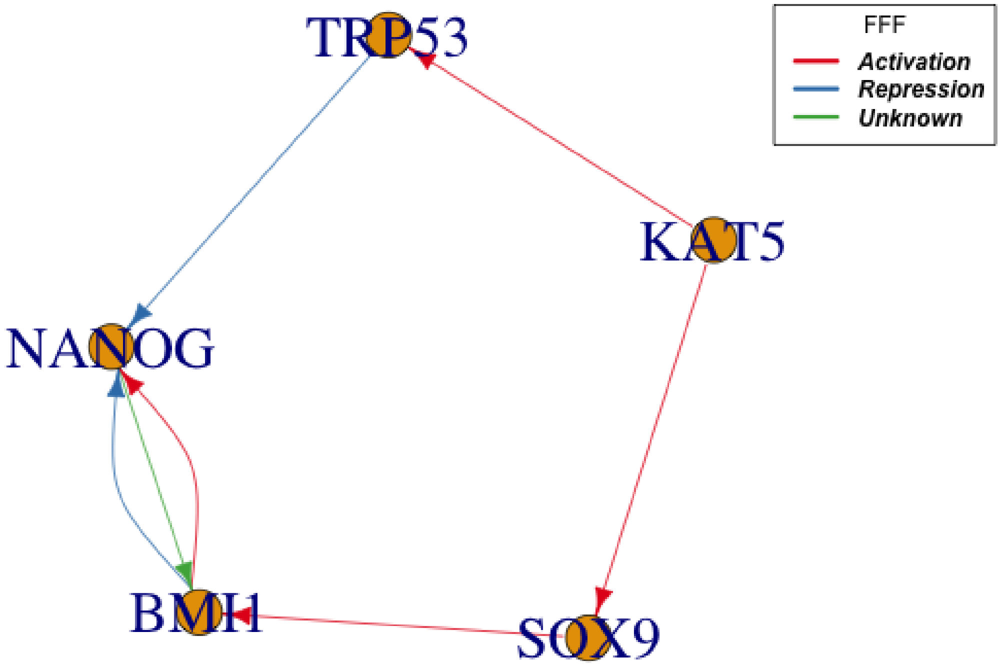

Mouse TRRUST

SR flip-flop

MYF6; MYOG

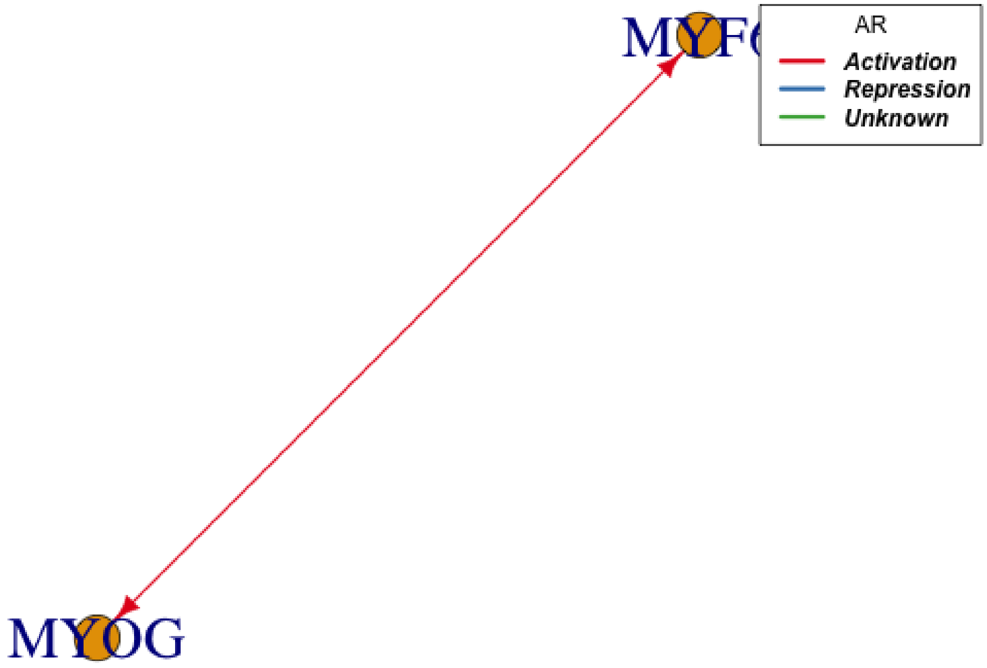

Mouse TRRUST

Clocked SR flip-flop

RARA; KLF5; PITX2; RUNX2; LEF1

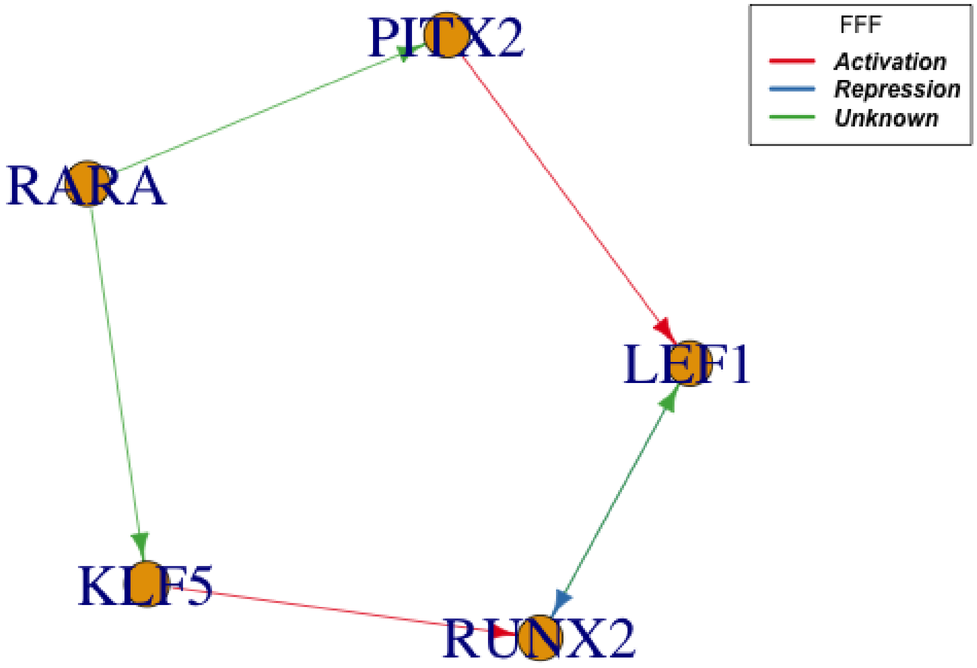

Mouse TRRUST

Clocked SR flip-flop

FOXO1; IKZF1; PPARG; MYC; TRP53

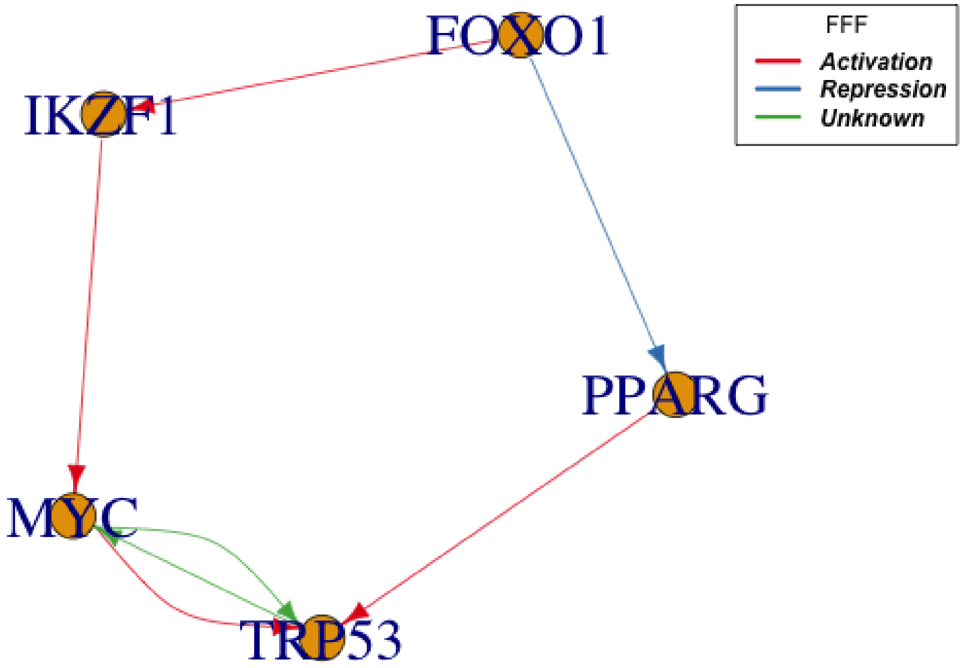

Mouse TRRUST

SR flip-flop

MYF5; MYOD1

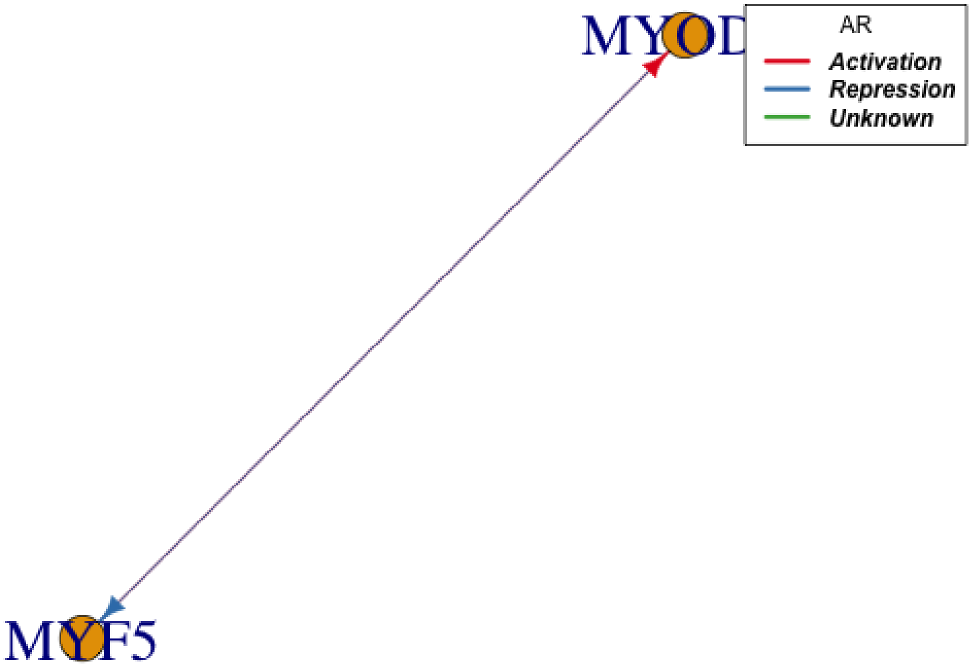

Mouse TRRUST

Clocked SR flip-flop

PELP1; NR3C1; NFKB1; RUNX2; LEF1

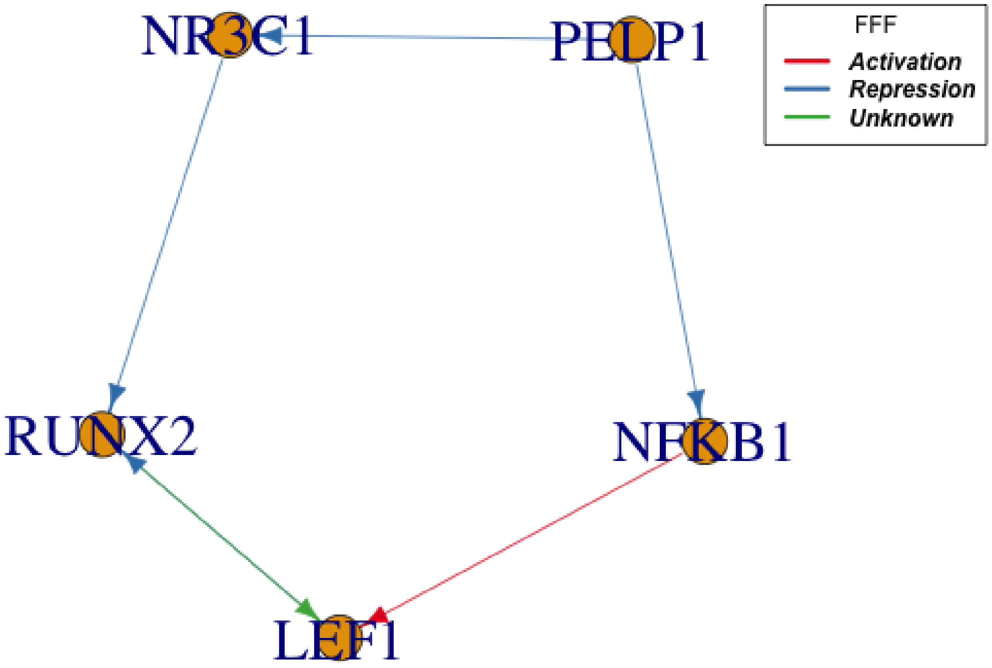

Mouse TRRUST

SR flip-flop

LEF1; RUNX2

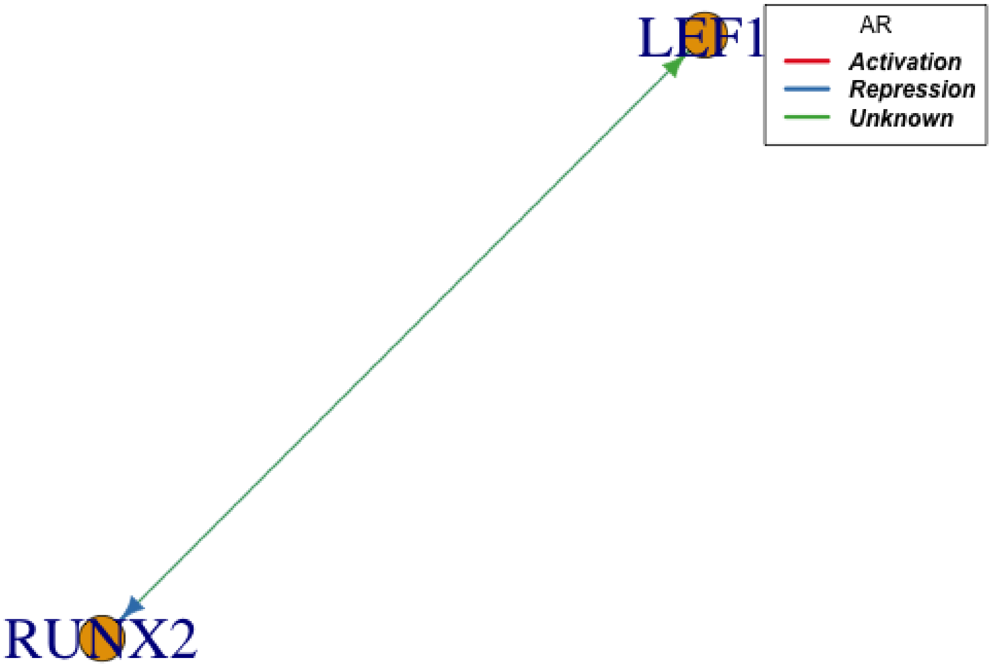

Mouse TRRUST

Clocked SR flip-flop

EGR1; JUN; SMAD7; NFKB1; NFKBIA

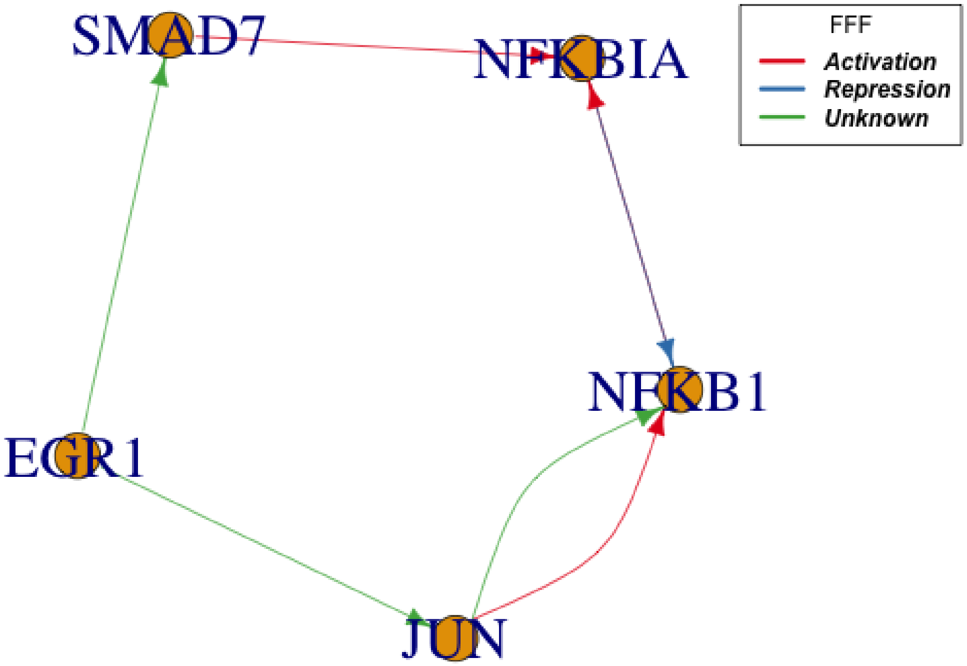

Mouse TRRUST

SR flip-flop

FOXH1; GSC

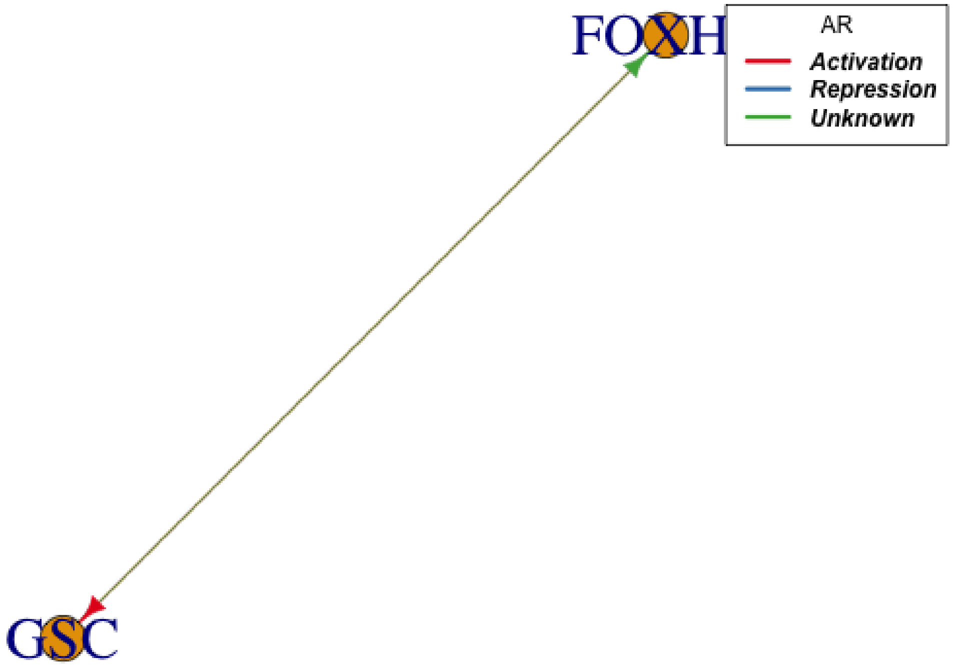

Mouse TRRUST

Clocked SR flip-flop

SMAD5; ID3; ID2; NOTCH1; TCF3

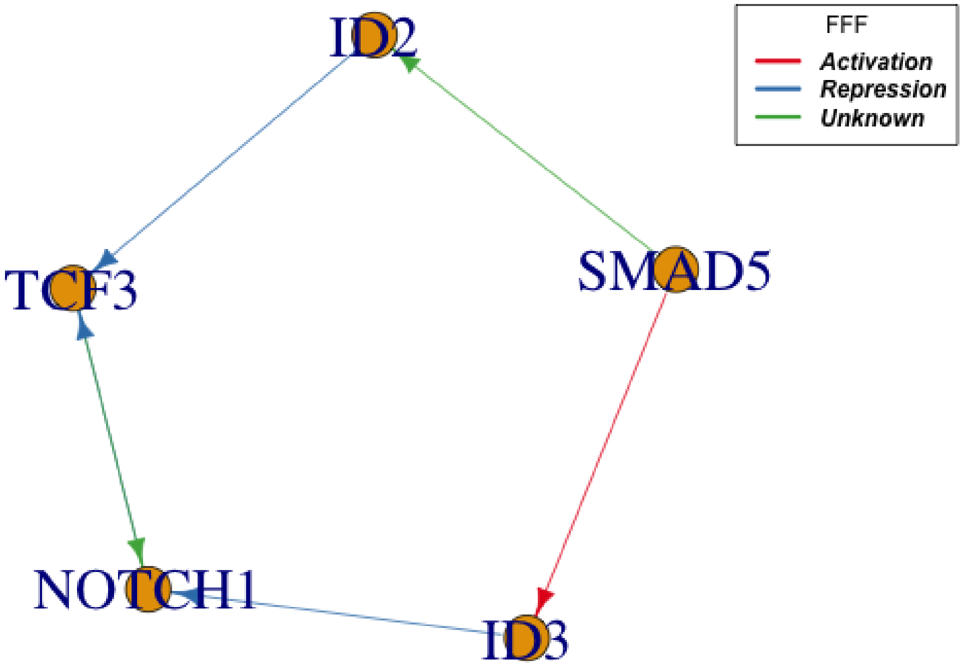

Mouse TRRUST

Clocked SR flip-flop

SOX4; CTNNB1; EZH2; E2F1; TRP53

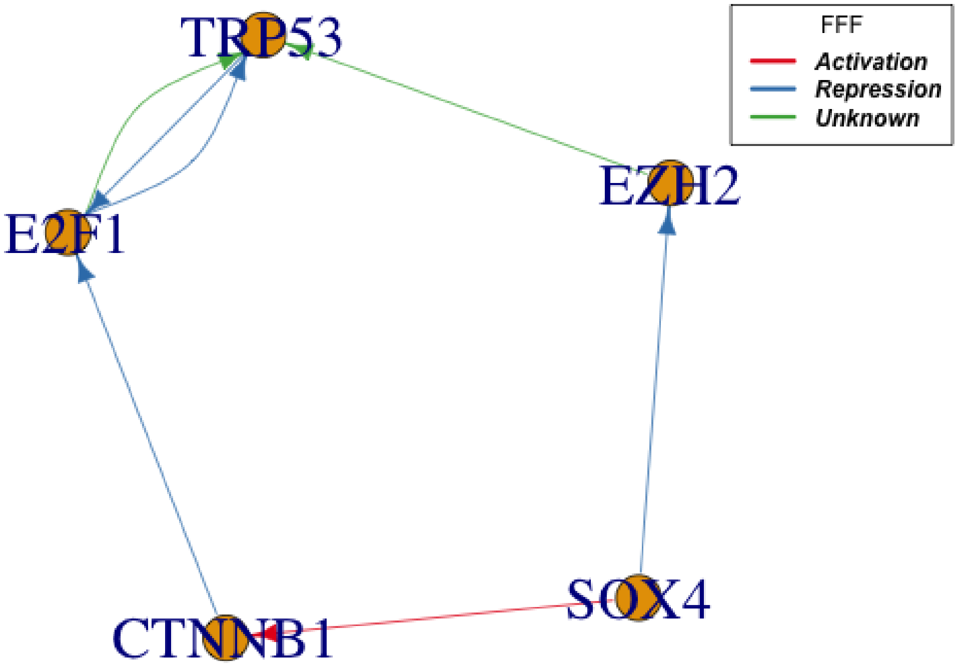

Mouse TRRUST

Clocked SR flip-flop

MYOD1; SMAD7; CREBBP; NFKBIA; NFKB1

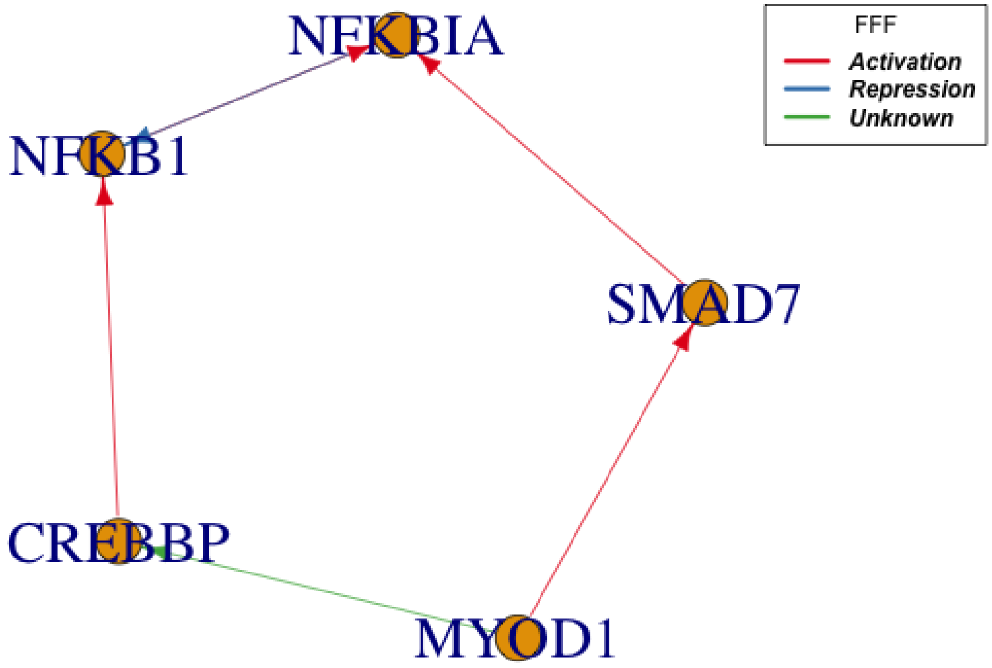

Mouse TRRUST

SR flip-flop

MYOD1; MYOG

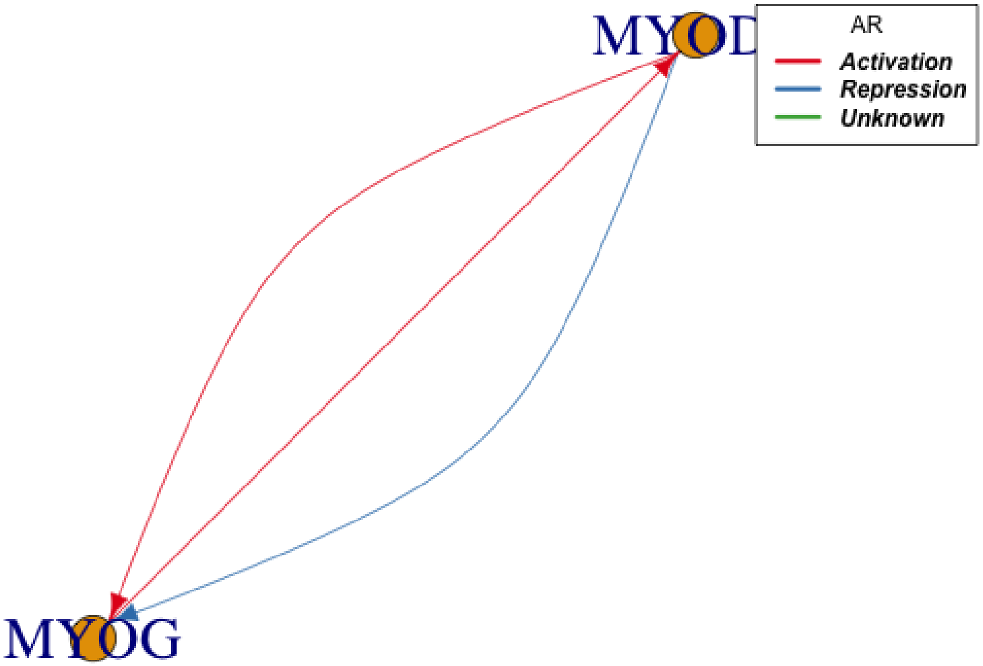

Mouse TRRUST

Clocked SR flip-flop

NR5A1; POU5F1; PBX1; MYOD1; MYOG

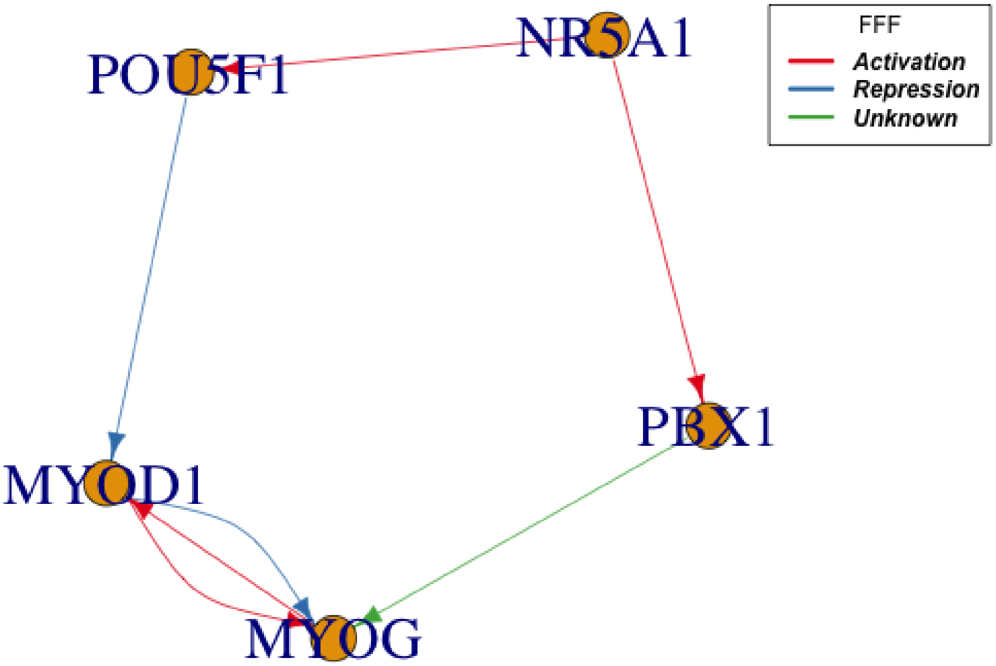

Yeast YTRP binding

Clocked SR flip-flop

SFP1; REB1; PDR1; TYE7; MSN2

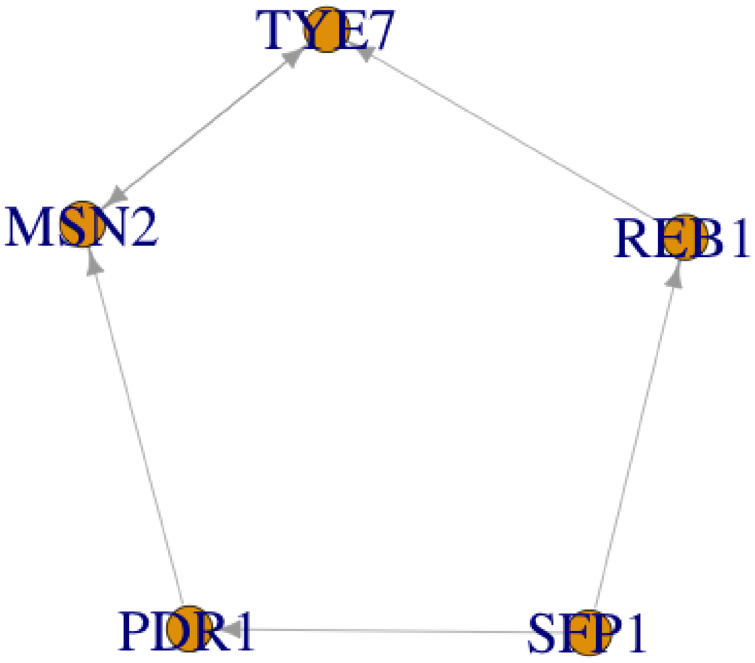

Yeast YTRP binding

SR flip-flop

ROX1; IXR1

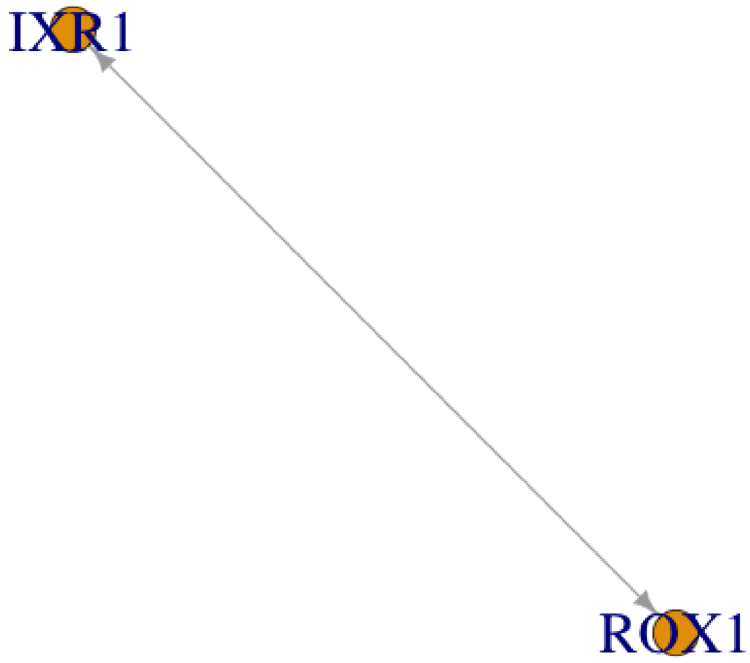

Yeast YTRP binding

Clocked SR flip-flop

TUP1; GLN3; GCR2; MSN2; UME6

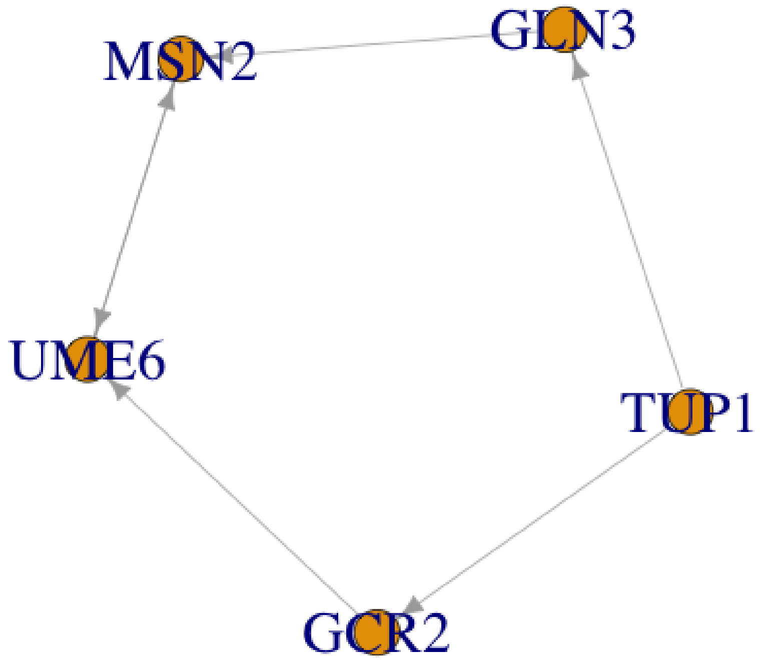

Yeast YTRP binding

Clocked SR flip-flop

SWI4; FKH2; IXR1; GZF3; GAT1

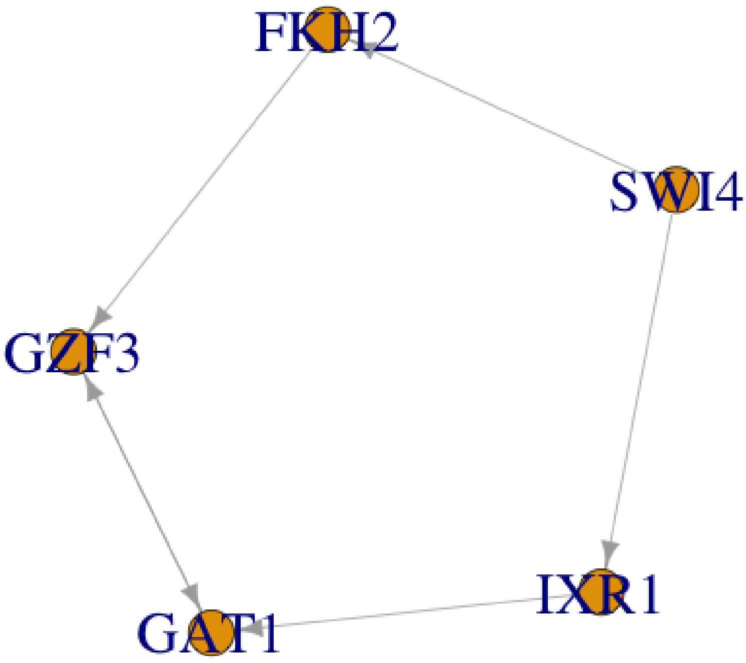

Yeast YTRP binding

Clocked SR flip-flop

PIP2; MSN2; FKH2; GAT1; GZF3

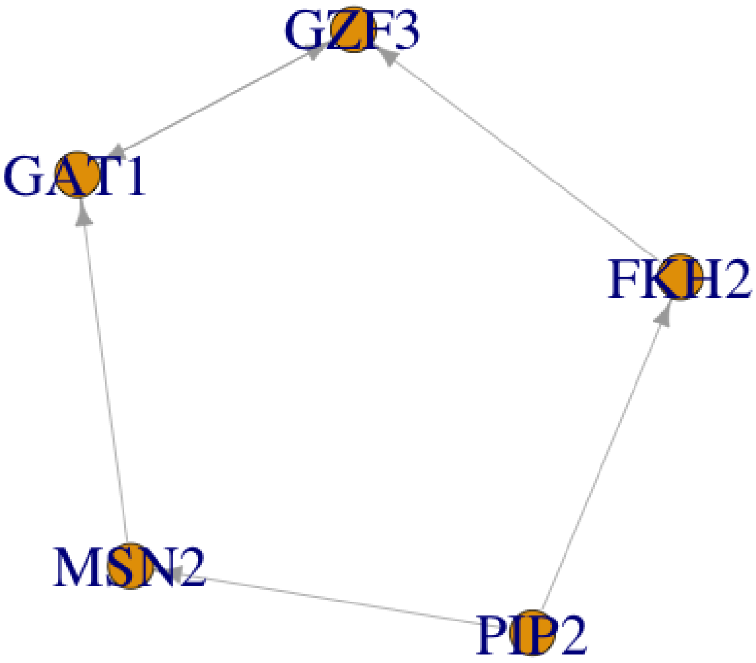

Yeast YTRP binding

Clocked SR flip-flop

STP1; YAP5; GCN4; MSN2; HMS1

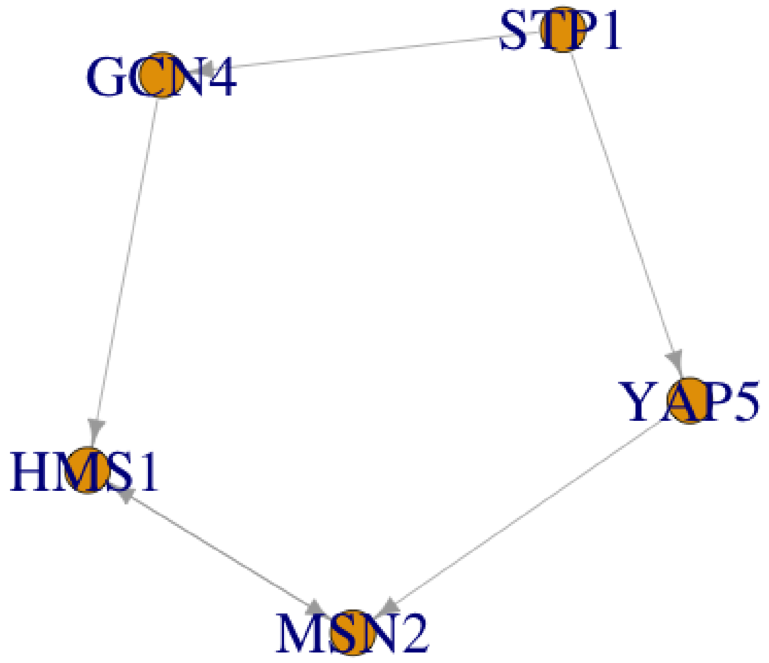

Yeast YTRP binding

Clocked SR flip-flop

TEC1; SUM1; YAP1; CBF1; TYE7

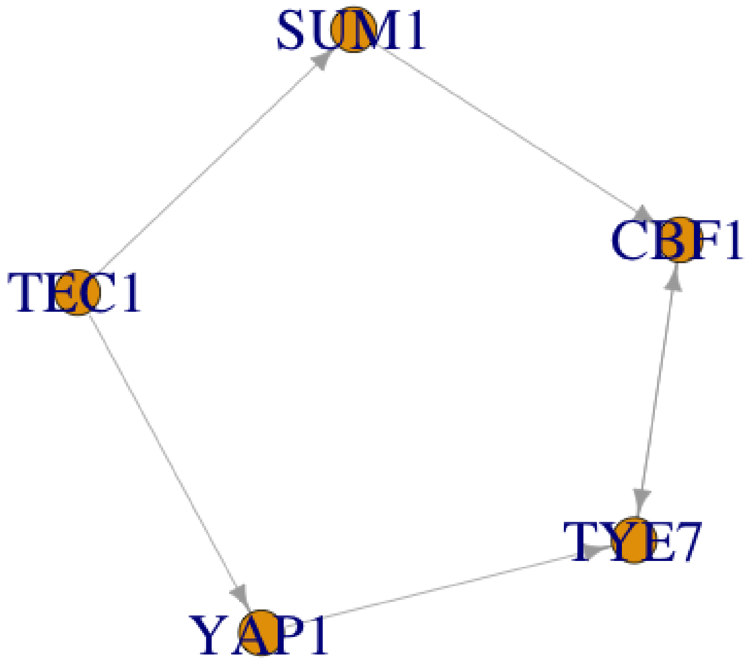

Yeast YTRP binding

Clocked SR flip-flop

ABF1; HAP1; MBP1; ROX1; MSN2

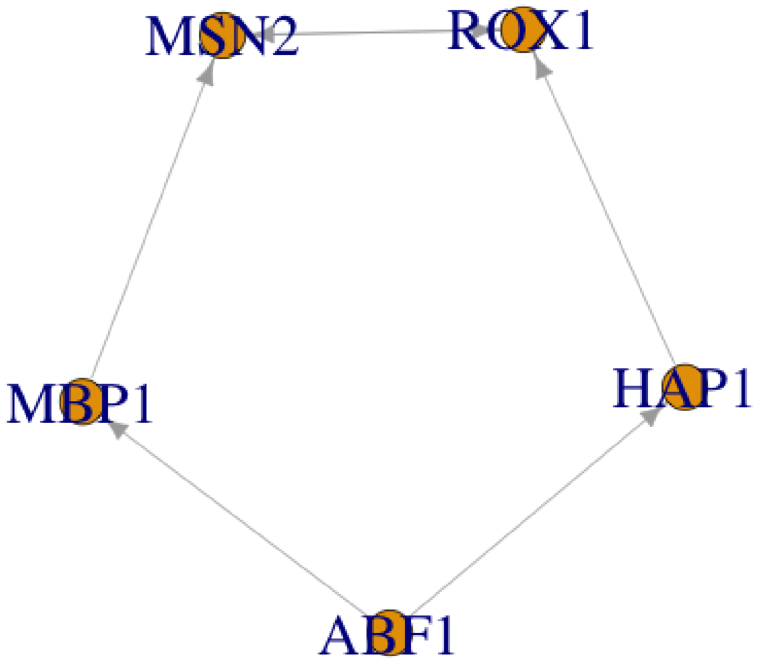

Yeast YTRP binding

Clocked SR flip-flop

STP1; YAP5; GCN4; MSN2; USV1

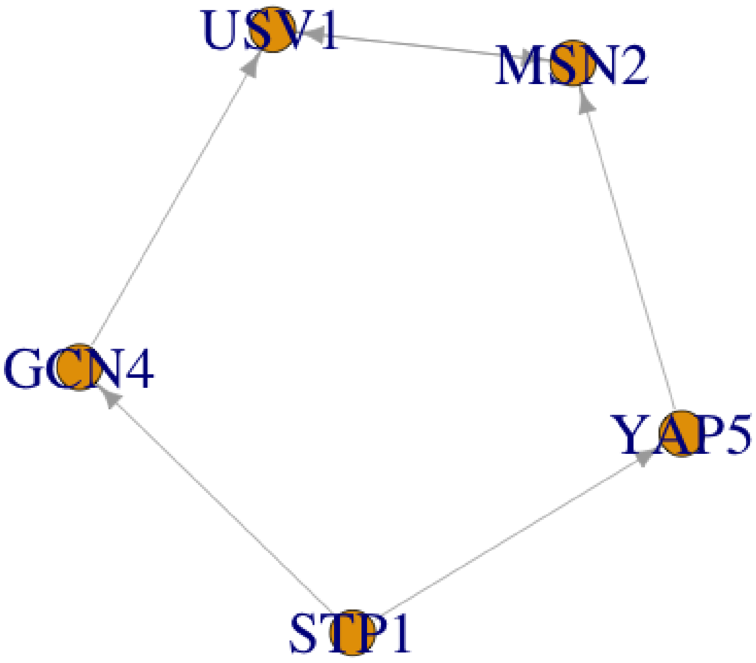

Yeast YTRP binding

Clocked SR flip-flop

STB1; HAP4; RPN4; UME6; YAP5

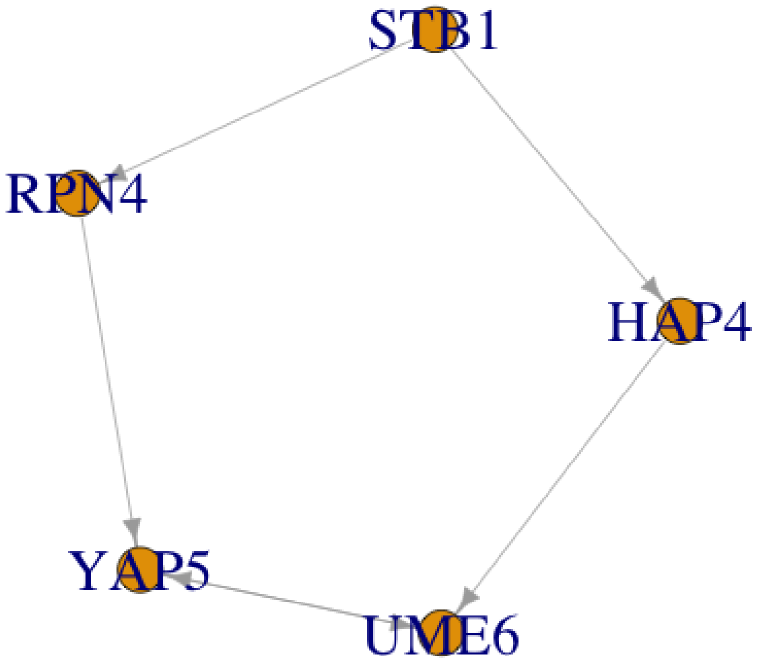

Yeast YTRP binding

Clocked SR flip-flop

SWI4; FKH2; PDR1; UME6; YAP5

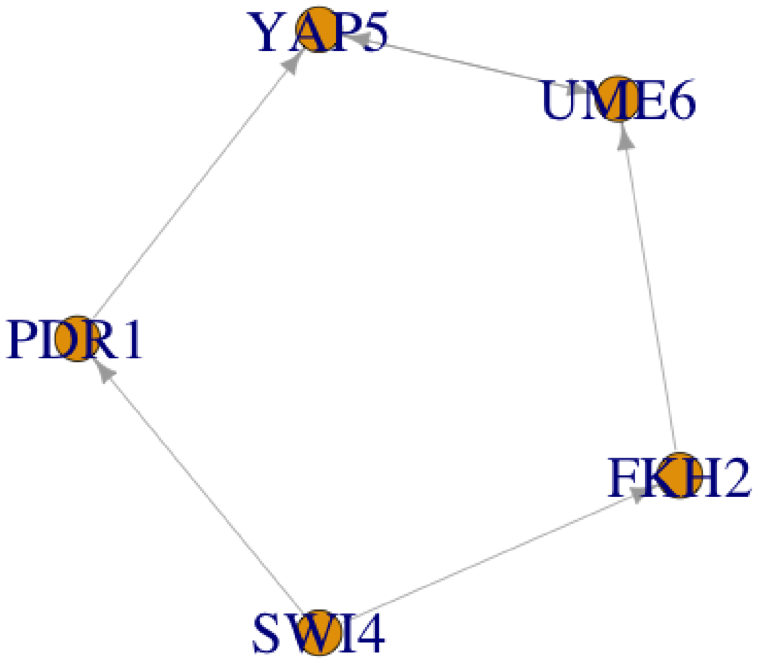

Yeast YTRP binding

SR flip-flop

MSN2; ROX1

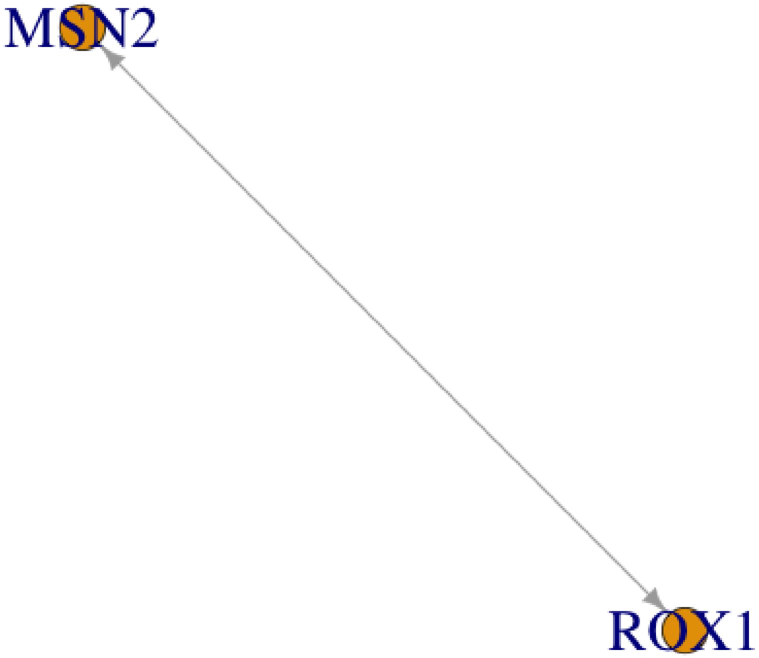

Yeast YTRP binding

Clocked SR flip-flop

GCN4; HSF1; FHL1; MSN2; ROX1

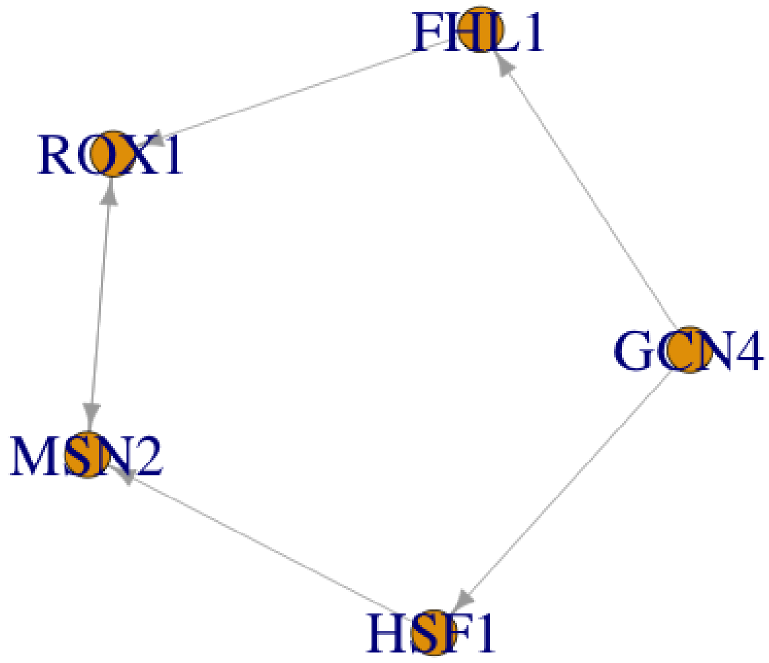

Yeast YTRP binding

Clocked SR flip-flop

GCN4; FLO8; HSF1; UME6; MSN2

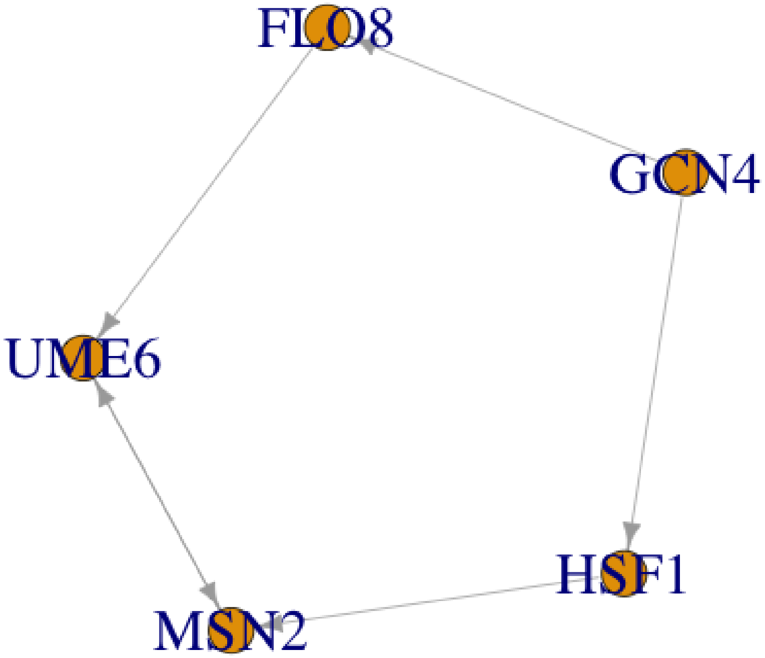

Yeast YTRP binding

SR flip-flop

MSN2; CIN5

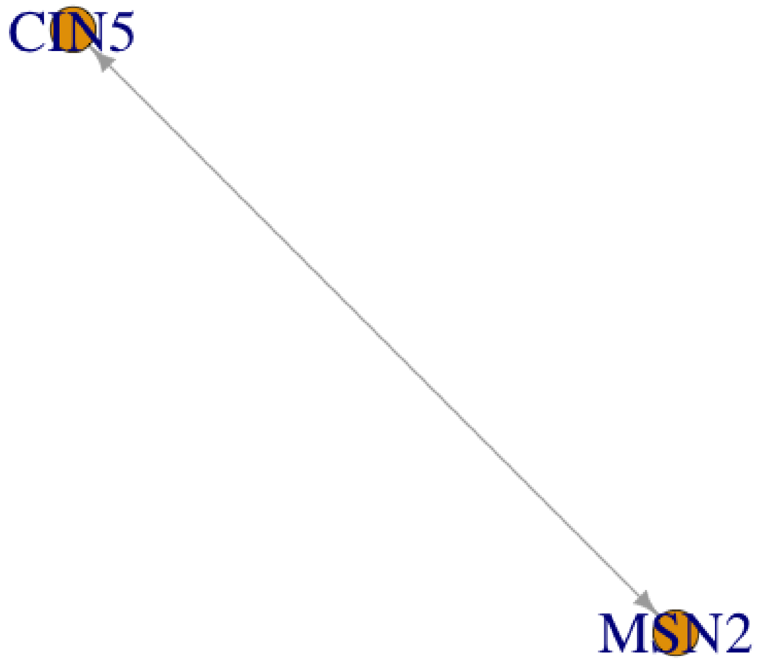

Yeast YTRP binding

Clocked SR flip-flop

MBP1; INO4; SWI4; ACE2; FKH2

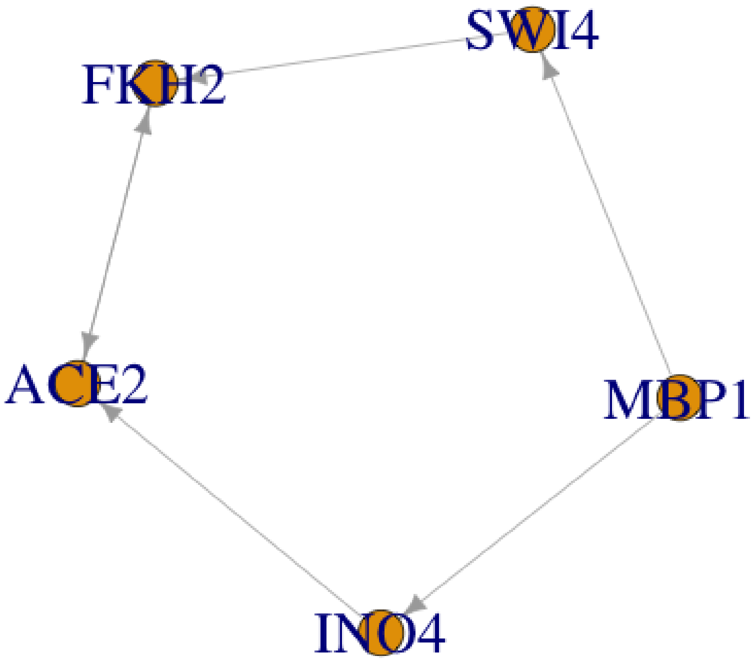

Yeast YTRP binding

Clocked SR flip-flop

PHO2; STB1; IXR1; RPN4; MSN4

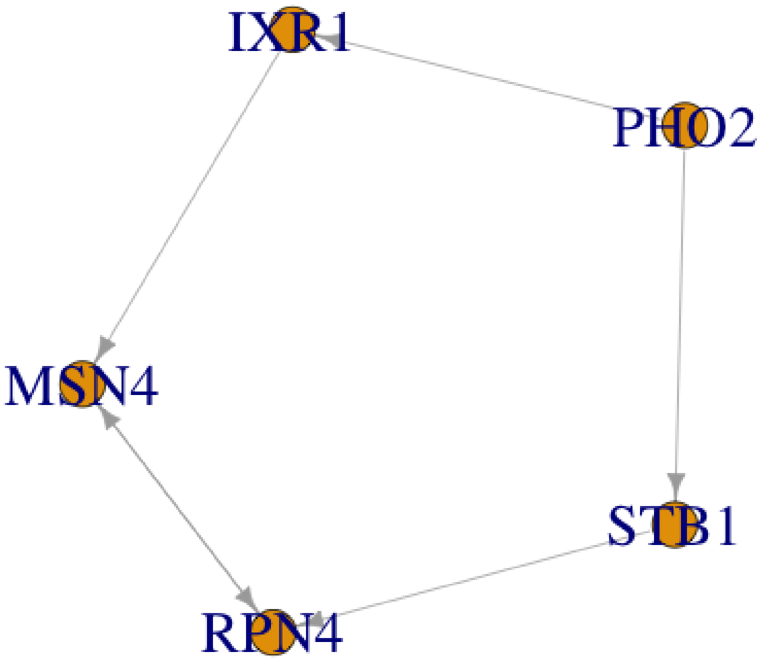

Yeast YTRP binding

SR flip-flop

YHP1; STE12

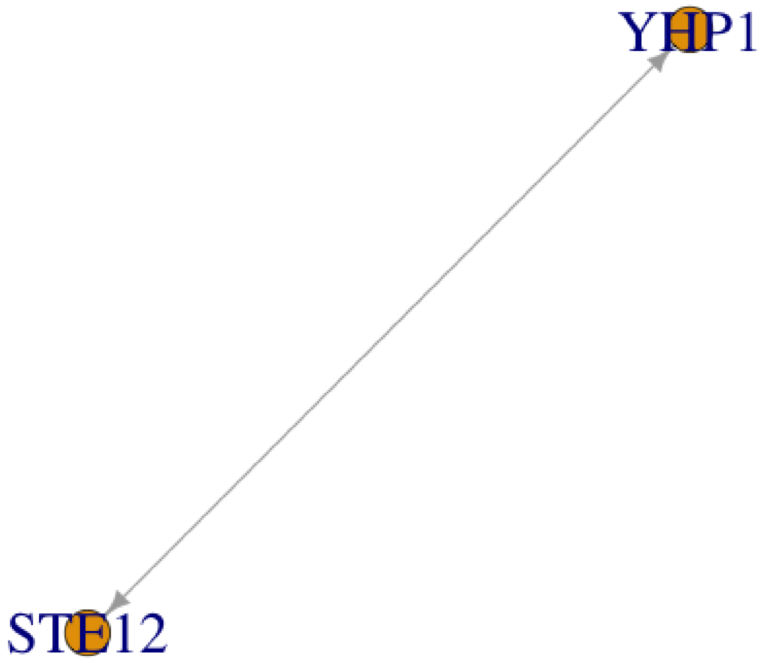

Yeast YTRP binding

Clocked SR flip-flop

ABF1; GTS1; SWI5; MSN2; TEC1

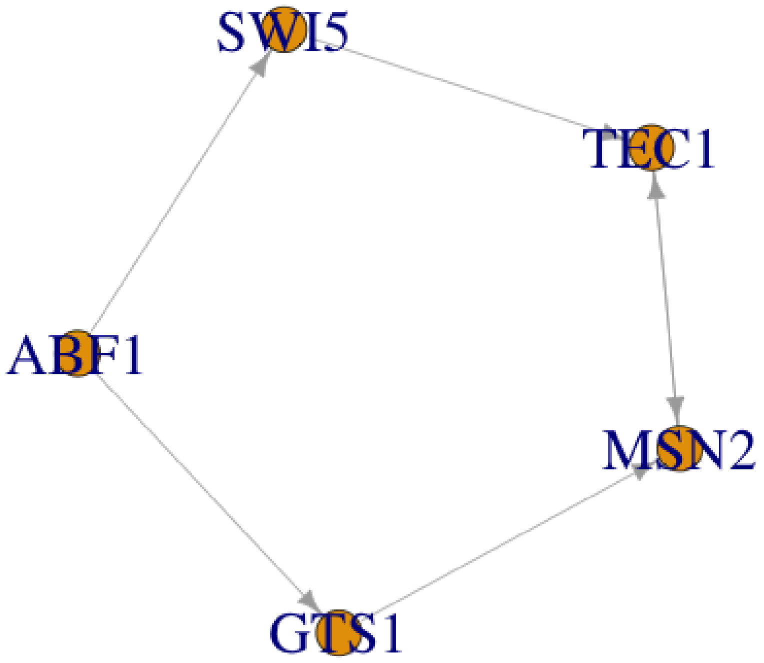

Yeast YTRP binding

Clocked SR flip-flop

FKH1; SWI5; SKO1; IXR1; ROX1

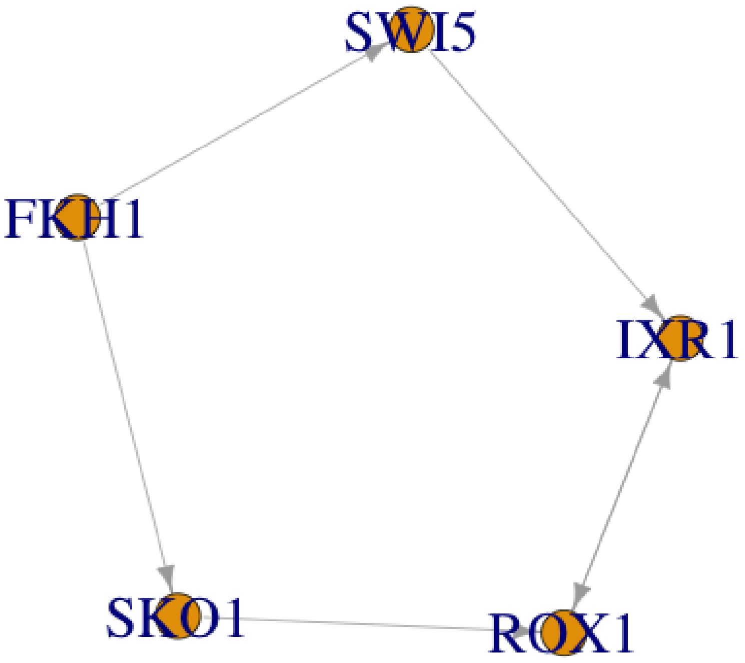

Yeast YTRP binding

Clocked SR flip-flop

STP1; GCN4; YAP1; CBF1; TYE7

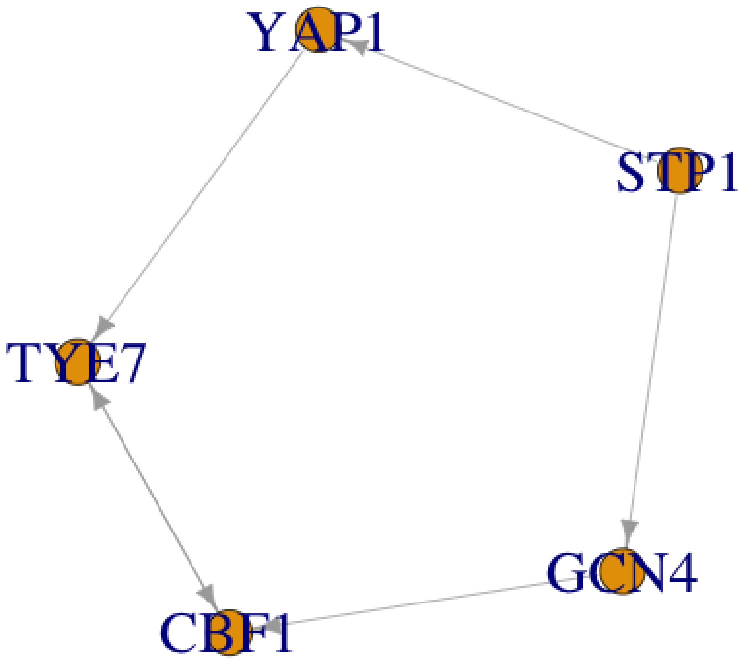

Yeast YTRP binding

Clocked SR flip-flop

STP1; GCN4; YAP1; HMS1; MSN2

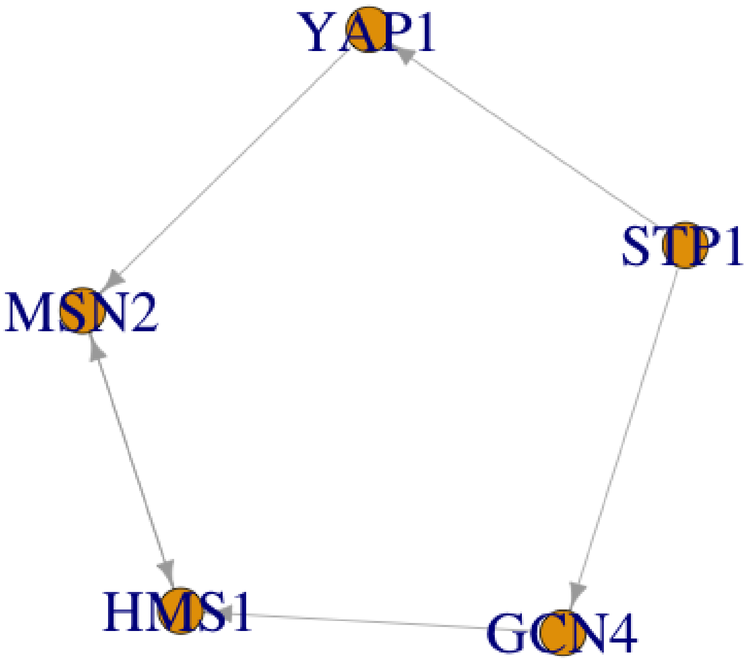

Yeast YTRP regulatory

SR flip-flop

TYE7; MSN2

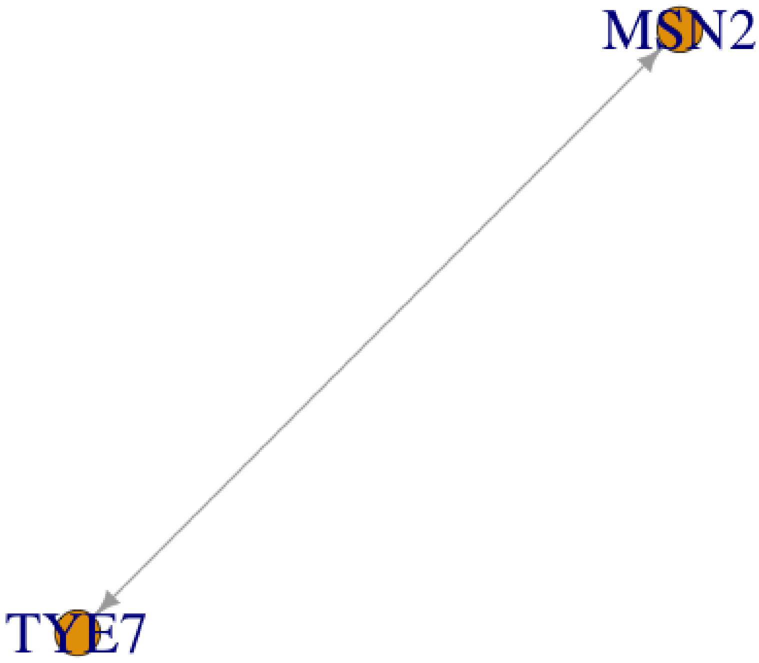

Ecoli

AR

Regulators:

Fiber: ARGA, ARGCBH, ARGE, ARGF, ARGI, ARGR, ARTJ, ARTPIQM, LYSO

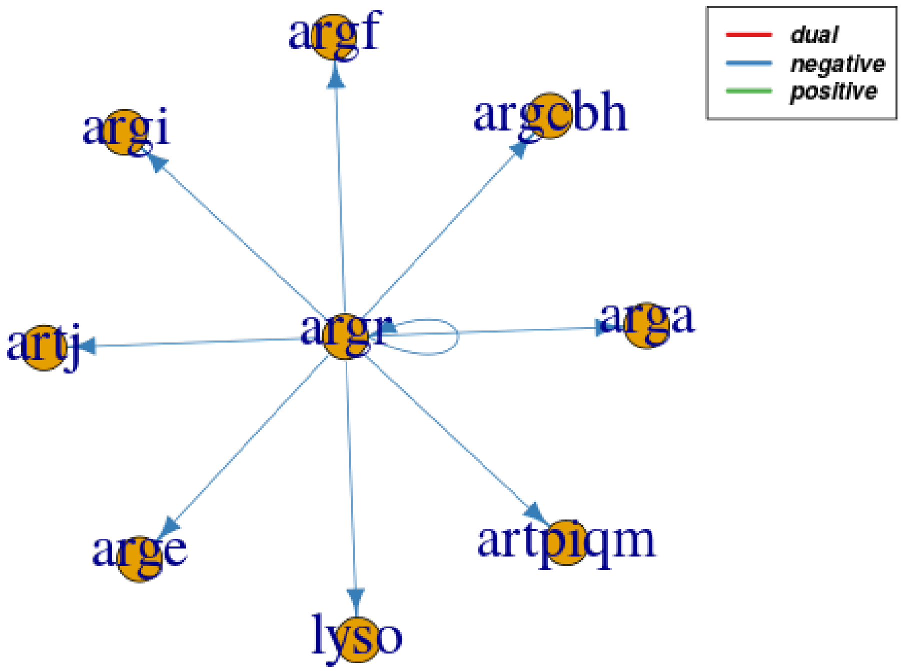

Ecoli

AR

Regulators:

Fiber: TTDABT, TTDR

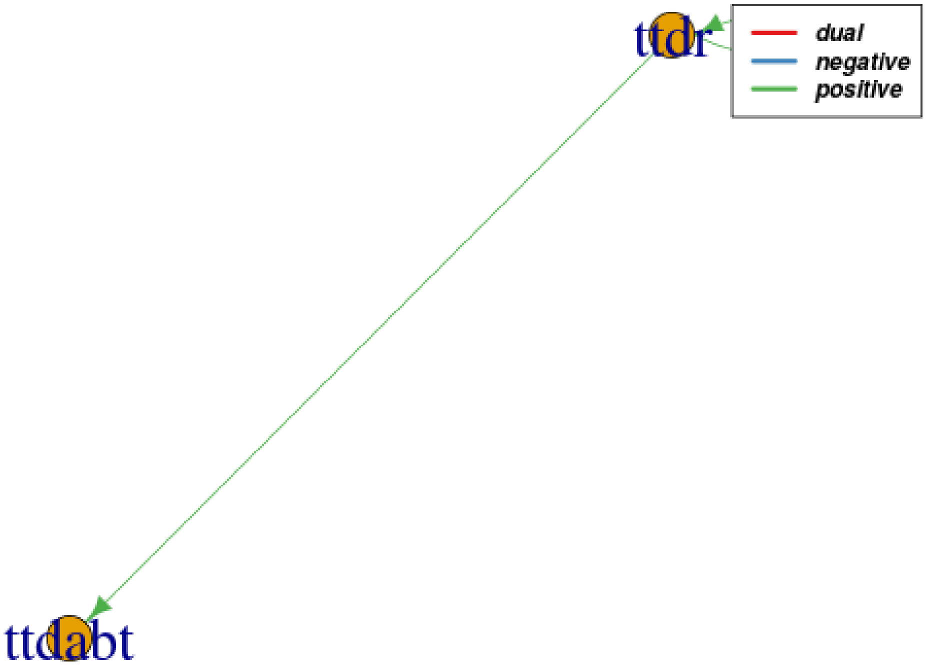

Ecoli

AR

Regulators:

Fiber: MNGAB, MNGR

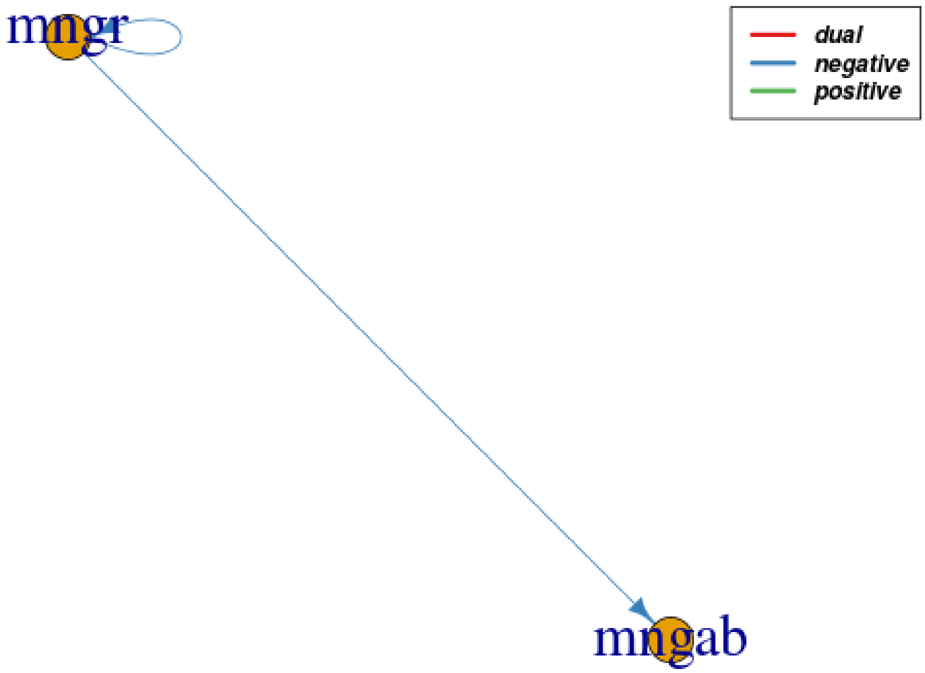

Ecoli

AR

Regulators:

Fiber: CHO, DINB-YAFNOP, DIND, DING-YBIB, DINQ, FTSK, HOKE, INSK, LEXA, POLB, PTRA-RECBD, RECAX, RECN, RECQ, RPSU-DNAG-RPOD, RUVAB, SYME, TISB, UMUDC, UVRB, UVRD, UVRY, YBFE, YBGA-PHR, YDJM, YEBG

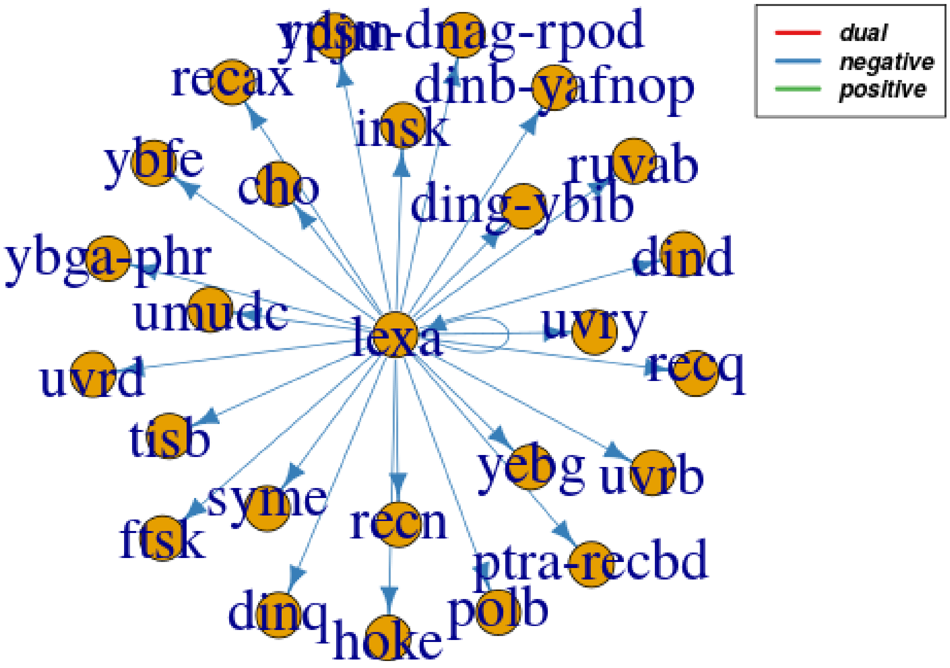

Ecoli

AR

Regulators:

Fiber: ERPA, ISCR, RNLAB

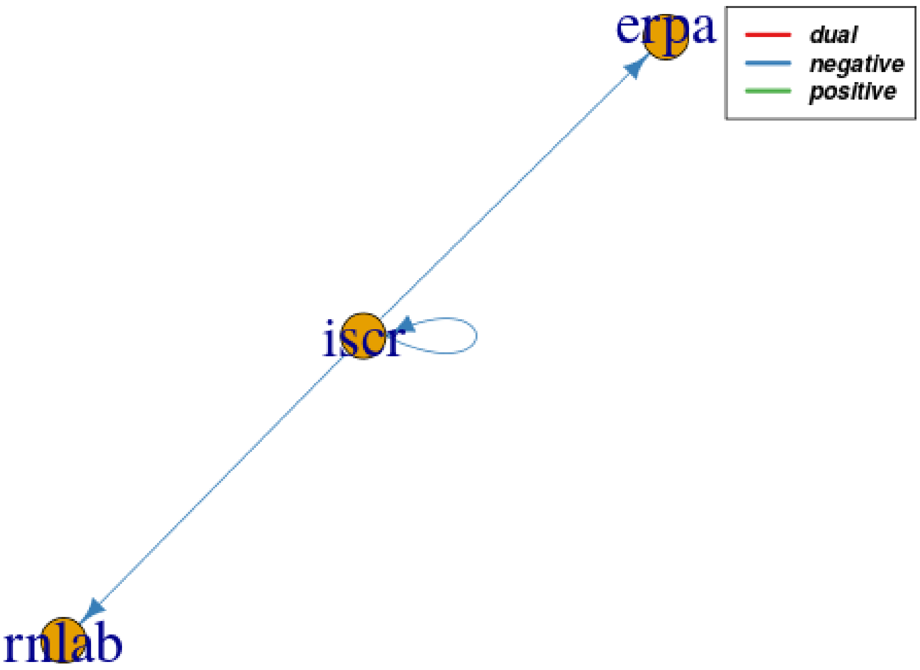

Ecoli

FFF

Regulators: HNS

Fiber: EVGA, NHAR

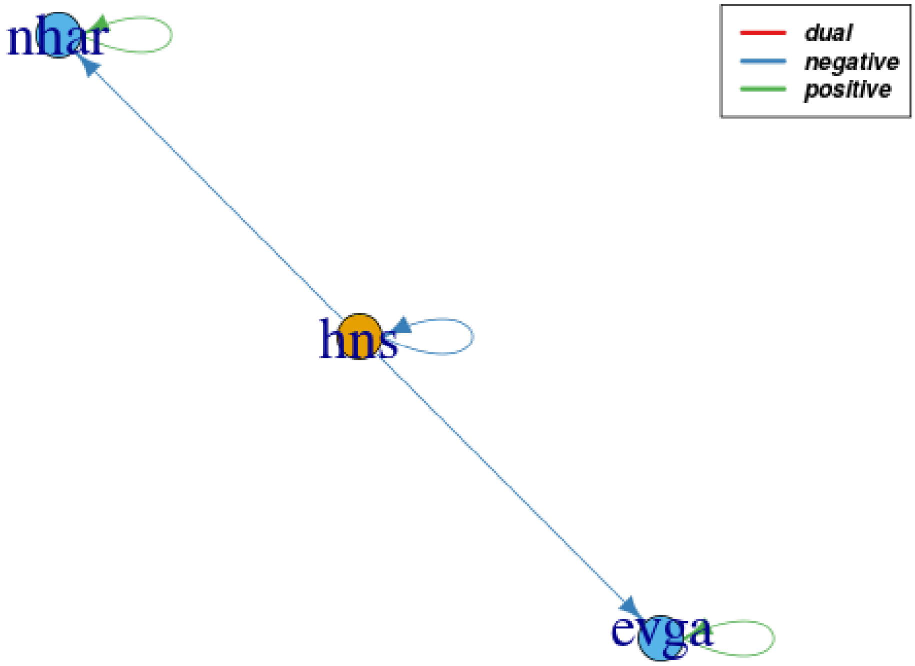

Ecoli

FFF

Regulators: CRP

Fiber: FUCAO, FUCR, ZRAR

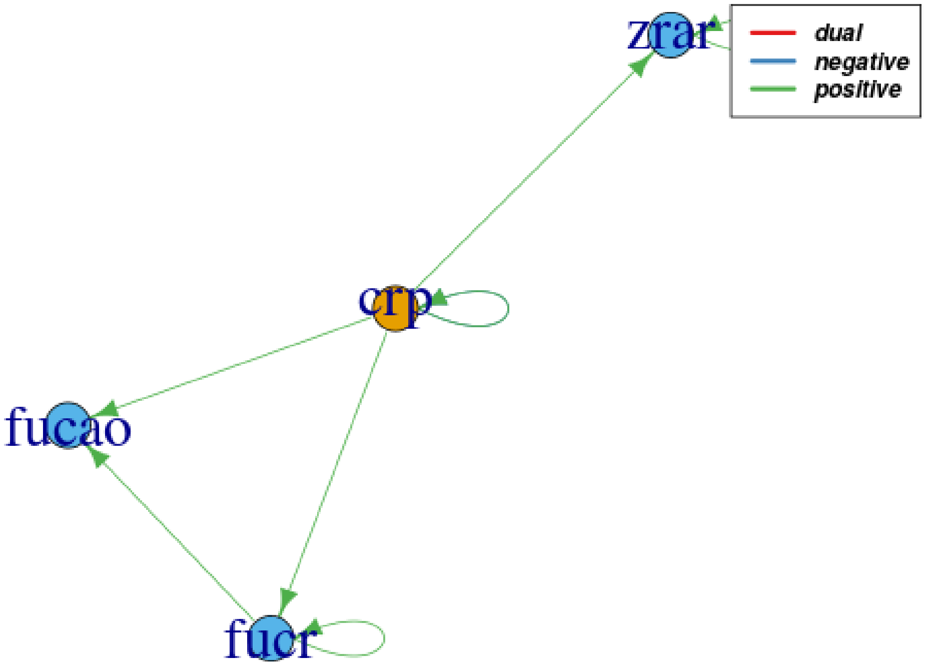

Ecoli

FFF

Regulators: FADR

Fiber: ACCB, ICLR

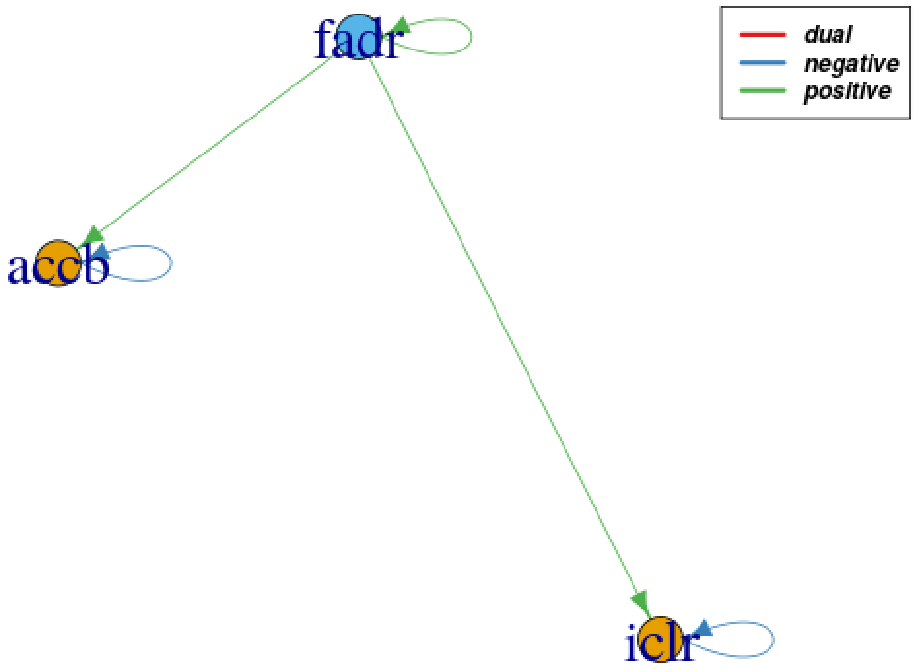

Ecoli

FFF

Regulators: CRP

Fiber: MALI, MLC

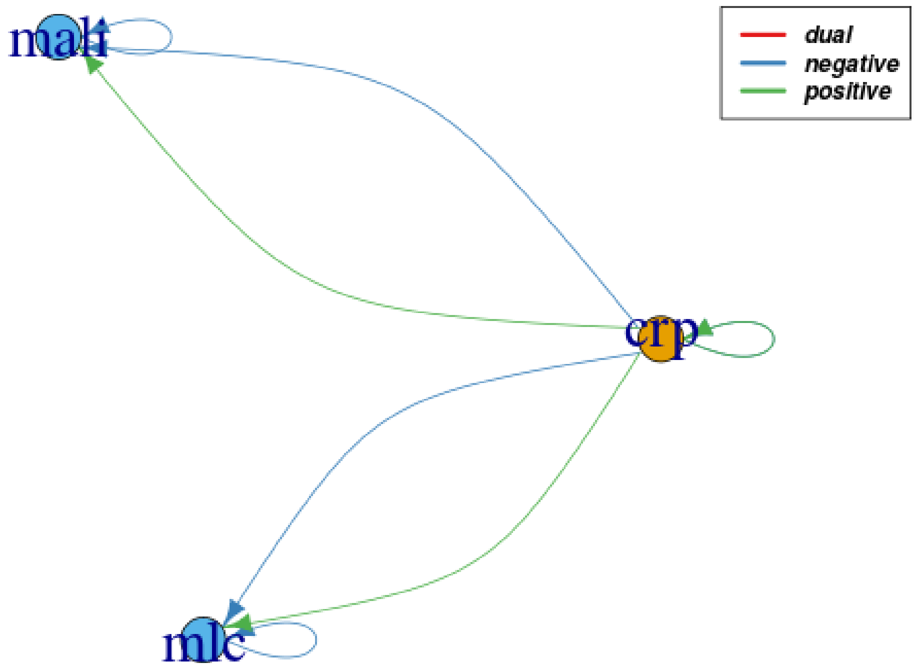

Ecoli

n = 2

Regulators: CRP

Fiber: RHAR, RHAS

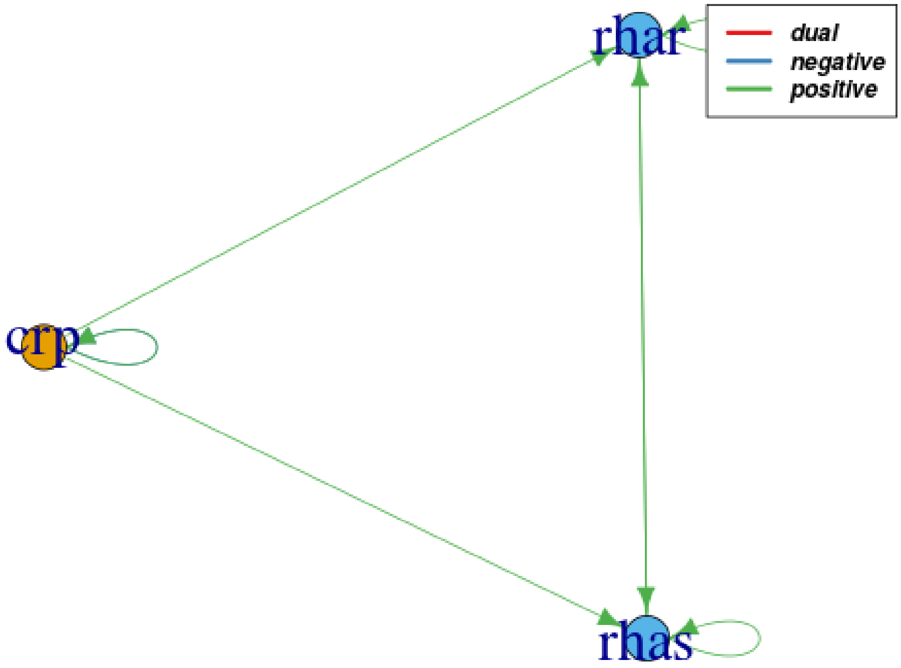

Human TRRUST

Fibonacci

Regulators: MYC

Fiber: DDX18, MTDH, RCC1, SART3, SHMT1, TFAM, TLX1, TOP1MT

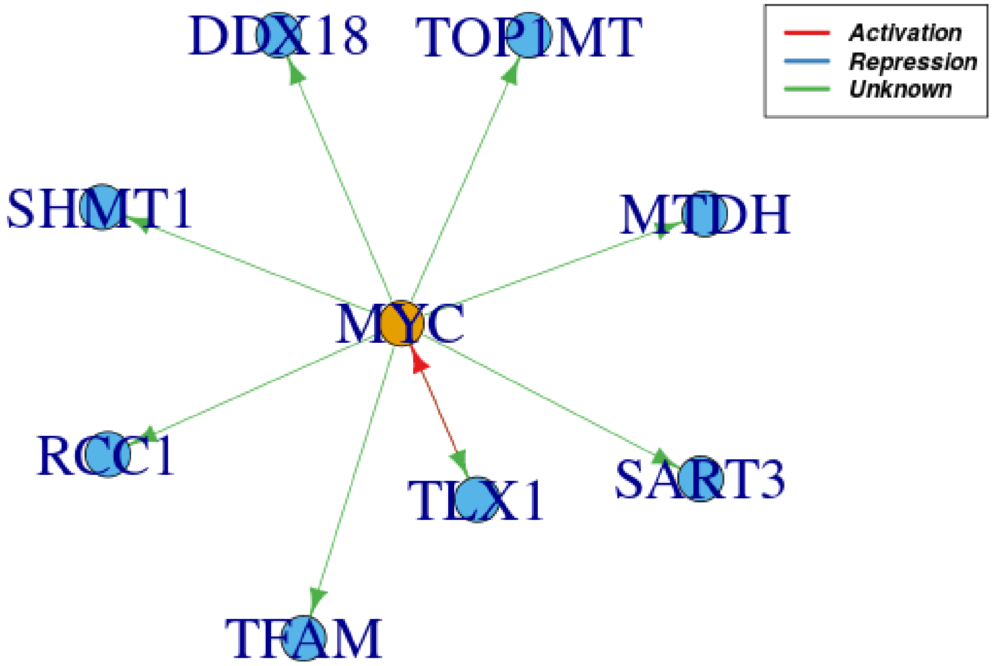

Human TRRUST

Fibonacci

Regulators: FOS, JUN, SP1

Fiber: CREM, EZR

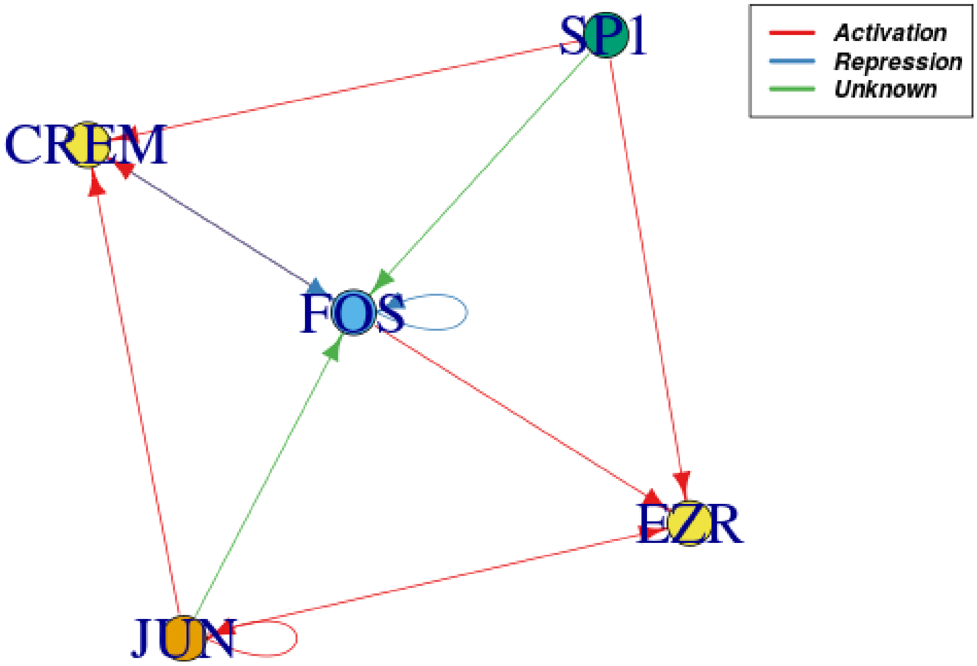

Human TRRUST

Fibonacci

Regulators: WT1

Fiber: CIAO1, PAX2, UBE2I

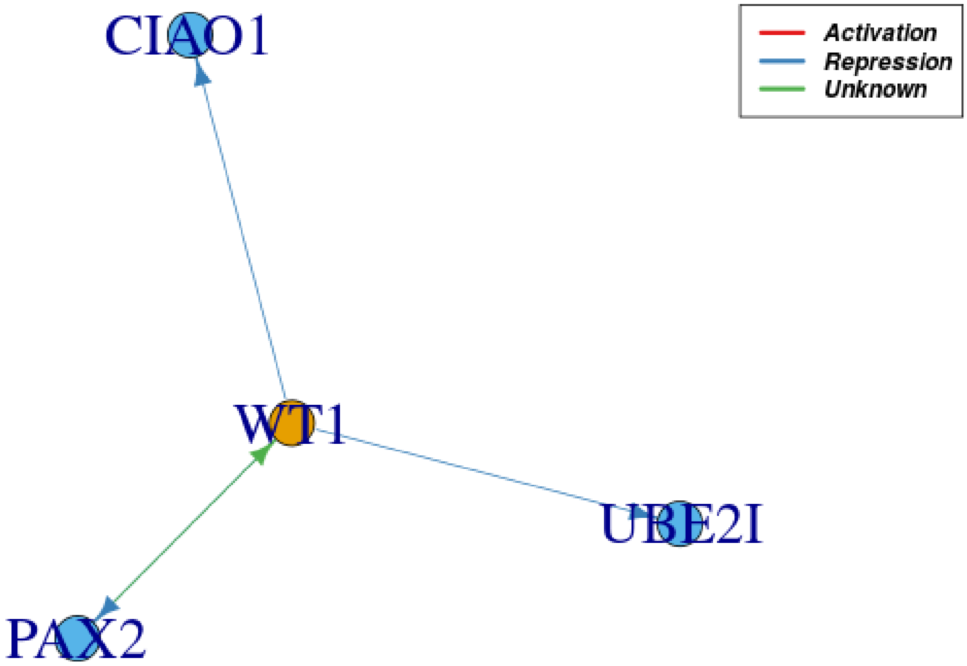

Micobacterium tuberculosis

AR

Regulators:

Fiber: RV0019C, RV0049, RV0104, RV0110, RV0134, RV0256C, RV0408, RV0409, RV0428C, RV0429C, RV0453, RV0457C, RV0458, RV0462, RV0475, RV0476, RV0477, RV0478, RV0658C, RV0680C, RV0681, RV0727C, RV0851C, RV0852, RV0904C, RV0905, RV0906, RV0950C, RV0993, RV0996, RV1157C, RV1158C, RV1159, RV1200, RV1212C, RV1213, RV1230C, RV1248C, RV1255C, RV1256C, RV1405C, RV1406, RV1407, RV1474C, RV1475C, RV1538C, RV1552, RV1553, RV1554, RV1566C, RV1579C, RV1580C, RV1581C, RV1719, RV1759C, RV1760, RV1779C, RV1780, RV1976C, RV1977, RV2077C, RV2078, RV2124C, RV2238C, RV2239C, RV2248, RV2293C, RV2294, RV2342, RV2406C, RV2407, RV2408, RV2436, RV2452C, RV2453C, RV2454C, RV2455C, RV2857C, RV2858C, RV2859C, RV2860C, RV2918C, RV3028C, RV3029C, RV3031, RV3063, RV3113, RV3114, RV3295, RV3302C, RV3316, RV3318, RV3319, RV3323C, RV3324A, RV3324C, RV3454, RV3635, RV3645, RV3650, RV3652, RV3676, RV3690, RV3696C, RV3724A, RV3724B, RV3729, RV3843C, RV3844, RV3856C, RV3857C, RV3893C

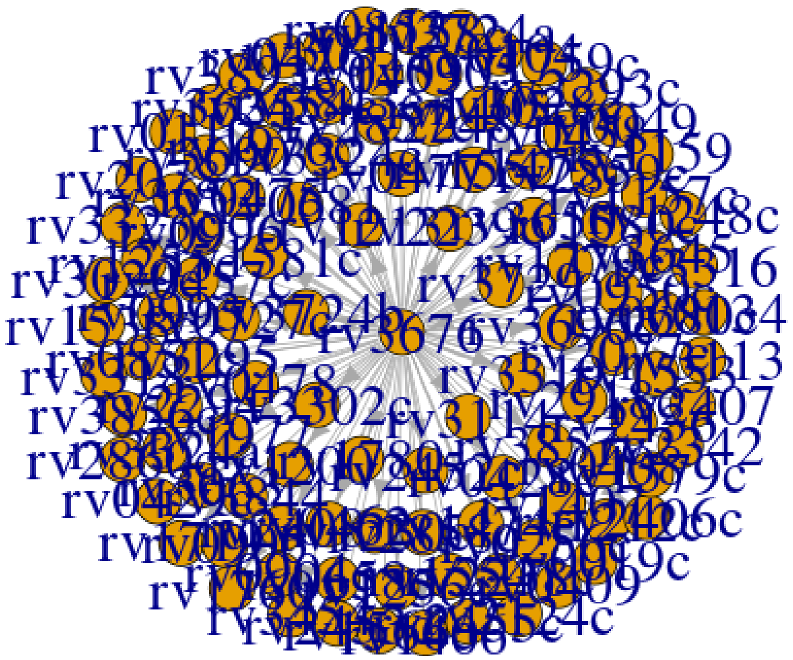

Micobacterium tuberculosis

AR

Regulators:

Fiber: RV0470A, RV0470C, RV0471C, RV1253, RV1261C, RV1262C, RV1263, RV1393C, RV1394C, RV1395, RV2320C, RV2321C, RV2322C, RV2323C, RV2727C, RV2728C, RV2781C, RV3407, RV3408, RV3482C, RV3816C, RV3817

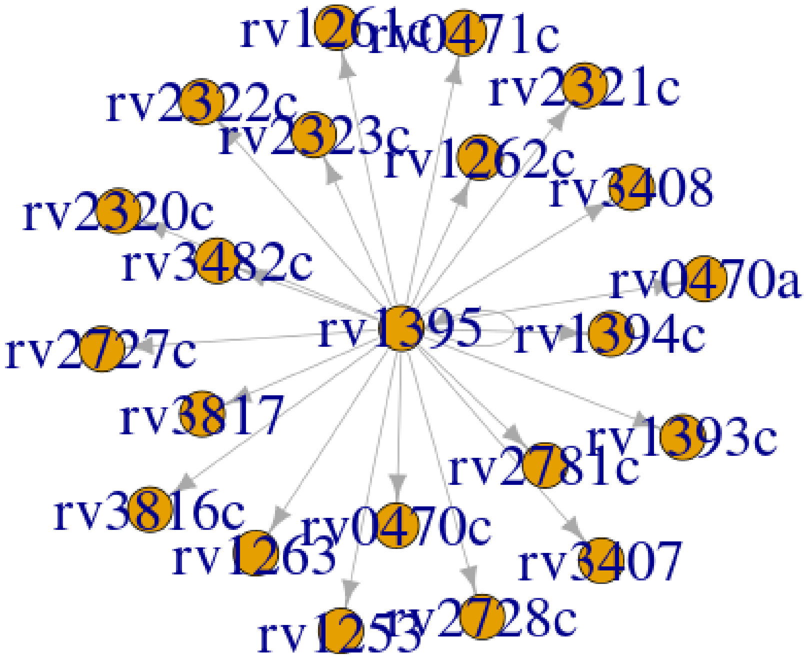

Micobacterium tuberculosis

AR

Regulators:

Fiber: RV0735, RV0736, RV1139C, RV1660, RV2877C, RV2878C

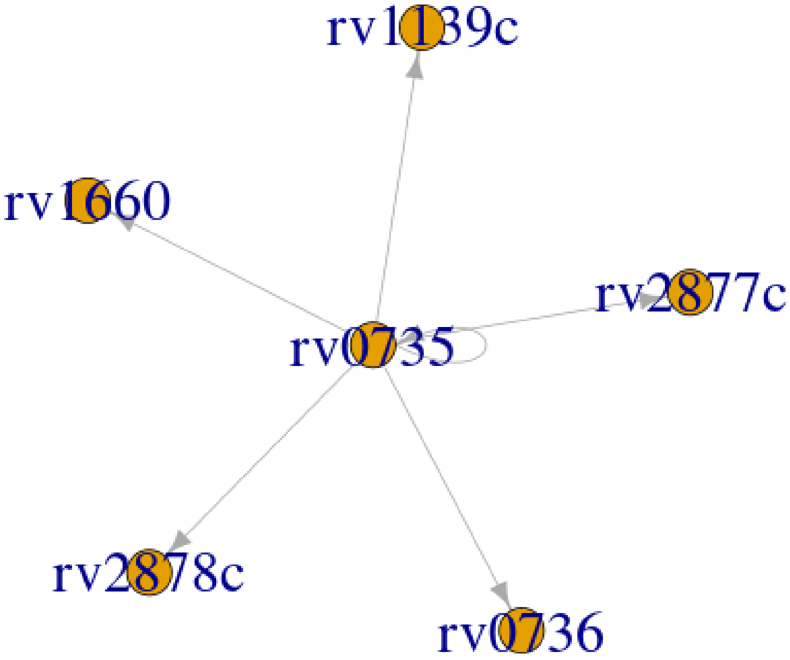

Micobacterium tuberculosis

AR

Regulators:

Fiber: RV1316C, RV1317C

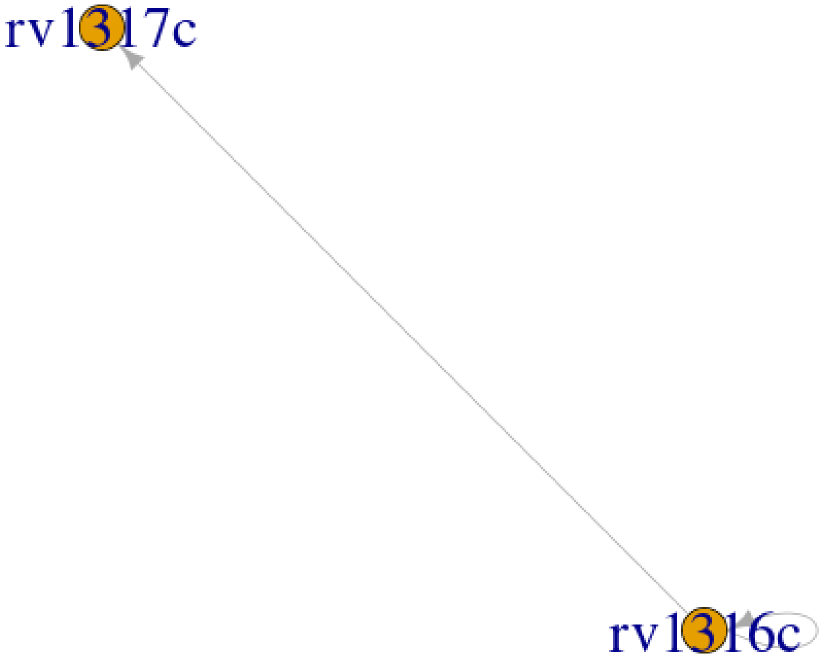

Micobacterium tuberculosis

FFF

Regulators: RV2711

Fiber: RV0586, RV0587

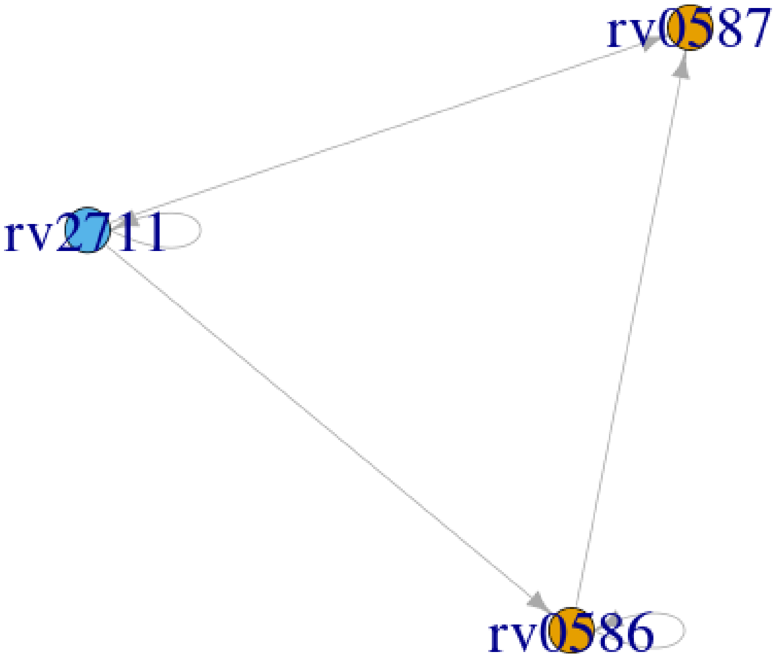

Micobacterium tuberculosis

FFF

Regulators: RV0485, RV0491, RV0757, RV2017, RV2669, RV3692, RV3833, RV3911

Fiber: RV3132C, RV3133C, RV3134C

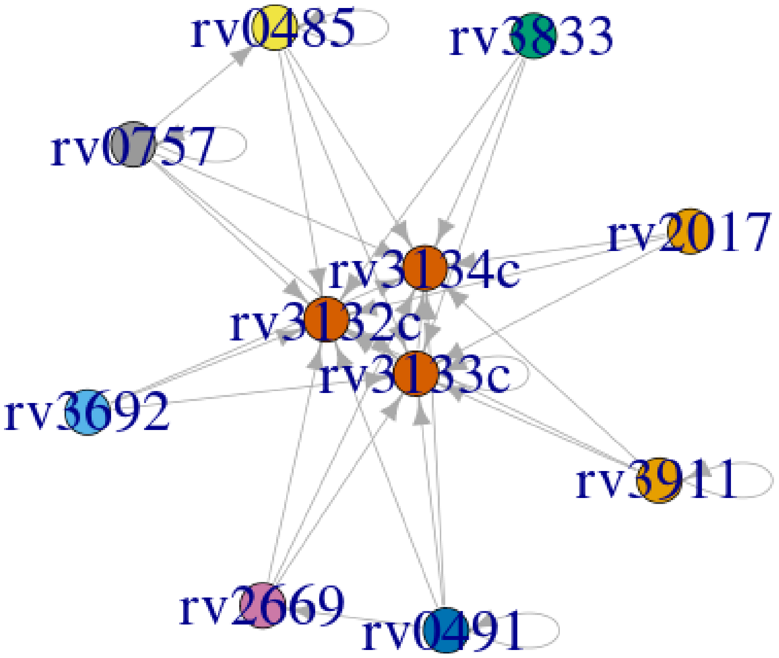

Micobacterium tuberculosis

Fibonacci

Regulators: RV1221

Fiber: RV0347, RV0348, RV0349, RV0981, RV0982, RV0983, RV0984, RV1297, RV1641, RV1642, RV1643, RV1644, RV2193, RV2194, RV2195, RV2196, RV2441C, RV2442C, RV2945C, RV2946C, RV2947C, RV2948C, RV2949C, RV2950C

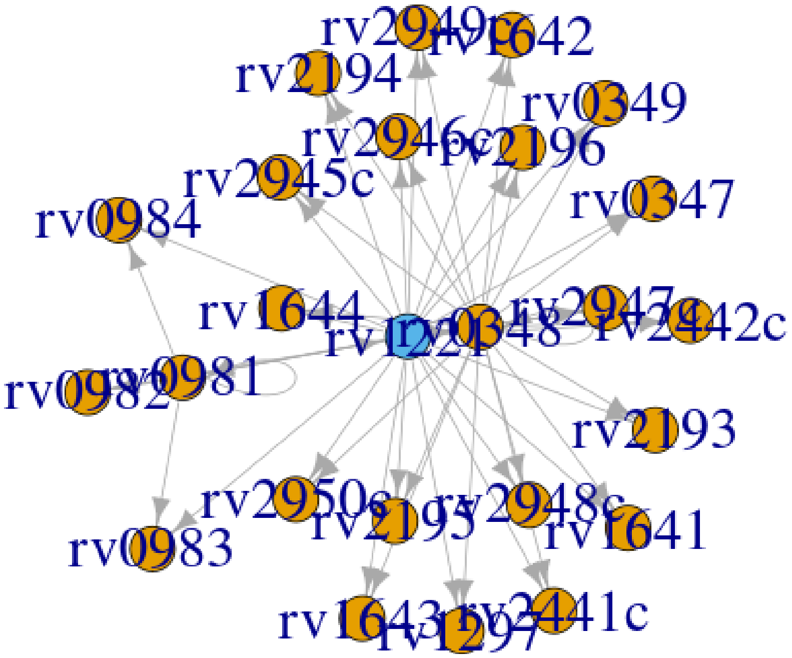

Micobacterium tuberculosis

n = 2

Regulators: RV3676

Fiber: RV2358, RV2359

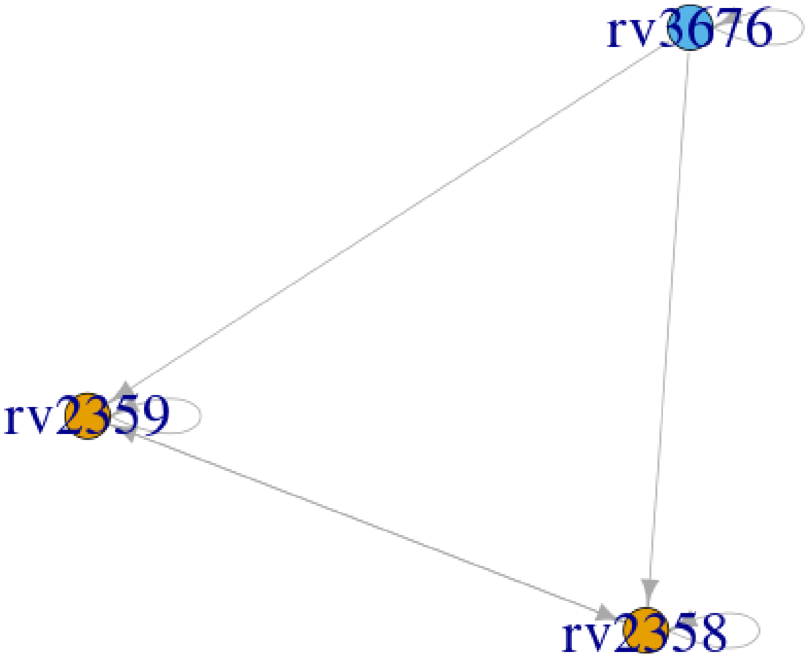

Bacillus subtilis

AR

Regulators:

Fiber: COMZ, DIVIVA, DNAA, DNAG, DNAN, FTSE, FTSX, MED, METS, PARA, PARB, PDEH, PDXS, PDXT, PIT, SACT, SEPF, SPO0A, TAGV, TKT, TSAC, YABD, YKAA, YLMD, YLME, YLMG, YLMH, YQXD, YQZC, YQZD, YRRL, YVYE, YWCI, YYDA

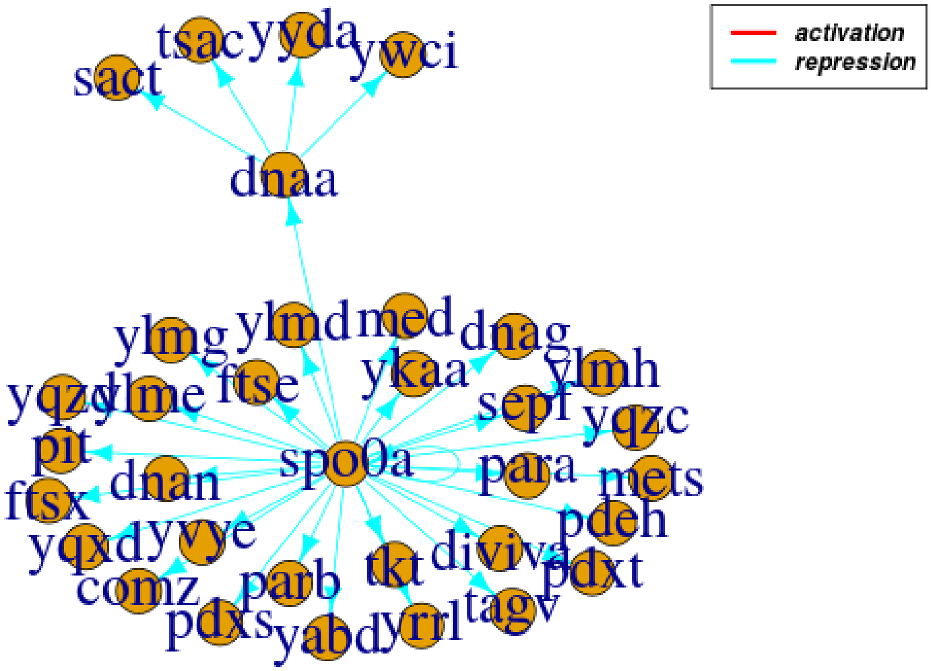

Bacillus subtilis

AR

Regulators:

Fiber: HYPO, HYPR

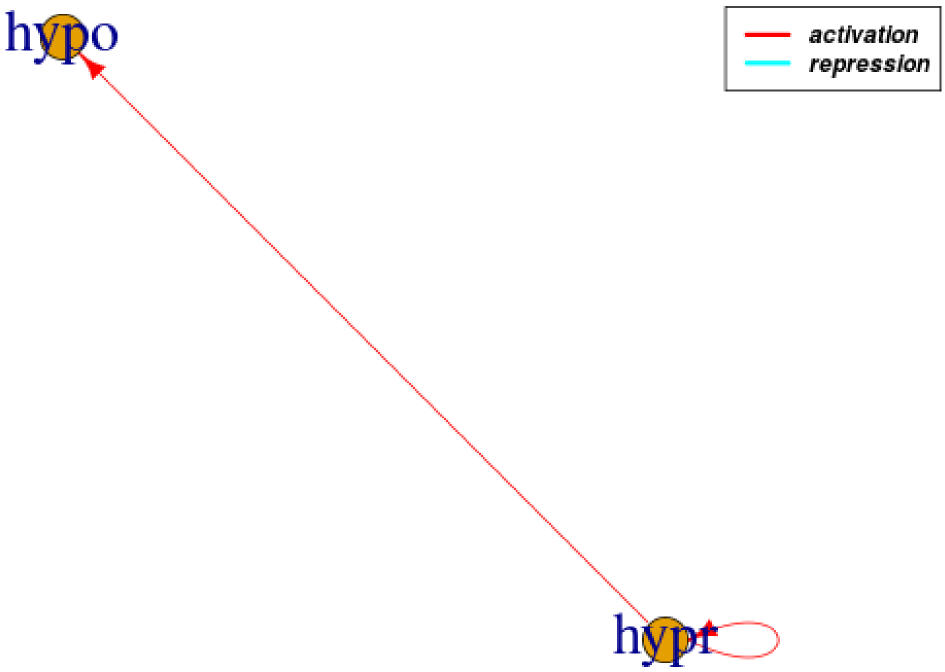

Bacillus subtilis

AR

Regulators:

Fiber: ANSA, ANSB, ANSR

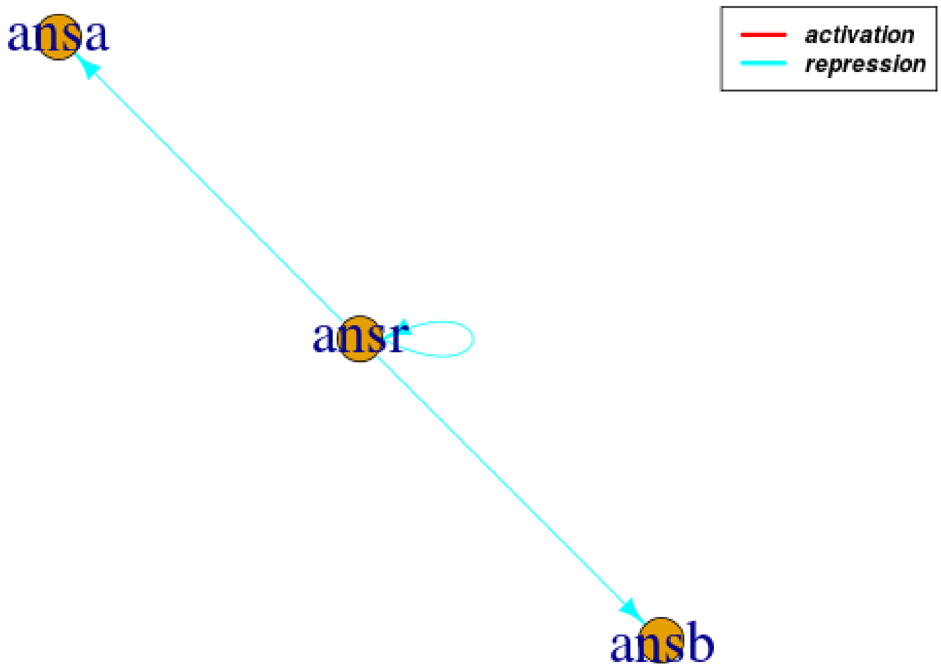

Bacillus subtilis

AR

Regulators:

Fiber: ARSB, ARSC, ARSR, YQCK

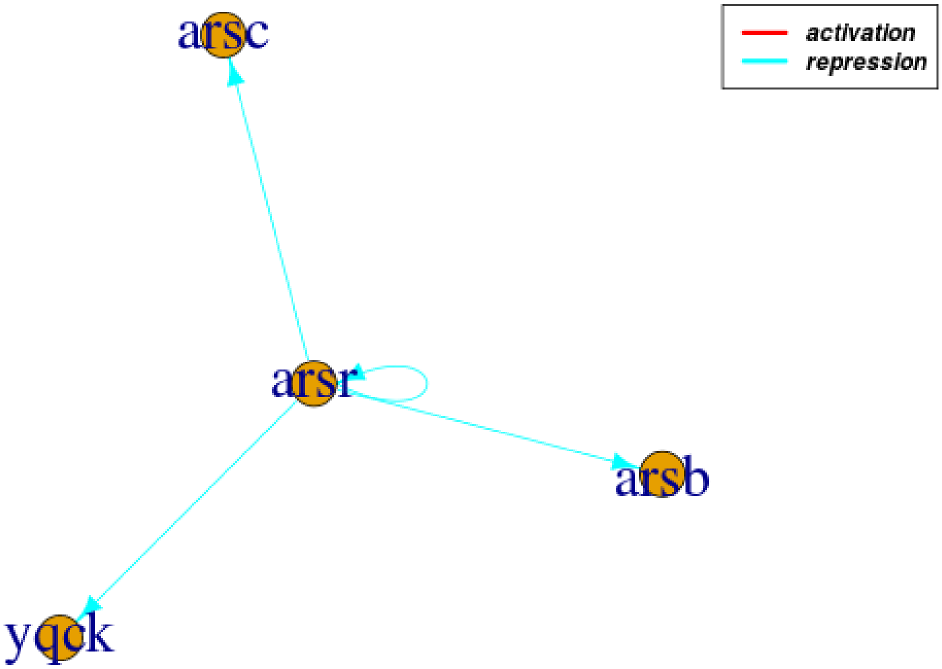

Bacillus subtilis

AR

Regulators:

Fiber: ADAA, ADAB, ALKA

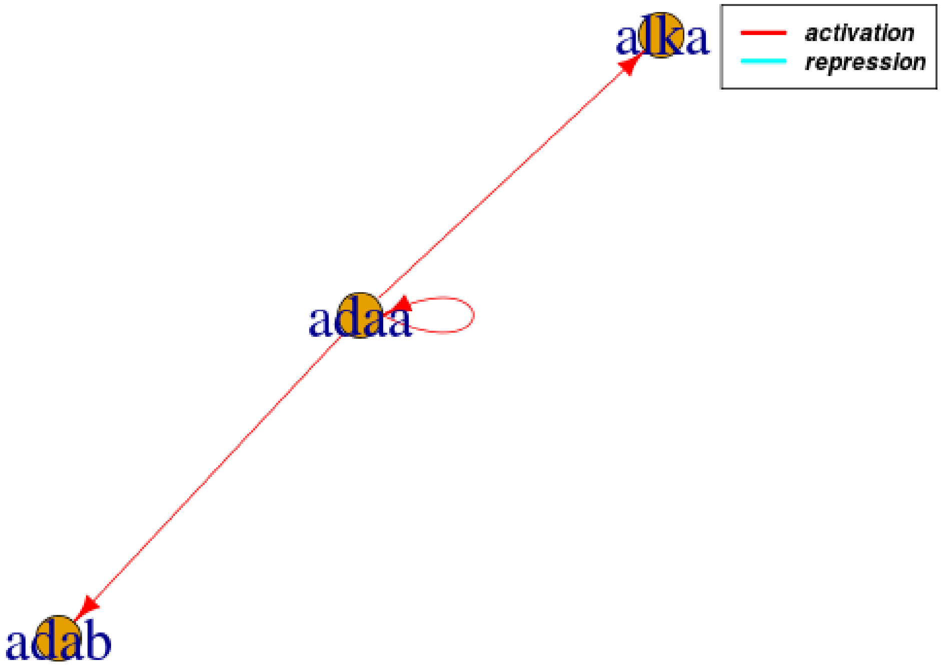

Bacillus subtilis

AR

Regulators:

Fiber: GAMA, GAMP, GAMR, YBGB

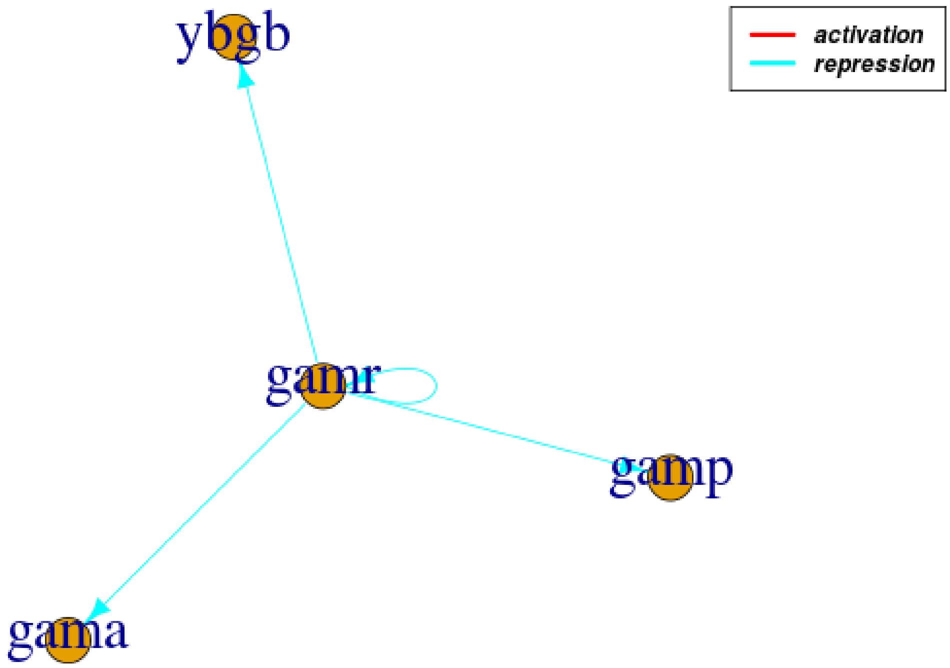

Bacillus subtilis

AR

Regulators:

Fiber: FATR, YRHJ

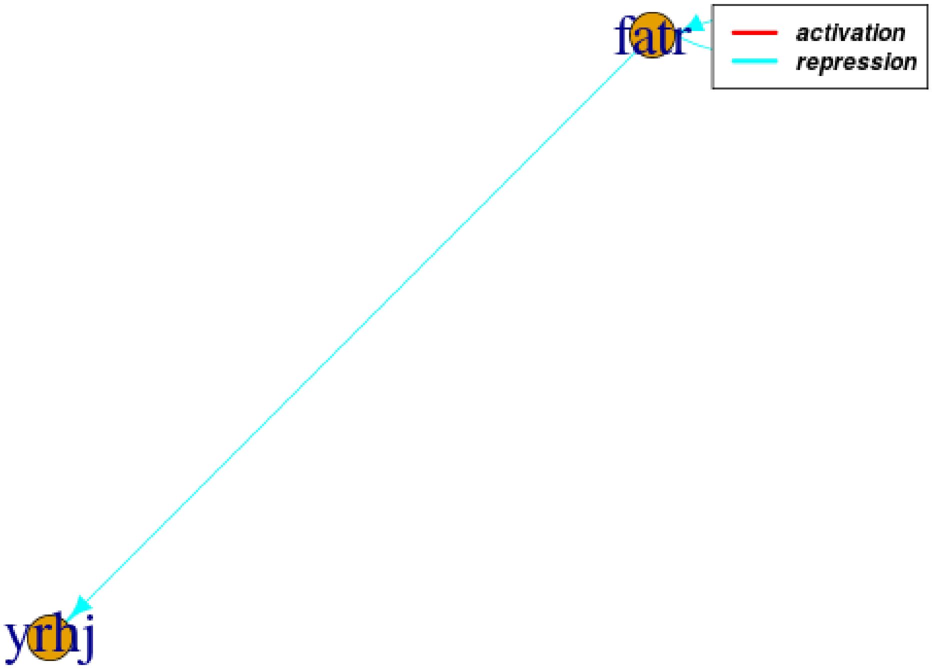

Bacillus subtilis

AR

Regulators:

Fiber: PSDA, PSDB, PSDR, PSDS

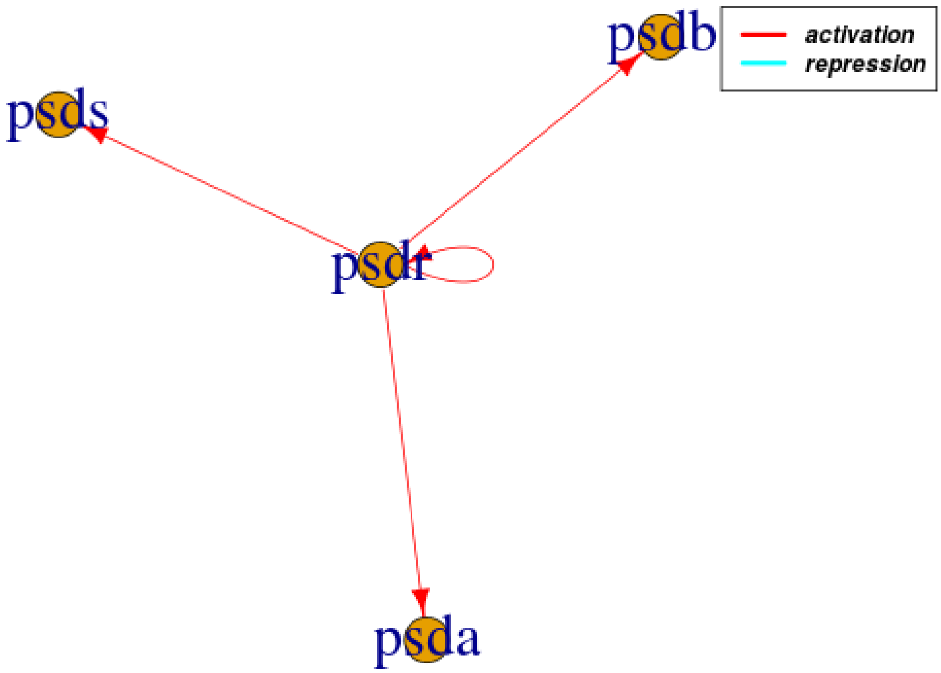

Bacillus subtilis

AR

Regulators:

Fiber: LMRA, LMRB

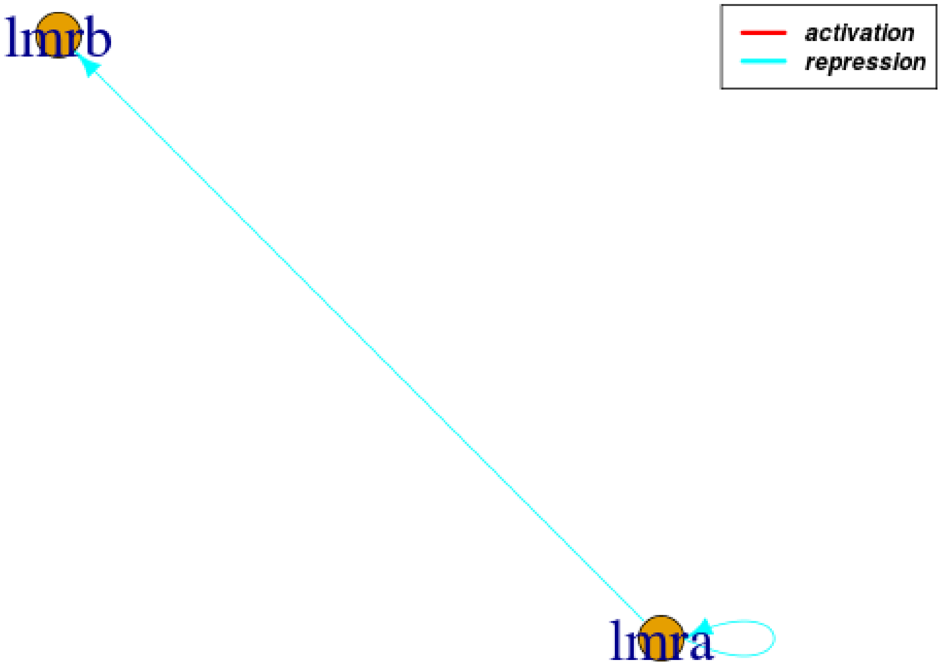

Bacillus subtilis

AR

Regulators:

Fiber: FOLD, GLYA, ISPE, NUSB, PBUG, PBUO, PBUX, PURA, PURB, PURC, PURD, PURE, PURF, PURH, PURK, PURL, PURM, PURN, PURQ, PURR, PURS, XPT, YABJ

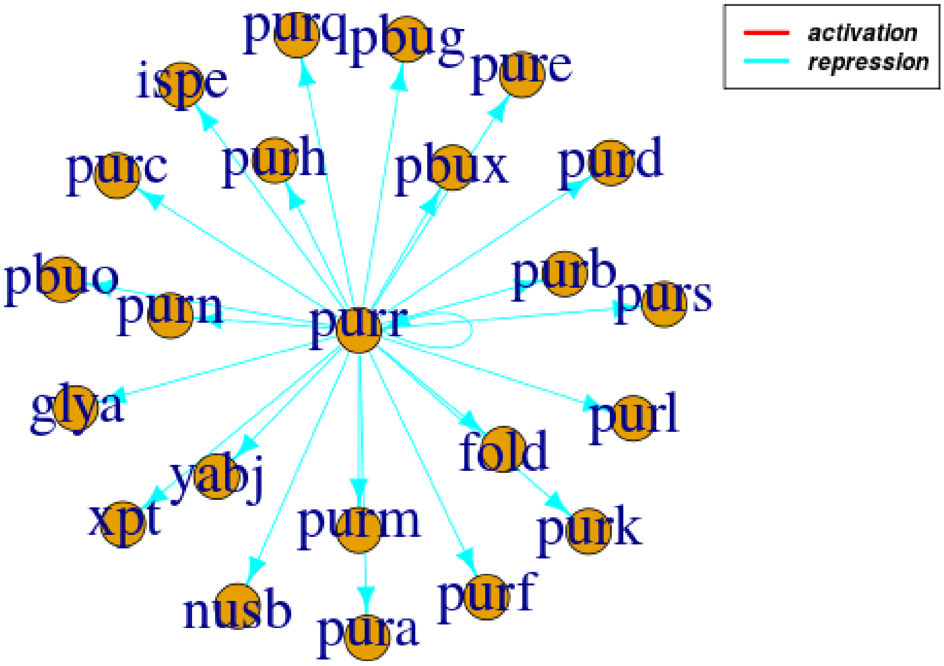

Bacillus subtilis

AR

Regulators:

Fiber: CSSR, CSSS, HTRA, HTRB

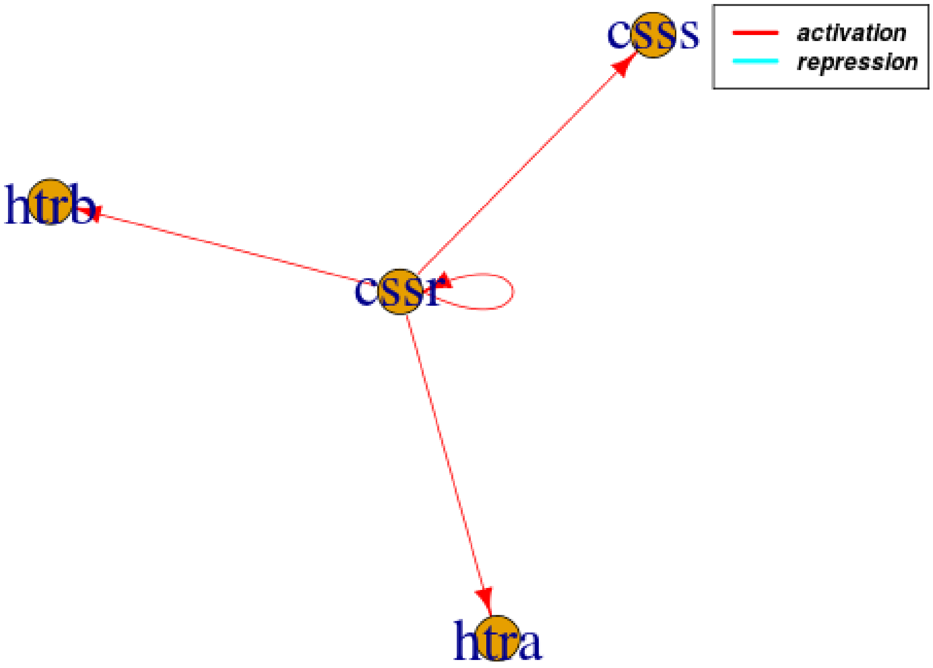

Bacillus subtilis

AR

Regulators:

Fiber: PAMR, YDCF, YDCG

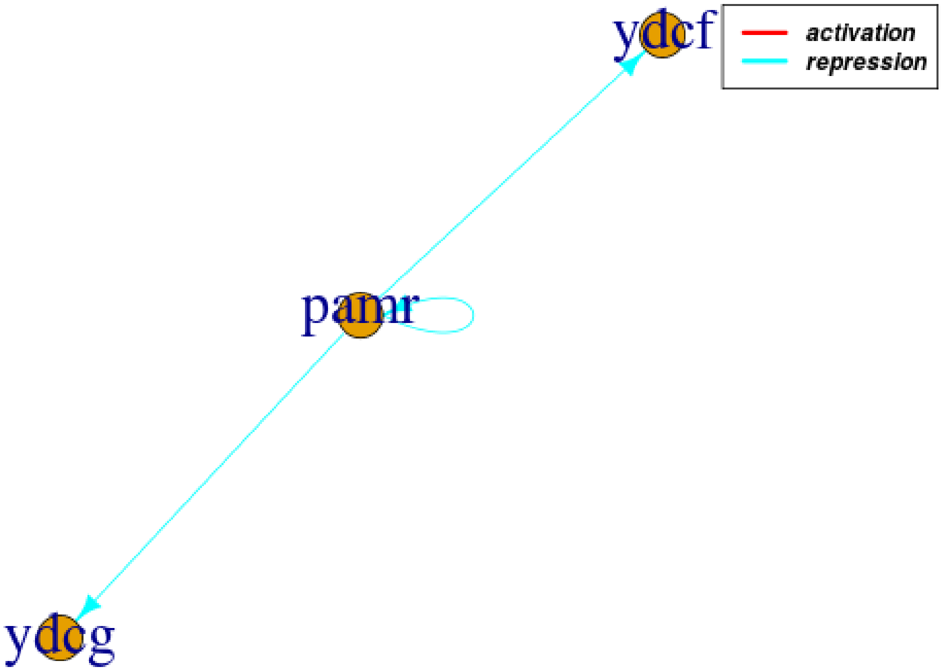

Bacillus subtilis

AR

Regulators:

Fiber: AMIE, MURP, MURQ, MURR, NAGZ, YBBC

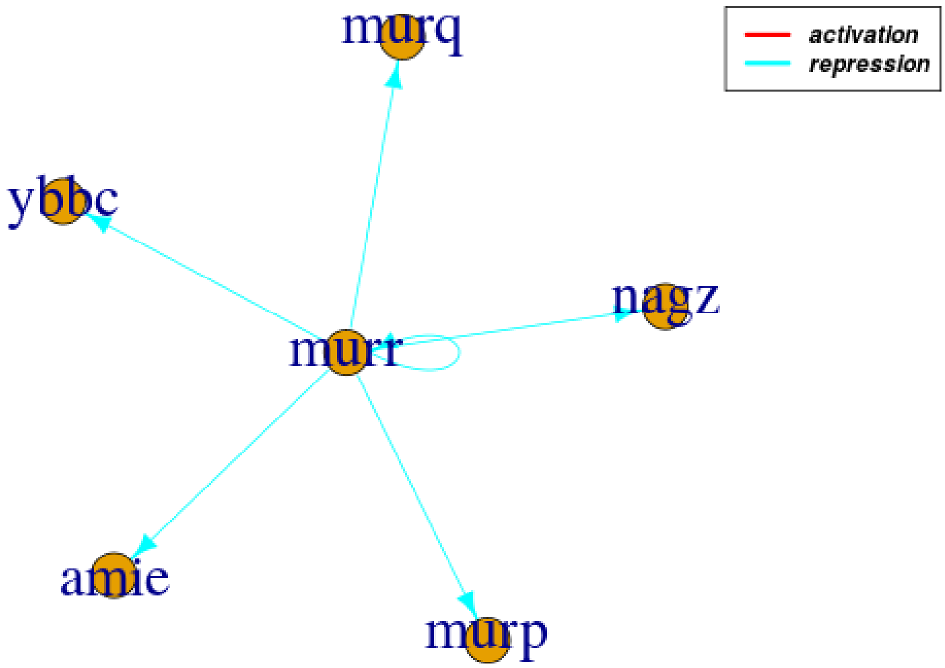

Bacillus subtilis

FFF

Regulators: TNRA

Fiber: KIPR, PUCR, PXPA, PXPB, PXPC, YCSG, YCSI, YFIR, YFIS

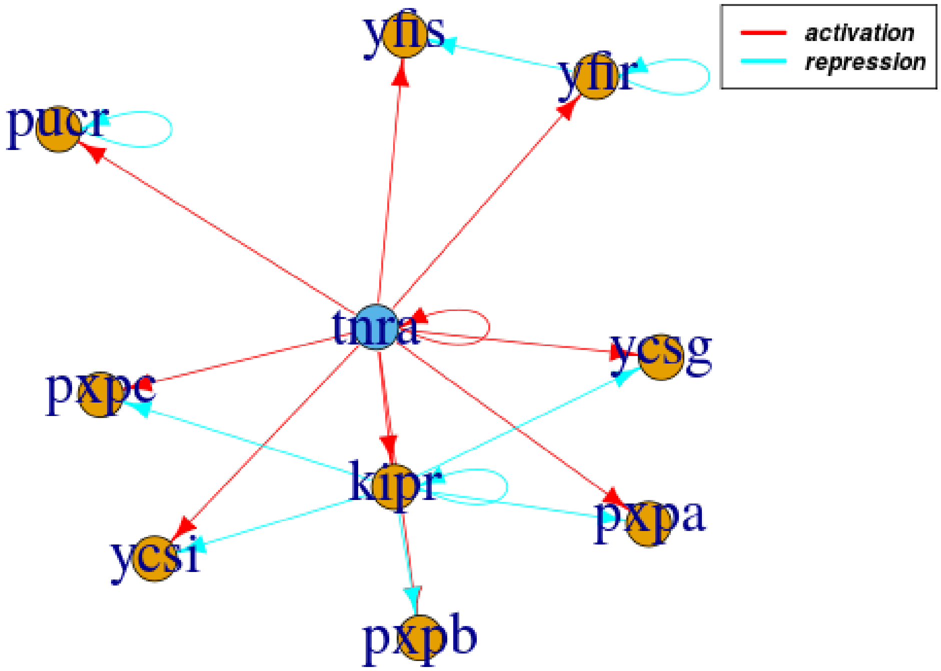

Bacillus subtilis

FFF

Regulators: CCPA, SINR, TNRA

Fiber: DEGS, DEGU

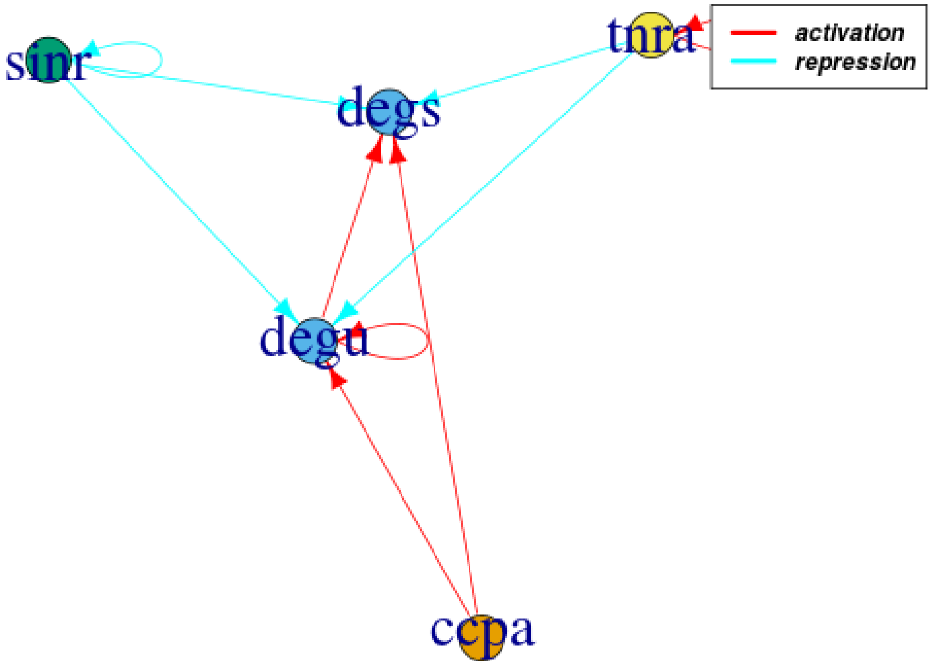

Bacillus subtilis

FFF

Regulators: ABRB

Fiber: SDPI, SDPR, YCBC, YCBD, YCBE, YCBF, YCBG, YCBH, YCBJ, YCNI, YCNJ, YCNK

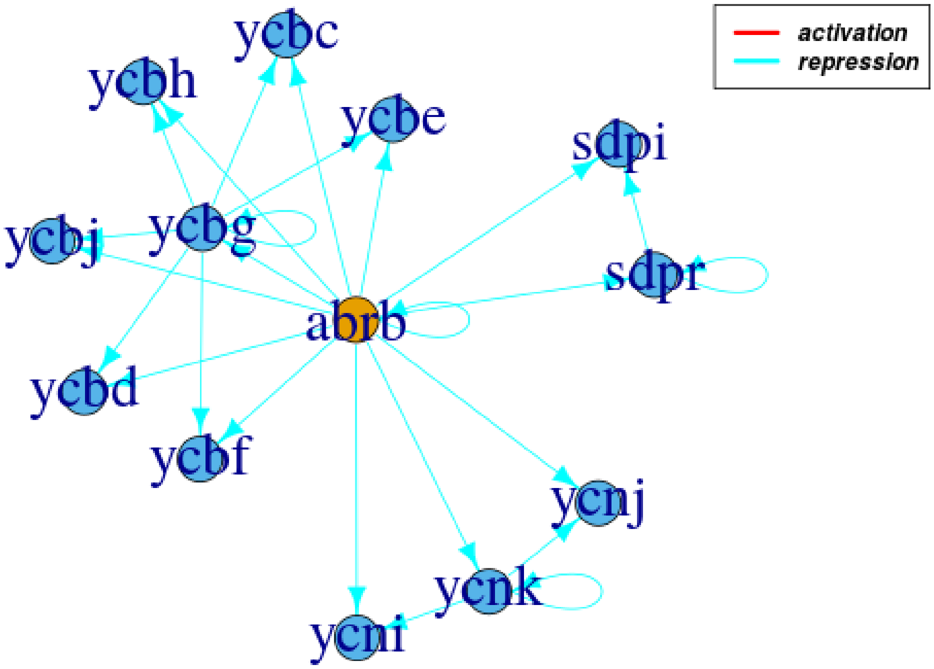

Bacillus subtilis

FFF

Regulators: CCPA

Fiber: GLVR, MALA, MALP, MANR

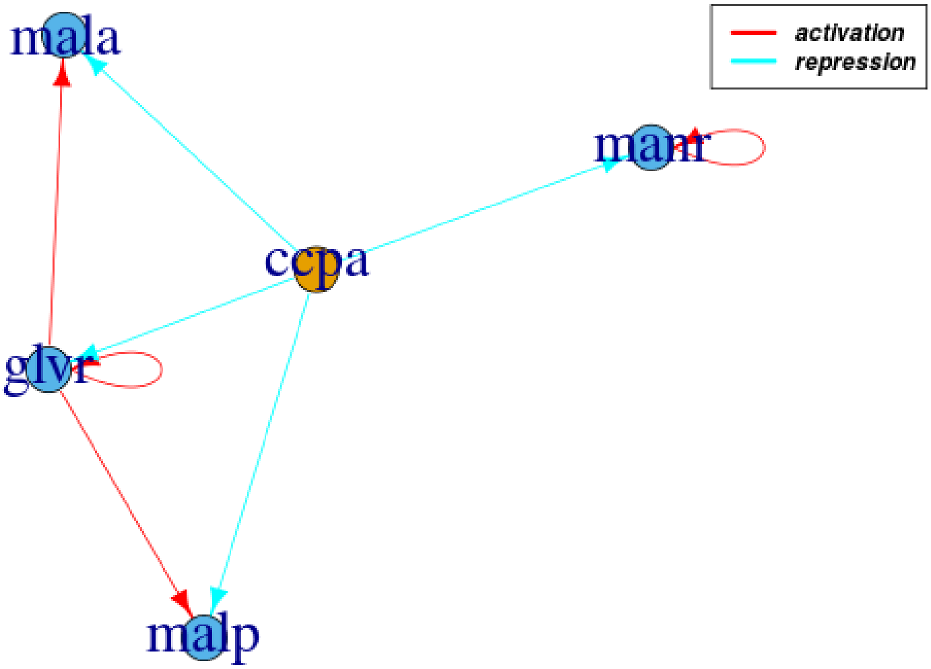

Bacillus subtilis

FFF

Regulators: MTA

Fiber: BMR, BMRR

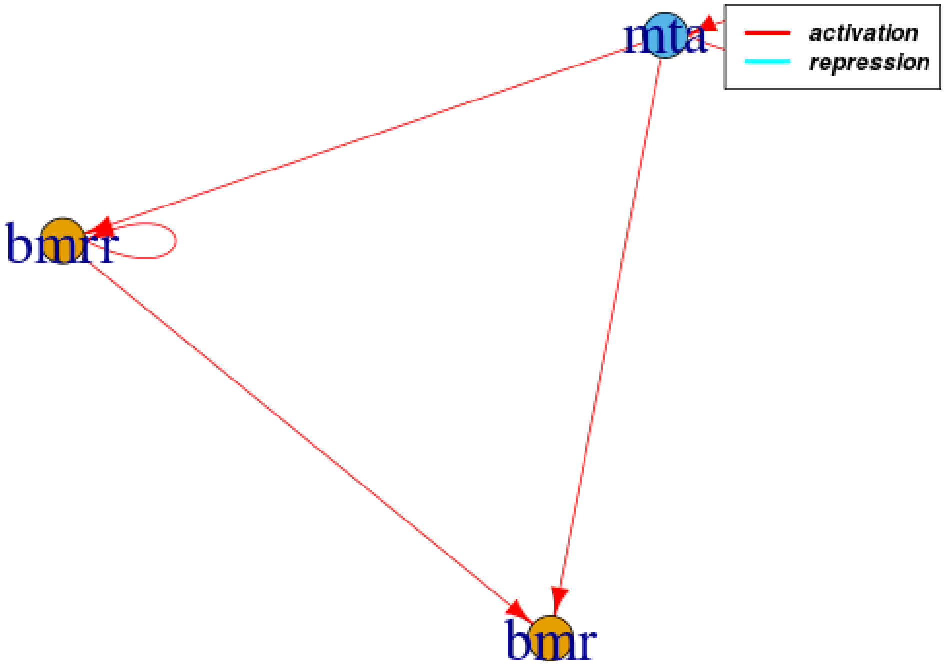

Bacillus subtilis

n = 2

Regulators:

Fiber: FTSH, HPRT, TILS

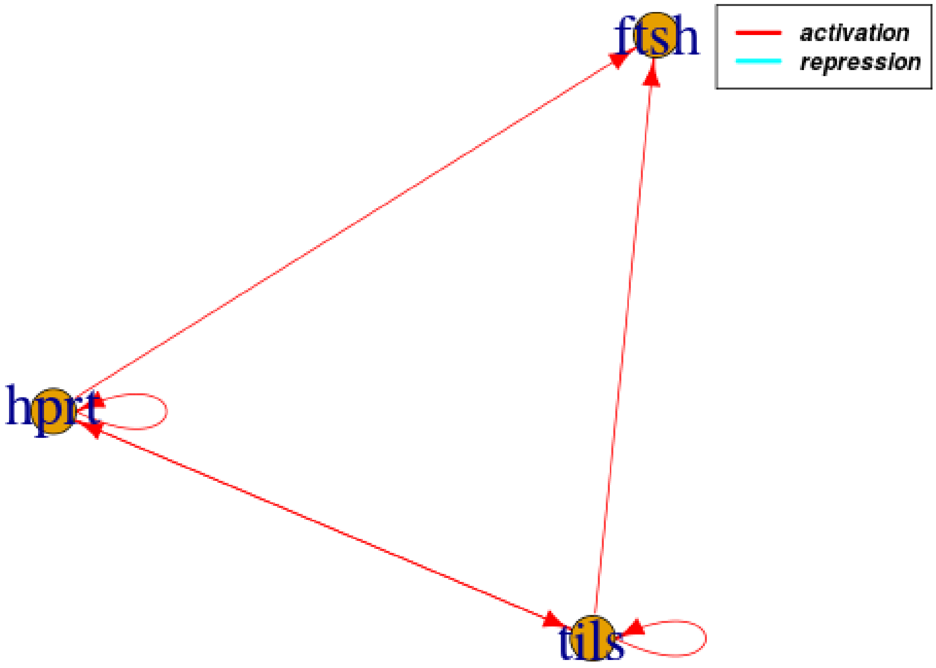

Bacillus subtilis

n = 2

Regulators:

Fiber: LEXA, ROCR

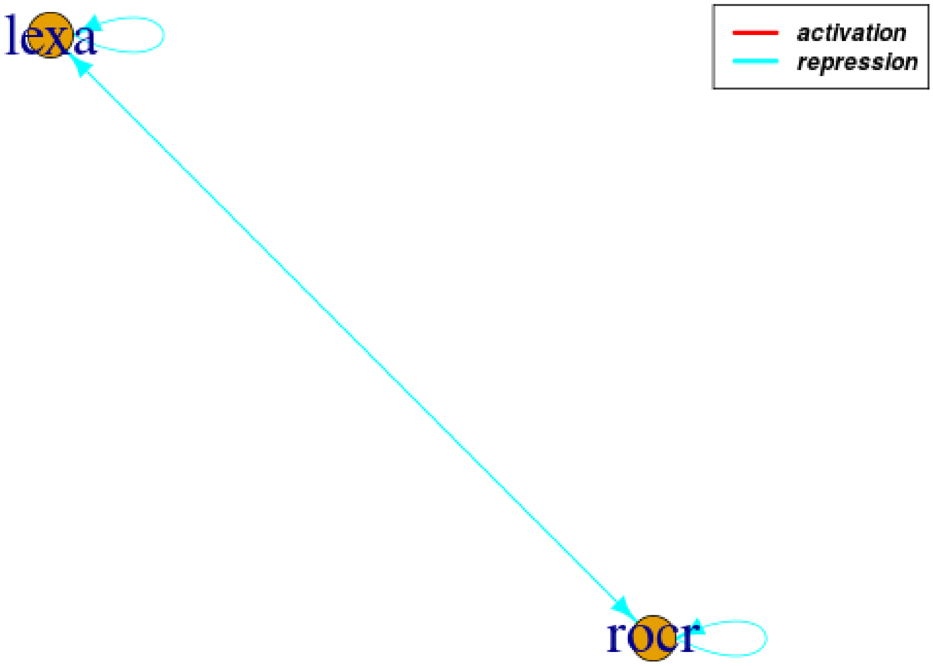

Arabidopsis Thaliana

Fibonacci

Regulators: EGL3, MYB5, TT8

Fiber: ABE4, MES6, MYBL2

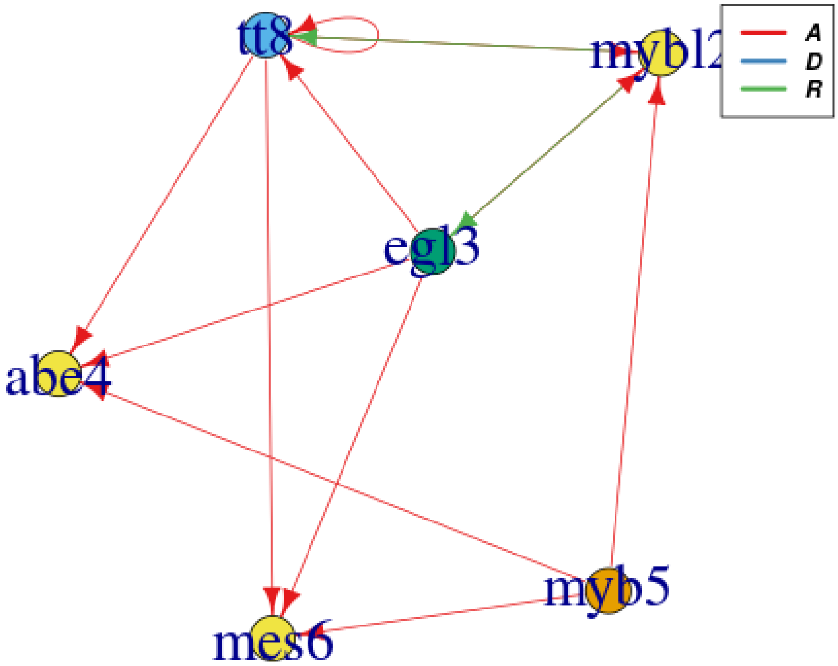

Salmonella SL1344

AR

Regulators:

Fiber: ICC, MAP, MGTB, PARE, PCKA, RAMA, RPSB, SL1344_0567, SL1344_0568, SL1344_1073, SL1344_1074, SL1344_1075, SL1344_2102, SL1344_2751, SL1344_2752, SL1344_2753, SL1344_2754, SL1344_3156, SL1344_3158, SL1344_3159, SL1344_P2_0054A, YHGE

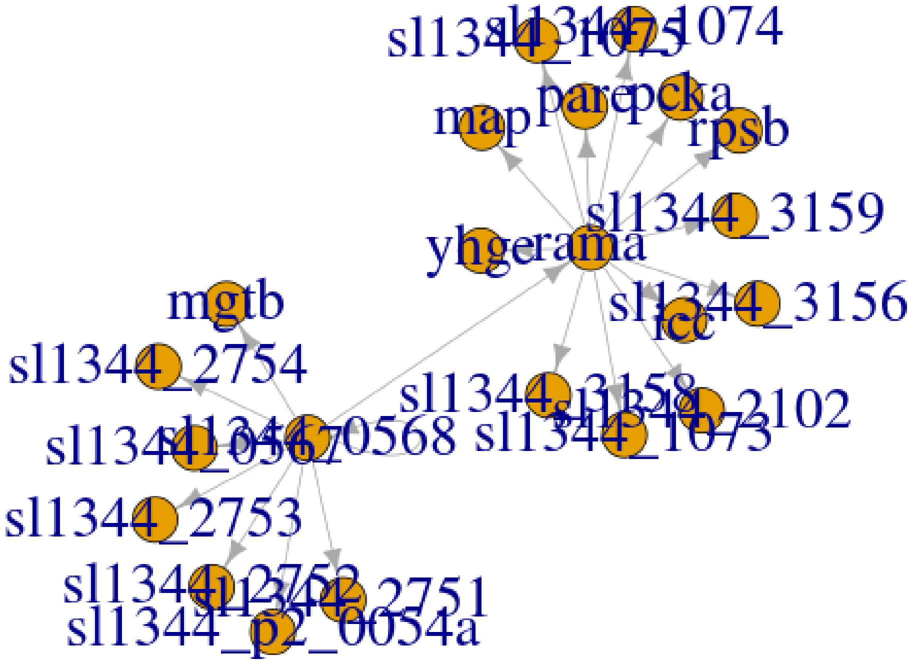

Salmonella SL1344

AR

Regulators:

Fiber: SL1344_2370, YABN

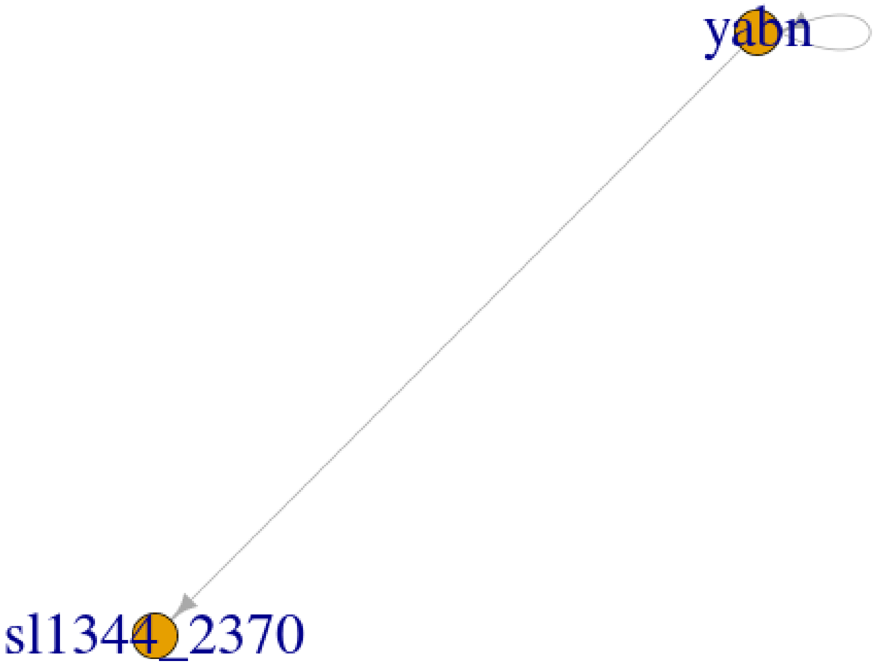

Salmonella SL1344

AR

Regulators:

Fiber: TORA, TORC, TORD, TORR

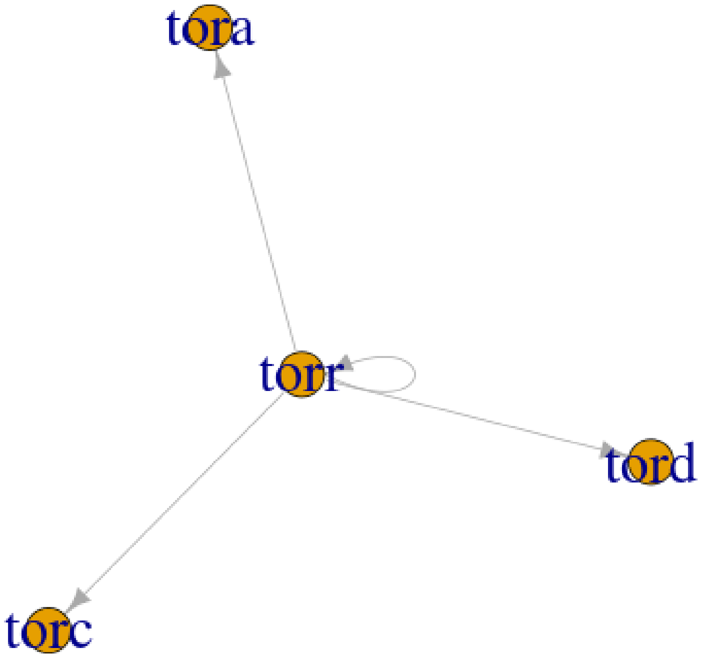

Salmonella SL1344

AR

Regulators:

Fiber: FOLA, TYRP, TYRR

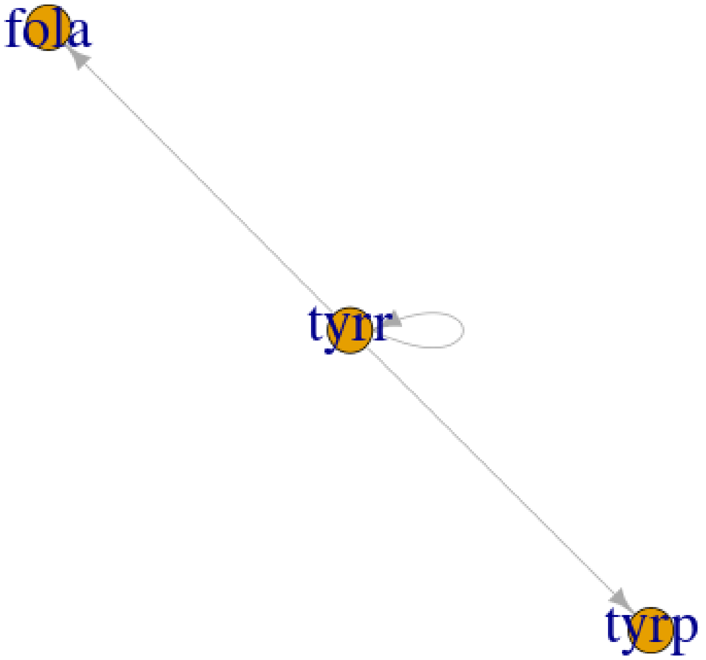

Salmonella SL1344

AR

Regulators:

Fiber: FABD, FABG, FABH, FADR, PLSX, SL1344_0448, USPA

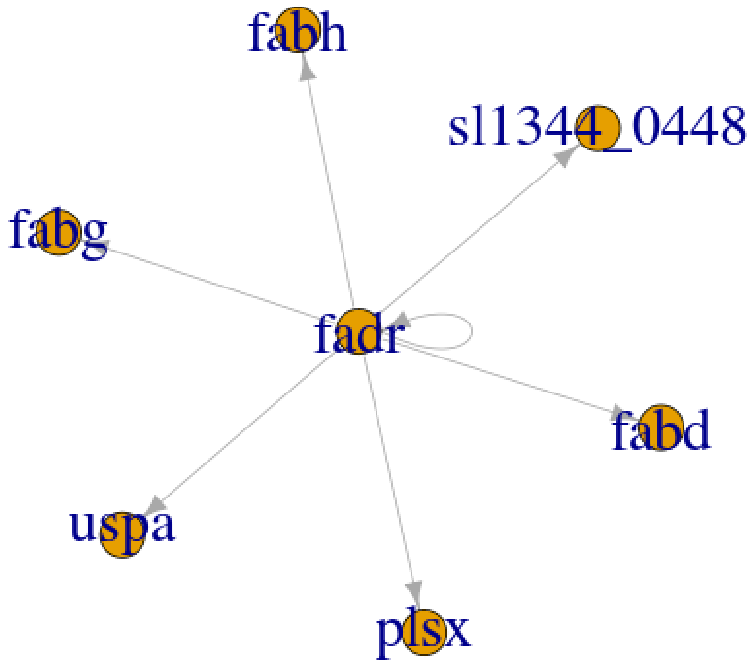

Salmonella SL1344

AR

Regulators:

Fiber: FLDA, FLDB, ISPZ, PGI, RIBA, SL1344_1404, SL1344_1663, SL1344_1664, SOXR, SOXS, YCIC

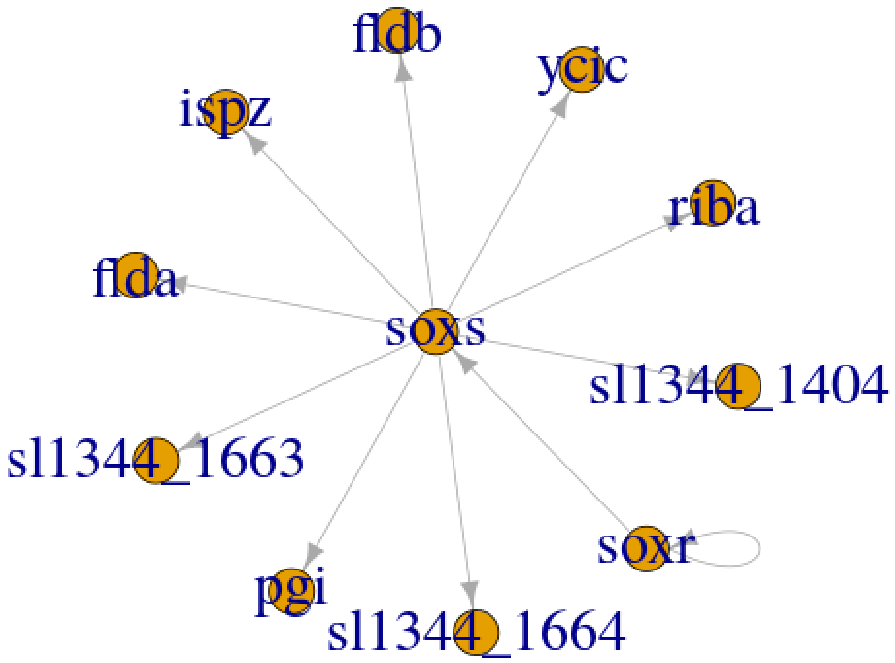

Salmonella SL1344

AR

Regulators:

Fiber: PSPA, PSPB, PSPC, PSPD, PSPE, PSPF, YJBO

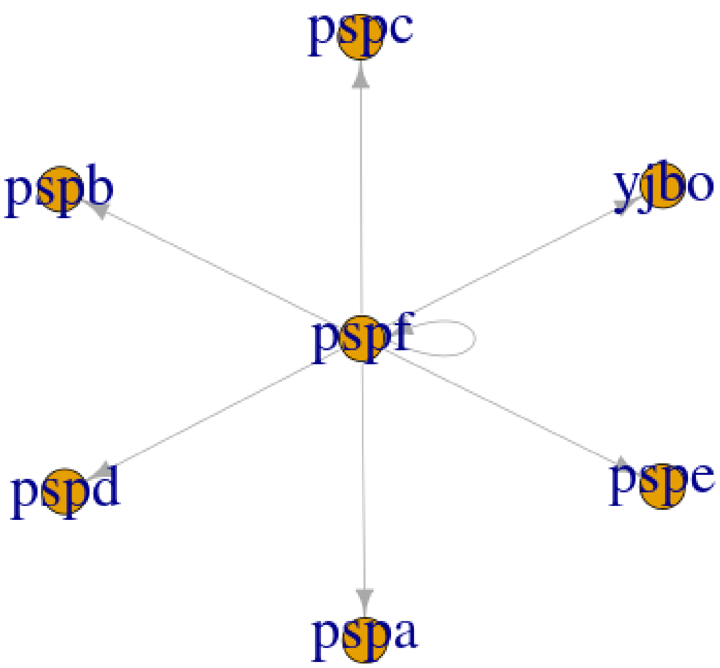

Salmonella SL1344

AR

Regulators:

Fiber: CHEA, CHEW, CPXA, CPXP, CPXR, DSBC, HTRA, MOTA, MOTB, PSD, RECJ, RPOE, RSEA, RSEB, RSEC, SL1344_1152, SL1344_3199, SL1344_3200, SL1344_3777, XERD, YECI, YJEP

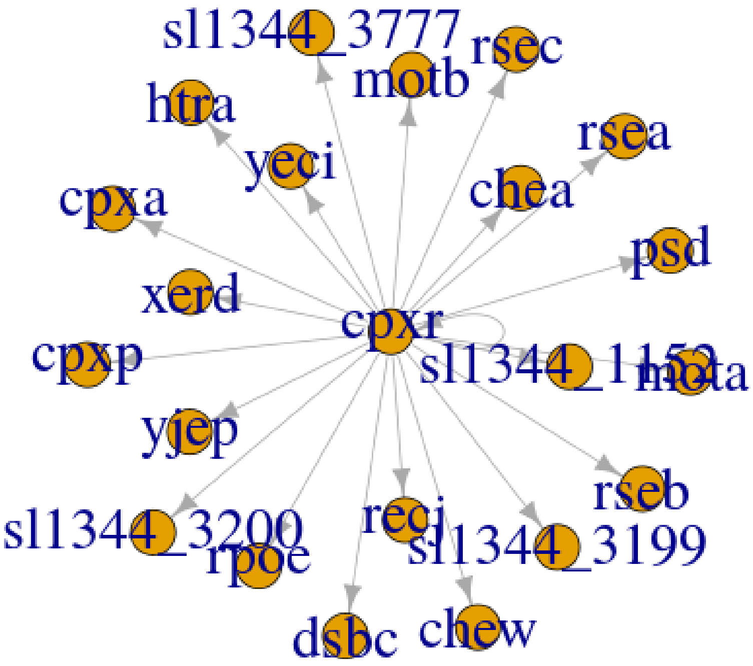

Salmonella SL1344

FFF

Regulators: LRP

Fiber: ADA, ALKB, YOJI

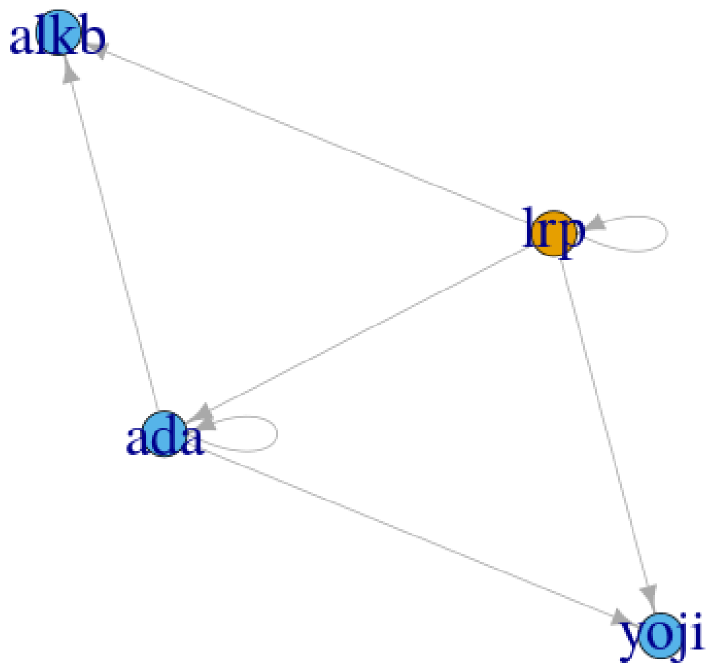

Salmonella SL1344

FFF

Regulators: SL1344_0674

Fiber: ASNC, GIDA, GIDB

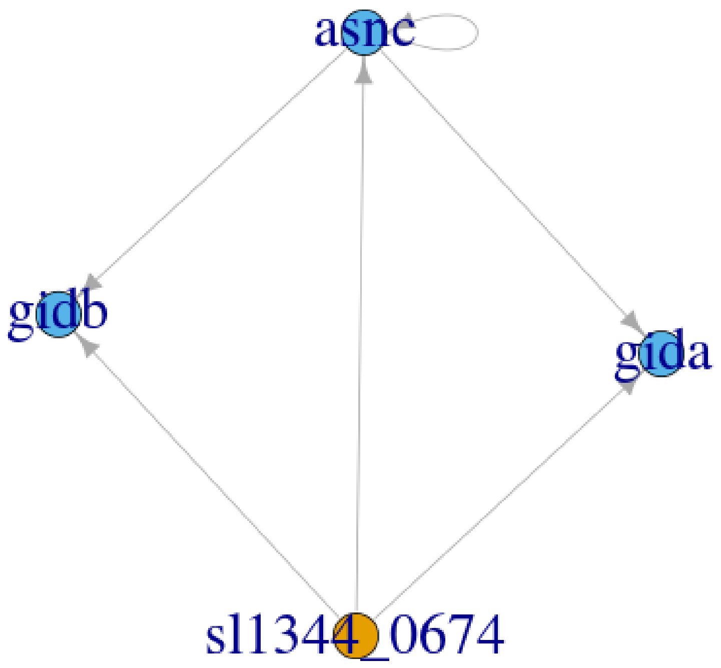

Salmonella SL1344

FFF

Regulators: OMPR

Fiber: HEXR, SOPD2, SSAV, SSEA, SSEG, SSRB

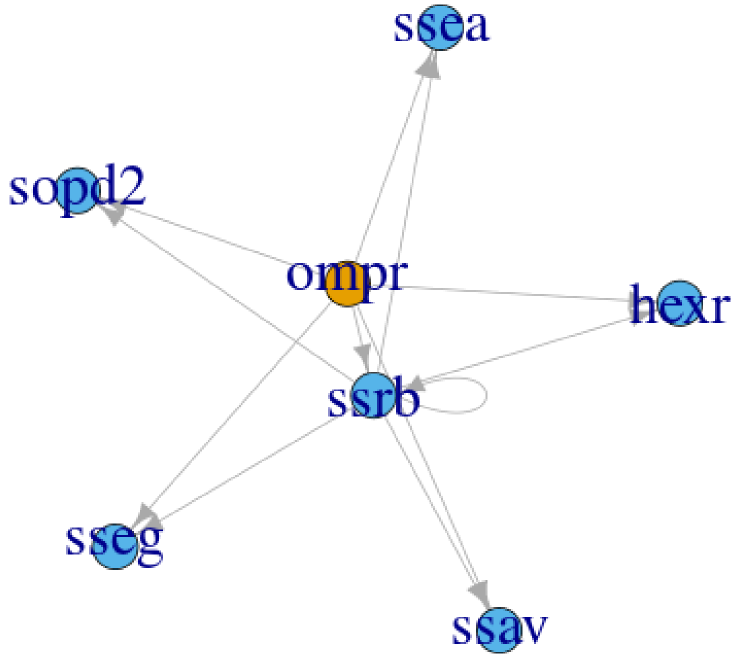

Salmonella SL1344

FFF

Regulators: SL1344_1060

Fiber: GLOA, NEMA, SL1344_1369

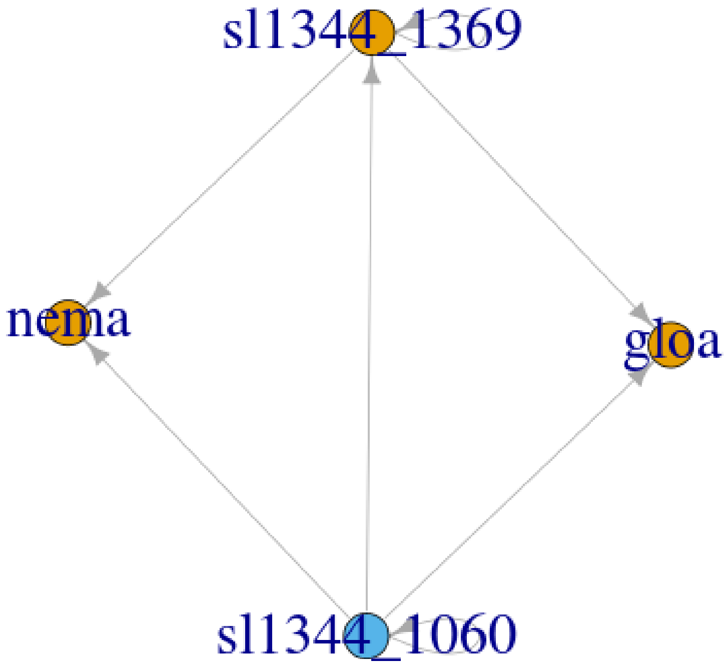

Salmonella SL1344

FFF

Regulators: FADR

Fiber: ICLR, SL1344_4121

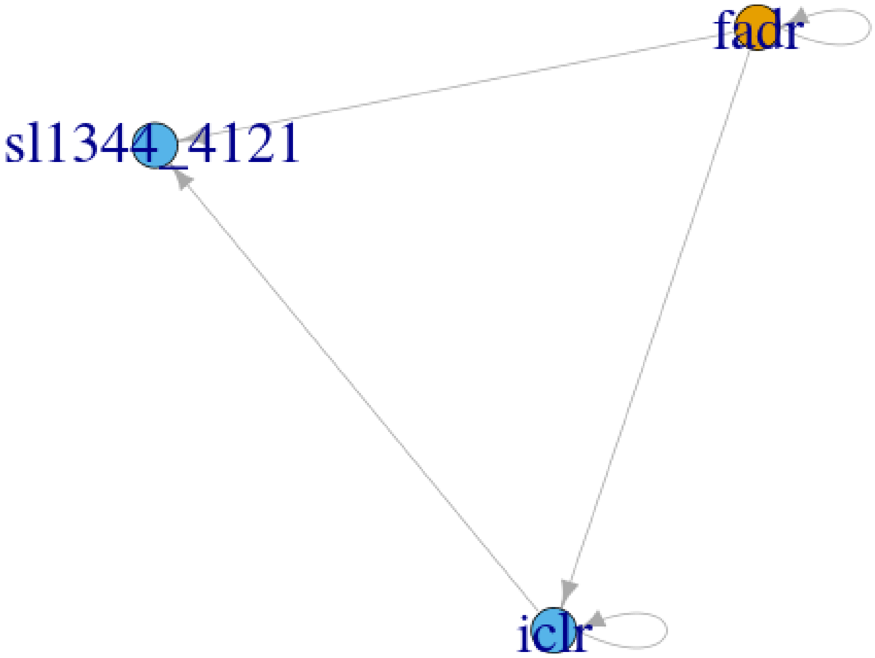

Salmonella SL1344

n = 2

Regulators:

Fiber: ACS, ALDB, ARAA, ARAB, ARAC, ARAD, ARAE, ARAJ, CAP, CSTA, CUER, CYTR, FIS, GALR, GALS, GLNA, HUPA, HUPB, HYDG, HYDH, MALK, MALT, MGLA, MGLC, NAGA, NAGB, NAGC, NAGD, NAGE, PROP, RPLM, RPSI, SL1344_0168, SL1344_0345, SL1344_0346, SL1344_0347, SL1344_0348, SL1344_0349, SL1344_0350, SL1344_1418, SL1344_2990, SL1344_3357, SL1344_3616, SL1344_4110, TRG, UDP, YACK, YBAR, YFIA, YHFK, YIAJ

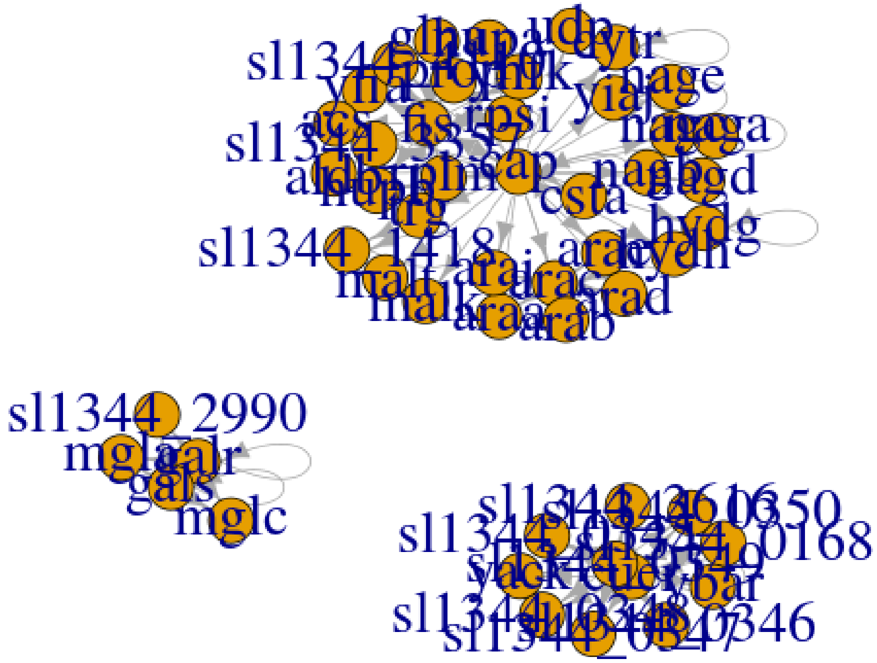

Human TRRUST2

Fibonacci

Regulators: FOS, JUN, SP1

Fiber: CREM, EZR

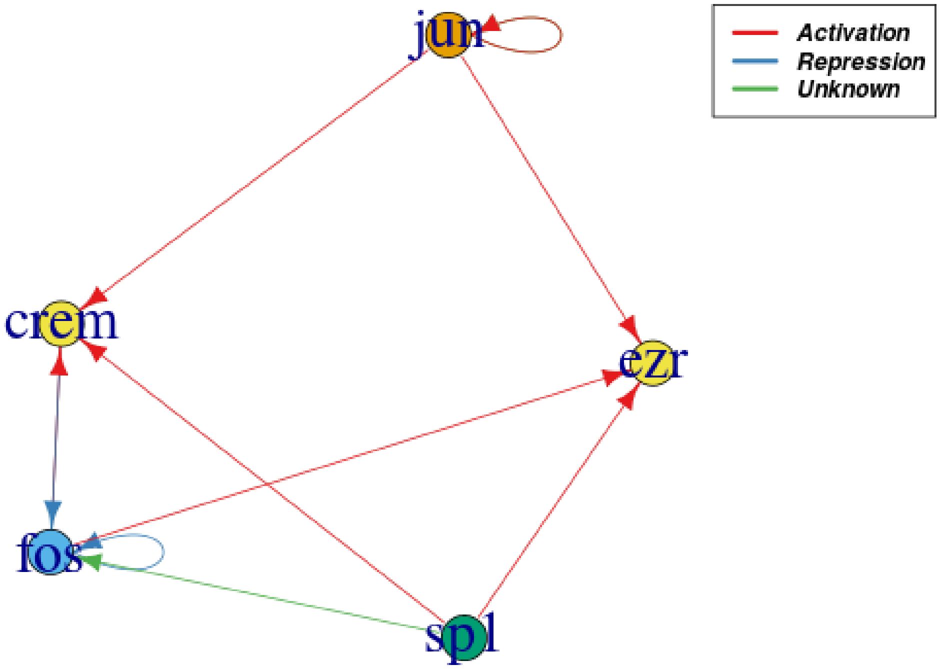

Human TRRUST2

Fibonacci

Regulators: HIF1A

Fiber: ARNT2, ERCC2, FAM162A, MUC3A, PFKFB3, PFKFB4, PPP1R3C, TLR6, VHL

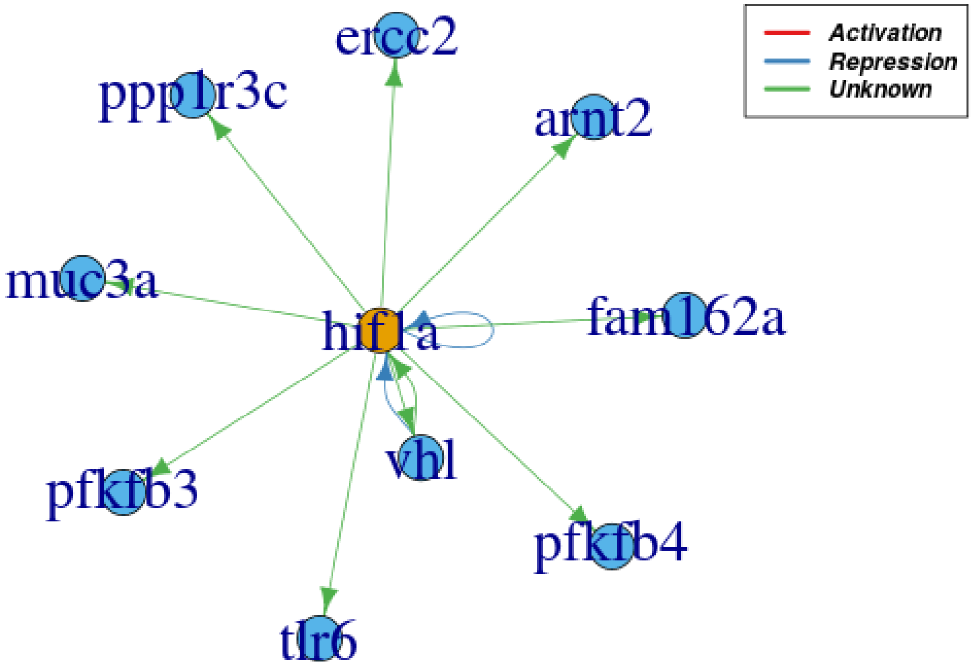

Human TRRUST2

Fibonacci

Regulators: TP53

Fiber: APCS, E2F7, GML, NLRC4, PAX5, SFN, UHRF1BP1

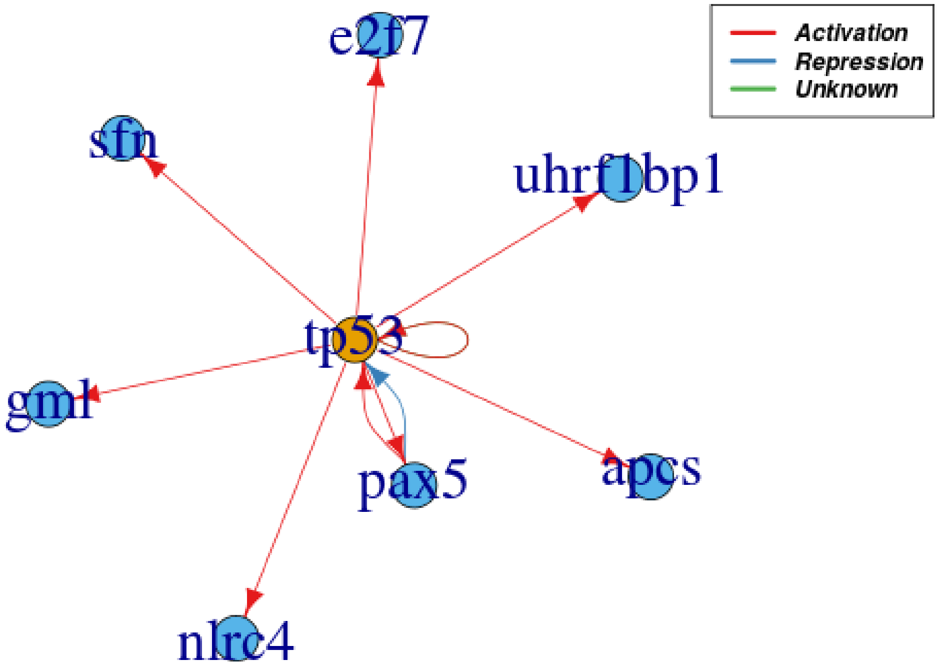

Human TRRUST2

Fibonacci

Regulators: MYC

Fiber: DDX18, MTDH, RCC1, SART3, SHMT1, TFAM, TLX1, TOP1MT

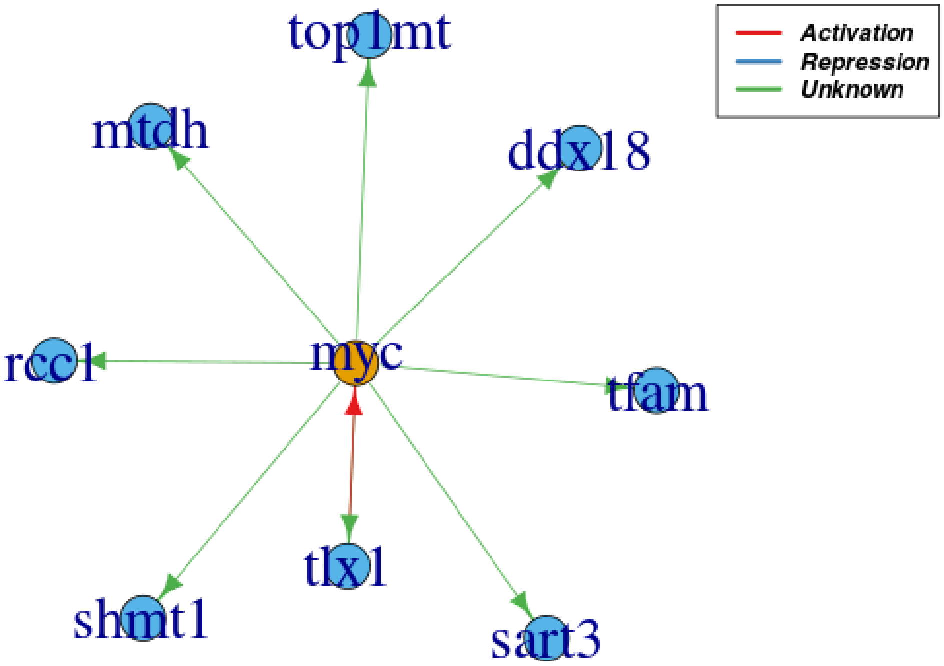

Mouse TRRUST

Fibonacci

Regulators: NR0B1

Fiber: ESRRG, RLF

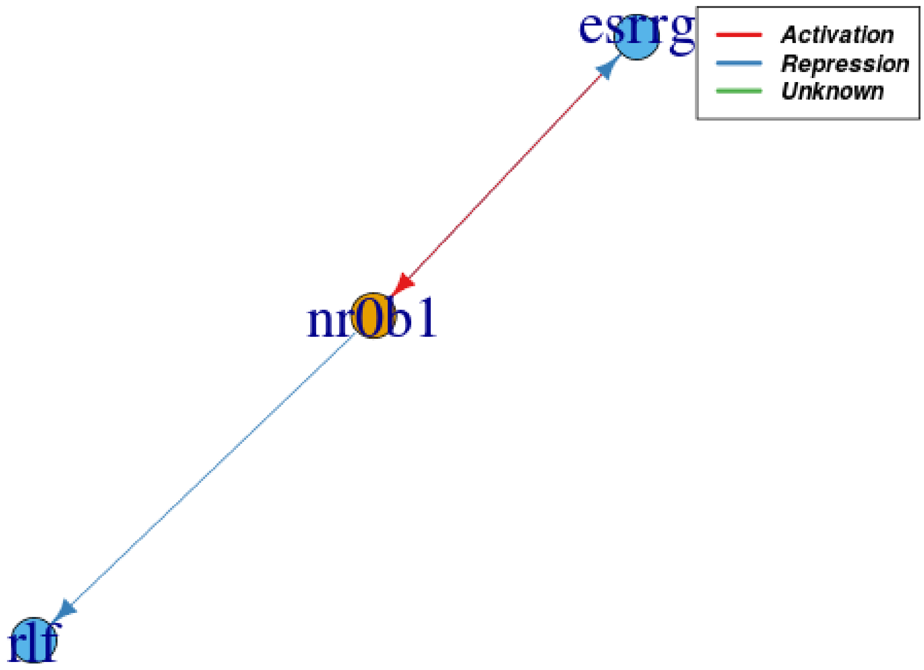

Mouse TRRUST

Fibonacci

Regulators: RUNX2

Fiber: ASRGL1, ELANEE, FGR18, LGALS3, SOX5, TCF7

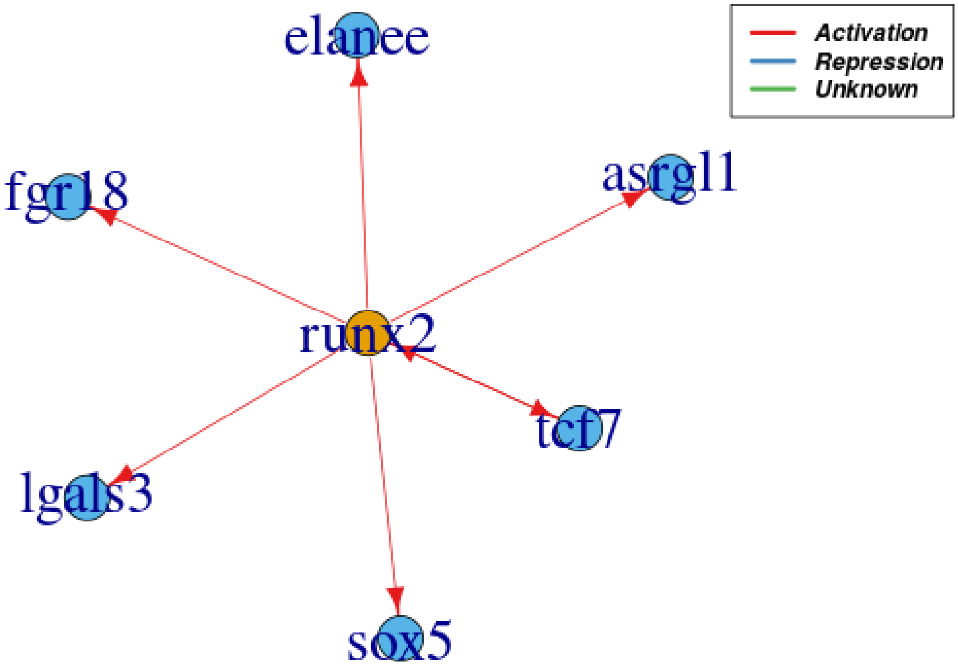

Yeast YTRP regulatory

AR

Regulators:

Fiber: ABF1, ANP1, ASP1, BDF2, BIR1, BIT61, BSD2, BYE1, CDC42, COS4, CPR5, DCW1, DFG10, DPP1, DSS4, ECM25, ERV46, EXO5, FIN1, GCN2, GON7, GRH1, GVP36, HFI1, HOF1, HUA1, IFA38, JID1, KRE1, KRE9, KTR4, LAT1, LDB16, LOT5, MAK16, MED2, MLC1, MNN11, MRP49, MRPL38, MRPL8, MTM1, NEO1, NGR1, NHX1, NIT3, NOB1, NTA1, NUP157, NUP57, ORM1, PBP2, PEX10, PMI40, PML39, PSK1, PSP1, RAD14, RGT2, RMD9, ROG1, RPB7, RSC2, RXT2, SEC11, SRY1, SSL2, SUP45, TAF11, TAF14, TCP1, TFB1, THP3, TIM18, UTP9, VAB2, VPS33, YBR096W, YBR197C, YCR085W, YDL012C, YDR157W, YDR340W, YDR366C, YDR476C, YEN1, YFL065C, YGL109W, YJR029W, YJR087W, YKL169C, YKR047W, YLR118C, YLR349W, YMR050C, YNL058C, YPL272C, YPQ1, YPR172W, YPT10, YTA7

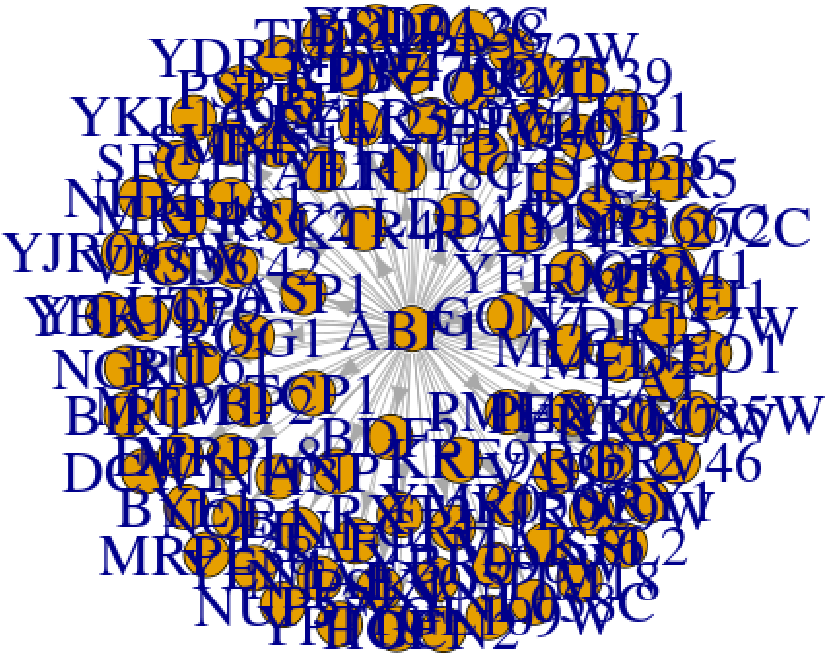

Yeast YTRP regulatory

AR

Regulators:

Fiber: ARG81, RHO4, YDL176W

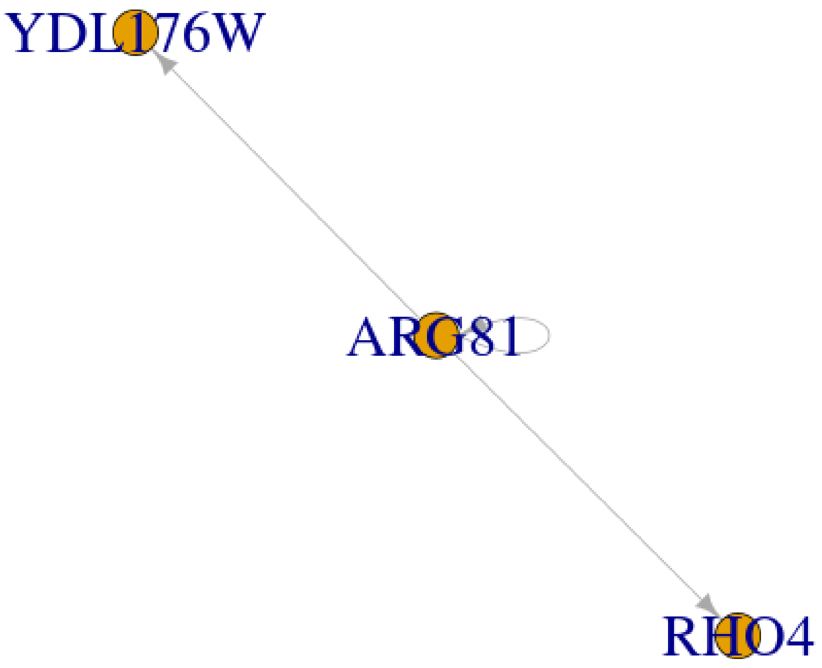

Yeast YTRP regulatory

AR

Regulators:

Fiber: AHC1, AIP1, ALG3, ARF1, BMH2, BRR1, BSC6, BUD22, CDC13, CEX1, CMS1, CSM2, EDS1, EGD1, FAF1, FAL1, FIR1, GIM3, HCS1, HMLALPHA2, IMD2, LCL2, LGE1, MAM3, MMF1, MOG1, MRE11, MRPL49, NMD5, NOP12, NSL1, NUP2, OMA1, ORC4, PAU18, PAU3, PAU8, PGA1, PIM1, PRO1, RAP1, RPL21B, RPL38, RPL40B, RPL43A, RPP2A, RPS17B, RPS18A, RPS23B, RPS29A, RTG1, RTR1, RVS167, SCP1, SEC22, SHR5, SLD5, SLM3, SNF4, SOP4, SPT20, SRB8, SSF2, TFA1, TIF34, TIM13, TOR2, TRM5, ULS1, VEL1, VMA6, VTS1, YAR075W, YDL032W, YDR095C, YDR187C, YDR391C, YDR417C, YDR541C, YEL008W, YEL023C, YFL034W, YFR018C, YGK3, YGL177W, YGR117C, YGR259C, YIL001W, YJL027C, YLR112W, YLR271W, YLR280C, YLR339C, YMR027W, YNL010W, YNR042W, YOR292C, YPL136W, YPL197C, YPL199C, YPL229W

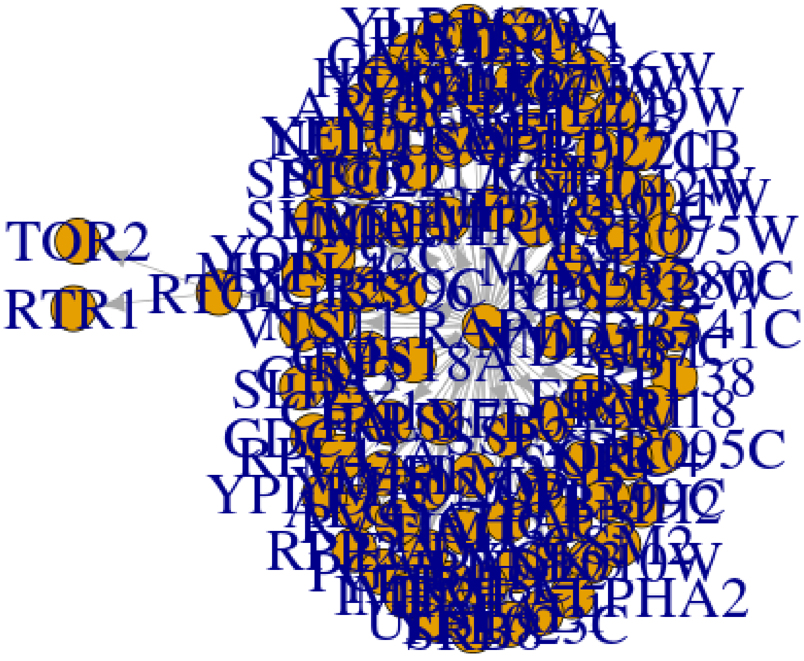

Yeast YTRP regulatory

FFF

Regulators: YAP1

Fiber: STB5, YRR1

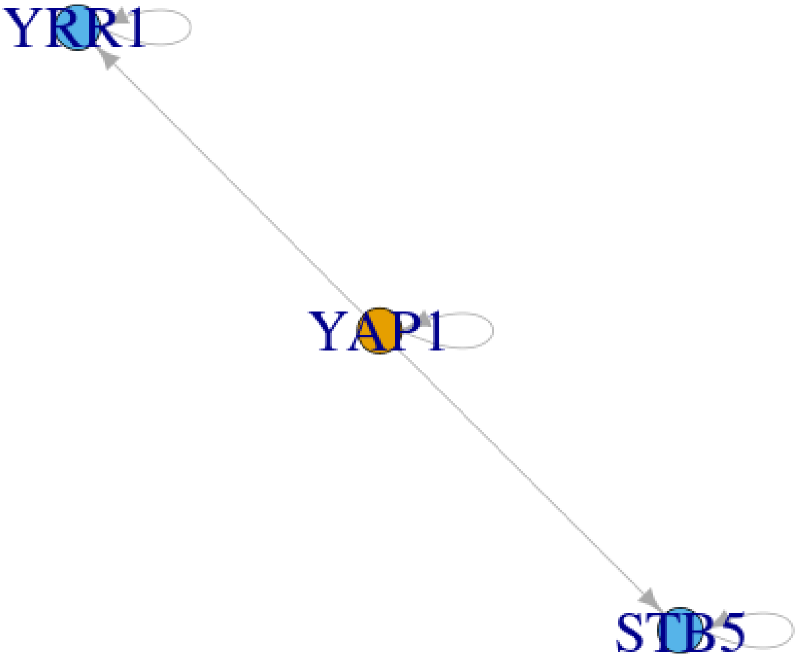

Human KEGG

Fibonacci

Regulators: GART

Fiber: PAICS, PFAS

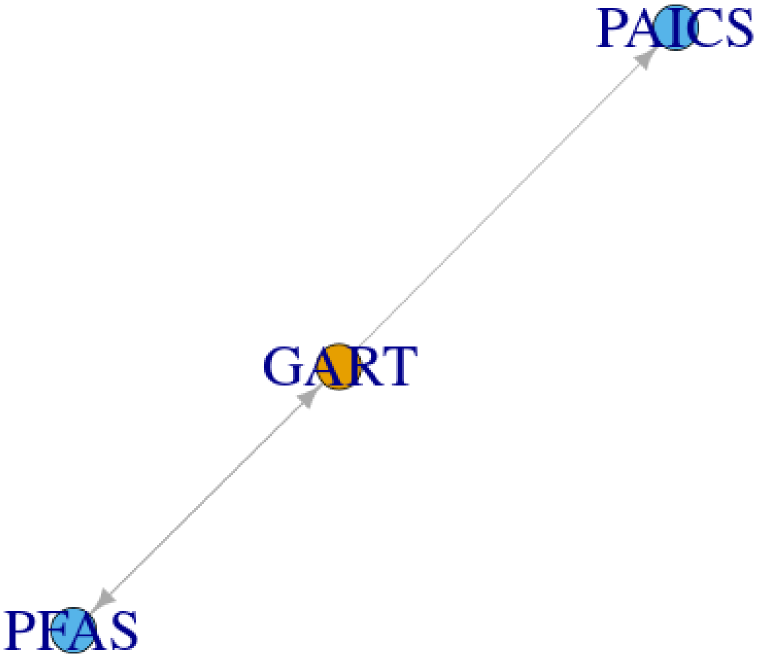

Human KEGG

Fibonacci

Regulators: HSD17B12, TECR

Fiber: ELOVL1, ELOVL2, ELOVL3, ELOVL4, ELOVL5, ELOVL6, ELOVL7

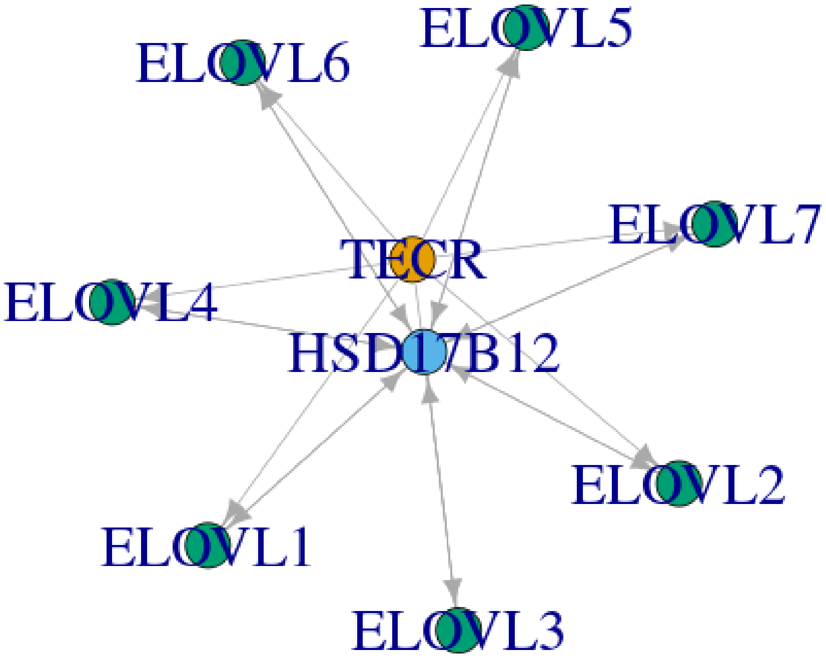

Human KEGG

Fibonacci

Regulators: ABL1, BCR, EGFR, INSR, MET, RAPGEF1, SH2B2

Fiber: CBLB, CBLC

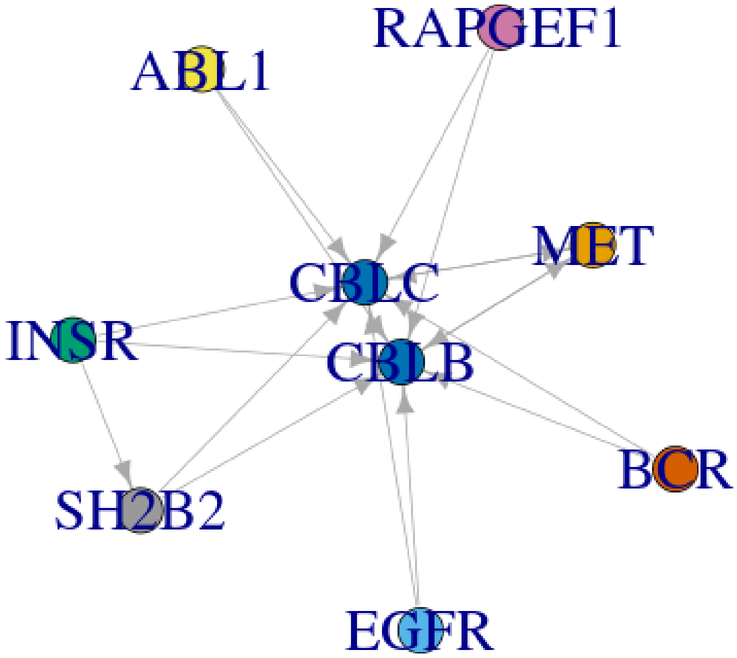

Human KEGG

Fibonacci

Regulators: PIGL

Fiber: GPLD1, PIGA, PIGC, PIGH, PIGP, PIGQ, PIGY

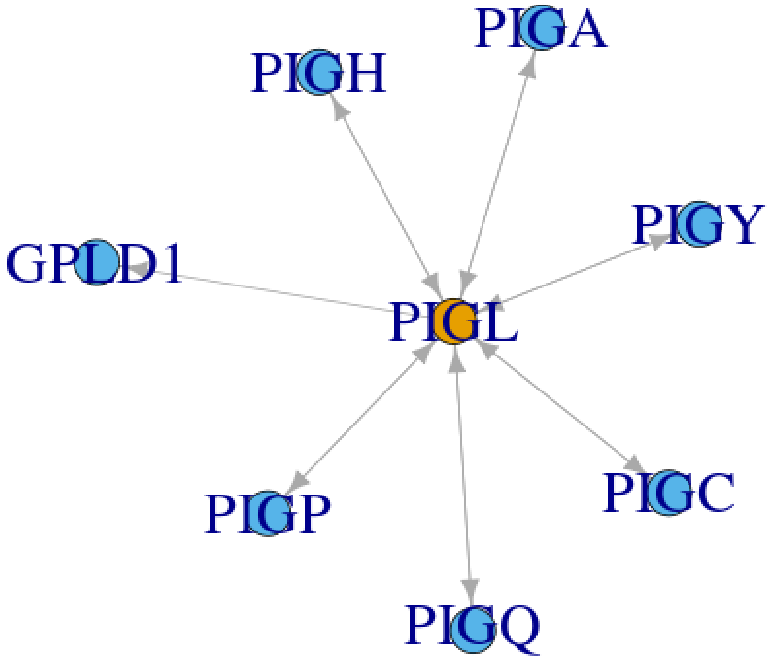

Human KEGG

Fibonacci

Regulators: GLO1, LDHD

Fiber: HAGH, HAGHL

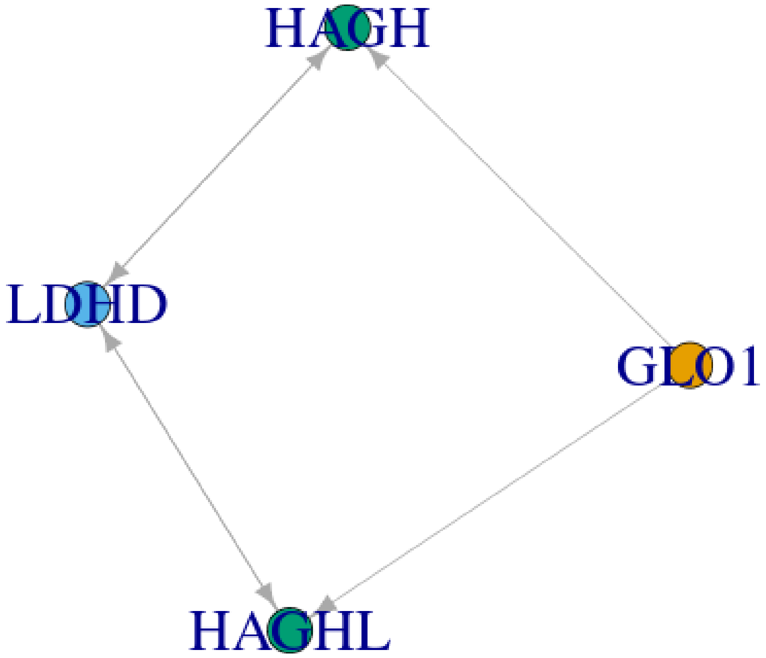

Human KEGG

Fibonacci

Regulators: PIGV, PIGW

Fiber: PIGM, PIGX

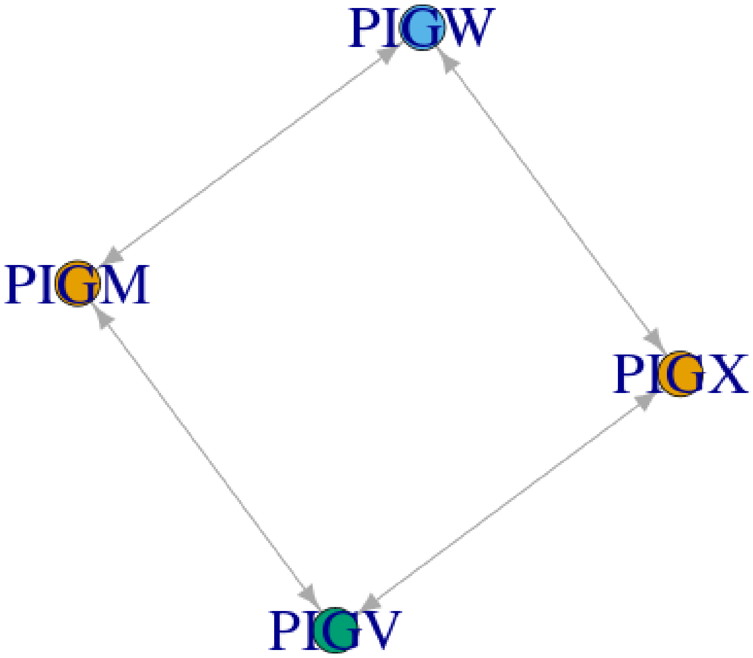

Human KEGG

Fibonacci

Regulators: MAPK1

Fiber: CLK3, DUSP16, NCOA1, PITPNM1, RGS19

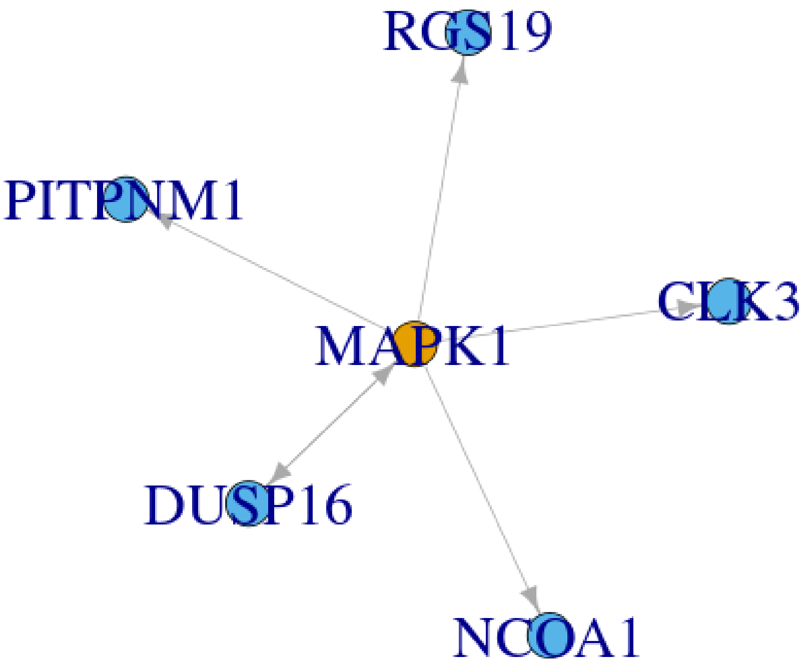

Human KEGG

Fibonacci

Regulators: A4GALT, B3GALNT1, B3GALT4, B3GALT5, B4GALNT1, CHIA, CHIT1, GBGT1, GLA, GLB1, GNS, NAGA, NAGK, RENBP

Fiber: HEXA, HEXB

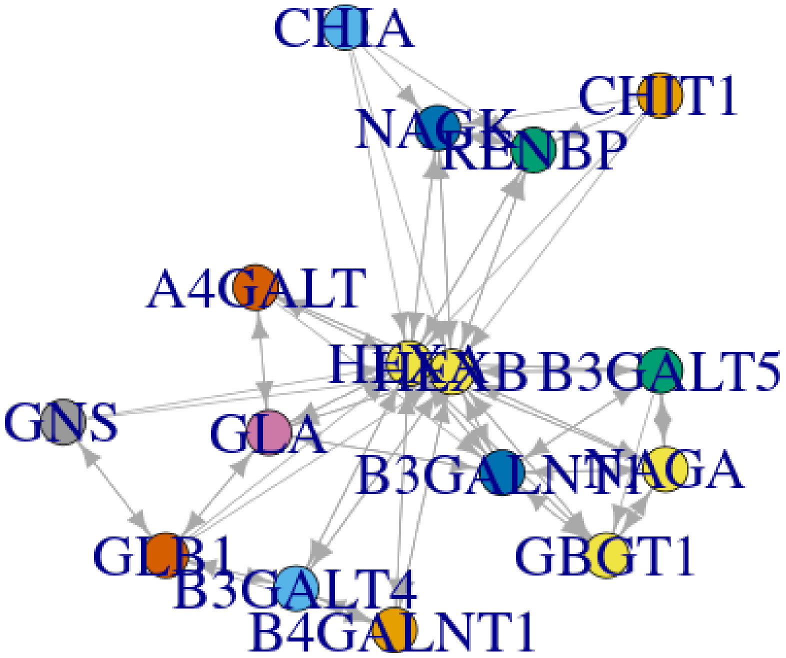

Human KEGG

Fibonacci

Regulators: AOX1, PDXP, PHOSPHO2

Fiber: PDXK, PNPO

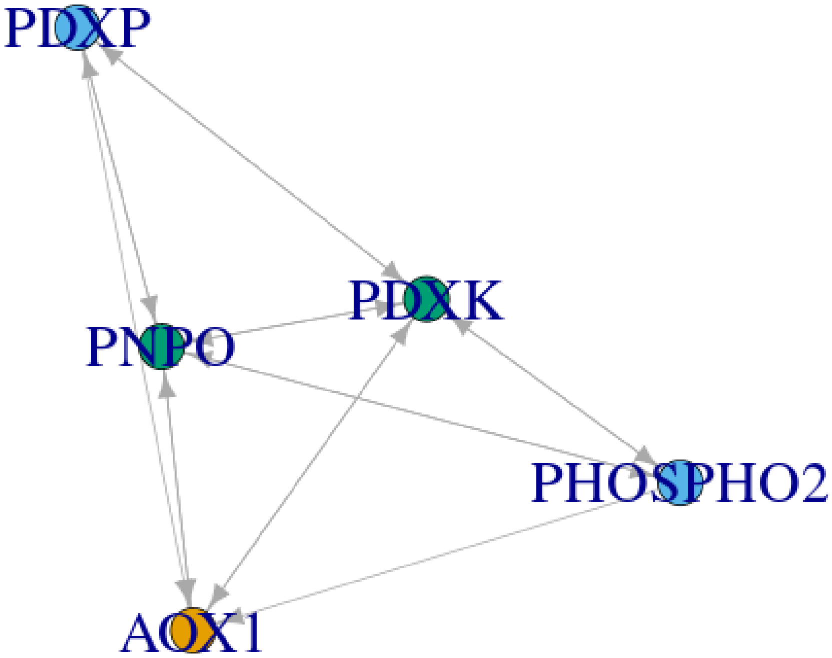

Human KEGG

Fibonacci

Regulators: EXTL2, EXTL3

Fiber: EXT1, EXT2

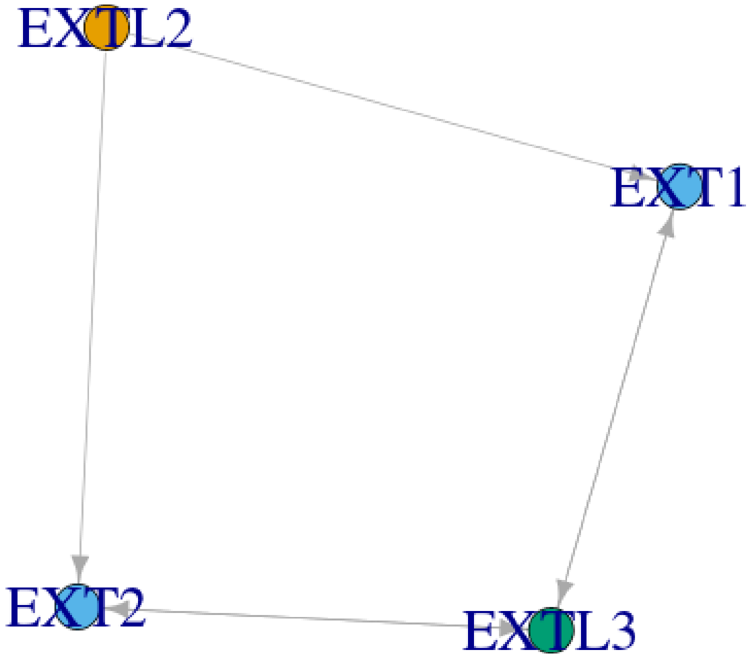

Human KEGG

Fibonacci

Regulators: CREBBP, EP300, HDAC1, HDAC2, HDAC3, KAT2A, KAT2B, MED1, MED12, MED12L, MED13, MED13L, MED14, MED16, MED17, MED24, MED27, MED30, MED4, NCOA1, NCOA2, NCOA3, NCOR1, PAX8, PPARG, SIN3A

Fiber: RXRB, RXRG

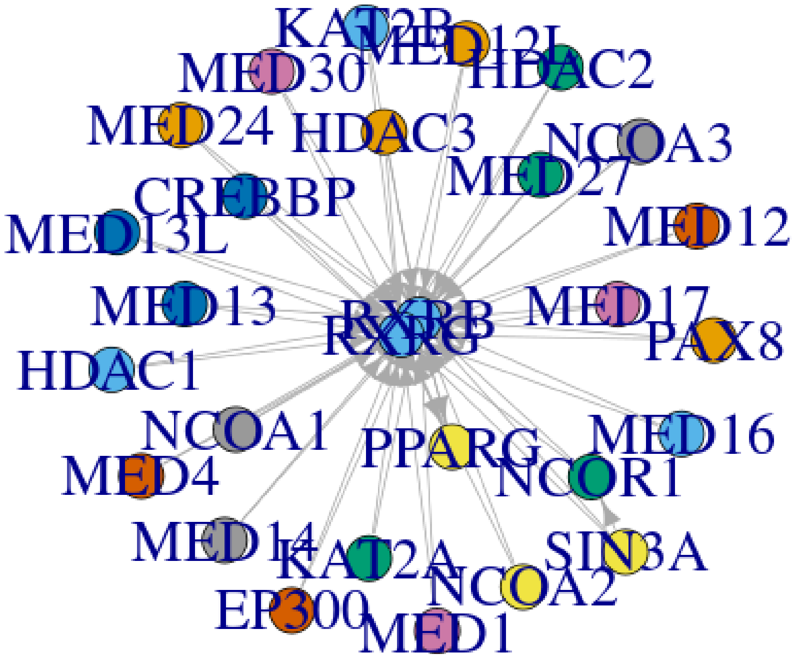

Human KEGG

Fibonacci

Regulators: GCH1, PTS

Fiber: ALPI, ALPL, ALPP, ALPPL2

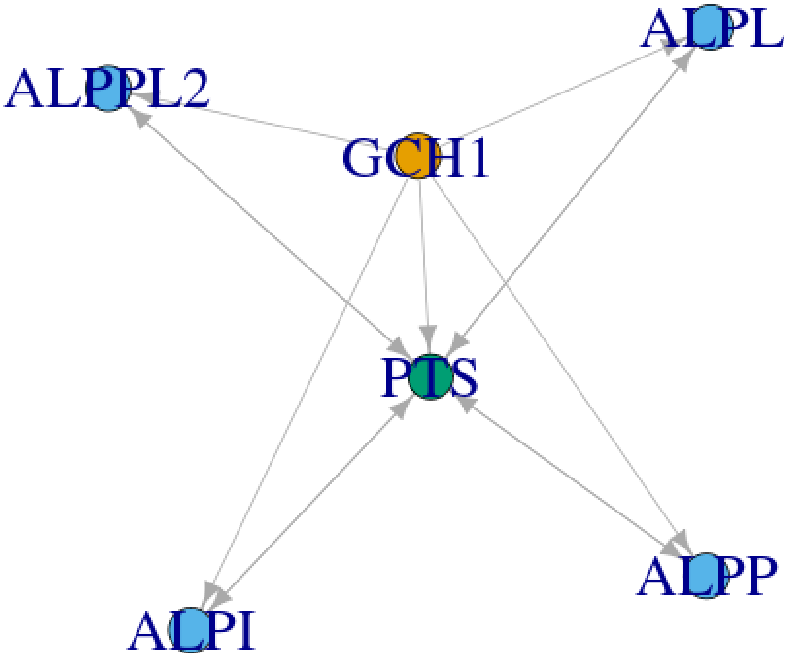

Human KEGG

Fibonacci

Regulators: AGXT, AGXT2

Fiber: GPT, GPT2

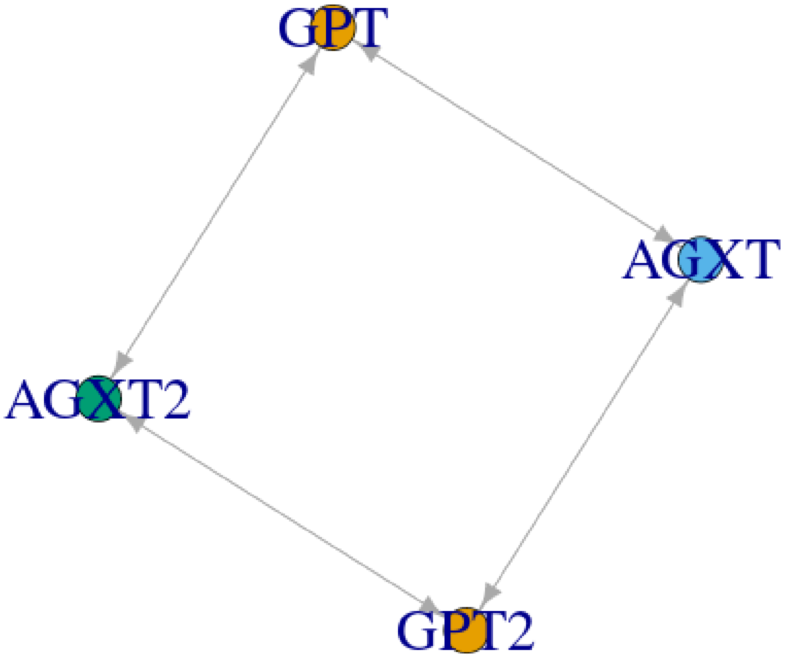

Human KEGG

Fibonacci

Regulators: MGAT2

Fiber: FUT8, MGAT3

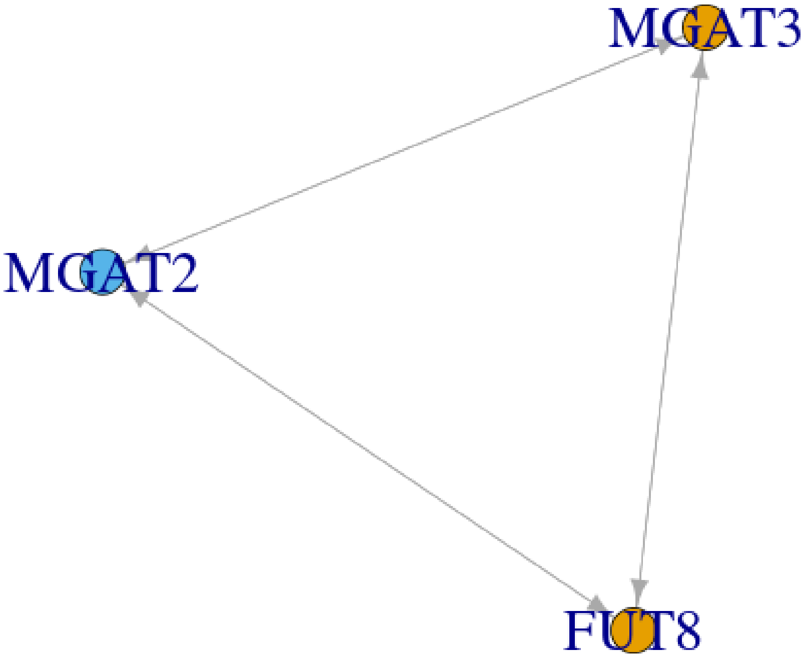

Human KEGG

Fibonacci

Regulators: MTR

Fiber: MARS, MARS2

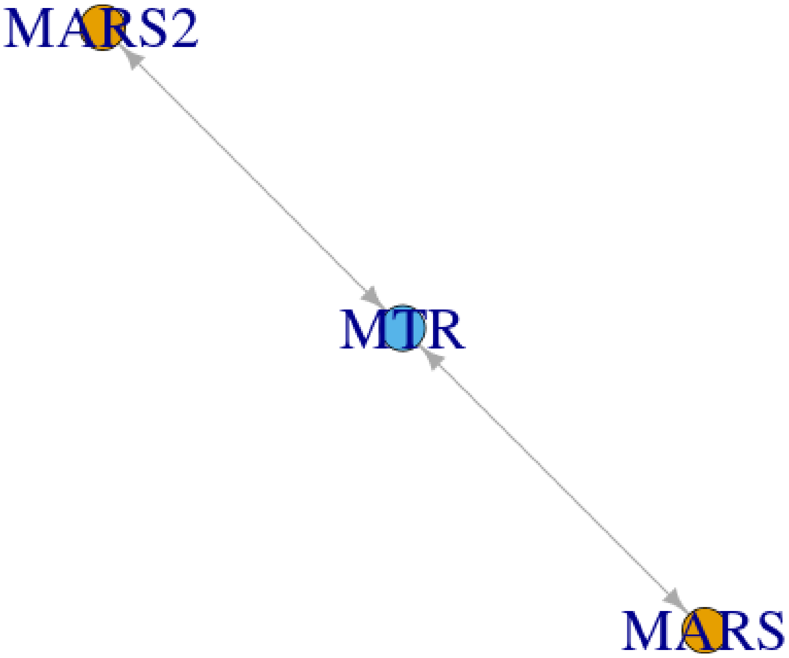

Human KEGG

Fibonacci

Regulators: ALG1, DPAGT1

Fiber: ALG13, ALG14

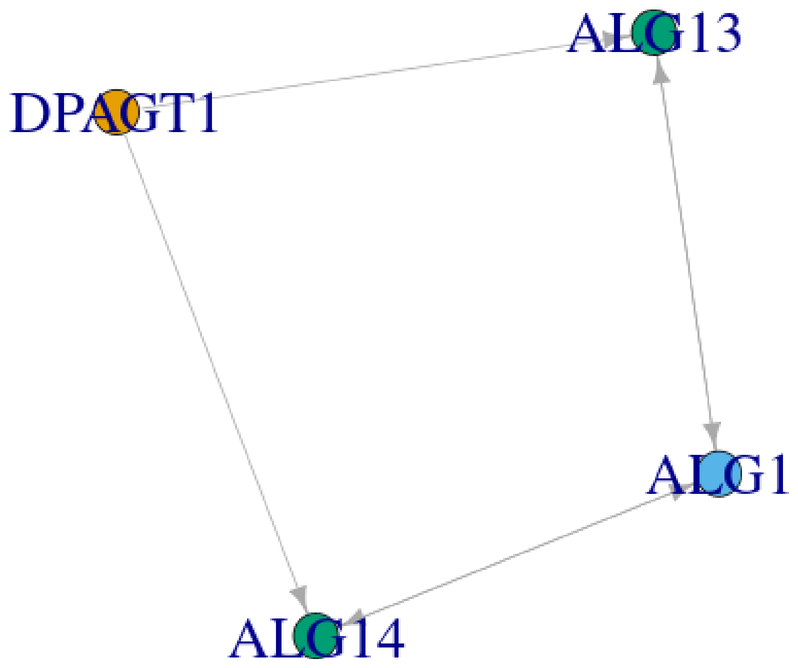

Human KEGG

Fibonacci

Regulators: MGAT1, MGAT2

Fiber: MAN2A1, MAN2A2

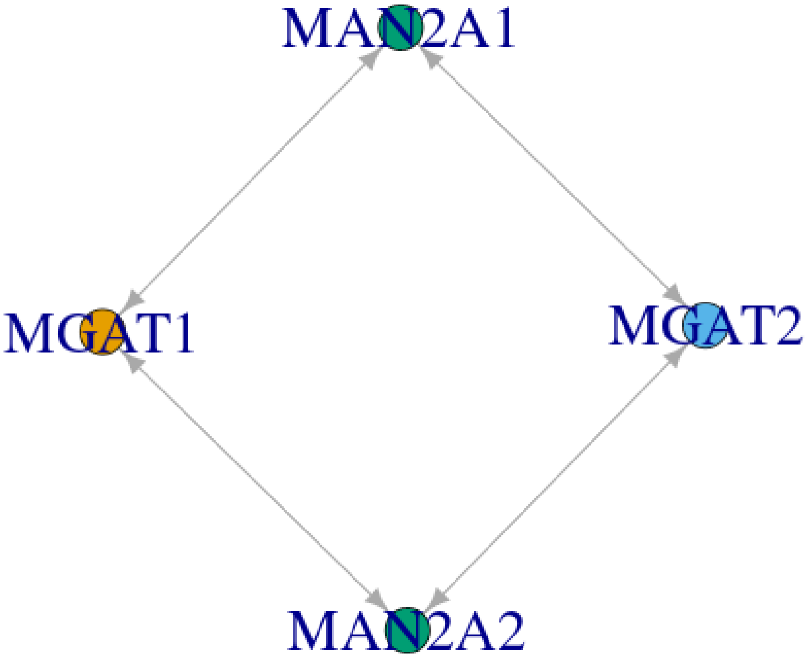

Human KEGG

Fibonacci

Regulators: ARNTL, CLOCK, NPAS2

Fiber: BHLHE40, BHLHE41, NR1D1, RORB

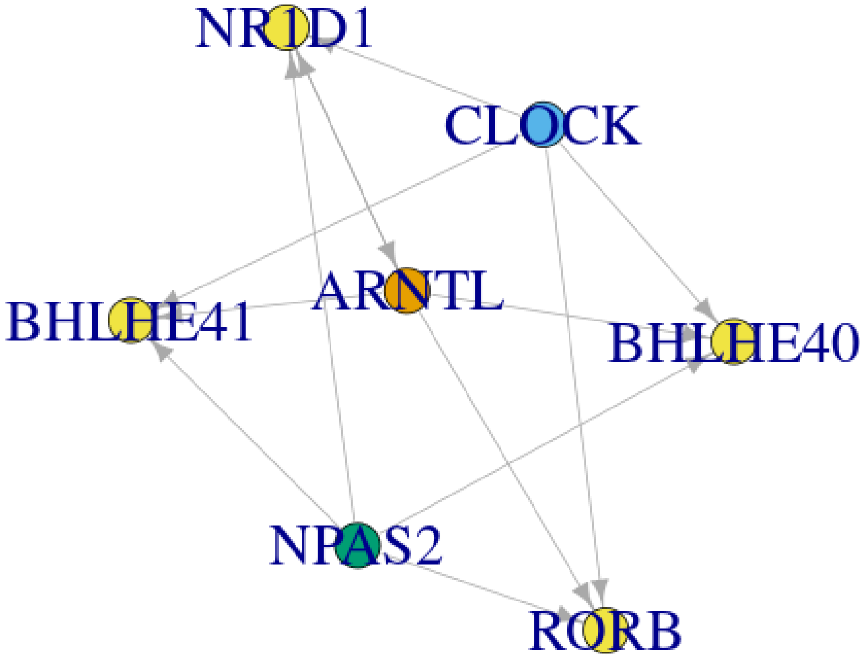

Human KEGG

Fibonacci

Regulators: AGL, GBE1

Fiber: AMY1A, AMY1B, AMY1C, AMY2A, AMY2B

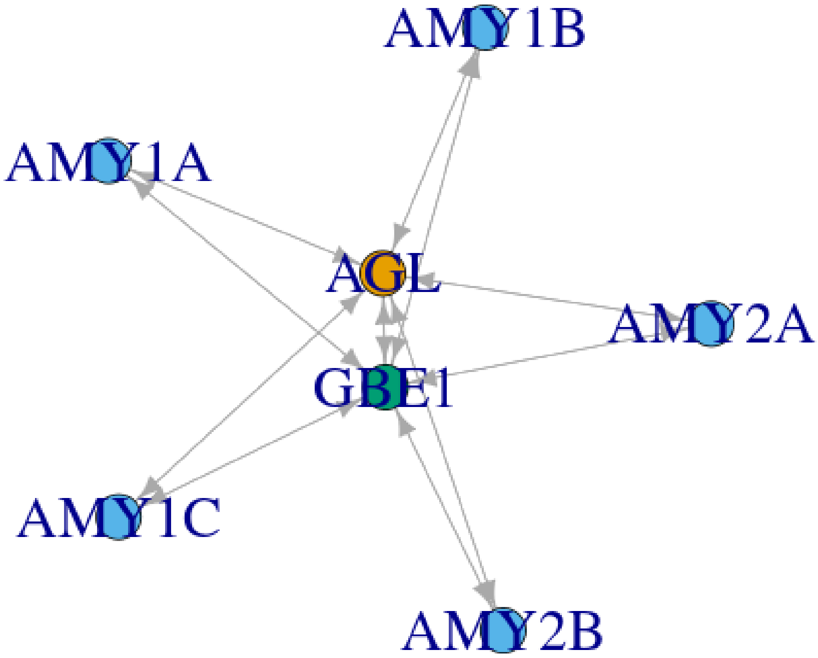

Human KEGG

Fibonacci

Regulators: ALDH4A1, LAP3, LOC102724788, PRODH, PRODH2, PYCR1, PYCR2, PYCRL

Fiber: P4HA1, P4HA2, P4HA3

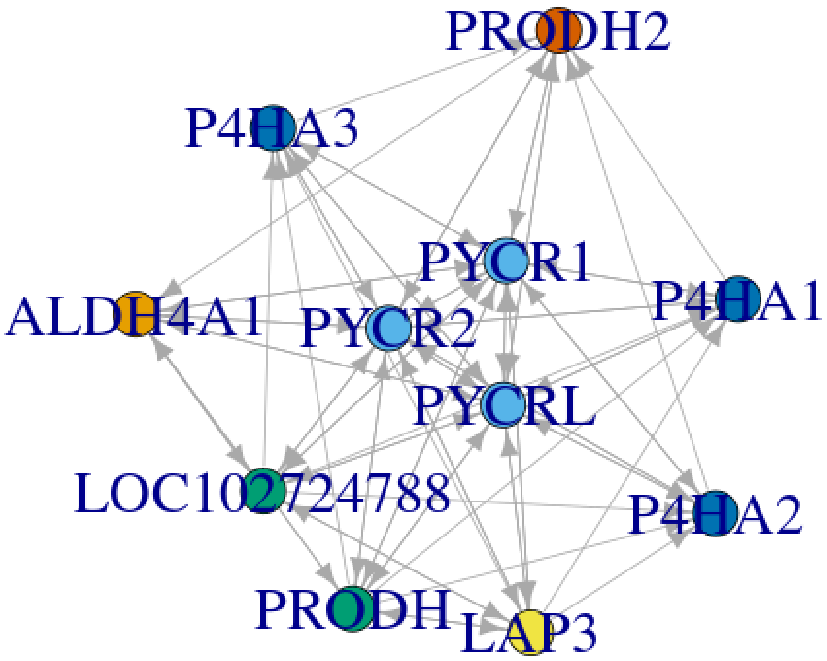

Human KEGG

Fibonacci

Regulators: AKR1C3, HPGDS, PTGDS, PTGES3, PTGIS, PTGS1, PTGS2, TBXAS1

Fiber: PTGES, PTGES2

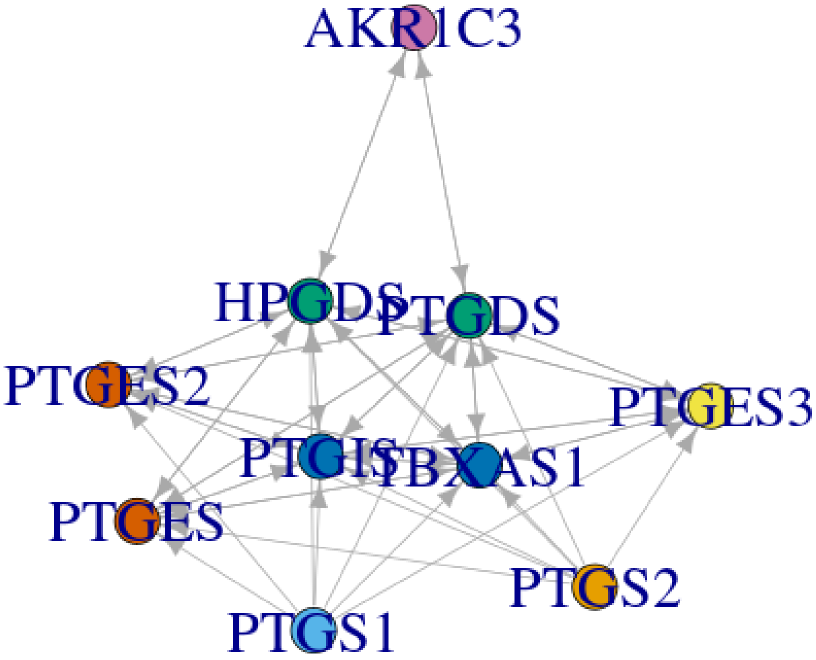

Human KEGG

Fibonacci

Regulators: A4GALT, B3GALNT1, B3GALT4, B3GALT5, B4GALNT1, CHIA, CHIT1, GLA, GLB1, GNS, HEXA, HEXB, NAGK, RENBP

Fiber: GBGT1, NAGA

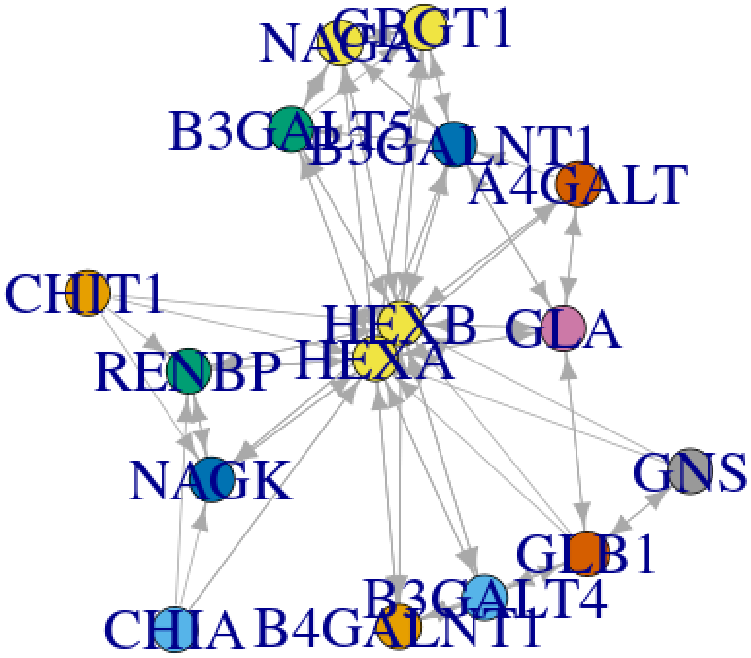

Human KEGG

Fibonacci

Regulators: GANAB, MGAT1

Fiber: MAN1A1, MAN1A2, MAN1B1, MAN1C1

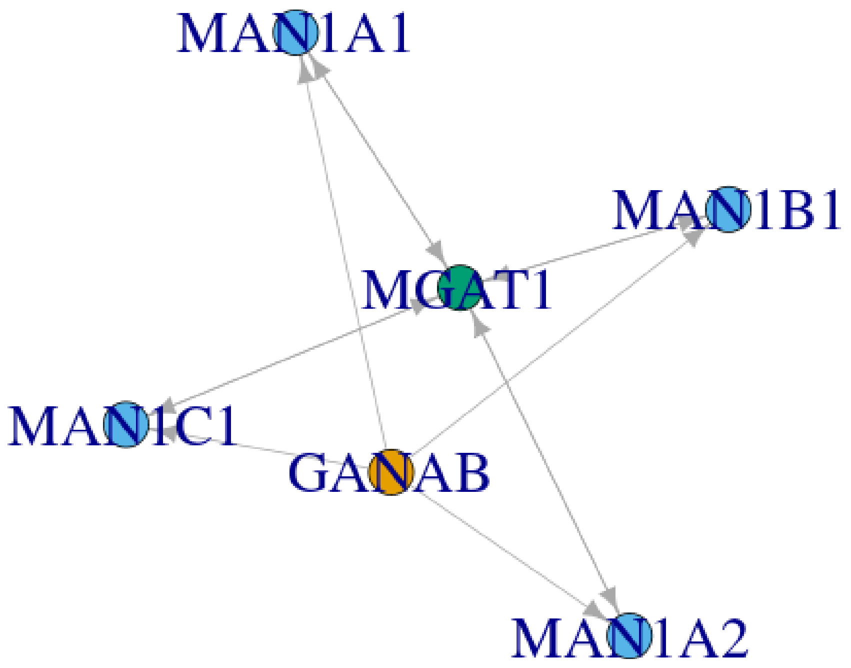

Human KEGG

Fibonacci

Regulators: AGXT, AGXT2, AHCY, AHCYL1, AHCYL2, ALAS1, ALAS2, ALDH1L1, ALDH1L2, AMT, ATIC, BHMT, CBS, DHFR, DHFRL1, DMGDH, DNMT1, DNMT3A, DNMT3B, FTCD, GART, GATM, GCAT, GCSH, GLDC, GNMT, LOC102724560, MTFMT, MTHFD1, MTHFD1L, MTHFD2, MTHFD2L, MTHFR, MTHFS, MTR, PIPOX, PSPH, SARDH, SDS, SDSL, SHMT1, SHMT2, SRR, ST20-MTHFS, TYMS

Fiber: FPGS, GGH

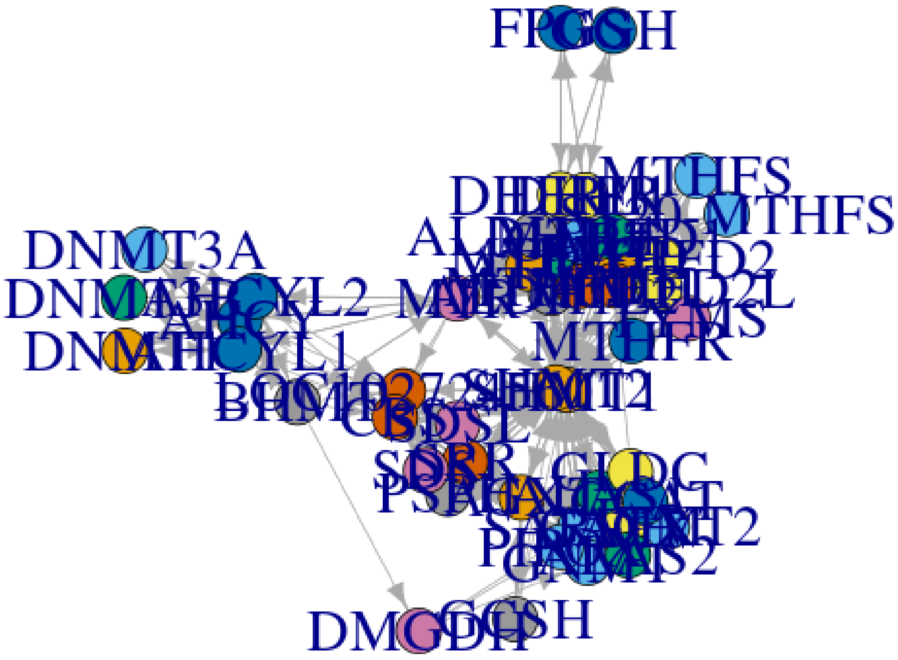

Human KEGG

Fibonacci

Regulators: ALDH4A1, LAP3, LOC102724788, P4HA1, P4HA2, P4HA3, PRODH, PRODH2

Fiber: PYCR1, PYCR2, PYCRL

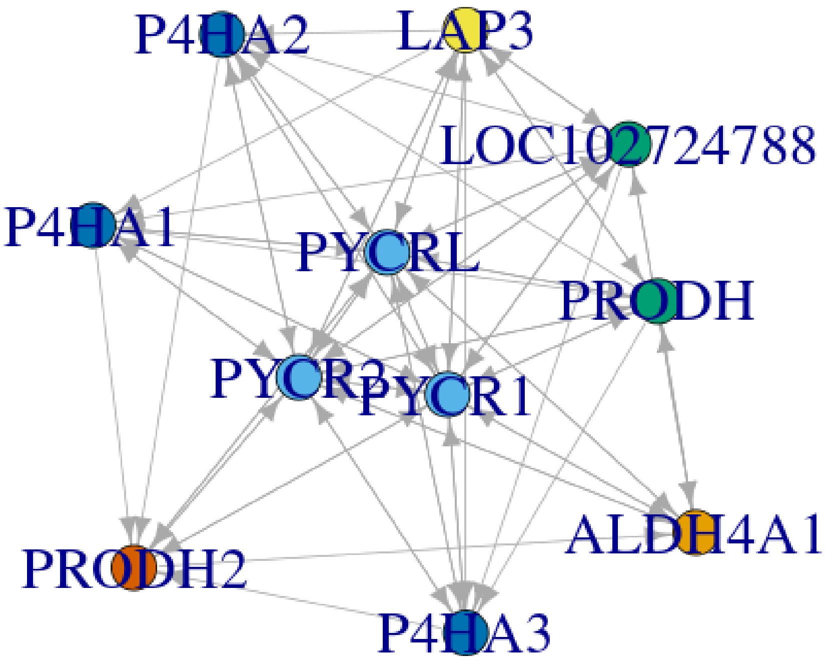

Human KEGG

Fibonacci

Regulators: FUT8, MGAT2, MGAT3, MGAT5, MGAT5B

Fiber: MGAT4A, MGAT4B, MGAT4D

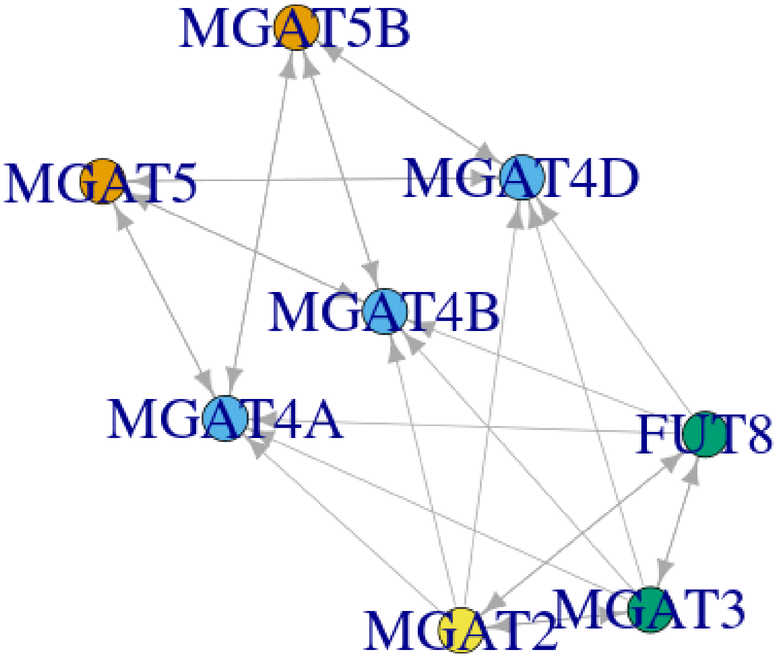

Human KEGG

Fibonacci

Regulators: LIPT2, NQO1

Fiber: GGCX, LIAS, LIPT1, VKORC1

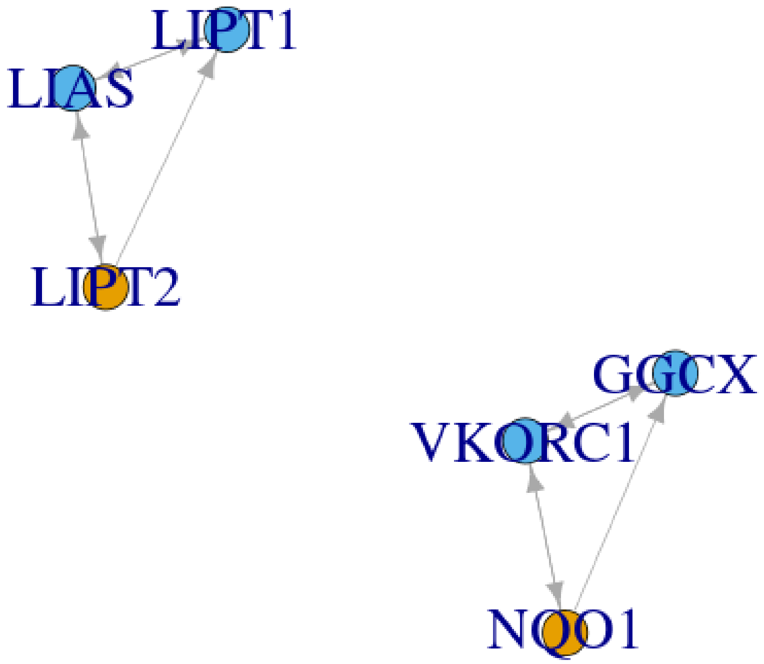

Human KEGG

Fibonacci

Regulators: CREBBP, CSNK1A1, CSNK1A1L, CTBP1, CTBP2, CTNNB1, EP300, GSK3B, JUP, LEF1, NLK, RUVBL1, SMAD4, SOX17, TCF7, TCF7L1, TCF7L2, TEAD1, TEAD2, TEAD3, TEAD4, YAP1

Fiber: AXIN1, AXIN2

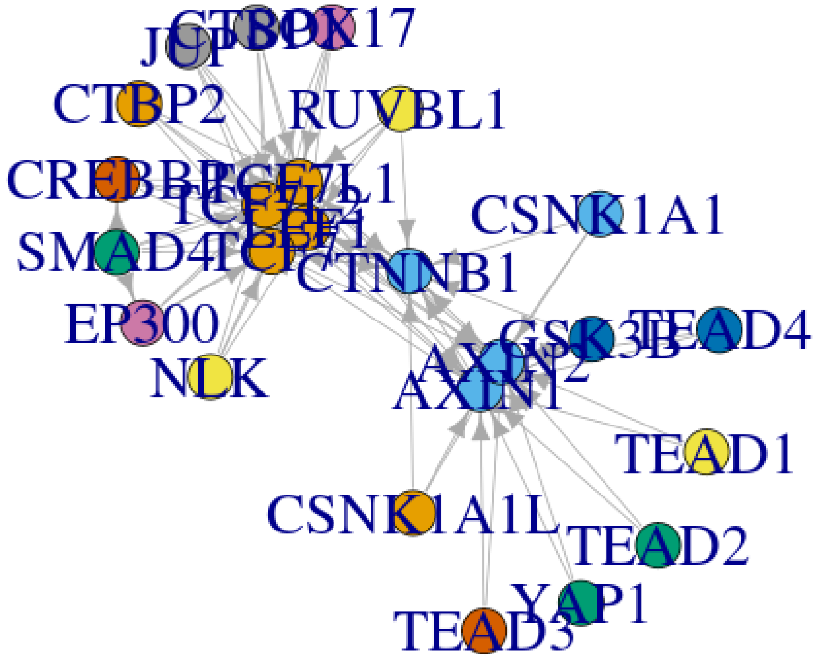

Human KEGG

Fibonacci

Regulators: FASN, OXSM

Fiber: MCAT, OLAH

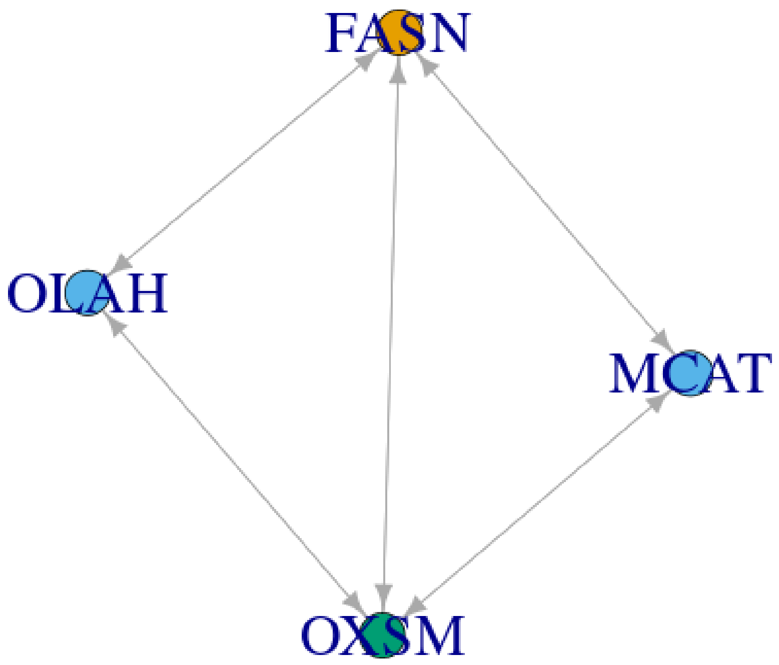

Human KEGG

Fibonacci

Regulators: AGXT, AGXT2, AHCY, AHCYL1, AHCYL2, ALAS1, ALAS2, ALDH1L1, ALDH1L2, AMT, ATIC, BHMT, DHFR, DHFRL1, DMGDH, DNMT1, DNMT3A, DNMT3B, FPGS, FTCD, GART, GATM, GCAT, GCSH, GGH, GLDC, GNMT, MTFMT, MTHFD1, MTHFD1L, MTHFD2, MTHFD2L, MTHFR, MTHFS, MTR, PIPOX, PSPH, SARDH, SDS, SDSL, SHMT1, SHMT2, SRR, ST20-MTHFS, TYMS

Fiber: CBS, LOC102724560

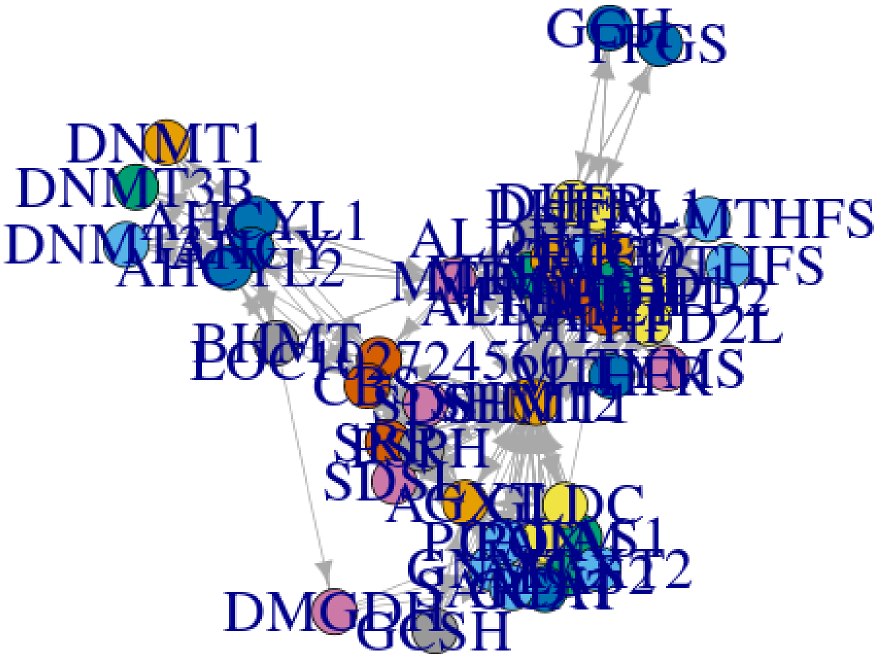

Human KEGG

Fibonacci

Regulators: BDNF, GRB2, NGF, NTF3, NTF4

Fiber: NTRK1, NTRK2

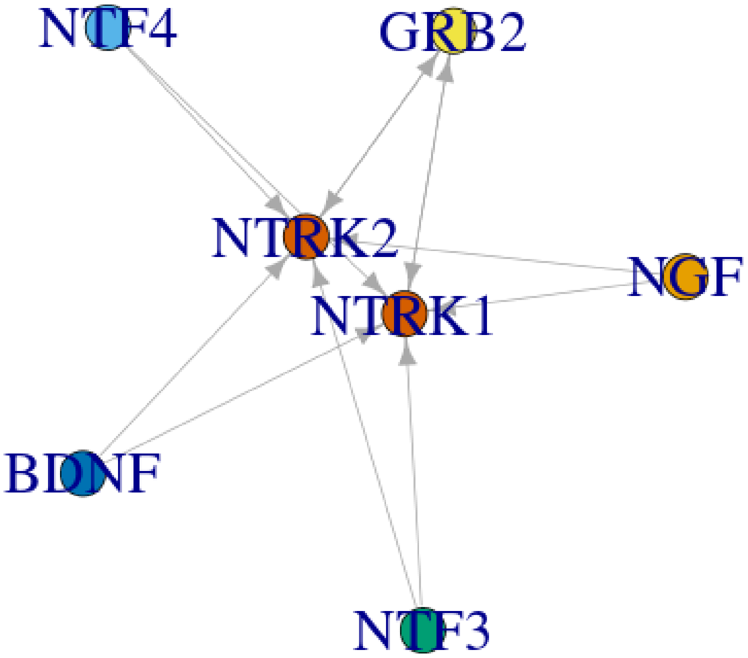

Human KEGG

Fibonacci

Regulators: ABO, B3GALT1, B3GALT2, B3GALT4, B3GALT5, B3GNT3, B3GNT4, B3GNT5, B4GALT1, B4GALT2, B4GALT3, B4GALT4, FUT1, FUT2, FUT3, FUT4, FUT5, FUT6, FUT7, FUT9, GCNT2, GLB1, ST3GAL1, ST3GAL2, ST3GAL3, ST3GAL4, ST3GAL6, ST6GALNAC1, ST6GALNAC3, ST6GALNAC4, ST6GALNAC6, ST8SIA1, ST8SIA5

Fiber: B3GNT2, B4GAT1

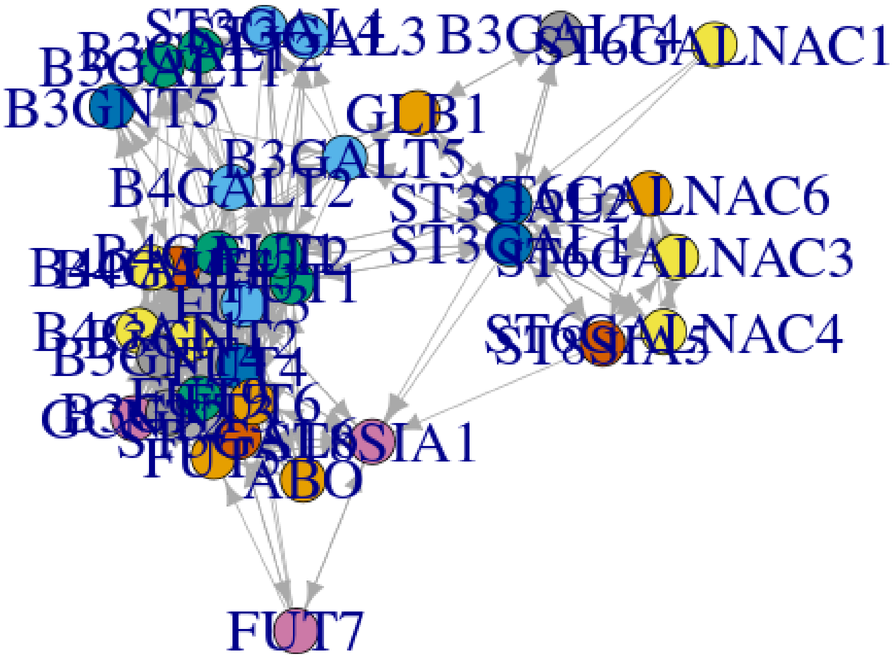

Human KEGG

Fibonacci

Regulators: AGXT, AGXT2, AHCY, AHCYL1, AHCYL2, ALAS1, ALAS2, ALDH1L1, ALDH1L2, AMT, ATIC, BHMT, CBS, DHFR, DHFRL1, DMGDH, DNMT1, DNMT3A, DNMT3B, FPGS, FTCD, GART, GATM, GCAT, GCSH, GGH, GLDC, GNMT, LOC102724560, MTFMT, MTHFD1, MTHFD1L, MTHFD2, MTHFD2L, MTHFR, MTR, PIPOX, PSPH, SARDH, SDS, SDSL, SHMT1, SHMT2, SRR, TYMS

Fiber: MTHFS, ST20-MTHFS

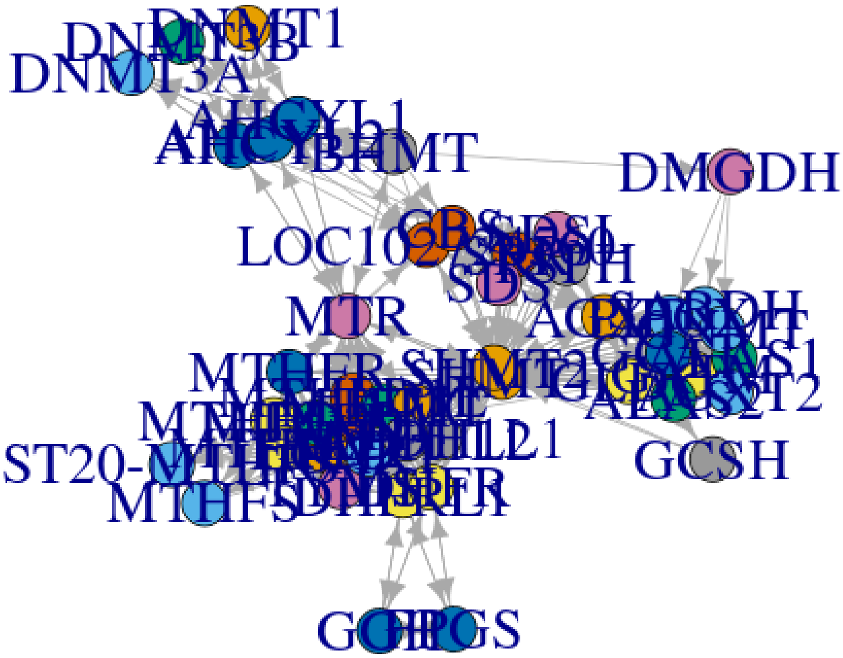

Human KEGG

Fibonacci

Regulators: ALDH4A1, LAP3, P4HA1, P4HA2, P4HA3, PRODH2, PYCR1, PYCR2, PYCRL

Fiber: LOC102724788, PRODH

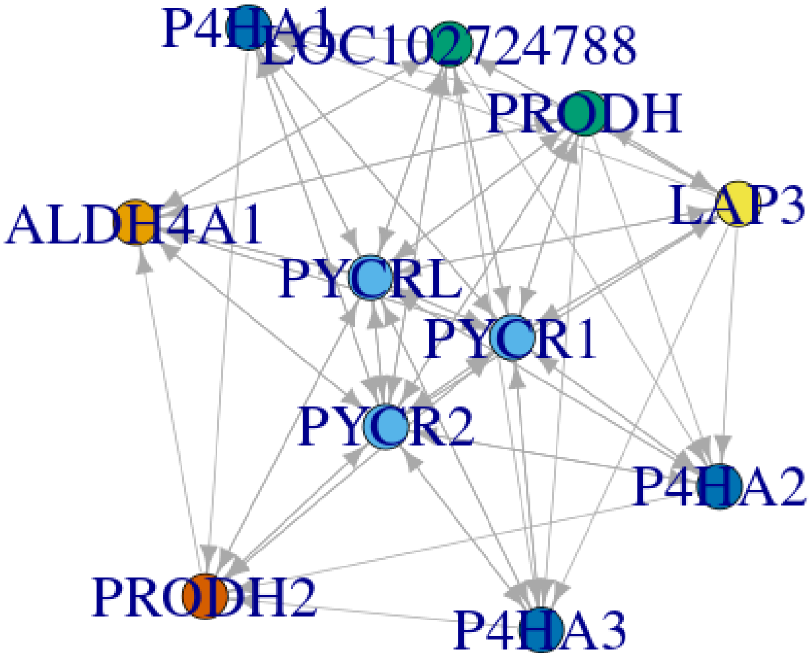

Human KEGG

Fibonacci

Regulators: GGCX, LIAS, LIPT1, VKORC1

Fiber: LIPT2, NQO1

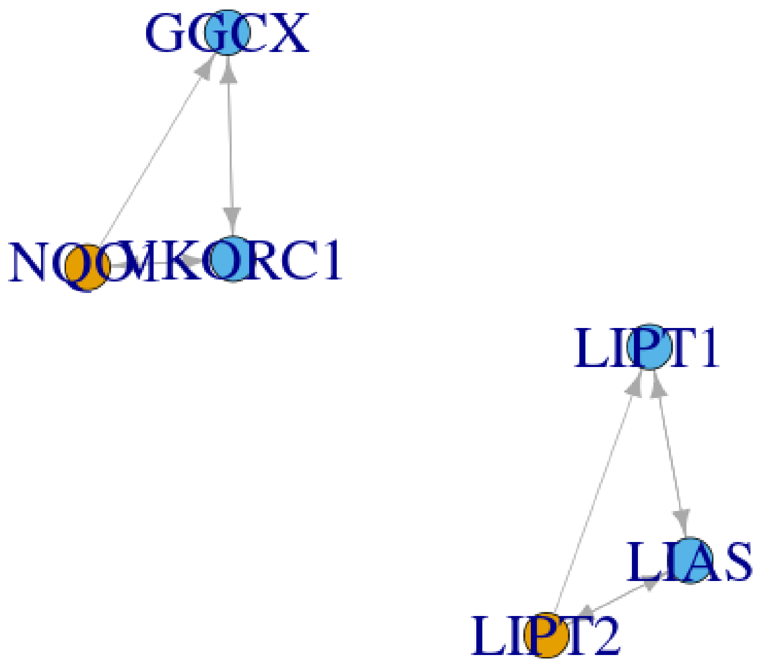

Human KEGG

Fibonacci

Regulators: FOLH1, NAT8L

Fiber: RIMKLA, RIMKLB

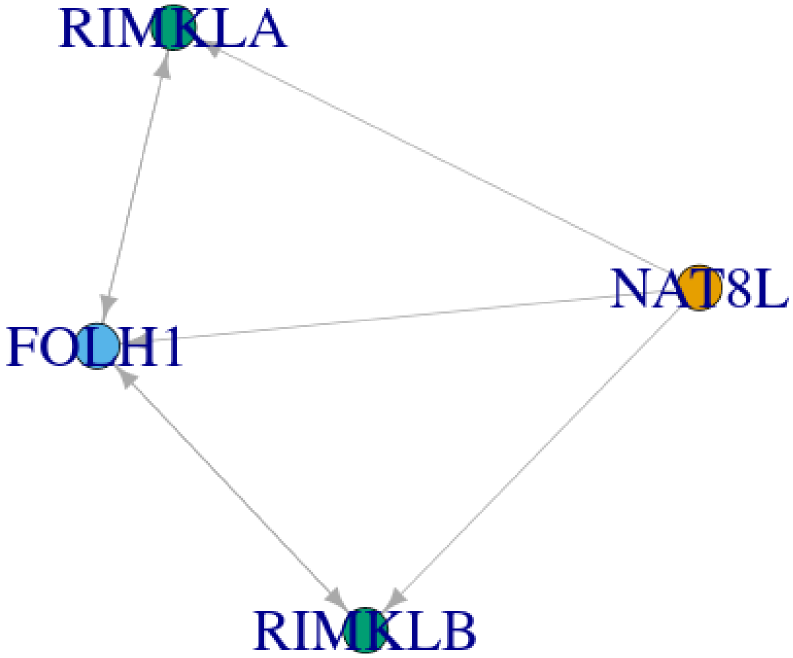

Human KEGG

n = 2

Regulators:

Fiber: GATB, GATC, NARS, NARS2, QARS, QRSL1

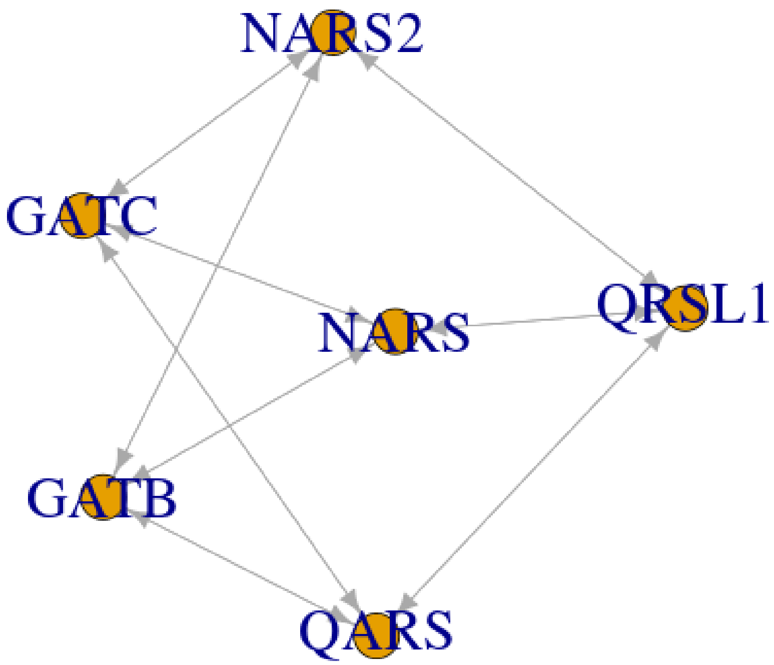

Human KEGG

n = 2

Regulators: FH

Fiber: SDHA, SDHB, SDHC, SDHD

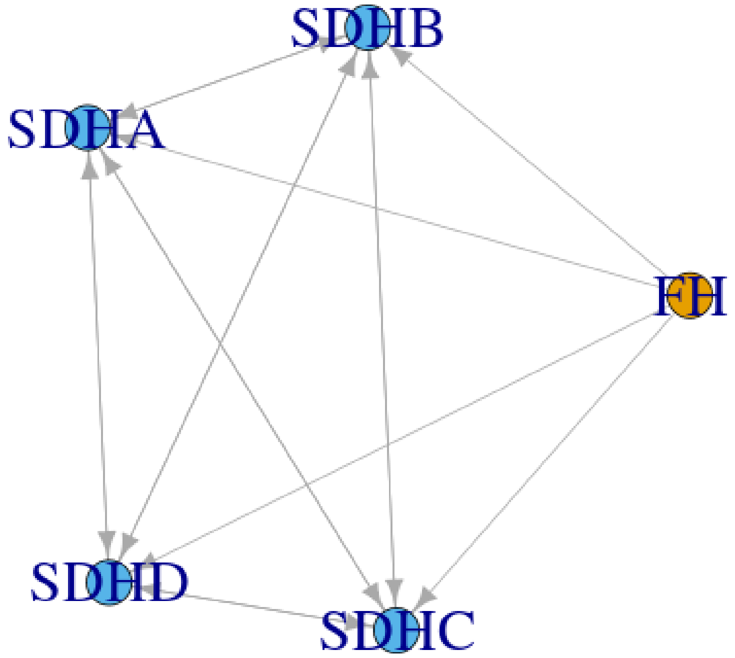

Supplement: S2 File — In S2 File we present a list of circuits found in different species. (PDF) [file pcbi.1007776.s002.pdf]
